# Supplementary material for: A simple and versatile nickel platform for the generation of branched high molecular weight polyolefins
Source: Nat Commun. 2020 Jan 17;11:372. doi: 10.1038/s41467-019-14211-0 (PMC6969022; doi:10.1038/s41467-019-14211-0)
Supplement: Supplementary file 1 — Supplementary Information [file 41467_2019_14211_MOESM1_ESM.pdf]

Supplementary Information

A Simple and Versatile Nickel Platform for the Generation of Branched High Molecular Weight Polyolefins

Changle Chen et al.

Table of Contents

Supplementary Methods 2

Supplementary Figures 11

Supplementary Tables...112

Supplementary References...115

Supplementary Methods

General Procedure for the Synthesis of ligand and Ni complexes.

Spectral data for the $[BAF]^-$ counterion. The ^1H NMR resonances of the $[B\text{-}(3,5\text{-}C_6H_3(CF_3)_3)_4]^-$ anion were the same for the different cationic (DAB)Pd complexes and are not repeated in the spectral data reported for each of the cationic complexes: ^1H NMR ($CDCl_3$, 400MHz): δ 7.75 (s, 8H, *Hp* BAF), 7.53 (s, 4H, *Ho* BAF). ^{13}C NMR ($CDCl_3$, 100 MHz): δ 162.24 (q, J_{CB} = 49.5 Hz, Cipso), 135.3 (Co). 129.88 (q, J_{CF} = 31.7 Hz, Cm), 126.43 (q, J_{CF} = 272.1 Hz, CF_3), 117.97 (m, Cp).

3-(2,6-diisopropylphenylimino)-butan-2-one,[1] (2,6-dibenzhydryl-4-methylphenylimino)butanone,[2] 4,4'-(9,9-Dimethylxanthene-4,5-diyl)-bis(2,6-diisopropylaniline)[3] and 1-methoxy-4-(prop-1-yn-1-yl)benzene[4] were prepared according to literature procedures.

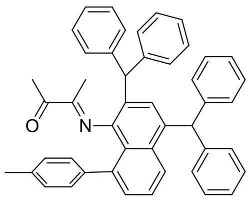

Preparation of L3. A solution of 2,4-dibenzhydryl-8-(p-tolyl)naphthalen-1-amine (1.13 g, 2 mmol), 2,3-butadione (344 mg, 4 mmol) and p-toluenesulfonic acid (20 mg) in toluene (20 mL) was stirred at 80 °C for 24 h, until there was one main point on the TLC plate. The solvent was evaporated under reduced pressure. The remaining mixture was diluted in methanol (30 mL) and stirred for 1 h. The yellow solid was isolated by filtration (887 mg, 70 %). ^1H NMR (400 MHz, $CDCl_3$) δ 8.00 (d, *J* = 8.3 Hz, 1H), 7.38 - 7.32 (m, 1H), 7.24 - 6.97 (m, 21H), 6.90 - 6.82 (m, 2H), 6.76 (d, *J* = 6.6 Hz, 2H), 6.65 (s, 1H), 6.21 (s, 1H, C*H*Ph_2), 5.41 (s, 1H, C*H*Ph_2), 2.33 (s, 3H, COCH_3), 1.99 (s, 3H, aryl-CH_3), 0.61 (s, 3H, N=C*Me*). ^{13}C NMR (100 MHz, $CDCl_3$) δ 198.91 (s, *C*OCH_3), 166.53 (s, N=*C*Me), 144.07 (s), 143.55 (s), 143.35 (s), 142.13 (s), 141.65 (s), 139.20 (s), 136.25 (s), 135.51 (s), 132.23 (s), 130.70 (s), 130.45 (s), 129.85 (s), 129.48 (s), 129.22 (s), 128.90 (s), 128.47 (s), 128.43 (s), 128.32 (s), 128.06 (s), 127.65 (s), 126.40 (s), 126.30 (s), 126.08 (s), 125.18 (s), 124.18 (s), 122.96 (s), 53.41 (s, *C*HPh_2), 52.24 (s, *C*HPh_2), 24.03 (s), 21.09 (s), 14.75 (s, N=C*Me*). HRMS (m/z): Calcd for: $C_{47}H_{40}ON$: $[M+H]^+$ 634.3104, Found: 634.3103.

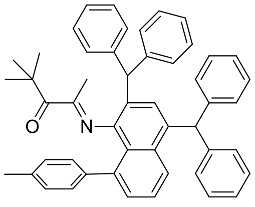

Preparation of L4. A solution of 1-tert-butyl-1-propyne (960 mg, 10 mmol), 0.01 eq $RuCl_3$ (20.7 mg, 0.1 mmol) and 3 equiv of $PhI(OAc)_2$ (9660 mg, 30 mmol) in DCM (40 ml) and water (10 mL) was stirred at 30 °C for 5 h. The mixture was then separated

with separatory funnel and the dichloromethane phase was dried over anhydrous Na_2SO_4, filtered and the solvent removed under vacuum. The crude product was purified via a standard silica gel chromatography using hexane/ethyl acetate as eluent to give the 4,4-dimethylpentane-2,3-dione. This method refers to the literature.[5]

4,4-dimethylpentane-2,3-dione has low boiling point, so should be directly used to next step. A solution of 2,4-dibenzhydryl-8-(p-tolyl)naphthalen-1-amine (2.26 g, 4 mmol), 4,4-dimethylpentane-2,3-dione and p-toluenesulfonic acid (20 mg) in toluene (30 mL) was stirred at 130 °C for 72 h, until there was one main point on the TLC plate. The solvent was evaporated under reduced pressure. The remaining mixture was diluted in methanol (40 mL) and stirred for several hours. The yellow solid was isolated by filtration (1.35 g, 50%). ^{1}H NMR (400 MHz, $CDCl_3$) δ 7.97 (d, *J* = 7.5 Hz, 1H), 7.39 - 7.01 (m, 20H), 6.95 (s, 3H), 6.84 (d, *J* = 17.4 Hz, 3H), 6.71 (s, 1H), 6.18 (s, 1H, C*H*Ph_2), 5.74 (s, 1H, C*H*Ph_2), 2.32 (s, 3H, aryl-C*H*$_3$), 1.09 (s, 3H, N=C*Me*), 1.02 (s, 9H, C(C*H*$_3$)$_3$). ^{13}C NMR (100 MHz, $CDCl_3$) δ 205.44 (s, *C*OCH_3), 168.37 (s, N=*C*Me), 144.84 (s), 144.05 (s), 143.50 (s), 142.44 (s), 141.40 (s), 139.77 (s), 135.71 (s), 135.49 (s), 132.35 (s), 131.32 (s), 130.66 (s), 130.53 (s), 129.52 (s), 129.47 (s), 129.07 (s), 128.49 (s), 128.33 (s), 128.13 (s), 128.07 (s), 126.67 (s), 126.60 (s), 126.42 (s), 126.28 (s), 126.25 (s), 126.11 (s), 125.26 (s), 123.92 (s), 123.70 (s), 53.40 (s, *C*HPh_2), 51.22 (s, *C*HPh_2), 43.60 (s), 27.55 (s), 21.17 (s), 19.24 (s, N=C*Me*). HRMS (m/z): Calcd for: $C_{50}H_{46}ON$: $[M+H]^+$ 676.3574, Found: 676.3570.

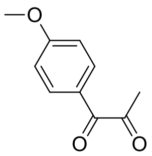

A solution of 1-methoxy-4-(prop-1-yn-1-yl)benzene (1460 mg, 10 mmol), 0.01 eq $RuCl_3$ (20.7 mg, 0.1 mmol) and 3 equiv of $PhI(OAc)_2$ (9660 mg, 30 mmol) in DCM (40 ml) and water (10 mL) was stirred at 30℃ for 5 h. The mixture was then separated with separatory funnel and the dichloromethane phase was dried over anhydrous Na_2SO_4, filtered and the solvent removed under vacuum. The crude product was purified via a standard silica gel chromatography using petroleum ether /ethyl acetate as eluent to give the 1-(4-methoxyphenyl)propane-1,2-dione (1420 mg, 80%). The NMR spectra of the compound agree well with literature report.[6]

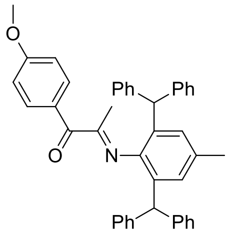

Preparation of L5. Similar procedure as ligand **L6** was employed except 1-(4-methoxyphenyl)propane-1,2-dione (712 mg, 4 mmol) was used. **L5** was obtained as a yellow powder (720 mg, 60%). ^{1}H NMR (400 MHz, $CDCl_3$) δ 7.90 (d, *J* = 8.8 Hz, 2H), 7.32 - 7.16 (m, 13H), 7.04 (dd, *J* = 10.9, 7.7 Hz, 8H), 6.74 (d, *J* = 8.8 Hz, 2H), 6.67 (s,

2H), 5.31 (s, 2H, C*H*Ph$_2$), 3.84 (s, 3H, O*Me*), 2.15 (s, 3H, aryl-C*H$_3$*), 1.08 (s, 3H, N=C*Me*). ^{13}C NMR (100 MHz, CDCl$_3$) δ 190.21 (s, *C*OCH$_3$), 170.45 (s), 163.57 (s), 144.71 (s), 143.67 (s), 142.64 (s), 133.28 (s), 132.56 (s), 131.28 (s), 129.96 (s), 129.65 (s), 129.22 (s), 128.55 (s), 128.26 (s), 127.69 (s), 126.55 (s), 126.28 (s), 113.53 (s), 55.46 (s), 51.92 (s), 21.40 (s, aryl-*C*H$_3$), 17.46 (s, N=C*Me*). HRMS (m/z): Calcd for: $C_{43}H_{38}O_2N$: $[M+H]^+$ 600.2897, Found: 600.2894.

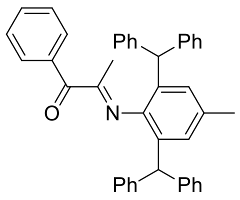

Preparation of L6. A solution of 2,6-Bis(diphenylmethyl)-4-methylaniline (879 mg, 2 mmol), 1-phenylpropane-1,2-dione (592 mg, 4 mmol) and p-toluenesulfonic acid (20 mg) in toluene (20 mL) was stirred at 120 °C for 48 h, until there was one main point on the TLC plate. The solvent was evaporated under reduced pressure. The remaining mixture was diluted in methanol (30 mL) and stirred for 1 h. The yellow solid was isolated by filtration (854 mg, 75 %). ^{1}H NMR (400 MHz, CDCl$_3$) δ 7.83 (d, *J* = 7.4 Hz, 2H), 7.48 (t, *J* = 7.4 Hz, 1H), 7.37 - 7.14 (m, 15H), 7.03 (dd, *J* = 12.0, 7.4 Hz, 7H), 6.67 (s, 2H), 5.28 (s, 2H, C*H*Ph$_2$), 2.15 (s, 3H, aryl-C*H$_3$*), 1.06 (s, 3H, N=C*Me*). ^{13}C NMR (100 MHz, CDCl$_3$) δ 191.89 (s, *C*OCH$_3$), 169.96 (s, N=*C*Me), 144.66 (s), 143.55 (s), 142.58 (s), 135.13 (s), 132.90 (s), 132.67 (s), 131.16 (s), 130.75 (s), 129.94 (s), 129.62 (s), 129.20 (s), 128.59 (s), 128.28 (s), 128.19 (s), 126.58 (s), 126.33 (s), 51.99 (s, *C*HPh$_2$), 21.42 (s, aryl-*C*H$_3$), 17.18 (s, N=C*Me*). HRMS (m/z): Calcd for: $C_{42}H_{36}ON$: $[M+H]^+$ 570.2791, Found: 570.2789.

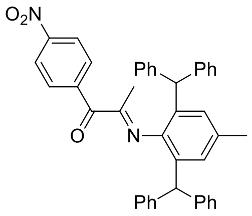

Preparation of L7. Similar procedure as ligand **L6** was employed except 1-(4-nitrophenyl)propane-1,2-dione (772 mg, 4 mmol) was used. **L7** was obtained as a yellow powder (860 mg, 70%). ^{1}H NMR (400 MHz, CDCl$_3$) δ 7.98 (d, *J* = 8.8 Hz, 2H), 7.66 (d, *J* = 8.8 Hz, 2H), 7.30 - 7.22 (m, 12H), 6.97 (d, *J* = 7.2 Hz, 8H), 6.63 (s, 2H), 5.10 (s, 2H, C*H*Ph$_2$), 2.15 (s, 3H, aryl-C*H$_3$*), 1.26 (s, 3H, N=C*Me*). ^{13}C NMR (100 MHz, CDCl$_3$) δ 190.18 (s, *C*OCH$_3$), 168.28 (s), 149.67 (s), 144.22 (s), 142.98 (s), 142.46 (s), 140.66 (s), 133.22 (s), 131.66 (s), 130.63 (s), 129.84 (s), 129.50 (s), 129.16 (s), 128.66 (s), 128.40 (s), 126.72 (s), 126.54 (s), 122.94 (s), 52.33 (s, *C*HPh$_2$), 21.40 (s, aryl-*C*H$_3$), 16.38 (s, N=C*Me*). HRMS (m/z): Calcd for: $C_{42}H_{35}O_3N_2$: $[M+H]^+$ 615.2642, Found: 615.2643.

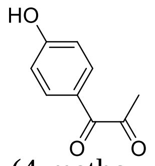

1-(4-methoxyphenyl)propane-1,2-dione (3560 mg, 20 mmol) was heated to reflux for 24 h in a mixture of aqueous HBr (48%) and glacial acetic acid (40 mL, 50/50). The reaction mixture was cooled by a bath of ice-water and extracted several times with ethyl acetate. All ethyl acetate was washed several times with water, dried with $MgSO_4$ and the solvent was removed under reduced pressure. The residue was purified by column chromatography (petroleum ether /ethyl acetate) giving a pure yellowish solid (1968 mg, 60%). The NMR spectra of the compound agree well with literature report.[7]

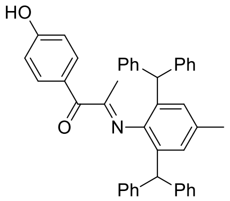

Preparation of L-OH. A solution of 2,6-Bis(diphenylmethyl)-4-methylaniline (1758 mg, 4 mmol), 1-(4-hydroxyphenyl)propane-1,2-dione (328 mg, 2 mmol) and p-toluenesulfonic acid (20 mg) in toluene (20 mL) was stirred at 80 °C for 12 h under nitrogen. The reaction was cooled to room temperature and a yellow solid precipitated. The yellow solid was isolated by filtration (585 mg, 50%). ^{1}H NMR (400 MHz, $CDCl_3$) δ 7.52 (br, 2H), 7.35 - 6.75 (m, 20H), 6.66 (br, 2H), 6.45 (br, 2H), 5.29 (s, 2H, C*H*Ph_2), 2.13 (s, 3H, aryl-C*H*$_3$), 1.15 (s, 3H, N=C*Me*). ^{13}C NMR (100 MHz, [D$_6$]DMSO) δ 189.82 (s, *C*OCH_3), 170.44 (s), 162.52 (s), 144.26 (s), 143.07 (s), 142.63 (s), 133.24 (s), 131.76 (s), 131.03 (s), 129.52 (s), 129.21 (s), 128.75 (s), 128.53 (s), 128.35 (s), 126.49 (s), 126.38 (s), 125.45 (s), 115.11 (s), 51.17 (s, *C*HPh_2), 21.02 (s), 17.40 (s). HRMS (m/z): Calcd for: $C_{42}H_{36}O_2N$: $[M+H]^+$ 586.2741, Found: 586.2742.

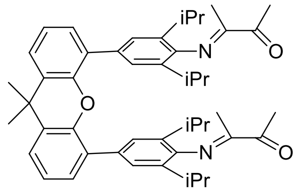

Preparation of L-L. A solution of 4,4'-(9,9-Dimethylxanthene-4,5-diyl)-bis(2,6-diisopropylaniline) (2240 mg, 4 mmol), 2,3-butadione (1720 mg, 20 mmol) and p-toluenesulfonic acid (20 mg) in toluene (20 mL) was stirred at 25 °C for 12 h. The solvent was evaporated under reduced pressure. The remaining mixture was diluted in methanol (30 mL) and stirred for 1 h. The yellow solid was isolated by filtration (1948 mg, 70%). ^{1}H NMR (400 MHz, $CDCl_3$) δ 7.44 (dd, J = 7.5, 1.8 Hz, 2H), 7.25 (s, 4H), 7.22 - 7.10 (m, 4H), 2.56 (s, 6H), 2.43 - 2.31 (m, 4H, C*H*(CH_3)$_2$), 1.81 (s, 6H), 1.80 (s, 6H), 1.05 - 0.80 (m, 24H, CH(C*H*$_3$)$_2$). ^{13}C NMR (100 MHz, $CDCl_3$) δ 200.31 (s), 166.42 (s), 147.02 (s), 144.21 (s), 134.48 (s), 133.85 (s), 130.58 (s), 130.38 (s), 130.31 (s), 125.40 (s), 124.39 (s), 122.93 (s), 34.36 (s), 34.00 (s), 28.42 (s), 25.01 (s), 22.97 (s),

22.84 (s), 15.41 (s). HRMS (m/z): Calcd for: $C_{47}H_{57}N_2O_3$: $[M+H]^+$ 697.4364, Found: 697.4362.

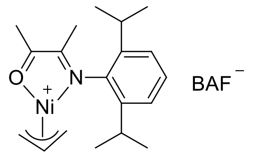

Preparation of Ni1. A mixture of 3-(2,6-diisopropylphenylimino)-butan-2-one (123 mg, 0.50 mmol), $[Ni(allyl)Cl]_2$ (67.5 mg, 0.5 mmol), and NaBAF (443 mg, 0.5 mmol) in 15 mL CH_2Cl_2 was stirred at room temperature for 12 h. The resulting mixture was filtrated over celite and evaporated to get a black red solid (513 mg, 85%). ^{1}H NMR (400 MHz, $CDCl_3$) δ 7.71 (s, 8H), 7.54 (s, 4H) 7.37 - 7.30 (m, 1H), 7.28 - 7.22 (m, 2H), 5.86 - 5.68 (m, 1H, allyl), 2.67 (br, 2H, C*H*(CH_3)$_2$), 2.51 (s, 3H, COC*H*$_3$), 2.03 (s, 3H, N=C*Me*), 1.23 (d, *J* = 6.6 Hz, 6H, CH(C*H*$_3$)$_2$), 1.05 (d, *J* = 6.4 Hz, 6H, CH(C*H*$_3$)$_2$). ^{13}C NMR (100 MHz, $CDCl_3$) δ 211.26 (s, *C*OCH$_3$), 172.69 (s, N=*C*Me), 141.26 (s), 136.93 (s), 129.85 (s), 125.00 (s), 120.43 (s, allyl), 29.30 (s, *C*H(CH_3)$_2$), 26.98 (s, CO*C*H$_3$), 23.52 (s, CH(*C*H$_3$)$_2$), 23.40 (s, CH(*C*H$_3$)$_2$), 18.19 (s, N=C*Me*). Key ^{1}H-^{13}C HSQC correlations ($CDCl_3$): 120.43 (s, allyl)/5.82 - 5.71 (m, 1H, allyl), 29.30 (s, *C*H(CH_3)$_2$)/2.67 (br, 2H, C*H*(CH_3)$_2$), 26.98 (s, CO*C*H$_3$)/2.51 (s, 3H, COC*H*$_3$), 23.52 (s, CH(*C*H$_3$)$_2$)/1.23 (d, J = 6.6 Hz, 6H, CH(C*H*$_3$)$_2$), 23.40 (s, CH(*C*H$_3$)$_2$)/1.05 (d, *J* = 6.4 Hz, 6H, CH(C*H*$_3$)$_2$, 18.19 (s, N=C*Me*)/ 2.03 (s, 3H, N=C*Me*). Anal. Calcd for $C_{51}H_{40}BF_{24}NNiO$: C, 50.69; H, 3.34; Found: C, 50.56; H, 3.37.

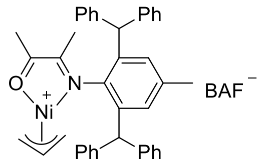

Preparation of Ni2. Similar procedure as catalyst **Ni1** was employed except **L2** (253 mg, 0.50 mmol) was used. **Ni2** was obtained as a black red powder (661 mg, 90%). ^{1}H NMR (400 MHz, $CDCl_3$) δ 7.72 (s, 8H), 7.52 (s, 4H).7.42 - 7.26 (m, 12H), 6.99 (d, *J* = 25.9 Hz, 8H), 6.83 (s, 2H), 5.58 - 5.46 (m, 1H, allyl), 5.23 (br, 2H, C*H*Ph$_2$), 2.23 (s, 3H), 2.20 (s, 3H), 0.86 (s, 3H, N=C*Me*). ^{13}C NMR (100 MHz, $CDCl_3$) δ 211.05 (s, *C*OCH$_3$), 174.64 (s, N=*C*Me), 141.06 (s), 140.25 (s), 140.06 (s), 138.84 (s), 133.80 (s), 129.69 (s), 129.42 (s), 129.40 (s), 129.36 (s), 127.89 (s), 127.87 (s), 120.20 (s, allyl), 53.25 (s, *C*HPh$_2$), 17.25 (s, N=C*Me*). Key ^{1}H-^{13}C HSQC correlations ($CDCl_3$): 120.20 (s, allyl)/5.59 - 5.46 (m, 1H, allyl), 53.25 (s, *C*HPh$_2$)/ 5.23 (br, 2H, C*H*Ph$_2$), 17.25 (s, N=C*Me*)/0.87 (s, 3H, N=C*Me*). Anal. Calcd for $C_{72}H_{50}BF_{24}NNiO$: C, 58.80; H, 3.43; Found: C, 58.72; H, 3.48.

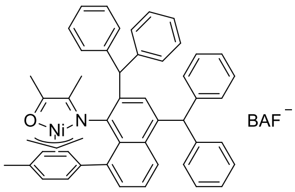

Preparation of Ni3. Similar procedure as catalyst **Ni1** was employed except **L3** (317 mg, 0.50 mmol) was used. **Ni3** was obtained as a black red powder (687 mg, 86%). ^{1}H NMR (400 MHz, $CDCl_3$) δ 8.18 (d, *J* = 8.1 Hz, 1H), 7.70 (s, 8H), 7.63 (d, *J* = 8.1 Hz, 1H), 7.56 (d, *J* = 9.1 Hz, 1H), 7.51 (s, 4H), 7.34 (d, *J* = 7.3 Hz, 1H), 7.30 - 7.10 (m, 14H), 7.04 (d, *J* = 7.6 Hz, 1H), 6.98 (br, 5H), 6.80 (s, 1H), 6.75 (s, 3H), 6.23 (s, 1H, C*H*Ph_2), 5.98 (br, 1H, allyl), 5.67 (s, 1H, C*H*Ph_2), 3.69 (s, 1H, allyl), 3.42 (s, 1H, allyl), 2.80 - 2.55 (m, 2H, allyl), 2.30 (s, 3H, COCH_3), 2.02 (s, 3H, aryl-CH_3), 0.72 (s, 3H, N=C*Me*). ^{13}C NMR (100 MHz, $CDCl_3$) δ 207.66 (s, *C*OCH_3), 170.22 (s, N=*C*Me), 142.65 (s), 142.49 (s), 142.09 (s), 141.71 (s), 140.31 (s), 139.92 (s), 139.48 (s), 139.42 (s), 136.45 (s), 132.47 (s), 131.46 (s), 131.09 (s), 130.77 (s), 130.44 (s), 129.89 (s), 129.47 (s), 129.41 (s), 129.29 (s), 129.09 (s), 128.86 (s), 128.60 (s), 128.12 (s), 127.86 (s), 127.56 (s), 127.36 (s), 127.08 (s), 127.03 (s), 126.88 (s), 123.02 (s), 116.82 (s, allyl), 59.29 (s, allyl), 57.44 (s, allyl), 53.66 (s, *C*HPh_2), 53.44 (s, *C*HPh_2), 25.73 (s), 20.90 (s), 18.24 (s, N=C*Me*). Key ^{1}H-^{13}C HSQC correlations ($CDCl_3$): 53.66 (s, *C*HPh_2)/6.23 (s, 1H, C*H*Ph_2), 116.82 (s, allyl)/5.98 (br, 1H, allyl), 53.44 (s, *C*HPh_2)/5.67 (s, 1H, C*H*Ph_2), 57.44 (s, allyl)/3.69 (s, 1H, allyl), 59.29 (s, allyl)/3.42 (s, 1H, allyl), 18.24 (s, N=C*Me*)/0.72 (s, 3H, N=C*Me*). Anal. Calcd for $C_{82}H_{56}BF_{24}NNiO$: C, 61.68; H, 3.53; Found: C, 61.55; H, 3.72.

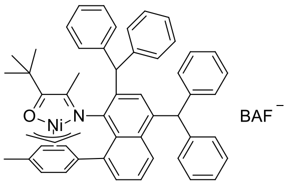

Preparation of Ni4. Similar procedure as catalyst **Ni1** was employed except **L4** (338 mg, 0.50 mmol) was used. **Ni4** was obtained as a red powder (696 mg, 85%). ^{1}H NMR (400 MHz, $CDCl_3$) δ 8.20 (d, *J* = 8.4 Hz, 1H), 7.70 (d, *J* = 17.4 Hz, 9H), 7.50 (d, *J* = 15.0 Hz, 6H), 7.33 (d, *J* = 6.9 Hz, 1H), 7.27 - 7.12 (m, 13H), 7.07 (d, *J* = 7.5 Hz, 1H), 7.04 - 6.96 (m, 4H), 6.87 (s, 1H), 6.72 (dd, *J* = 18.8, 4.0 Hz, 4H), 6.27 (s, 1H, C*H*Ph_2), 6.00 - 5.80 (m, 1H, allyl), 5.58 (s, 1H, C*H*Ph_2), 3.71 (s, 1H, allyl), 3.40 (s, 1H, allyl), 2.74 (d, *J* = 13.0 Hz, 1H, allyl), 2.58 (d, *J* = 13.1 Hz, 1H, allyl), 2.35 (s, 3H, aryl-CH_3), 1.15 (s, 3H, N=C*Me*), 1.04 (s, 9H, C(CH_3)$_3$). ^{13}C NMR (100 MHz, $CDCl_3$) δ 214.78 (s, *C*OC$(CH_3)_3$), 169.07 (s, N=*C*Me), 142.74 (s), 142.46 (s), 142.42 (s), 141.54 (s), 140.68 (s), 140.18 (s), 139.58 (s), 138.77 (s), 136.68 (s), 132.60 (s), 132.02 (s), 131.08 (s), 131.04 (s), 130.62 (s), 130.57 (s), 129.54 (s), 129.33 (s), 129.31 (s), 129.06 (s), 128.88 (s), 128.86 (s), 128.70 (s), 127.69 (s), 127.52 (s), 127.44 (s), 127.09 (s), 127.04 (s), 126.93 (s), 126.38 (s), 126.14 (s), 123.32 (s), 116.49 (s, allyl), 59.47 (s, allyl), 57.57 (s, allyl), 53.65 (s, *C*HPh_2), 52.90 (s, *C*HPh_2), 45.14 (s, *C*$(CH_3)_3$), 27.00 (s, C(*C*H_3)$_3$), 21.40 (s, N=C*Me*), 21.03 (s, aryl-*C*H_3). Key ^{1}H-^{13}C HSQC correlations ($CDCl_3$): 116.49 (s,

allyl)/ 6.00 - 5.80 (m, 1H, allyl), 59.47 (s, allyl)/ 2.58 (d, *J* = 13.1 Hz, 1H, allyl), 59.47 (s, allyl)/ 3.40 (s, 1H, allyl), 57.57 (s, allyl)/ 2.74 (d, *J* = 13.0 Hz, 1H, allyl), 57.57 (s, allyl)/ 3.71 (s, 1H, allyl), 53.65 (s, *C*HPh$_2$)/ 6.27 (s, 1H, C*H*Ph$_2$), 52.90 (s, *C*HPh$_2$)/ 5.58 (s, 1H, C*H*Ph$_2$), 27.00 (s, C(*C*H$_3$)$_3$)/ 1.04 (s, 9H, C(C*H$_3$*)$_3$), 21.40 (s, N=C*Me*)/ 1.15 (s, 3H, N=C*Me*), 21.03 (s, aryl-*C*H$_3$)/ 2.35 (s, 3H, aryl-C*H$_3$*). Anal. Calcd for $C_{85}H_{62}BF_{24}NNiO$: C, 62.29; H, 3.81; Found: C, 62.35; H, 3.70.

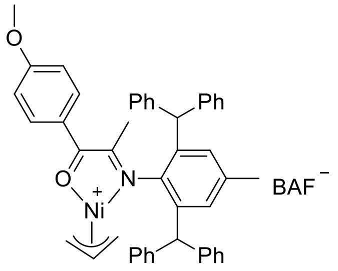

Preparation of Ni5. Similar procedure as catalyst **Ni1** was employed except **L5** (300 mg, 0.50 mmol) was used. **Ni5** was obtained as a red powder (663 mg, 85%). ^{1}H NMR (400 MHz, CDCl$_3$) δ 7.82 (d, *J* = 7.9 Hz, 2H), 7.72 (s, 8H), 7.50 (s, 4H), 7.45 - 7.30 (m, 6H), 7.26 (s, 2H), 7.22 (s, 4H), 7.07 (s, 4H), 6.99 (s, 3H), 6.91 (s, 1H), 6.89 (s, 3H), 5.43 (br, 1H, allyl), 5.34 (s, 2H, C*H*Ph$_2$), 3.87 (s, 3H, O*Me*), 2.26 (s, 3H, aryl-C*H$_3$*), 1.14 (s, 3H, N=C*Me*). ^{13}C NMR (100 MHz, CDCl$_3$) δ 195.10 (s, *C*OPh), 176.76 (s), 169.37 (s), 142.10 (s), 140.61 (s), 140.47 (s), 138.39 (s), 135.36 (s), 133.81 (s), 129.89 (s), 129.54 (s), 129.46 (s), 129.35 (s), 127.84 (s), 127.65 (s), 124.64 (s), 119.33 (s, allyl), 115.94 (s), 56.39 (s, O*Me*), 53.18 (s, *C*HPh$_2$), 21.52 (s, aryl-*C*H$_3$), 20.57 (s, N=C*Me*). Key ^{1}H-^{13}C HSQC correlations (CDCl$_3$): 119.33 (s, allyl)/ 5.43 (br, 1H, allyl), 56.39 (s, O*Me*)/ 3.87 (s, 3H, O*Me*), 53.18 (s, *C*HPh$_2$)/ 5.34 (s, 2H, C*H*Ph$_2$), 21.52 (s, aryl-*C*H$_3$)/ 2.26 (s, 3H, aryl-C*H$_3$*), 20.57 (s, N=C*Me*)/ 1.14 (s, 3H, N=C*Me*). Anal. Calcd for $C_{78}H_{54}BF_{24}NNiO_2$: C, 59.95; H, 3.48; Found: C, 60.11; H, 3.59.

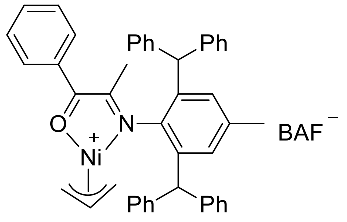

Preparation of Ni6. Similar procedure as catalyst **Ni1** was employed except **L6** (284 mg, 0.50 mmol) was used. **Ni6** was obtained as a red powder (613 mg, 80%). ^{1}H NMR (400 MHz, CDCl$_3$) δ 7.82 - 7.76 (m, 1H), 7.70 (br, 9H), 7.54 - 7.44 (m, 6H), 7.43 - 7.30 (m, 6H), 7.24 (s, 7H), 7.06 (d, *J* = 5.6 Hz, 4H), 6.99 (s, 4H), 6.90 (s, 2H), 5.55 - 5.37 (m, 1H, allyl), 5.31 (s, 2H, C*H*Ph$_2$), 2.27 (s, 3H, aryl-C*H$_3$*), 1.18 (s, 3H, N=C*Me*). ^{13}C NMR (100 MHz, CDCl$_3$) δ 200.14 (s, *C*OPh), 175.81 (s, N=*C*Me), 141.85 (s), 140.47 (s), 140.34 (s), 138.87 (s), 138.71 (s), 134.94 (s), 133.77 (s), 132.11 (s), 131.15 (s), 130.02 (s), 129.93 (s), 129.65 (s), 129.50 (s), 129.45 (s), 129.42 (s), 127.93 (s), 127.75 (s), 120.25 (s, allyl), 53.26 (s, *C*HPh$_2$), 21.50 (s, aryl-*C*H$_3$), 20.31 (s, N=C*Me*). Key ^{1}H-^{13}C HSQC correlations (CDCl$_3$): 120.25 (s, allyl)/ 5.55 - 5.37 (m, 1H, allyl), 53.26 (s,

CHPh$_2$)/ 5.31 (s, 2H, CHPh$_2$), 21.50 (s, aryl-CH$_3$)/ 2.27 (s, 3H, aryl-CH_3), 20.31 (s, N=CMe)/ 1.18 (s, 3H, N=CMe). Anal. Calcd for $C_{77}H_{52}BF_{24}NNiO$: C, 60.34; H, 3.42; Found: C, 60.21; H, 3.55.

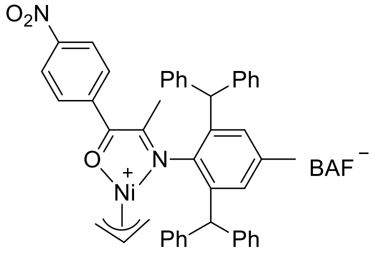

Preparation of Ni7. Similar procedure as catalyst **Ni1** was employed except **L7** (307 mg, 0.50 mmol) was used. **Ni7** was obtained as a red powder (670 mg, 85%). ^{1}H NMR (400 MHz, CDCl$_3$) δ 8.27 (d, J = 9.0 Hz, 2H), 7.80 (d, J = 9.0 Hz, 2H), 7.71 (s, 8H), 7.49 (s, 4H), 7.42 - 7.31 (m, 8H), 7.25 - 7.22 (m, 4H), 7.04 (d, J = 7.2 Hz, 4H), 6.98 (dd, J = 6.4, 2.7 Hz, 4H), 6.92 (s, 2H), 5.53 - 5.44 (m, 1H, allyl), 5.26 (s, 2H, CHPh$_2$), 2.27 (s, 3H, aryl-CH_3), 1.12 (s, 3H, N=CMe). ^{13}C NMR (100 MHz, CDCl$_3$) δ 199.80 (s, COPh), 174.26 (s), 152.20 (s), 141.66 (s), 140.42 (s), 140.19 (s), 139.25 (s), 136.64 (s), 133.75 (s), 131.71 (s), 130.08 (s), 129.54 (s), 129.41 (s), 128.09 (s), 127.87 (s), 124.77 (s), 121.46 (s, allyl), 53.37 (s, CHPh$_2$), 21.53 (s, aryl-CH$_3$), 19.95 (s, N=CMe). Key ^{1}H-^{13}C HSQC correlations (CDCl$_3$): 121.46 (s, allyl)/ 5.53 - 5.44 (m, 1H, allyl), 53.37 (s, CHPh$_2$)/ 5.26 (s, 2H, CHPh$_2$), 21.53 (s, aryl-CH$_3$)/ 2.27 (s, 3H, aryl-CH_3), 19.95 (s, N=CMe)/ 1.12 (s, 3H, N=CMe). Anal. Calcd for $C_{77}H_{51}BF_{24}N_2NiO_3$: C, 58.62; H, 3.26; Found: C, 58.51; H, 3.35.

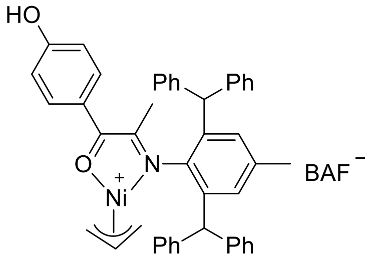

Preparation of Ni-OH. Similar procedure as catalyst **Ni1** was employed except **L-OH** (293 mg, 0.50 mmol) was used. **Ni-OH** was obtained as a red powder (619 mg, 80%). ^{1}H NMR (400 MHz, CDCl$_3$) δ 7.78 - 7.50 (m, 10H), 7.37 (s, 4H), 7.24 (t, J = 7.3 Hz, 4H), 7.18 (d, J = 6.7 Hz, 2H), 7.08 (s, 6H), 6.94 (s, 4H), 6.87 (s, 3H), 6.76 (s, 2H), 6.67 (d, J = 8.6 Hz, 2H), 5.68 (s, 1H), 5.40 - 4.95 (m, 3H), 2.12 (s, 3H, aryl-CH_3), 1.02 (s, 3H, N=CMe). ^{13}C NMR (100 MHz, CDCl$_3$) δ 195.40 (s), 176.51 (s), 165.68 (s), 142.02 (s), 140.56 (s), 140.41 (s), 138.44 (s), 135.55 (s), 133.76 (s), 129.87 (s), 129.50 (s), 129.44 (s), 129.34 (s), 127.84 (s), 127.64 (s), 125.02 (s), 119.46 (s, allyl), 117.45 (s), 53.17 (s), 21.50 (s, aryl-CH$_3$), 20.50 (s, N=CMe). Key ^{1}H-^{13}C HSQC correlations (CDCl$_3$): 119.46 (s, allyl)/ 5.40 - 4.95 (m, 3H), 21.50 (s, aryl-CH$_3$)/ 2.12 (s, 3H, aryl-CH_3), 20.50 (s, N=CMe)/ 1.02 (s, 3H, N=CMe). Anal. Calcd for $C_{77}H_{52}BF_{24}NNiO_2$: C, 59.72; H, 3.38; Found: C, 59.86; H, 3.25.

Preparation of the supported catalyst Ni-OH@SiO$_2$. The silica gel was calcined at 600 °C for 6 h, then the SiO$_2$ (100 mg) was added to a toluene solution (1 mL) of catalyst **Ni-OH** (1 μmol) under a nitrogen atmosphere and the mixture was stirred at room temperature for 6 h. The supported catalyst was isolated by filtration. Then the supported catalyst washed two times with toluene and dried under vacuum. The nickel content (0.057% wt) in the prepared catalysts was determined by AES-ICP.

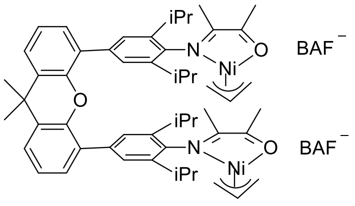

Preparation of Ni-Ni. A mixture of **L-L** (174 mg, 0.25 mmol), [Ni(allyl)Cl]$_2$ (67.5 mg, 0.5 mmol), and NaBAF (443 mg, 0.5 mmol) in 15 mL CH$_2$Cl$_2$ was stirred at room temperature for 12 h. The resulting mixture was filtrated over celite and evaporated to get a black red solid (557 mg, 85%). ^{1}H NMR (400 MHz, CDCl$_3$) δ 7.70 (s, 16H), 7.62 - 7.53 (m, 4H), 7.51 (s, 8H), 7.26 - 7.10 (m, 6H), 5.72 - 5.57 (m, 2H), 2.51 (s, 4H), 2.28 (s, 6H), 1.92 (s, 6H), 1.78 (s, 6H), 1.14 (d, J = 5.9 Hz, 12H), 0.75 (d, J = 5.6 Hz, 12H). ^{13}C NMR (100 MHz, CDCl$_3$) δ 210.66 (s), 170.83 (s), 147.02 (s), 140.88 (s), 140.26 (s), 136.84 (s), 131.42 (s), 131.31 (s), 127.27 (s), 127.08 (s), 125.42 (s), 124.02 (s), 120.45 (s), 113.97 (s), 34.51 (s), 33.50 (s), 29.14 (s), 26.41 (s), 23.44 (s), 23.23 (s), 17.92 (s). Anal. Calcd for $C_{117}H_{90}B_2F_{48}N_2Ni_2O_3$: C, 53.58; H, 3.46; Found: C, 53.71; H, 3.29.

Supplementary Figures

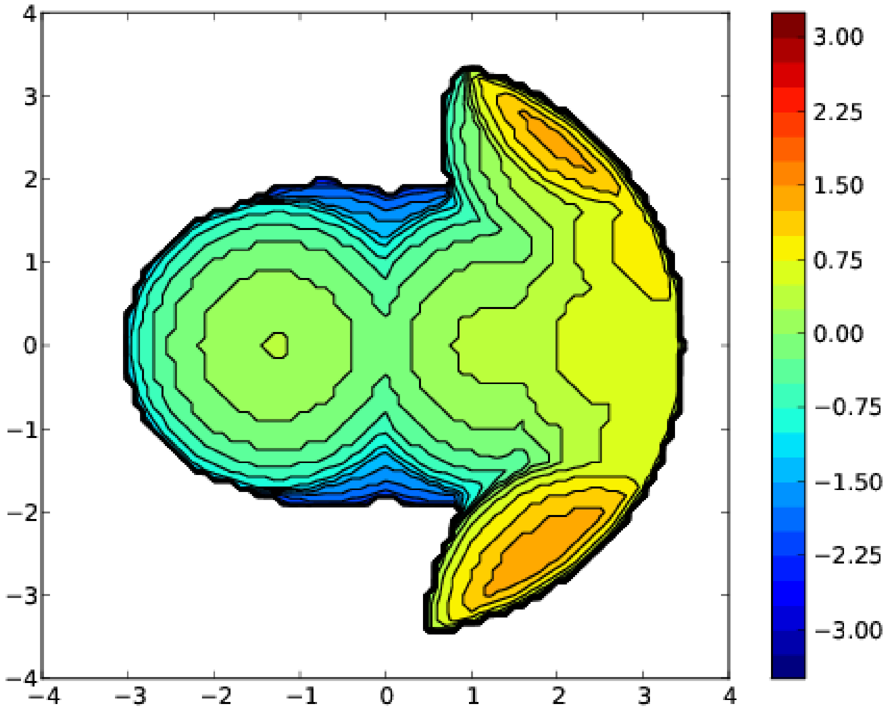

%V_Free	%V_Bur	% Tot/Ex
60.1	**39.9**	100.0

Supplementary Figure 1. Topographical steric map of **Ni1**.

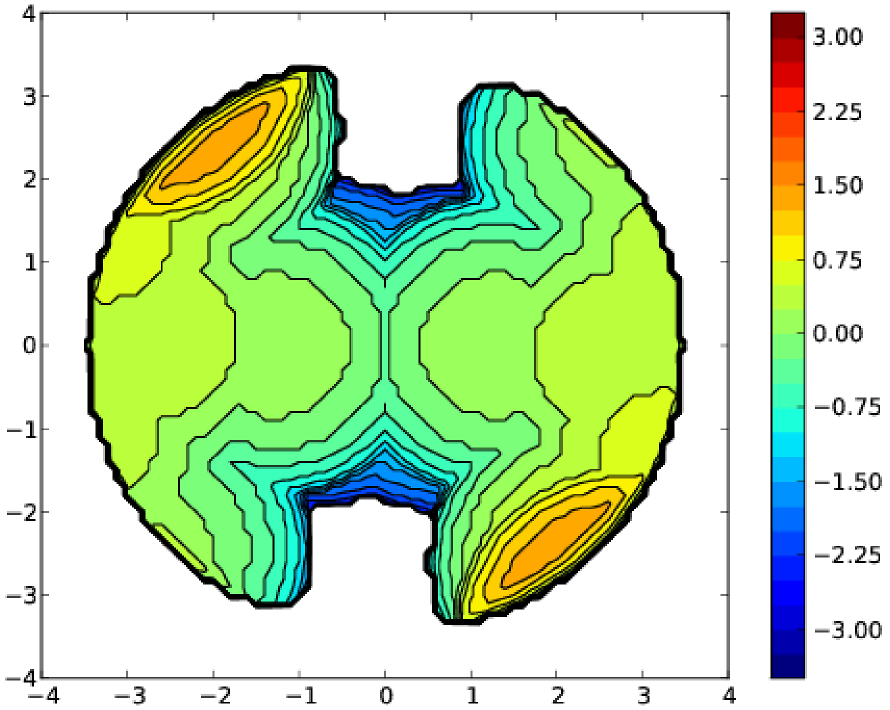

%V_Free	%V_Bur	% Tot/Ex
53.4	**46.6**	100.0

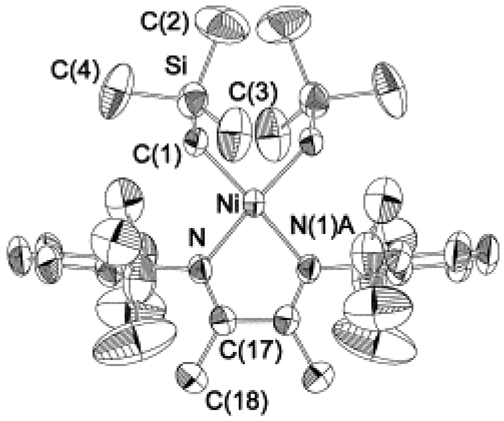

Supplementary Figure 2. Topographical steric map of an analogous α-diimine based nickel complex.

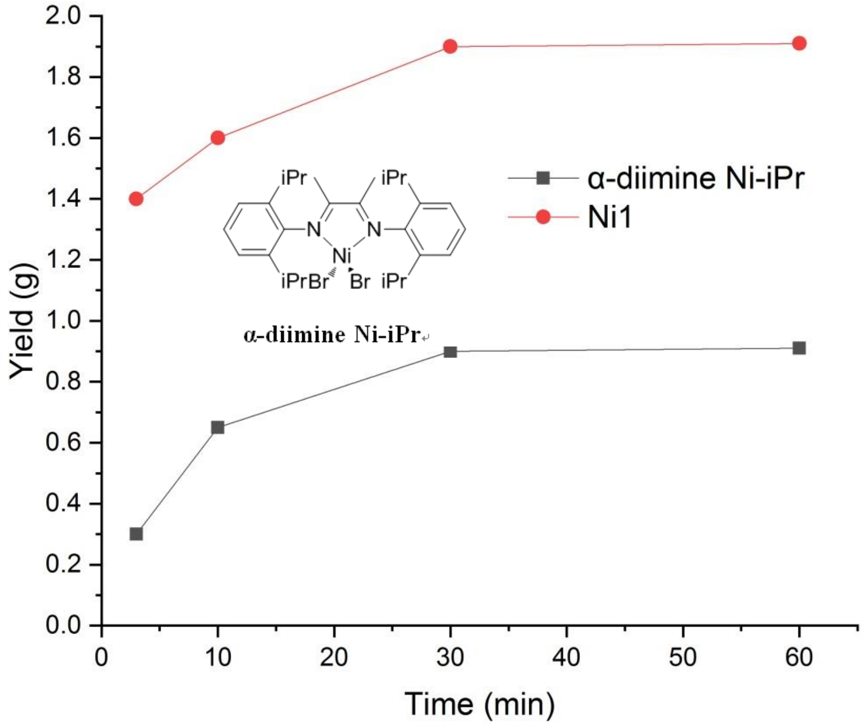

Supplementary Figure 3. Polymer yield as a function of time for **Ni1** and **α-diimine Ni-iPr** in ethylene polymerization at 80 °C. Polymerization conditions: **Ni1** = 1 μmol, toluene = 28 mL, CH_2Cl_2 = 2 mL; **α-diimine Ni-iPr** = 1 μmol, Al/Ni=500, CH_2Cl_2 = 2 mL, toluene = 28 mL, ethylene = 8 atm.

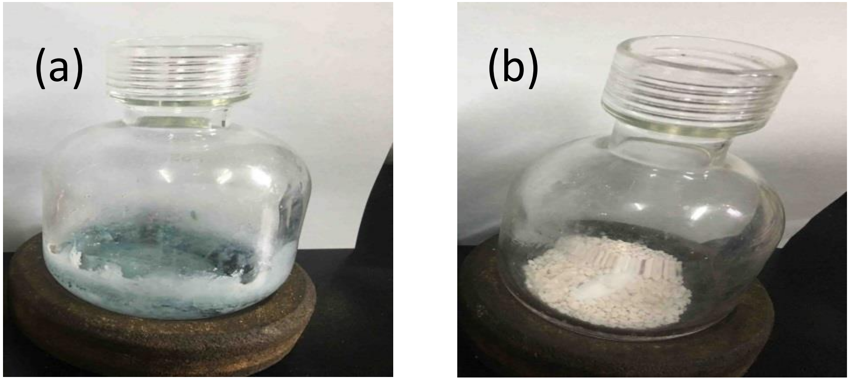

Supplementary Figure 4. Photograph of the polyethylene: (a) polyethylene by homo-catalyst at 100 °C; (b) polyethylene by hetero-catalyst at 100 °C.

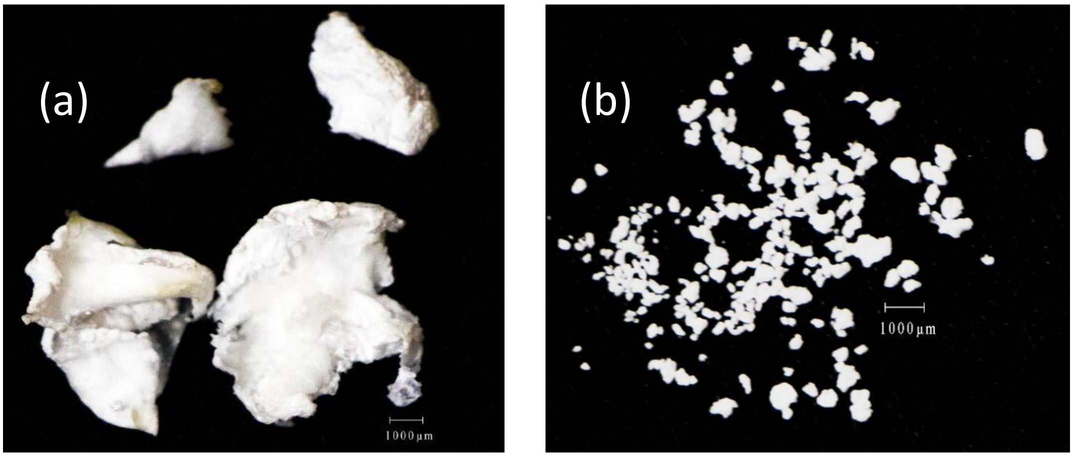

Supplementary Figure 5. Photograph of the polyethylene: (a) polyethylene by homo-catalyst; (b) polyethylene by hetero-catalyst.

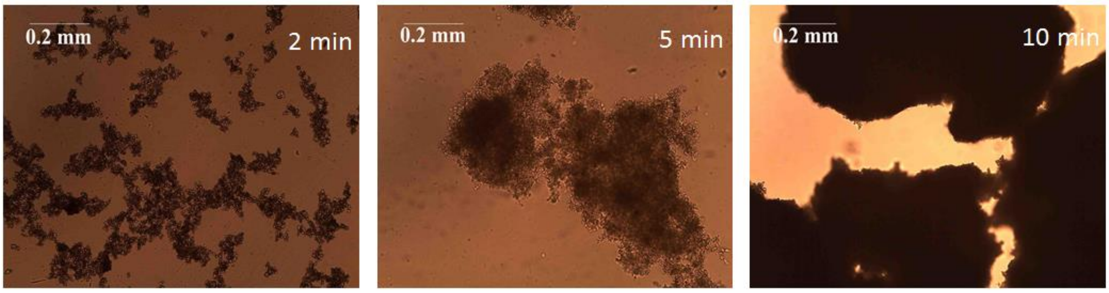

Supplementary Figure 6. Microphotographs of polymers obtained by **Ni-OH@SiO$_2$** at 50 °C.

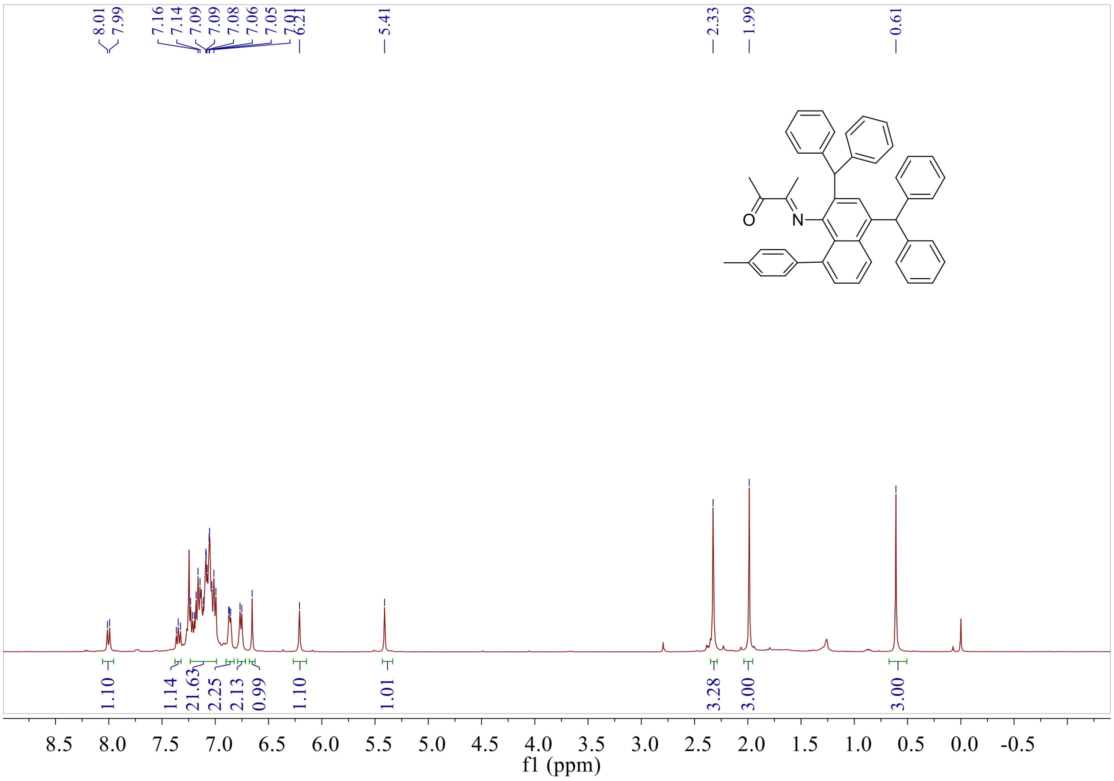

Supplementary Figure 7. ^{1}H NMR spectrum (400 MHz, $CDCl_3$) of **L3.**

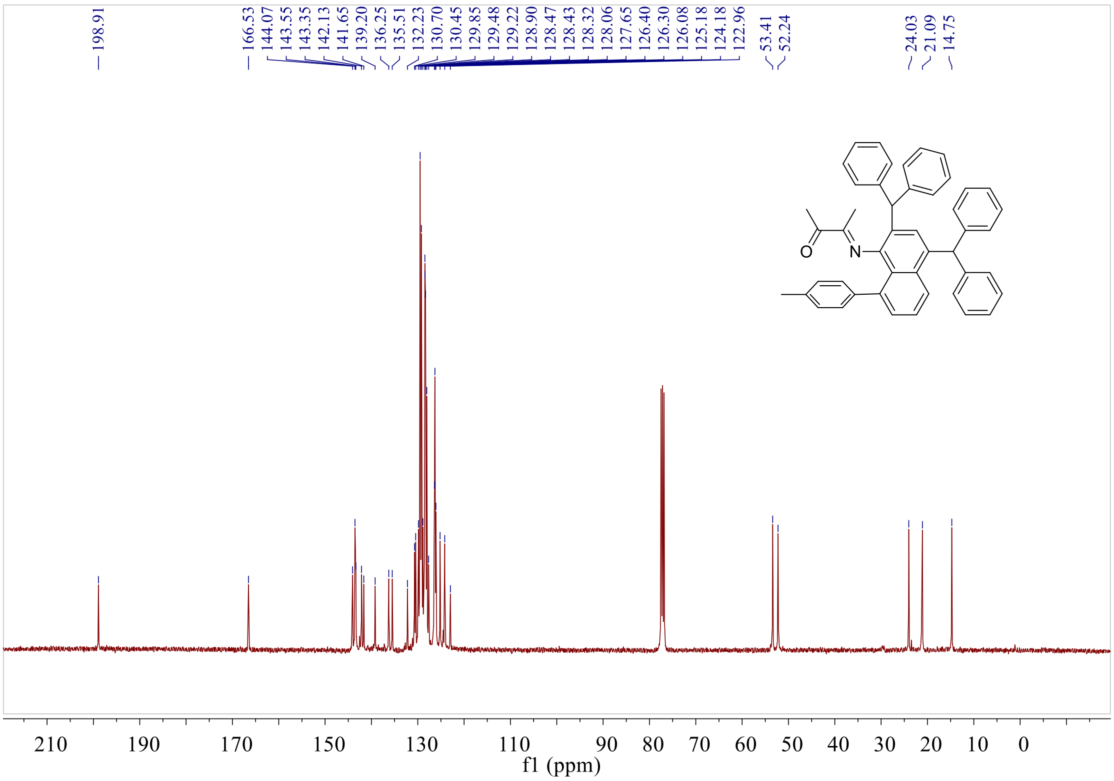

Supplementary Figure 8. ^{13}C NMR spectrum in $CDCl_3$ of **L3.**

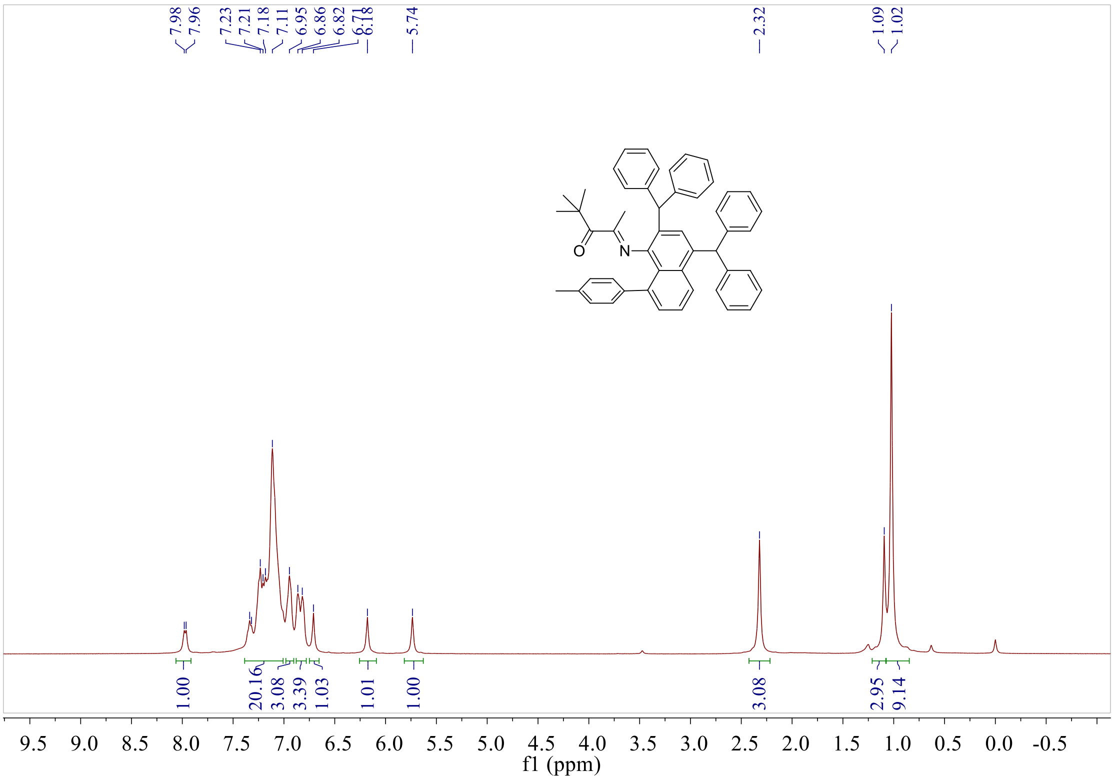

Supplementary Figure 9. ^{1}H NMR spectrum (400 MHz, $CDCl_3$) of **L4**.

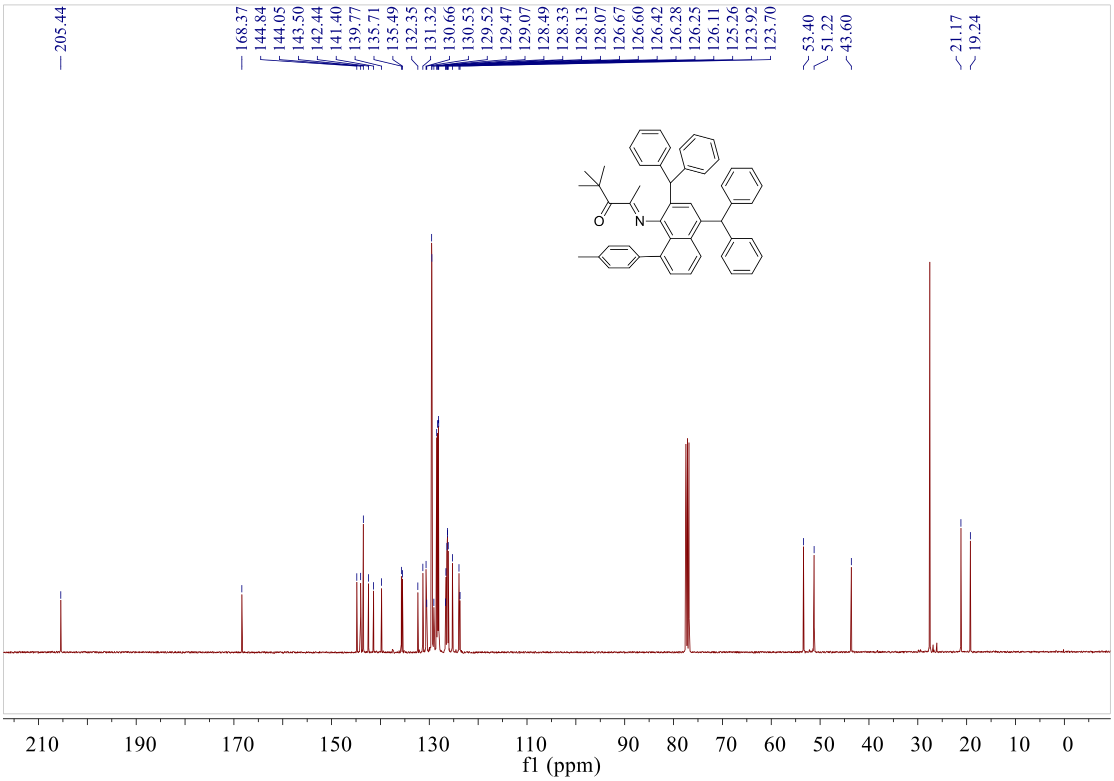

Supplementary Figure 10. ^{13}C NMR spectrum in $CDCl_3$ of **L4.**

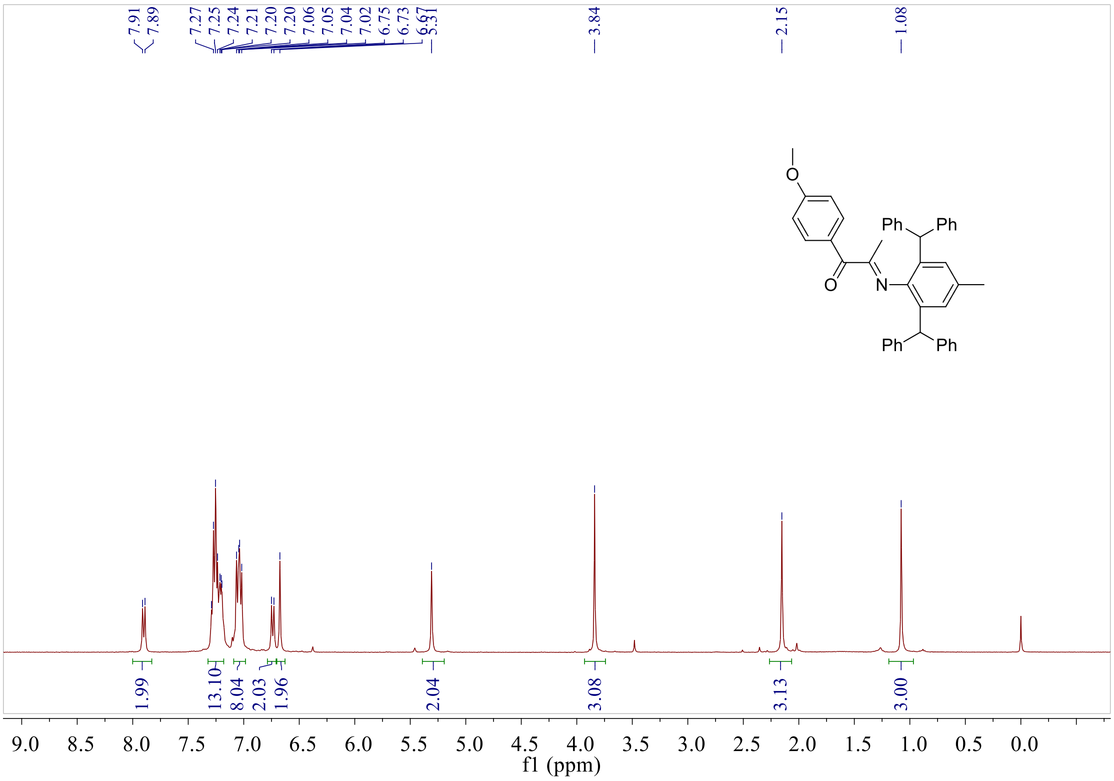

Supplementary Figure 11. ^{1}H NMR spectrum (400 MHz, $CDCl_3$) of **L5.**

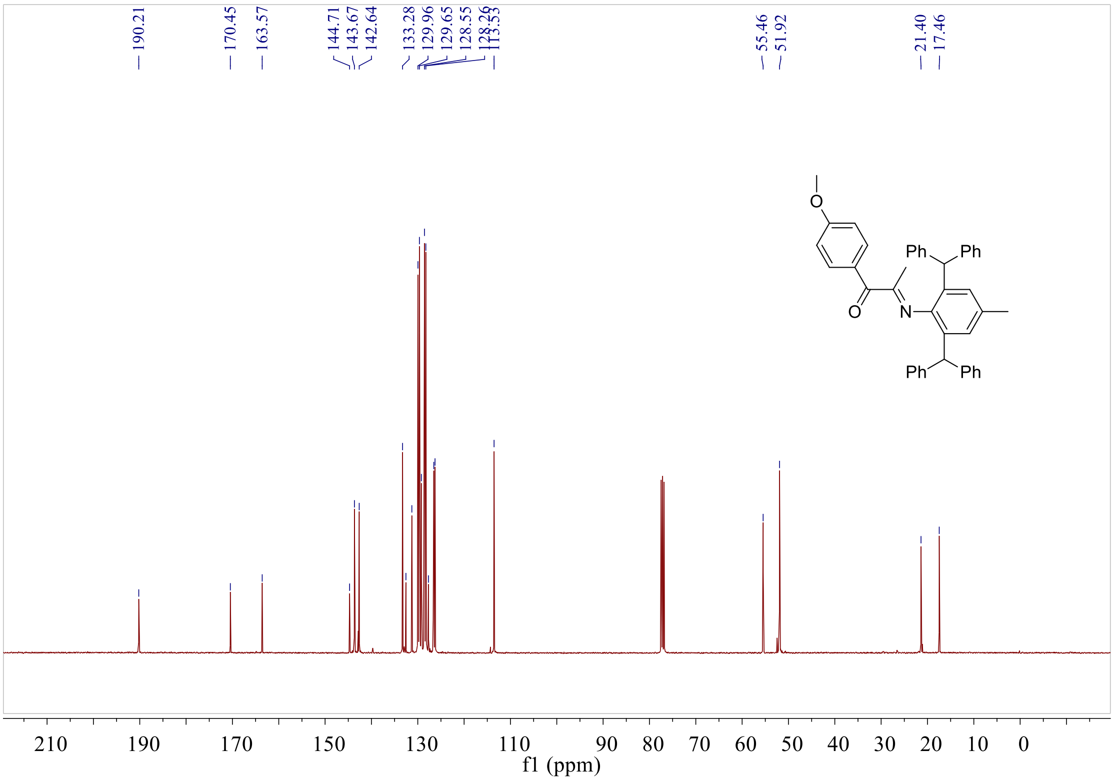

Supplementary Figure 12. ^{13}C NMR spectrum in $CDCl_3$ of **L5.**

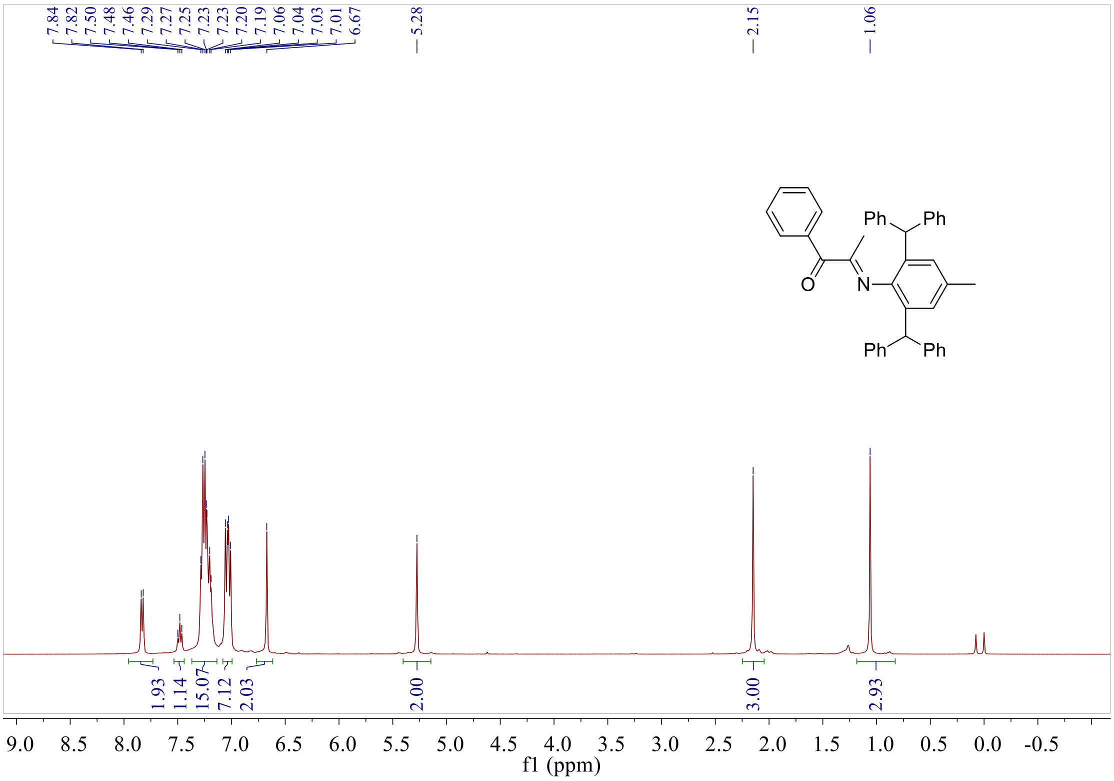

Supplementary Figure 13. ^{1}H NMR spectrum (400 MHz, $CDCl_3$) of **L6.**

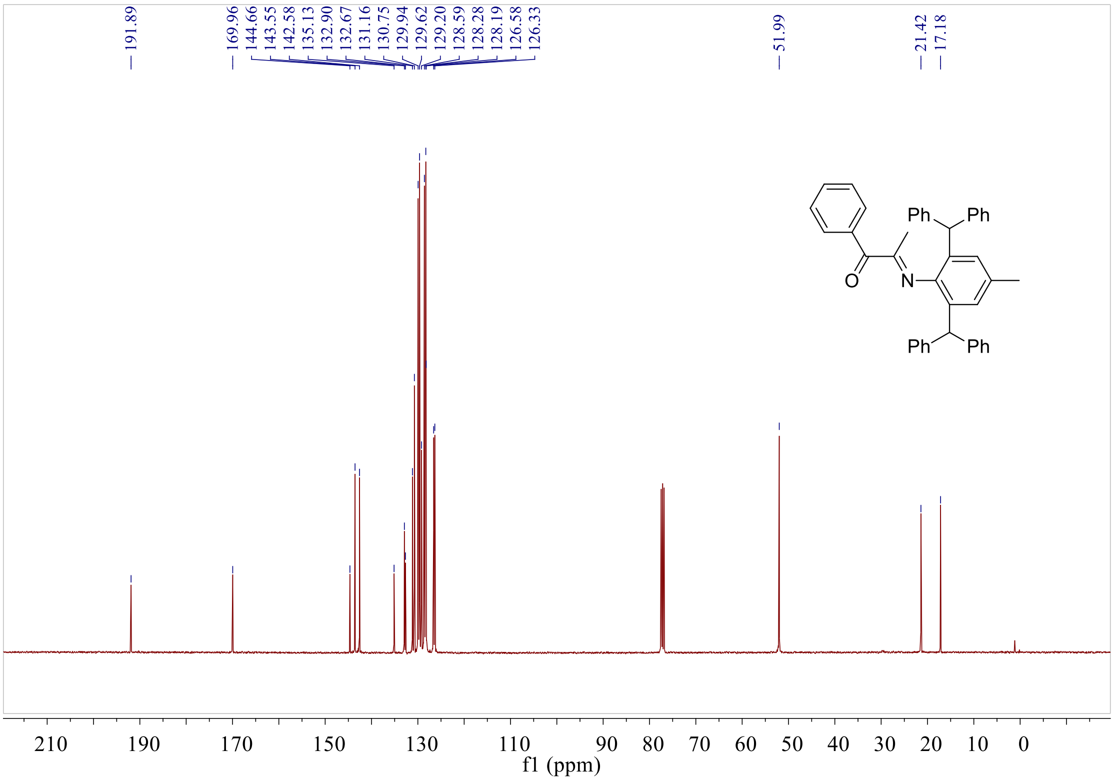

Supplementary Figure 14. ^{13}C NMR spectrum in $CDCl_3$ of **L6.**

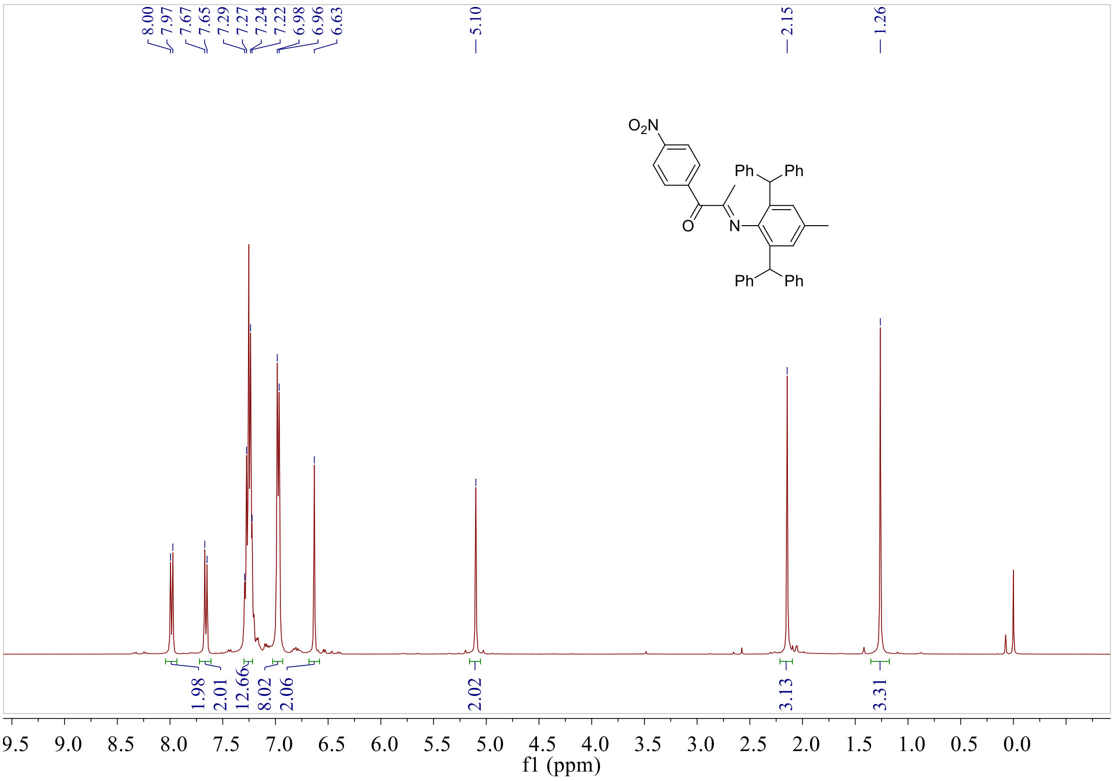

Supplementary Figure 15. ^{1}H NMR spectrum (400 MHz, $CDCl_3$) of **L7.**

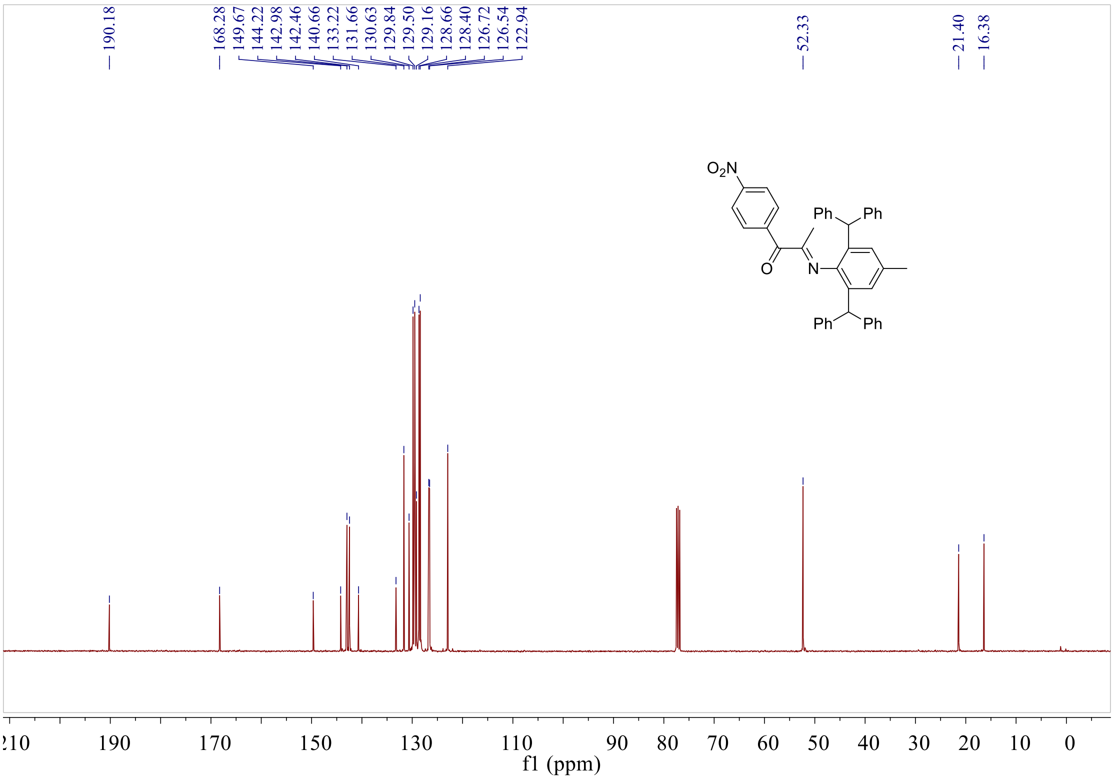

Supplementary Figure 16. ^{13}C NMR spectrum in $CDCl_3$ of **L7.**

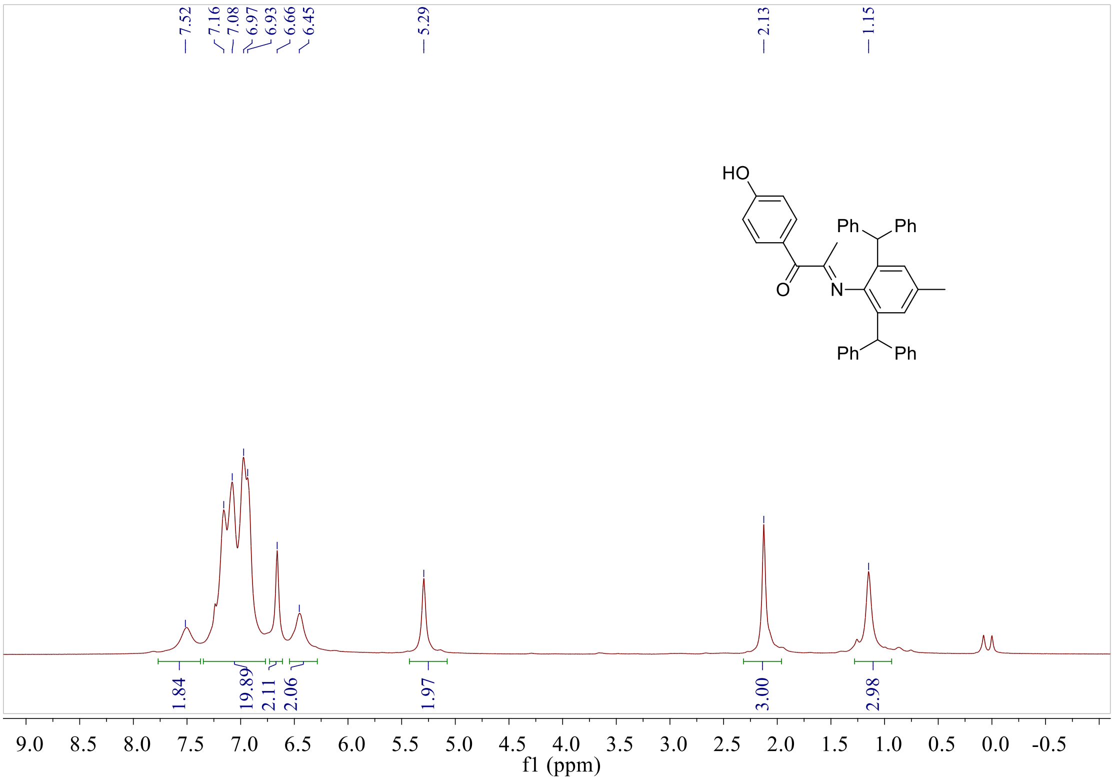

Supplementary Figure 17. ^{1}H NMR spectrum (400 MHz, $CDCl_3$) of **L-OH.**

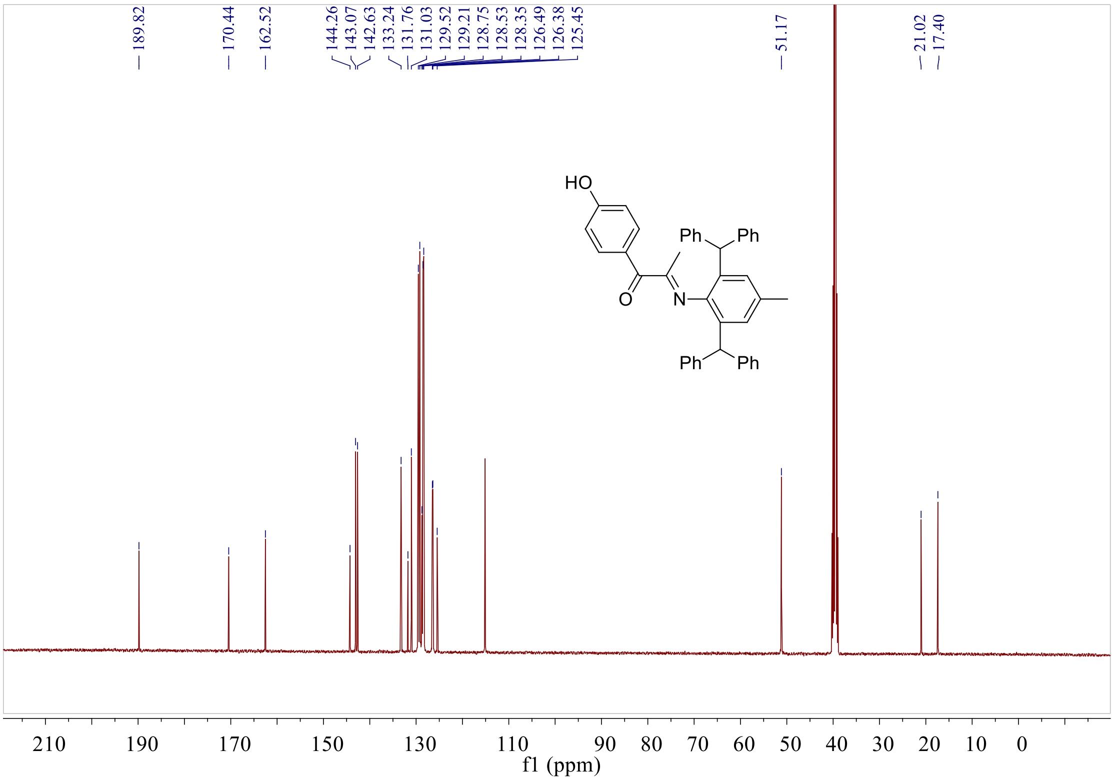

Supplementary Figure 18. ^{13}C NMR spectrum (100 MHz, [D_6]DMSO) of **L-OH**.

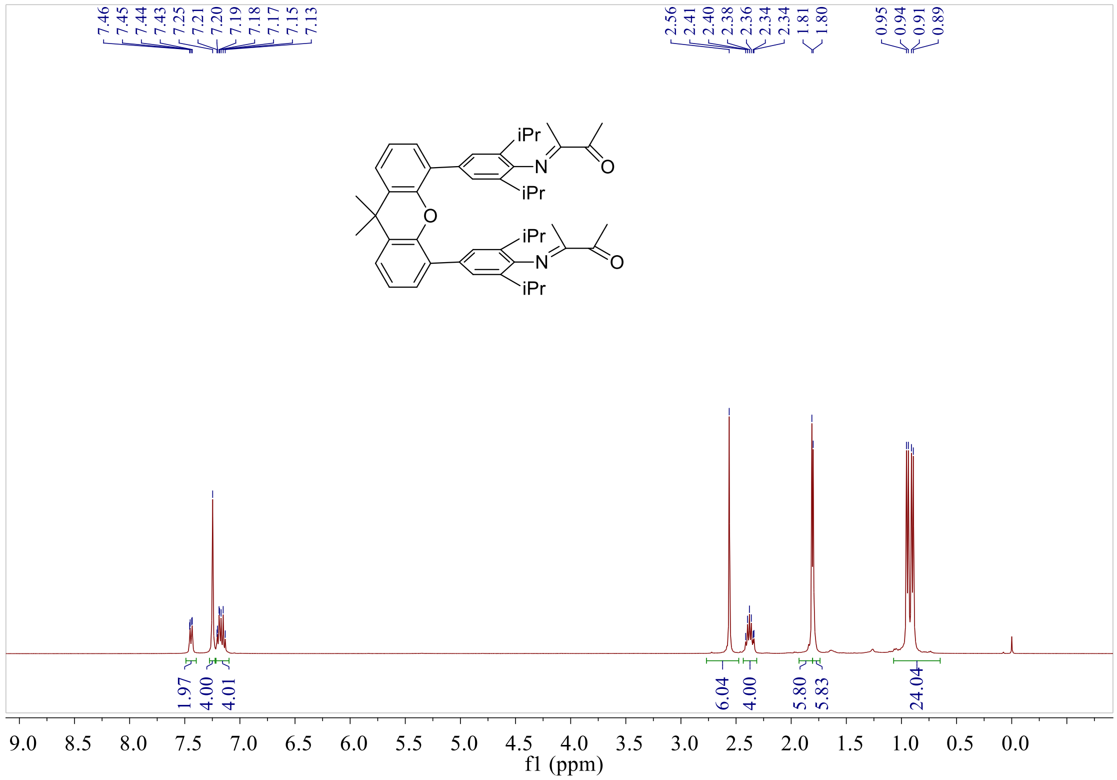

Supplementary Figure 19. ^{1}H NMR spectrum (400 MHz, $CDCl_3$) of **L-L.**

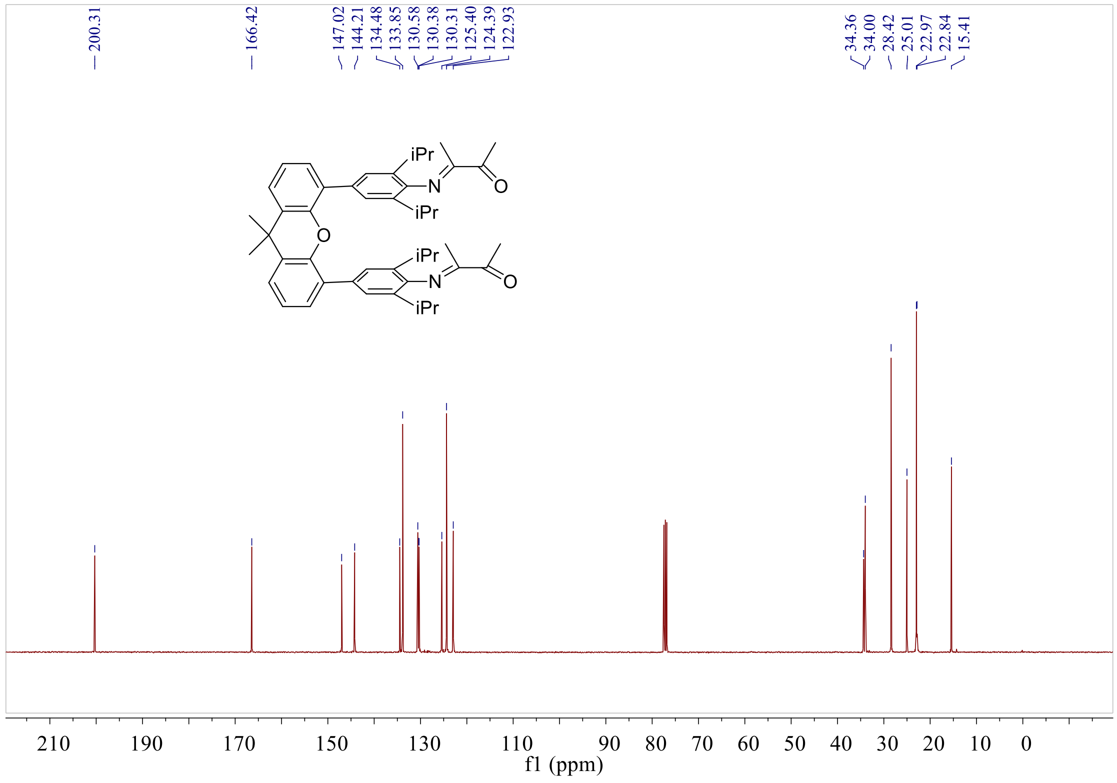

Supplementary Figure 20. ^{13}C NMR spectrum in $CDCl_3$ of **L-L.**

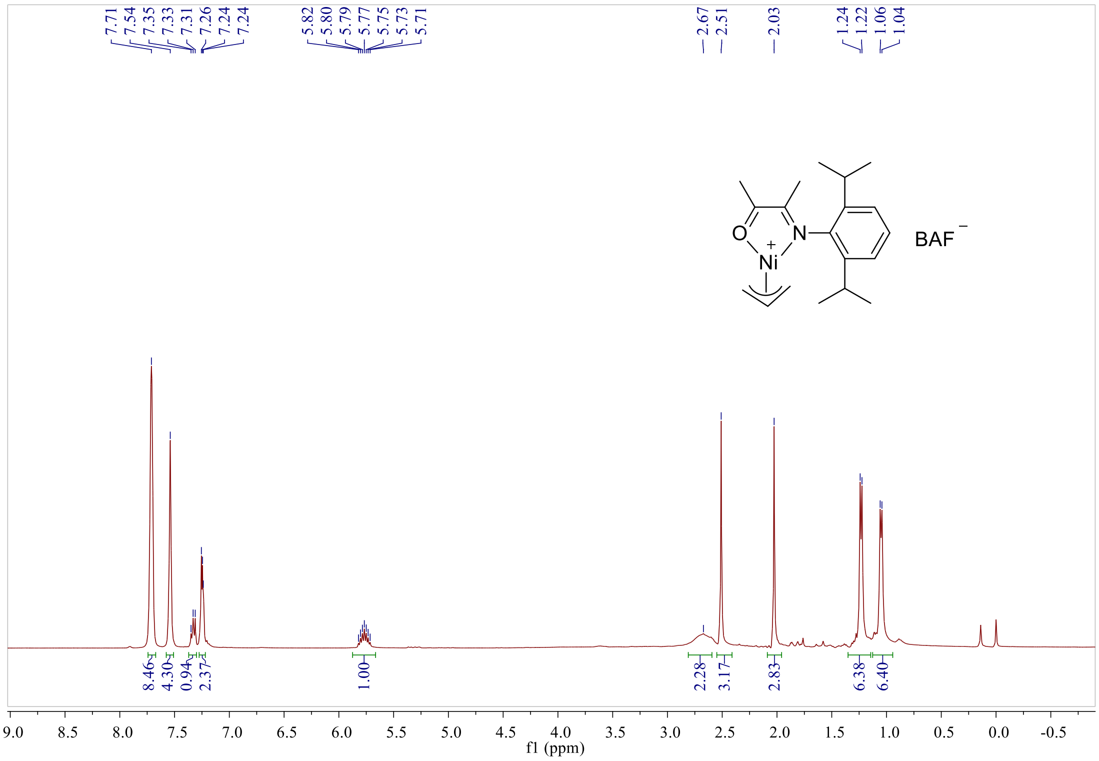

Supplementary Figure 21. ^{1}H NMR spectrum (400 MHz, $CDCl_3$) of **Ni1.**

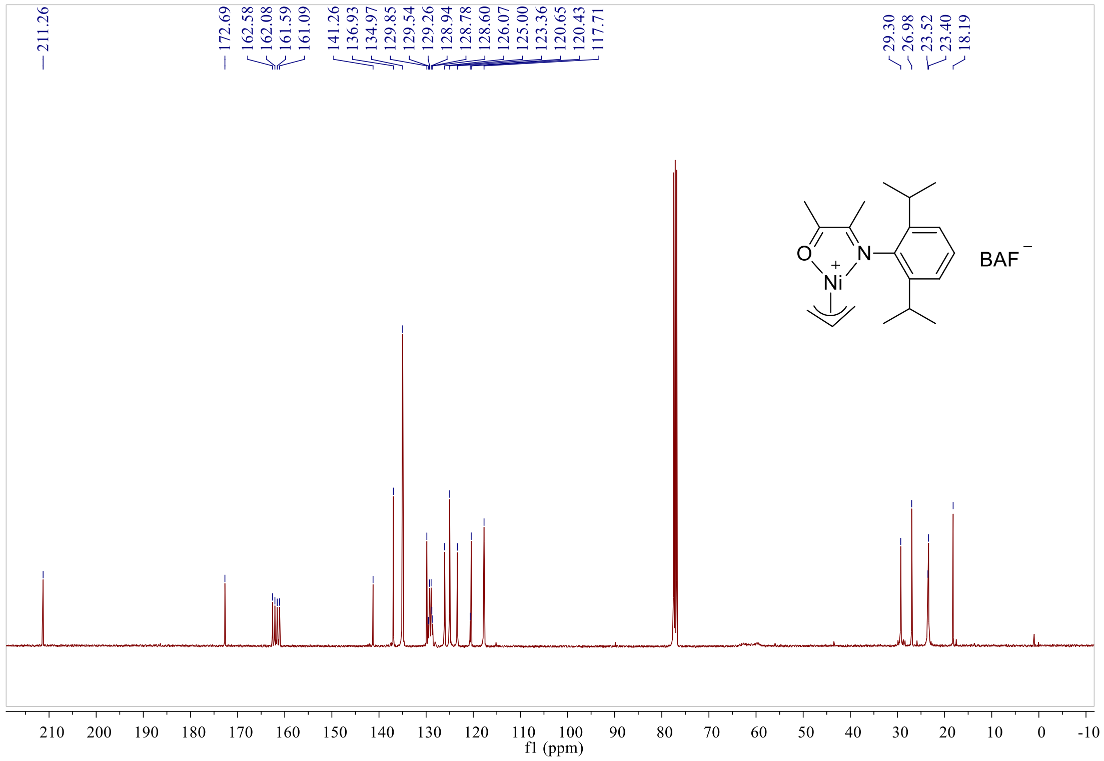

Supplementary Figure 22. ^{13}C NMR spectrum in $CDCl_3$ of **Ni1.**

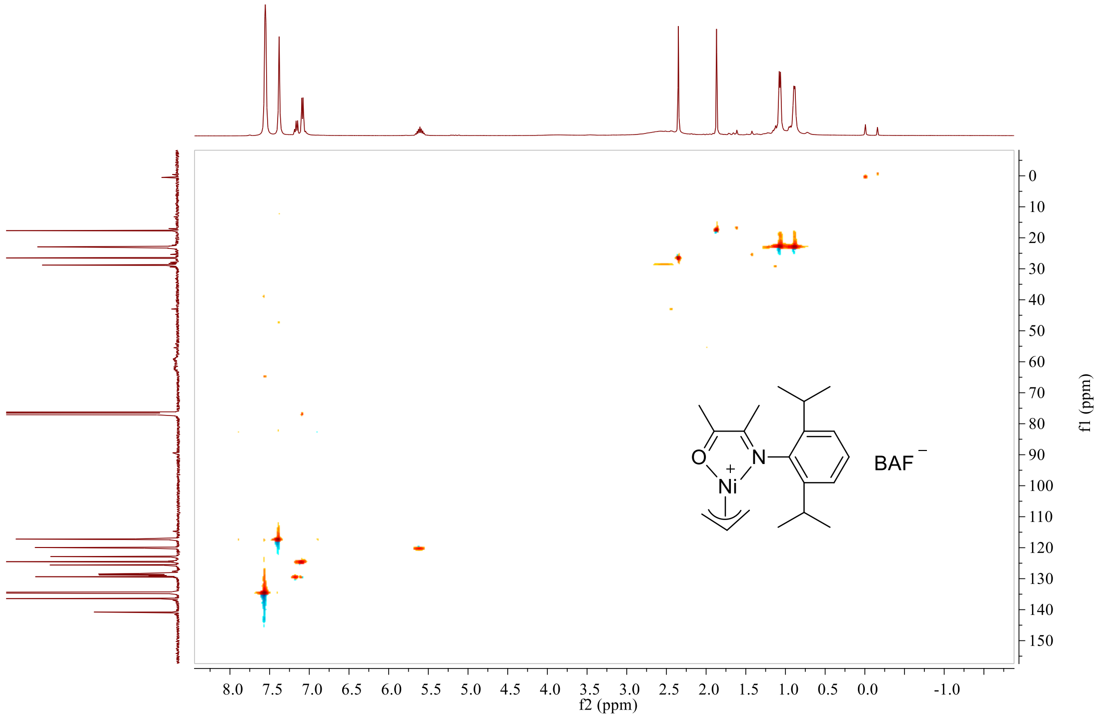

Supplementary Figure 23. ^{1}H-^{13}C HSQC NMR spectrum of **Ni1** in $CDCl_3$.

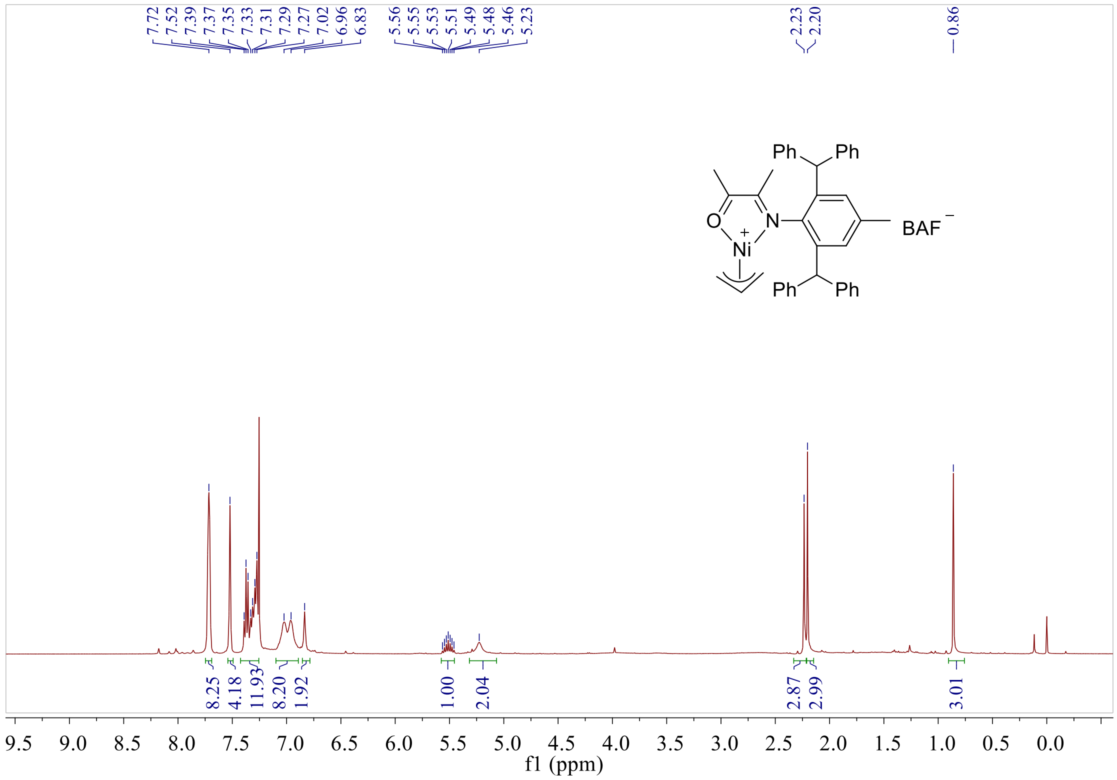

Supplementary Figure 24. ^{1}H NMR spectrum (400 MHz, $CDCl_3$) of **Ni2.**

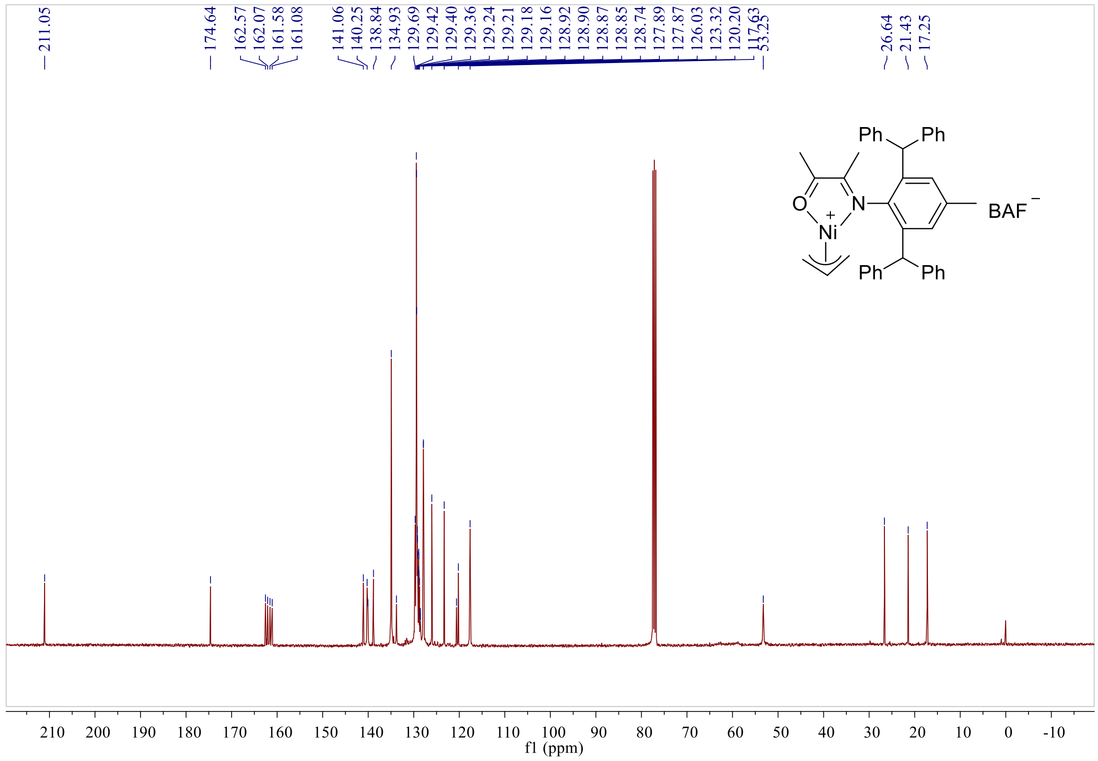

Supplementary Figure 25. ^{13}C NMR spectrum in $CDCl_3$ of **Ni2.**

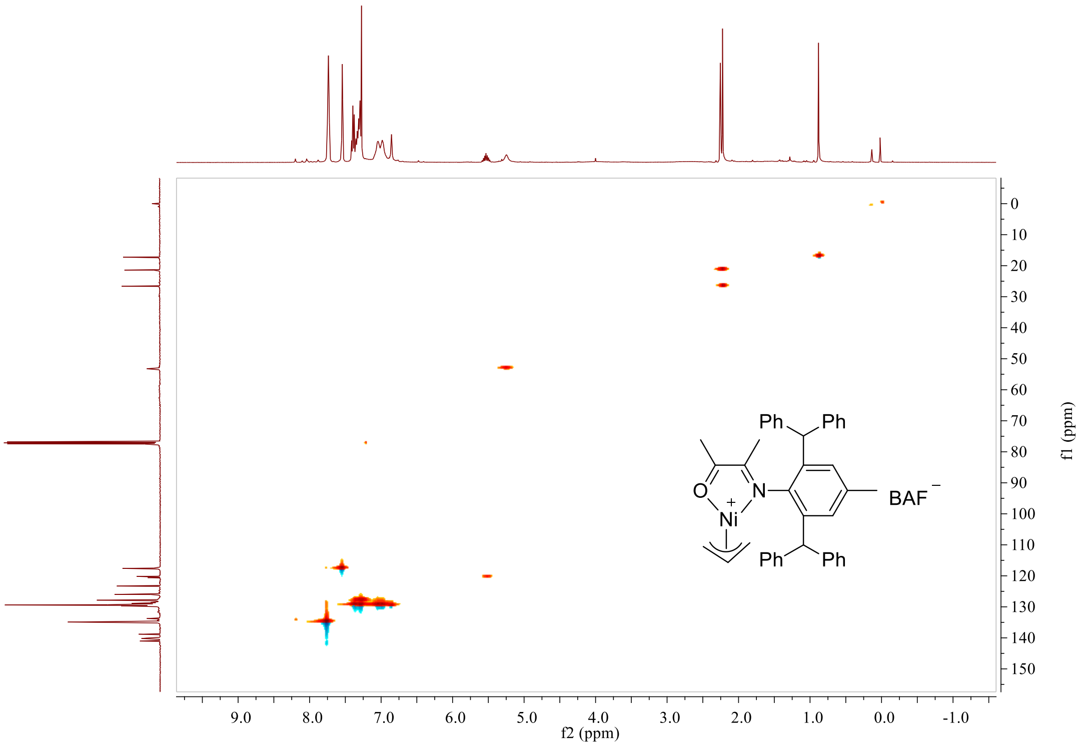

Supplementary Figure 26. ^{1}H-^{13}C HSQC NMR spectrum of **Ni2** in $CDCl_3$.

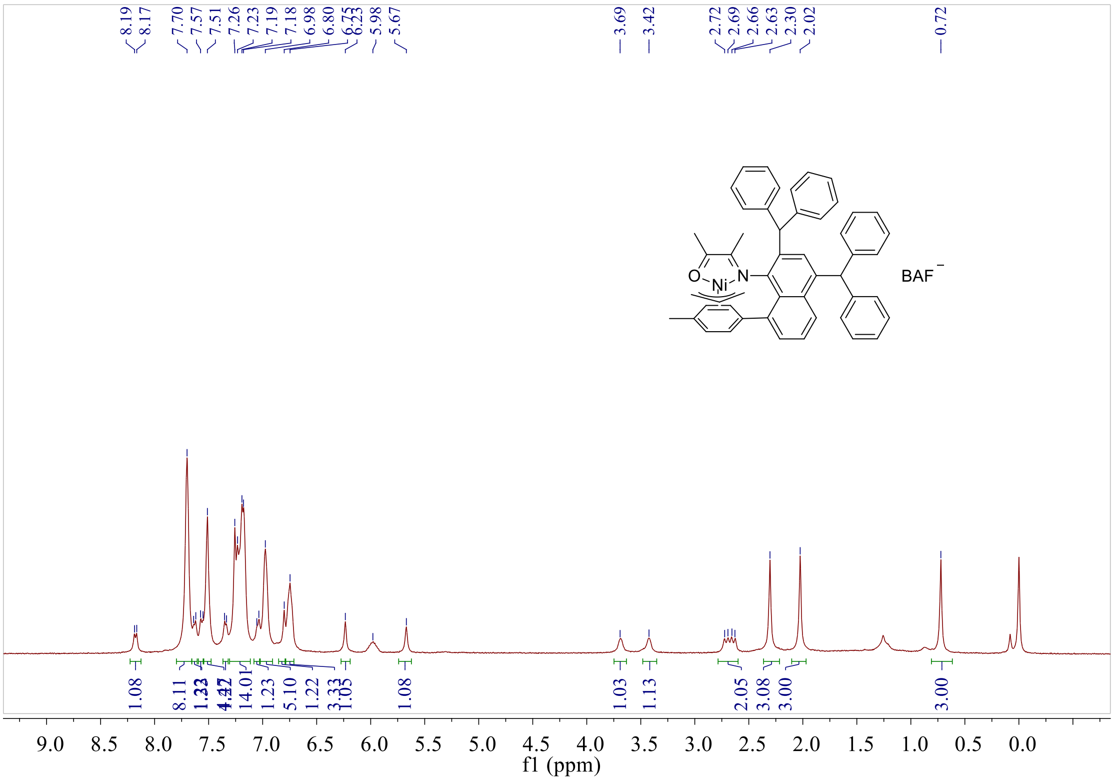

Supplementary Figure 27. ^{1}H NMR spectrum (400 MHz, $CDCl_3$) of **Ni3.**

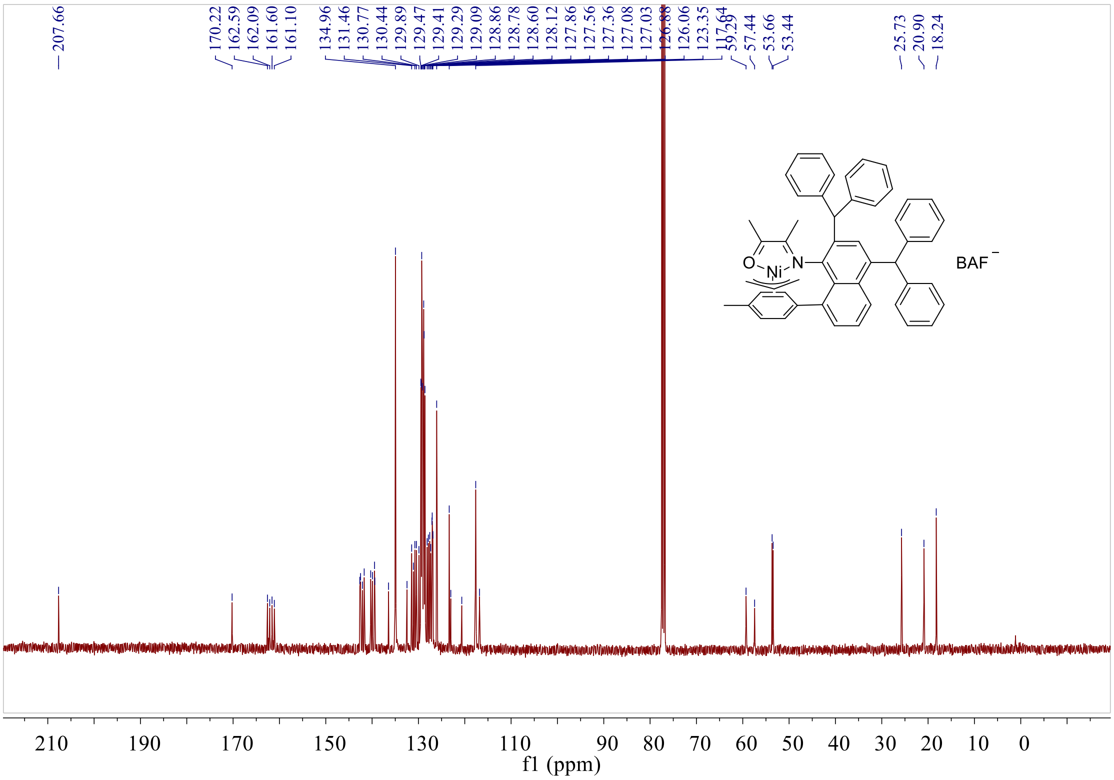

Supplementary Figure 28. ^{13}C NMR spectrum in $CDCl_3$ of **Ni3.**

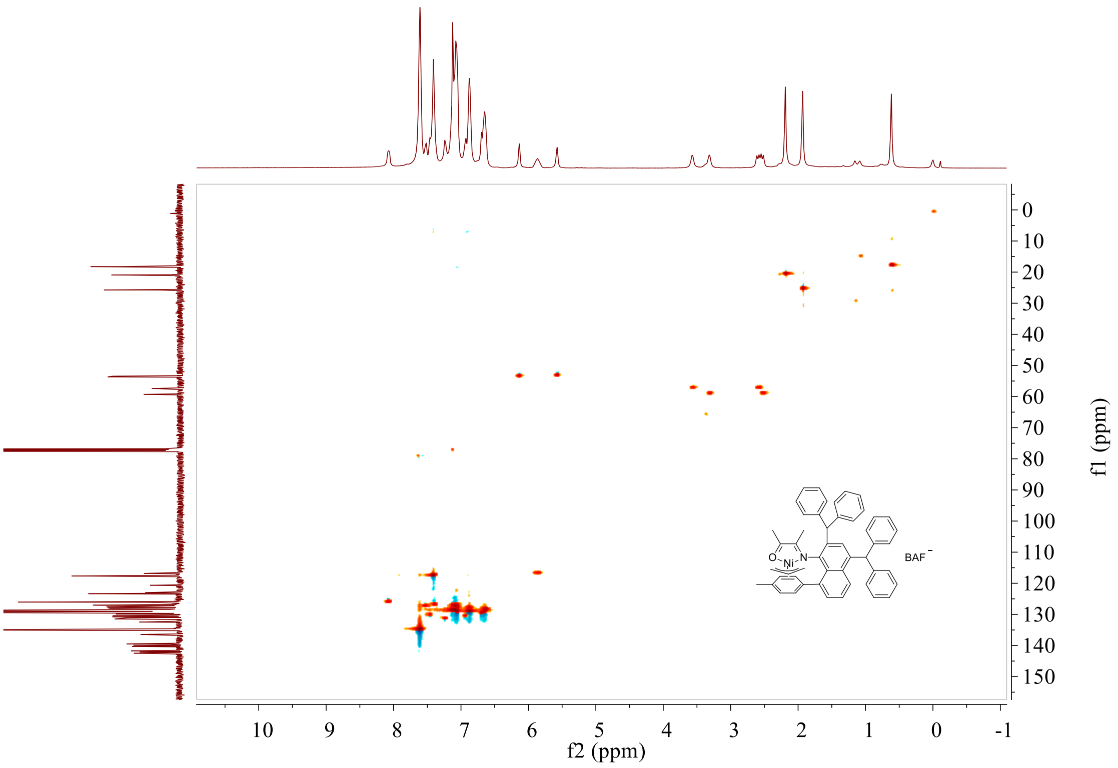

Supplementary Figure 29. ^{1}H-^{13}C HSQC NMR spectrum of **Ni3** in $CDCl_3$.

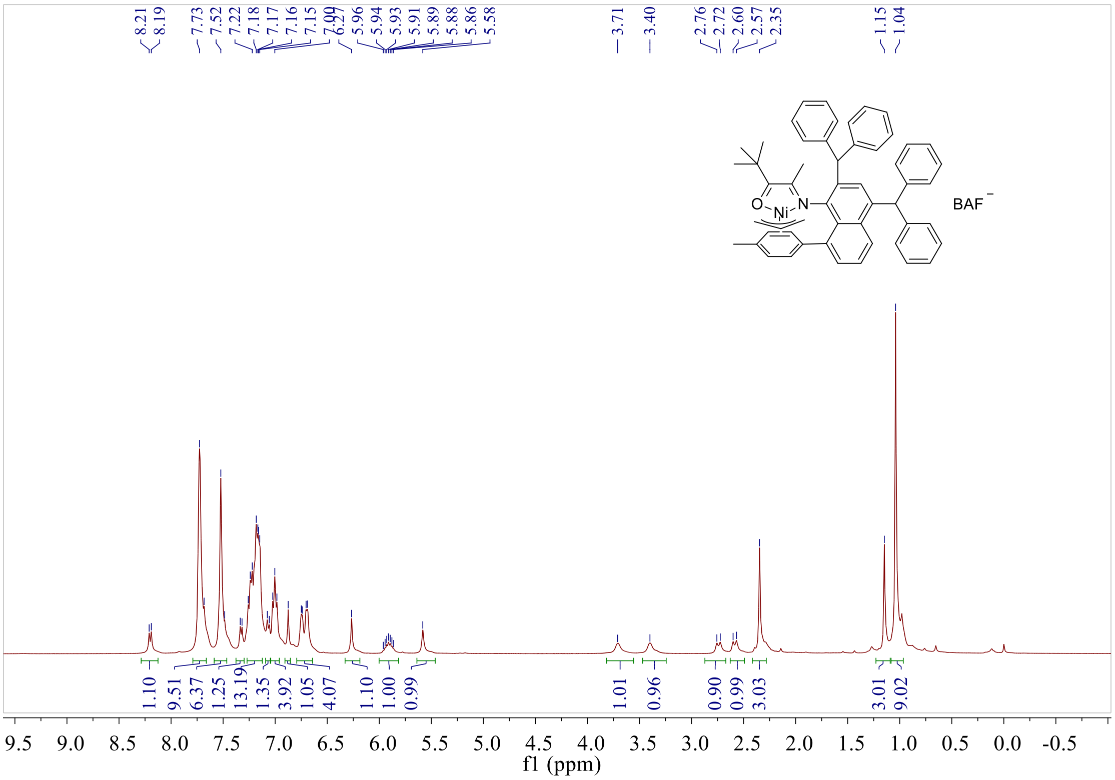

Supplementary Figure 30. ^{1}H NMR spectrum (400 MHz, $CDCl_3$) of **Ni4.**

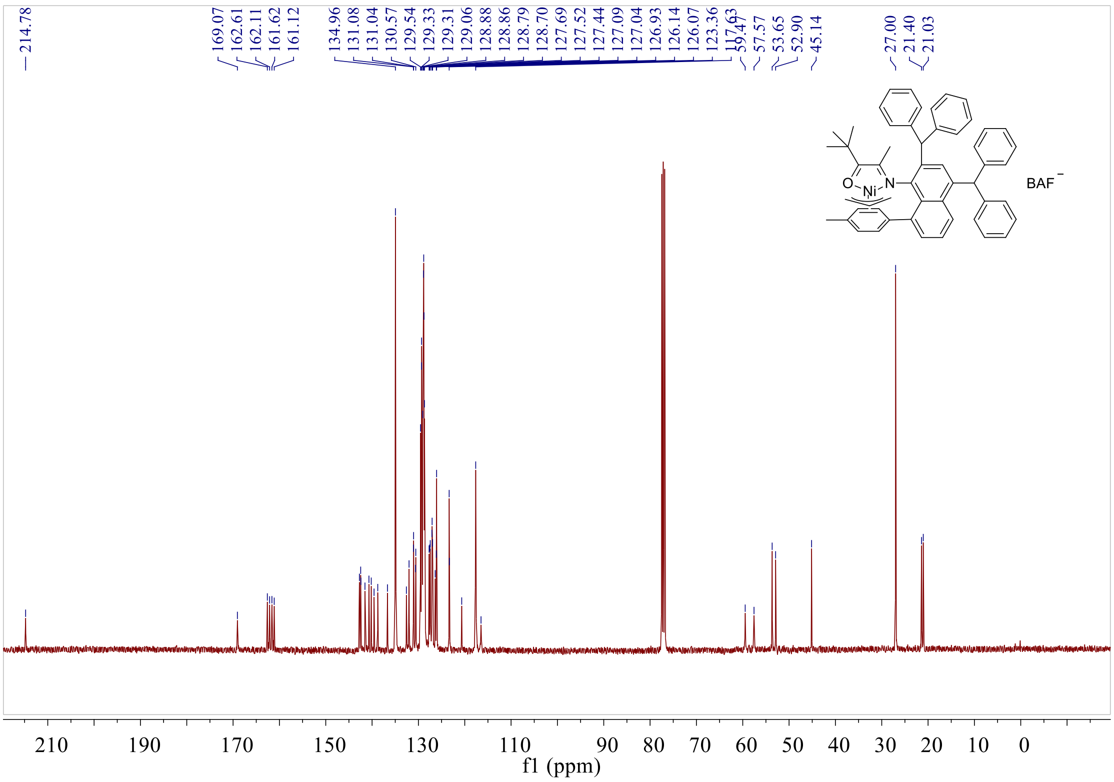

Supplementary Figure 31. ^{13}C NMR spectrum in $CDCl_3$ of **Ni4.**

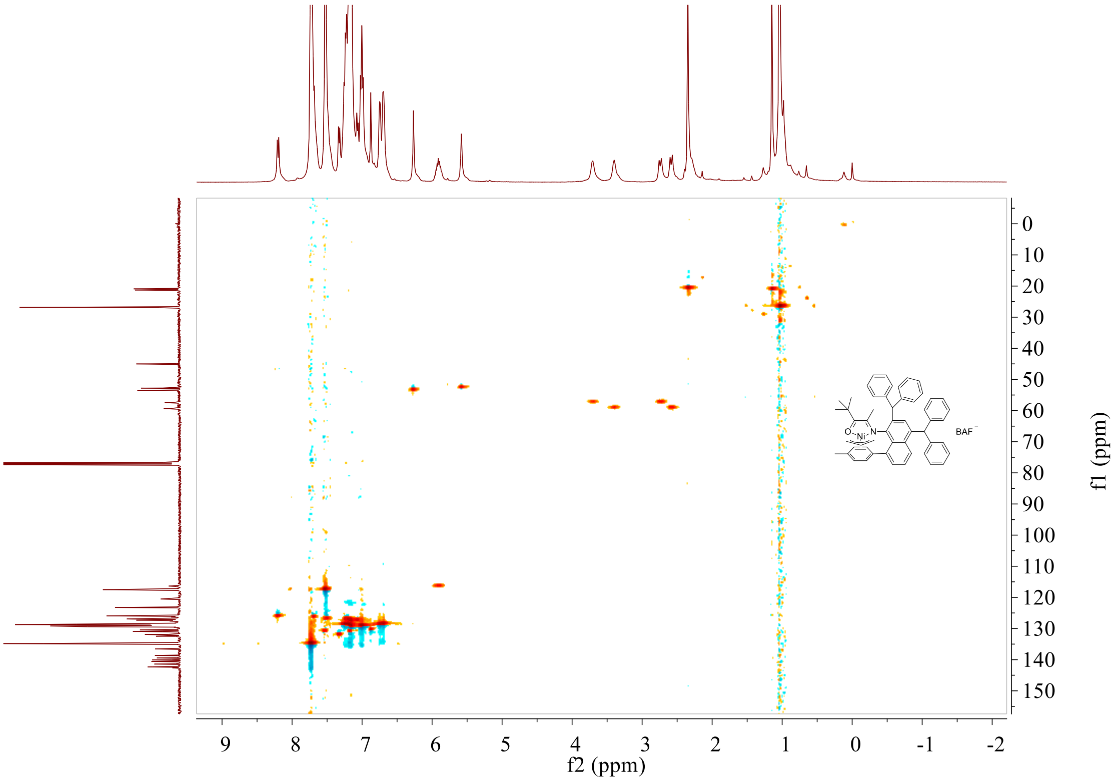

Supplementary Figure 32. ^{1}H-^{13}C HSQC NMR spectrum of **Ni4** in $CDCl_3$.

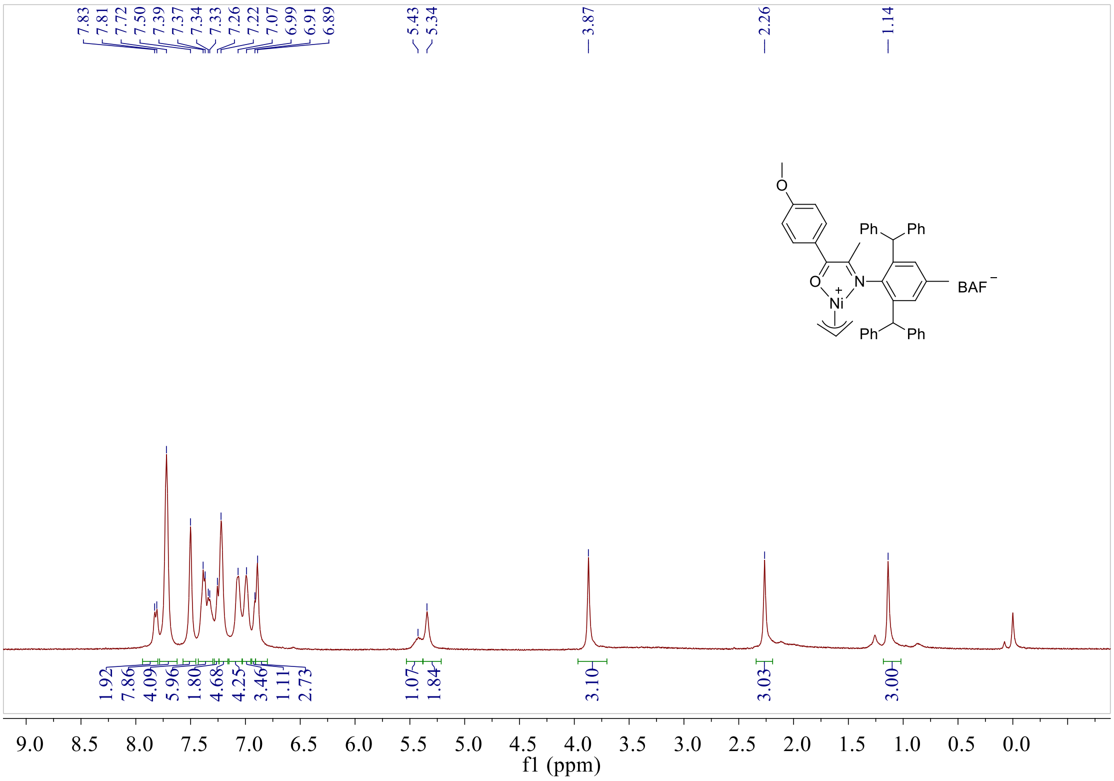

Supplementary Figure 33. ^{1}H NMR spectrum (400 MHz, $CDCl_3$) of **Ni5.**

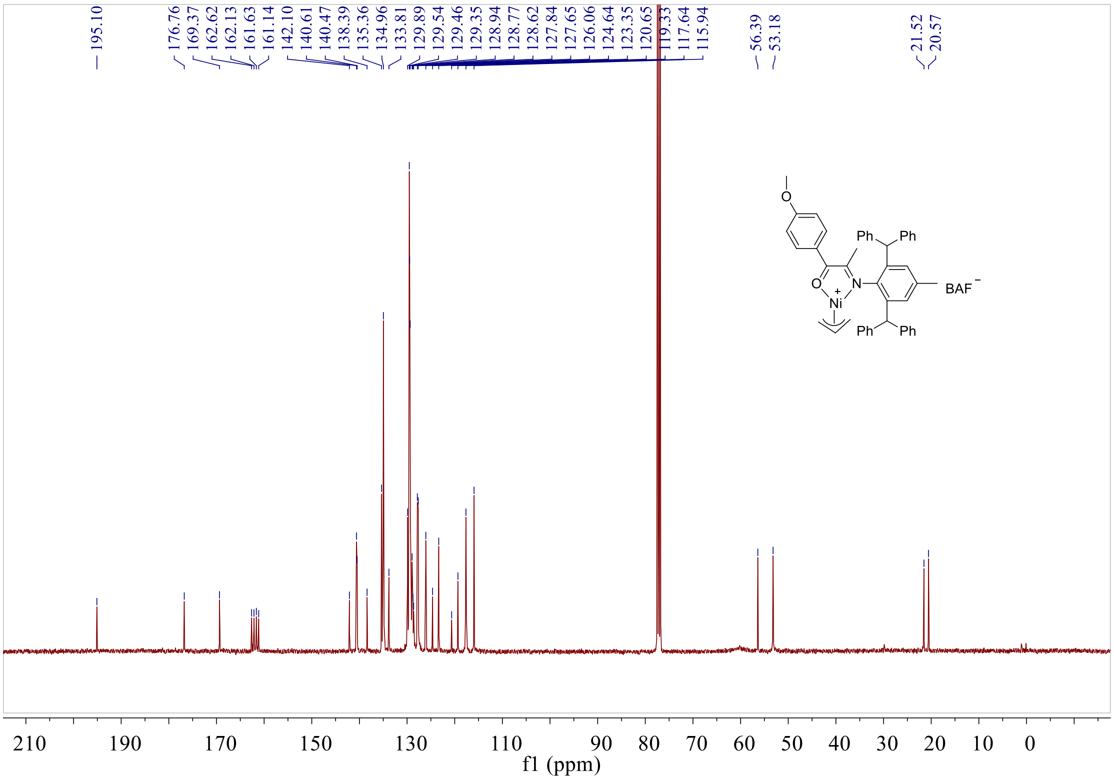

Supplementary Figure 34. ^{13}C NMR spectrum in $CDCl_3$ of **Ni5.**

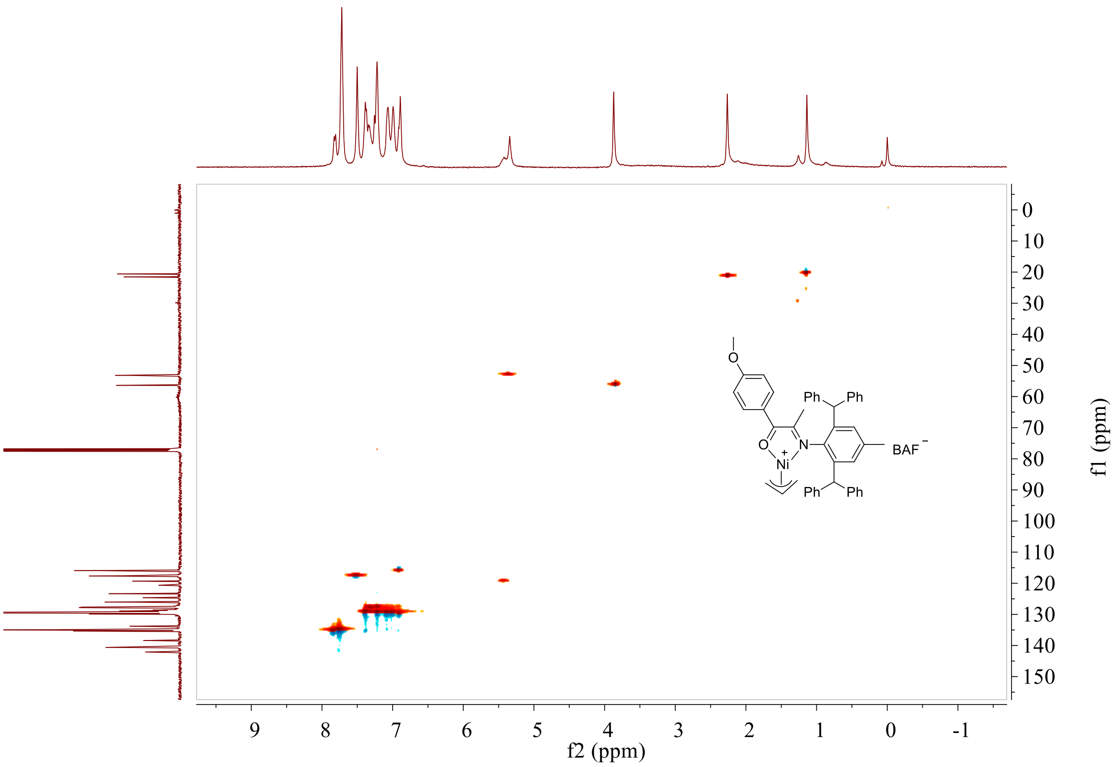

Supplementary Figure 35. ^1H-^{13}C HSQC NMR spectrum of **Ni5** in $CDCl_3$.

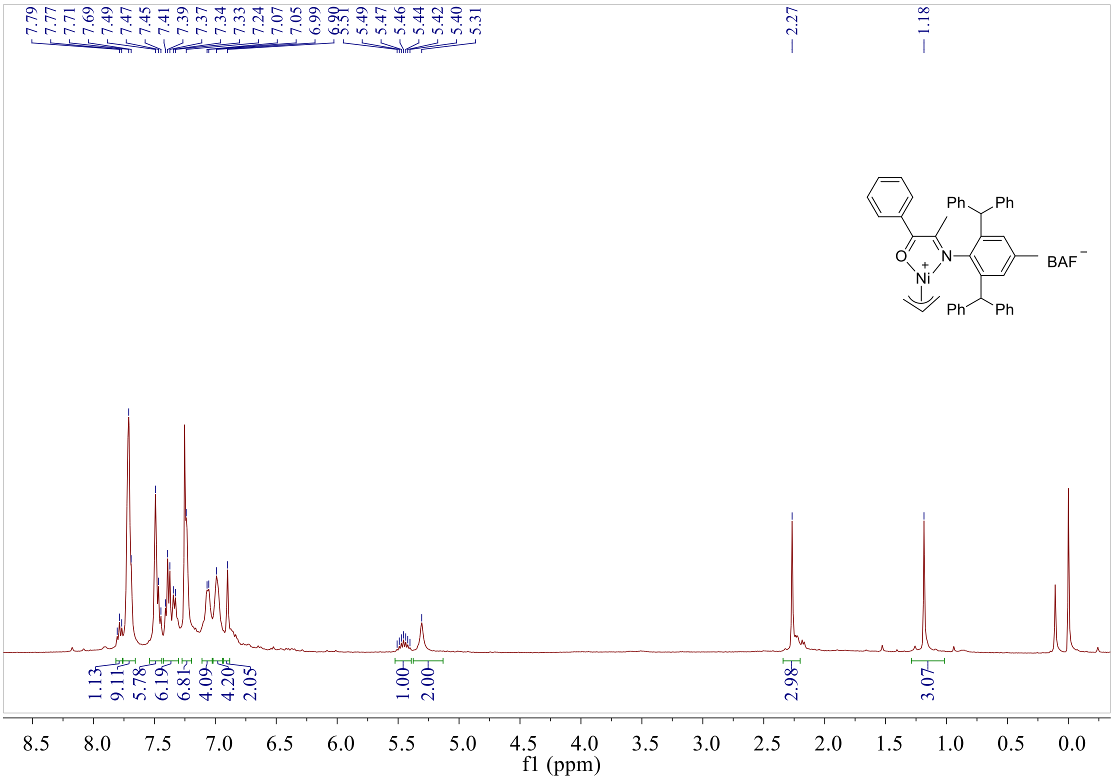

Supplementary Figure 36. ^1H NMR spectrum (400 MHz, $CDCl_3$) of **Ni6.**

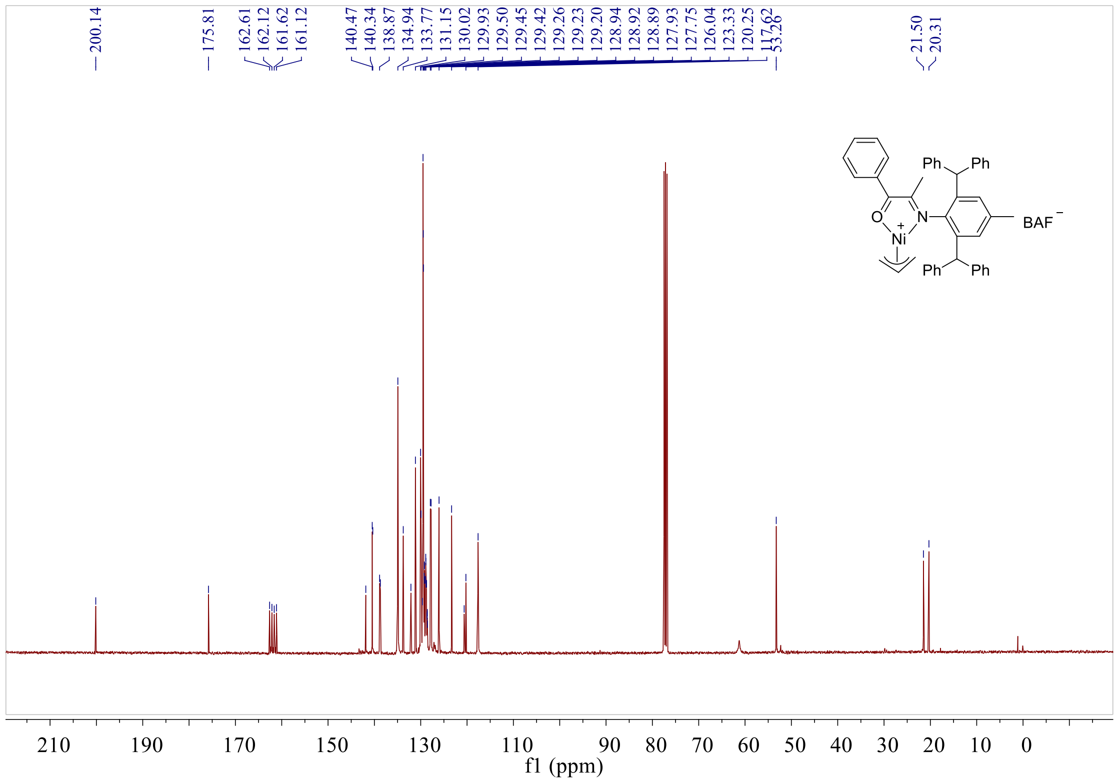

Supplementary Figure 37. ^{13}C NMR spectrum in $CDCl_3$ of **Ni6.**

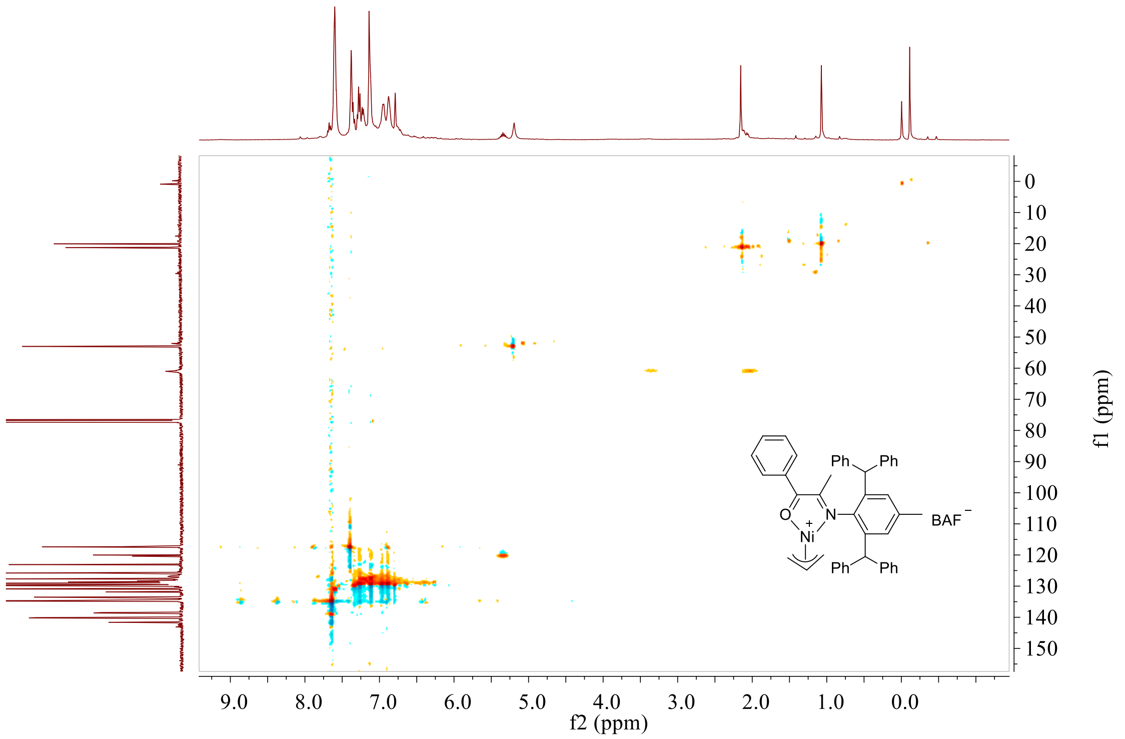

Supplementary Figure 38. ^{1}H-^{13}C HSQC NMR spectrum of **Ni6** in $CDCl_3$.

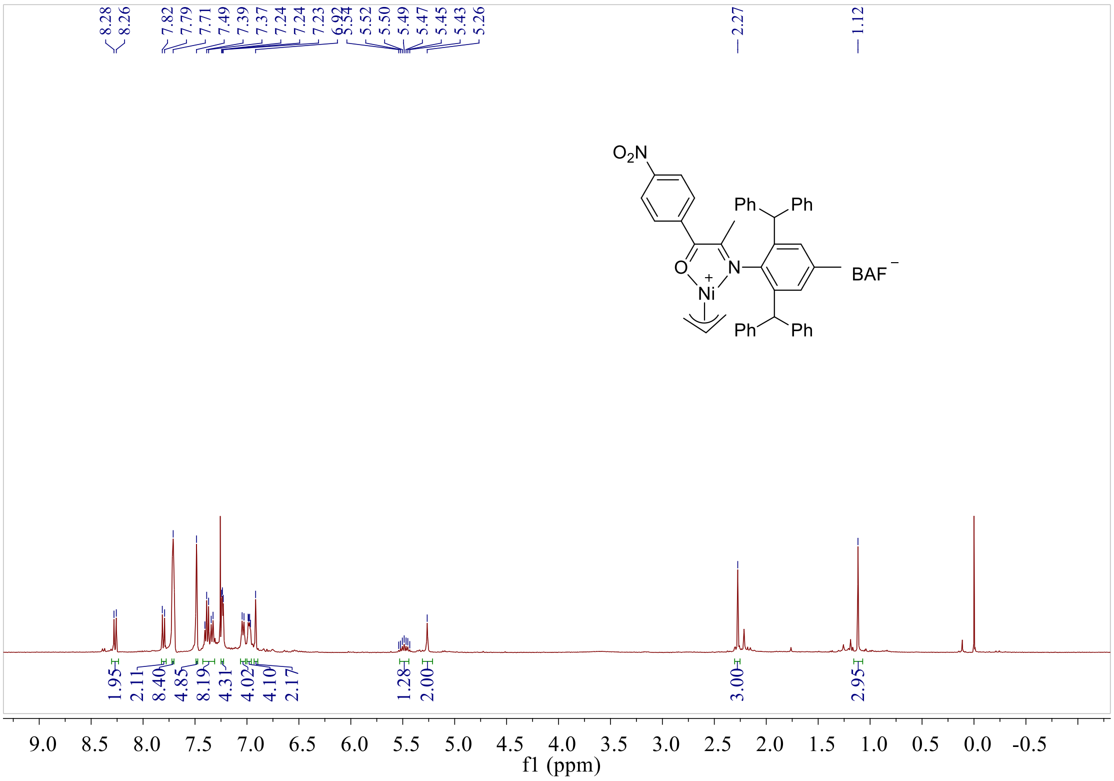

Supplementary Figure 39. ^{1}H NMR spectrum (400 MHz, $CDCl_3$) of **Ni7.**

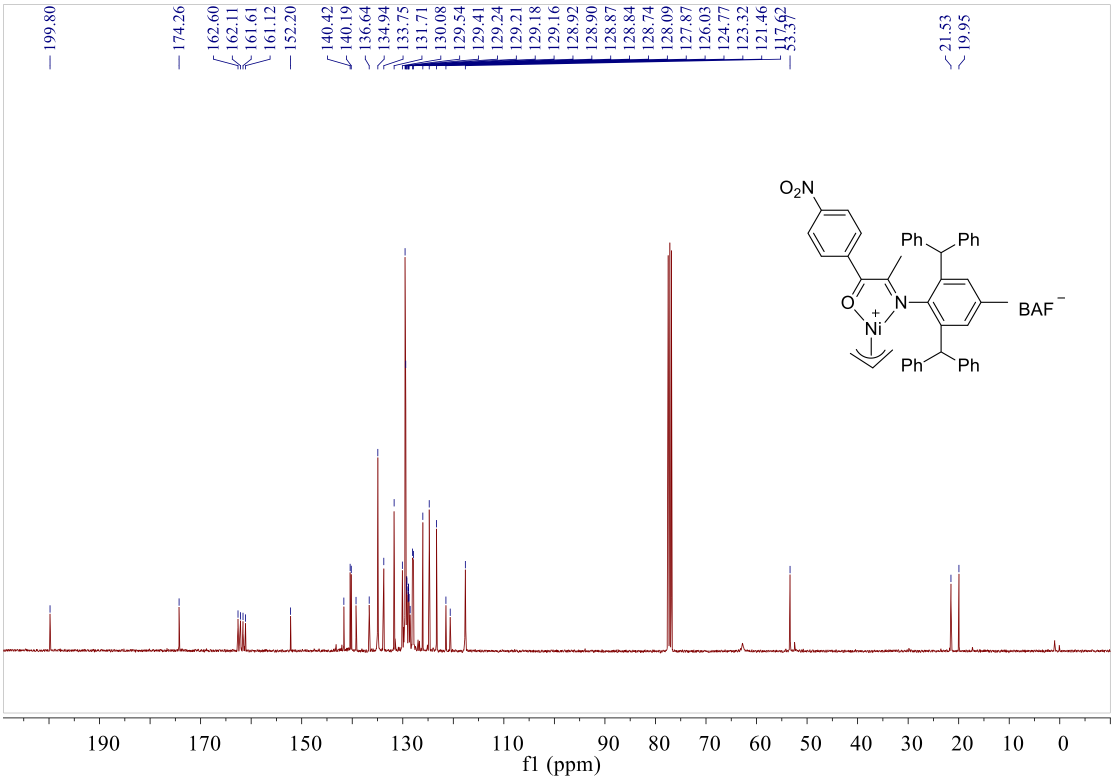

Supplementary Figure 40. ^{13}C NMR spectrum in $CDCl_3$ of **Ni7.**

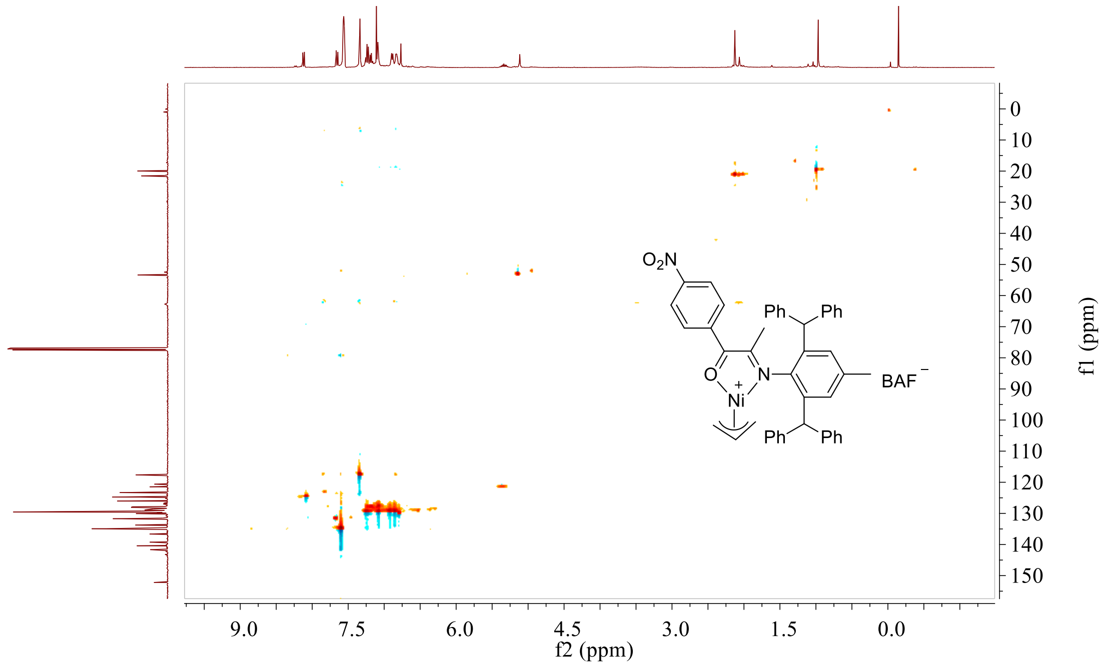

Supplementary Figure 41. ^{1}H-^{13}C HSQC NMR spectrum of **Ni7** in $CDCl_3$.

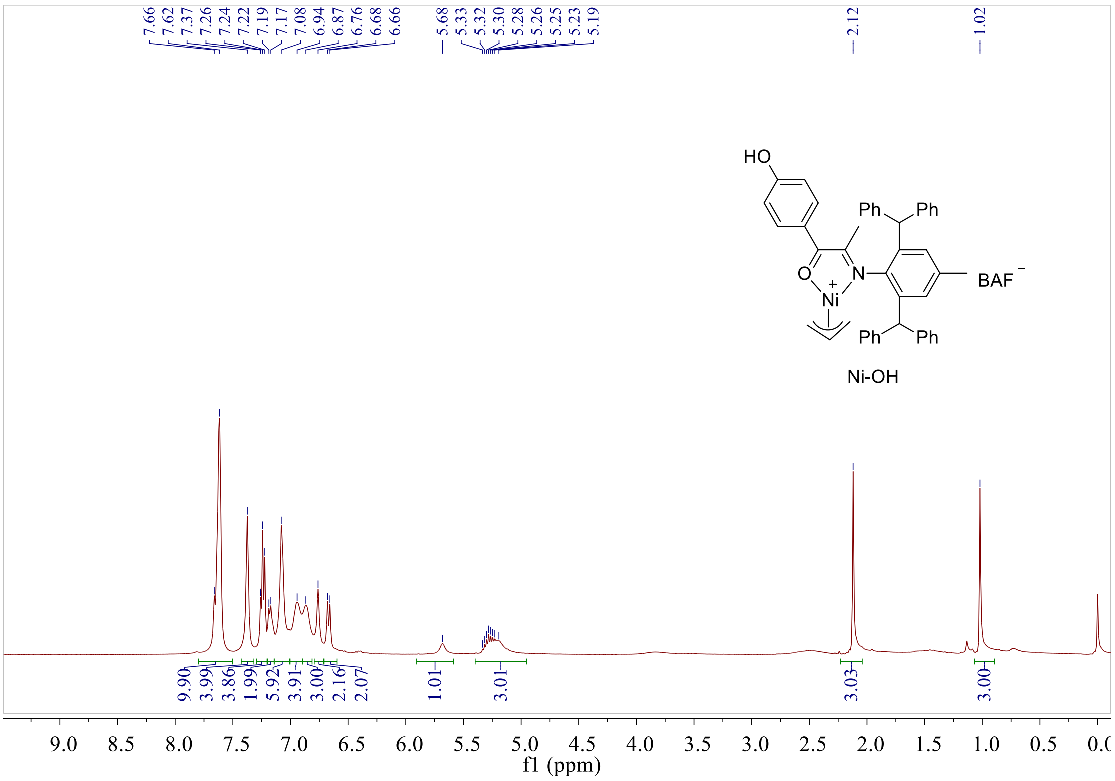

Supplementary Figure 42. ^{1}H NMR spectrum (400 MHz, $CDCl_3$) of **Ni-OH.**

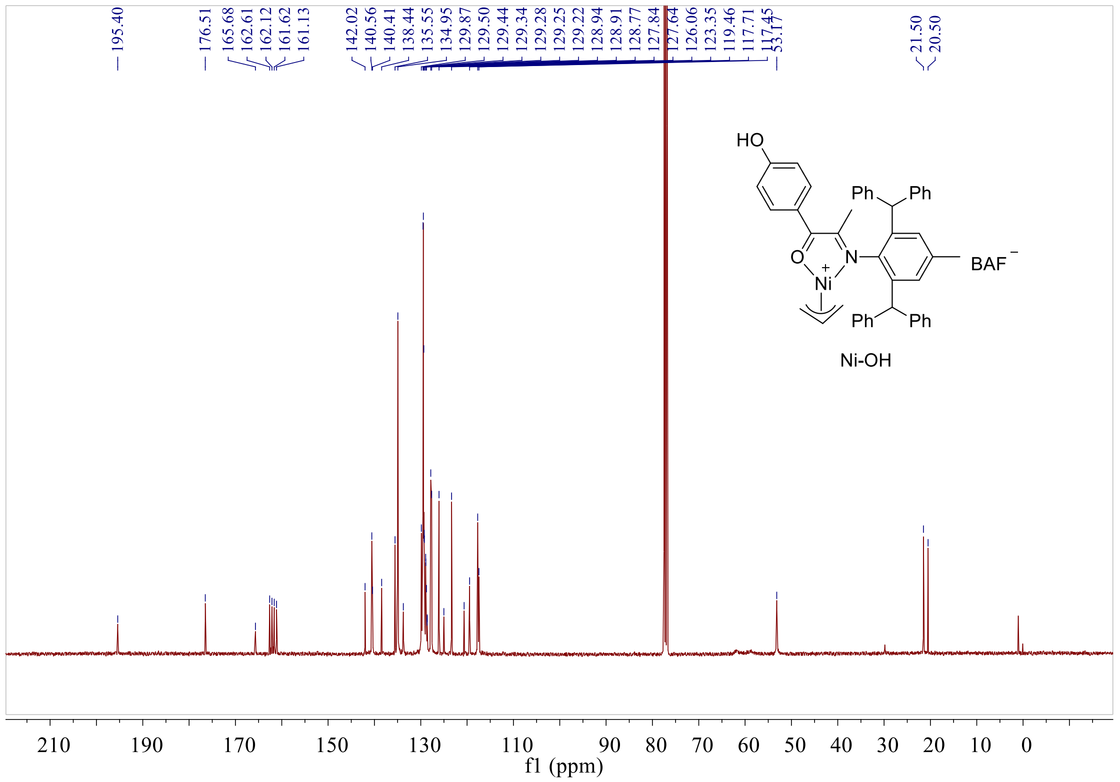

Supplementary Figure 43. ^{13}C NMR spectrum in $CDCl_3$ of **Ni-OH.**

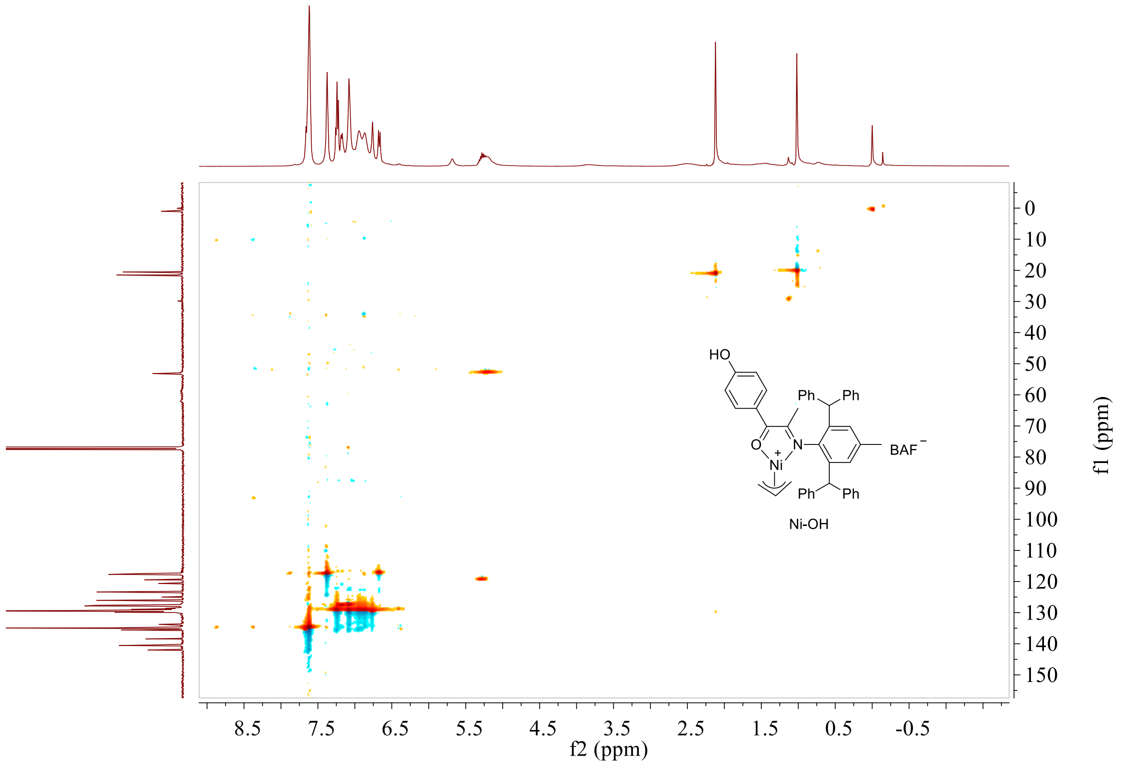

Supplementary Figure 44. ^{1}H-^{13}C HSQC NMR spectrum of **Ni-OH** in $CDCl_3$.

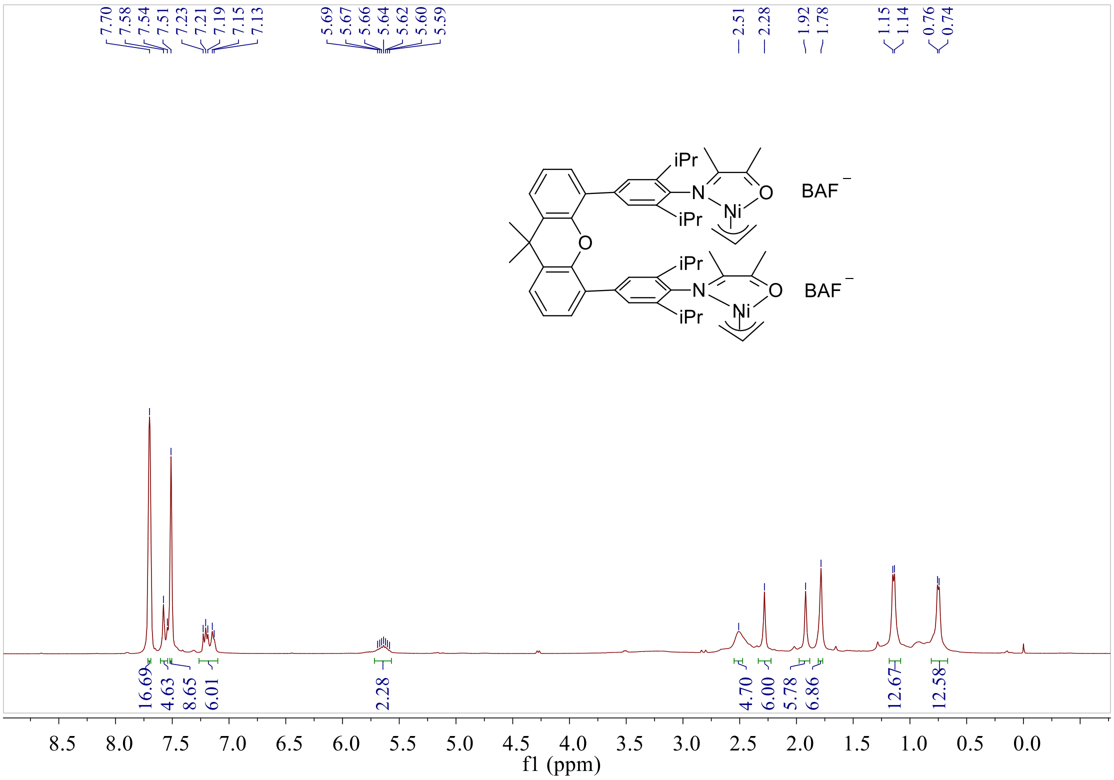

Supplementary Figure 45. ^{1}H NMR spectrum (400 MHz, $CDCl_3$) of **Ni-Ni.**

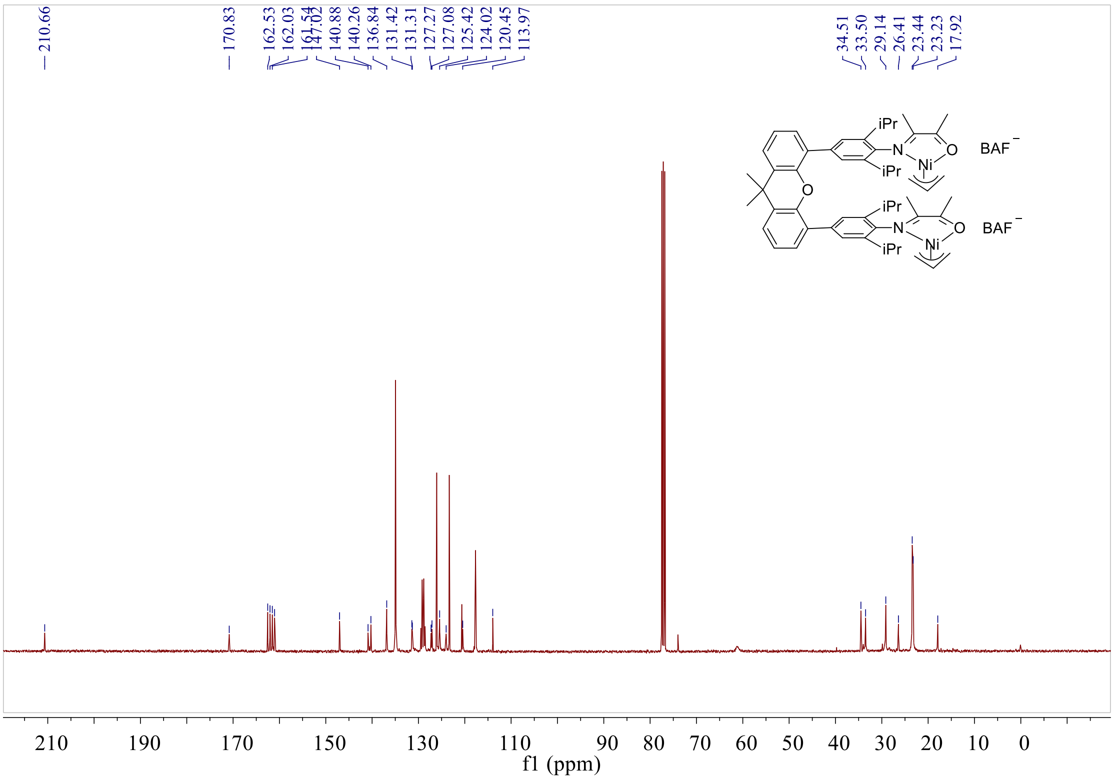

Supplementary Figure 46. ^{13}C NMR spectrum in $CDCl_3$ of **Ni-Ni.**

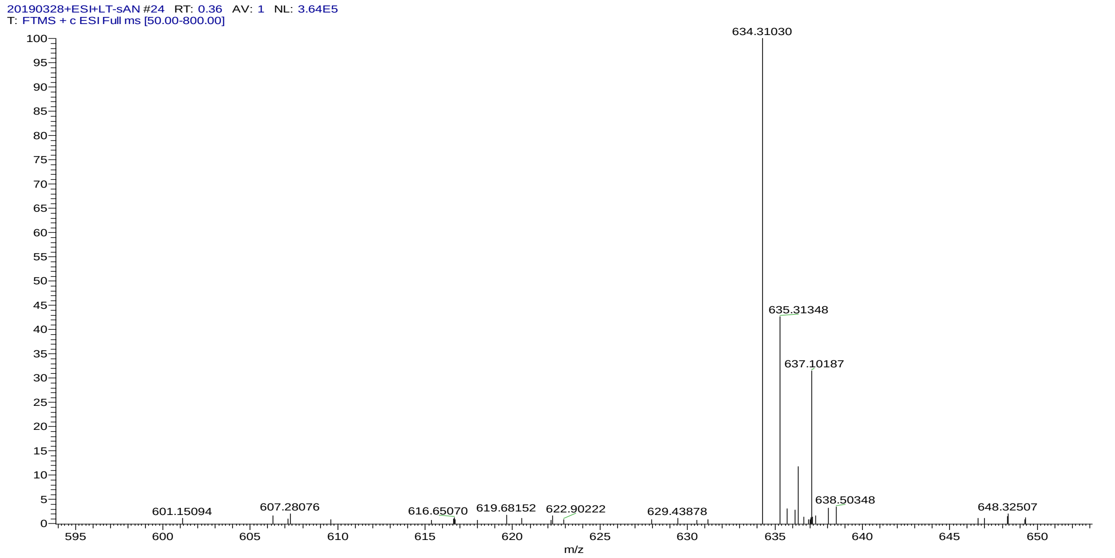

Supplementary Figure 47. ESI-MS of **L3.**

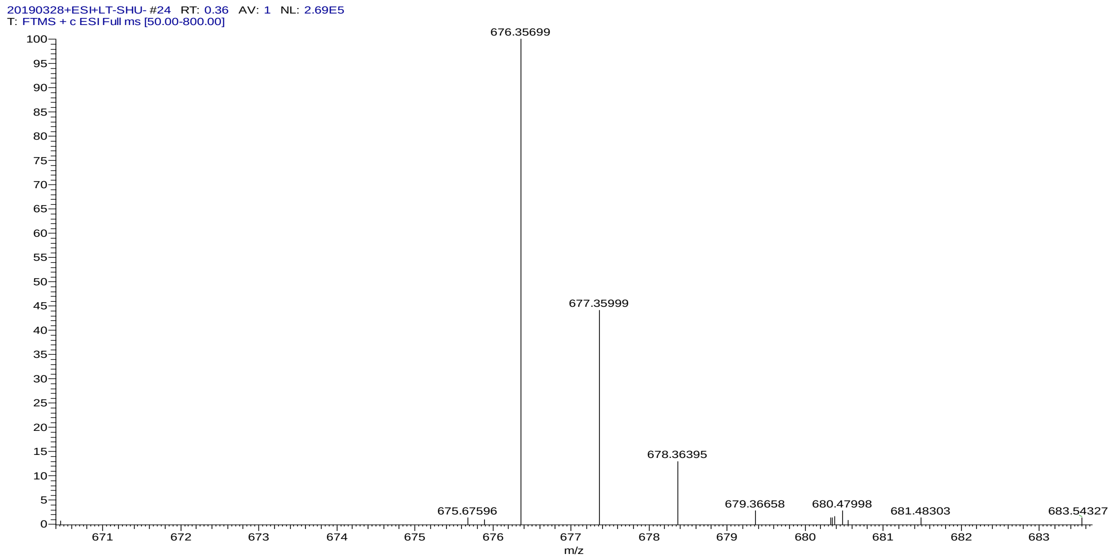

Supplementary Figure 48. ESI-MS of **L4.**

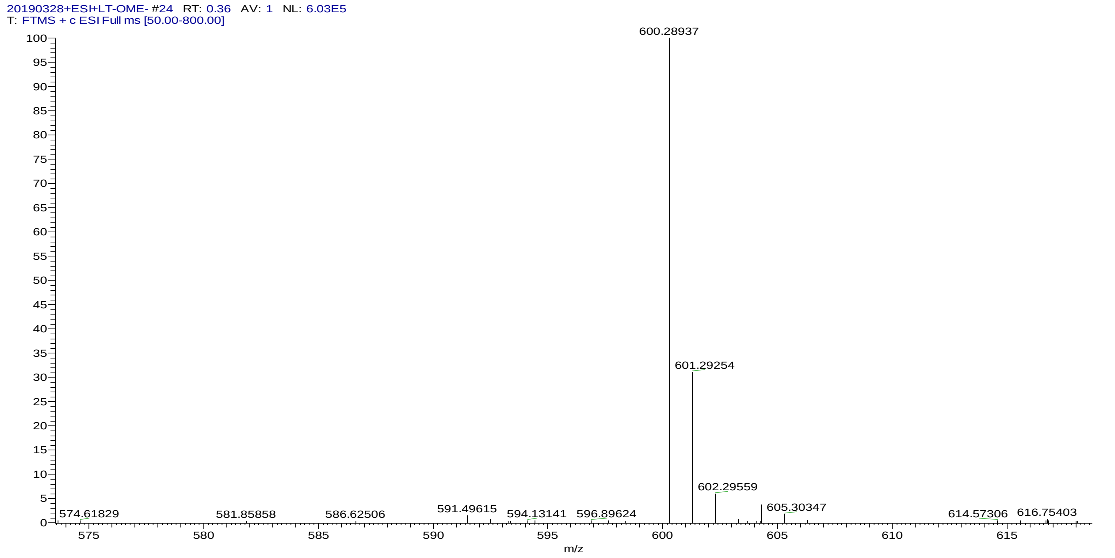

Supplementary Figure 49. ESI-MS of **L5.**

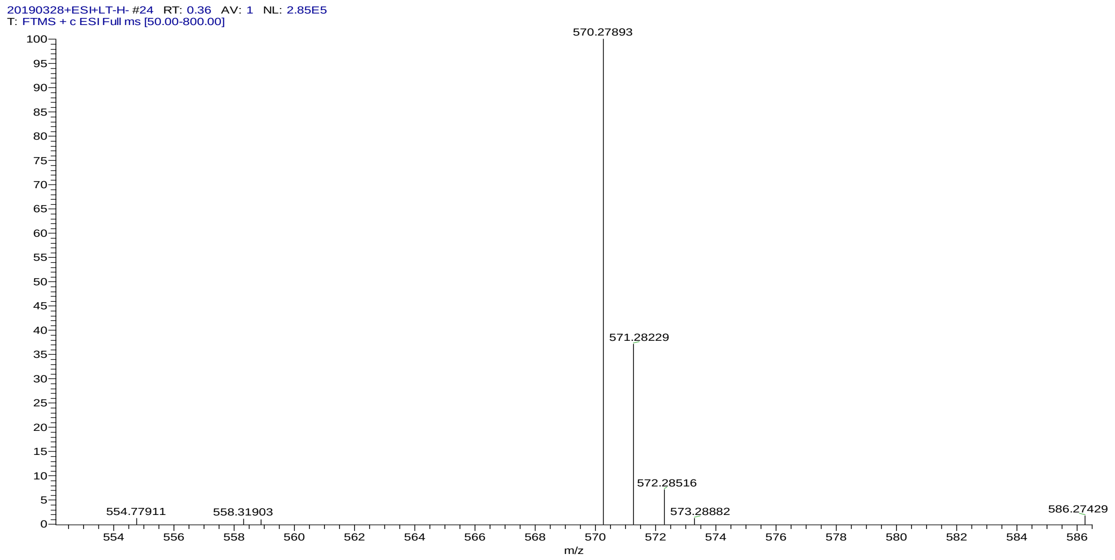

Supplementary Figure 50. ESI-MS of **L6.**

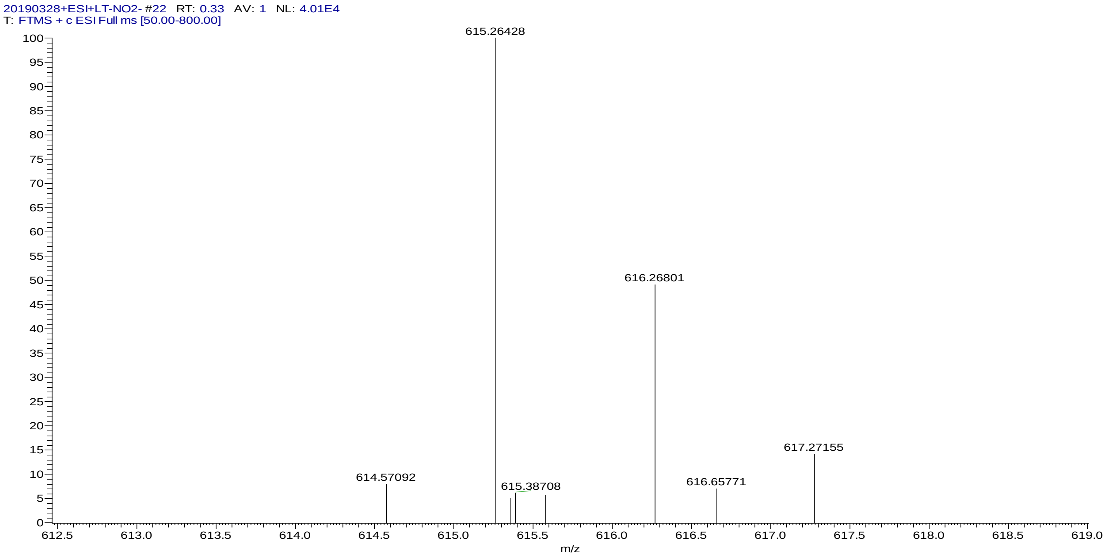

Supplementary Figure 51. ESI-MS of **L7.**

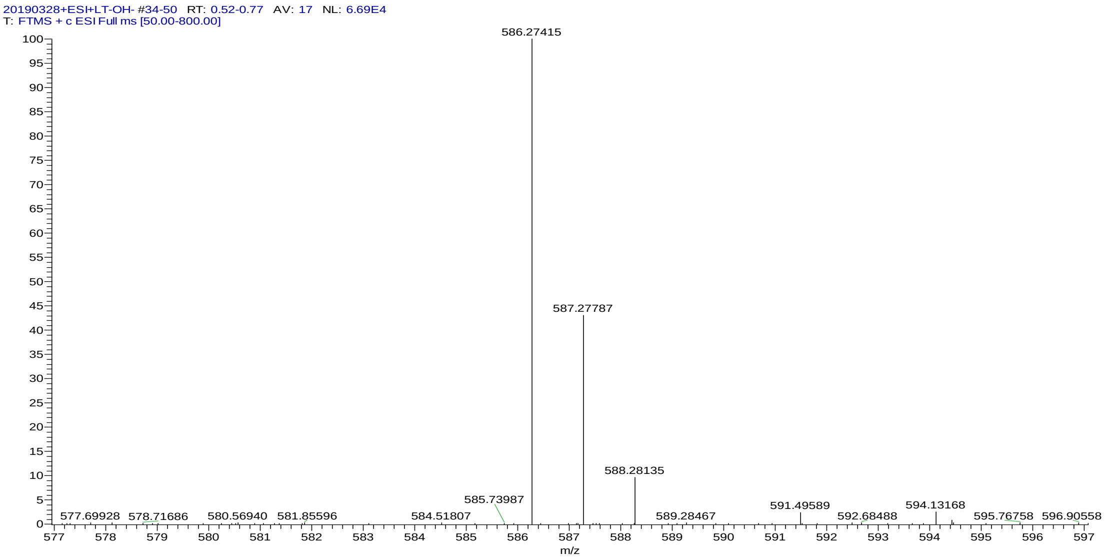

Supplementary Figure 52. ESI-MS of **L-OH.**

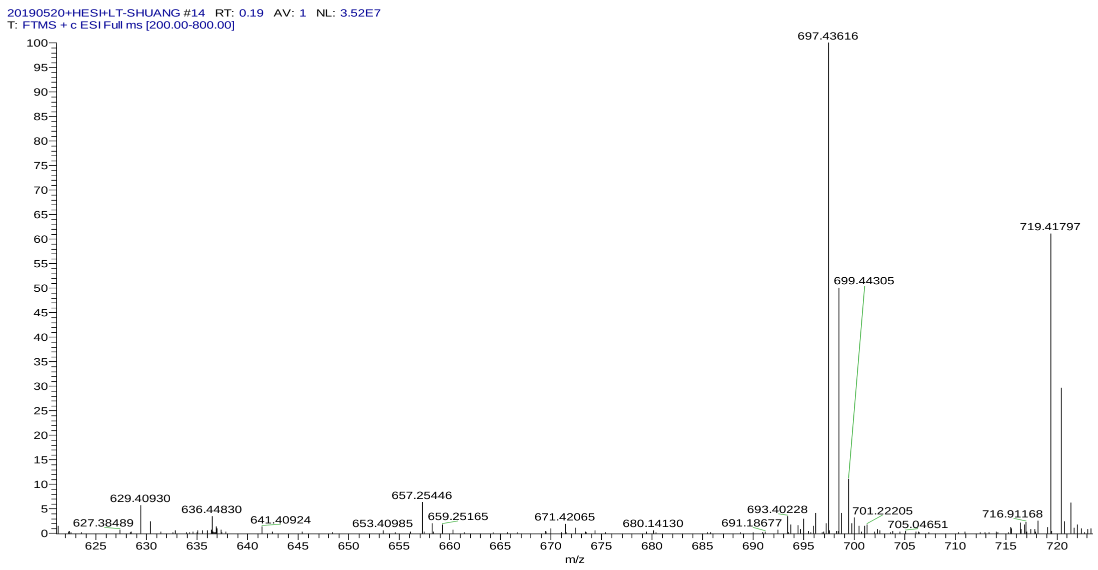

Supplementary Figure 53. ESI-MS of **L-L.**

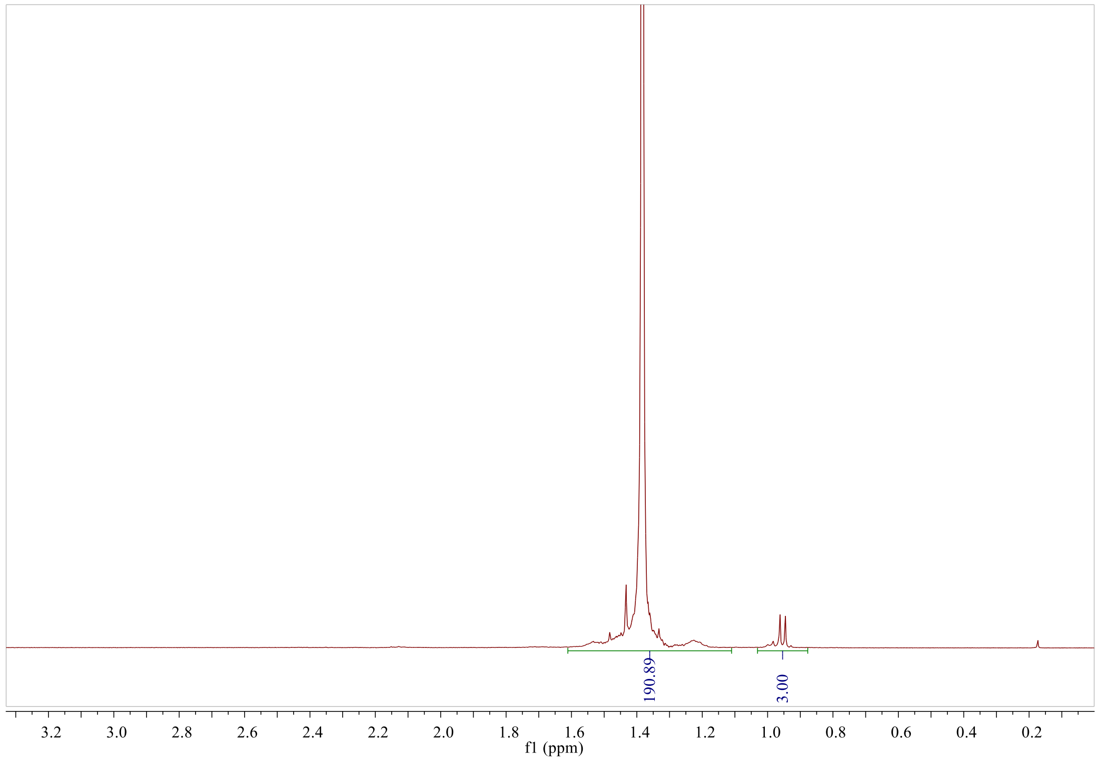

Supplementary Figure 54. ^{1}H NMR spectrum of the polymer from table 1, entry 1 ($C_2D_2Cl_4$, 120 °C).

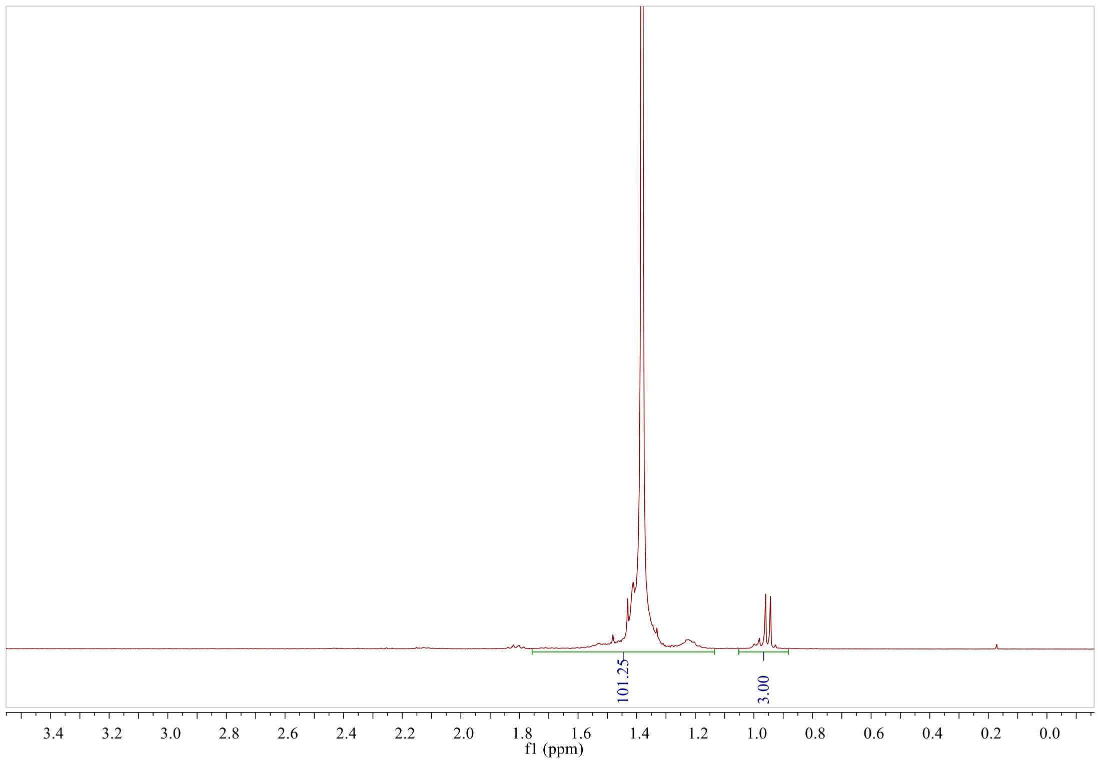

Supplementary Figure 55. ^{1}H NMR spectrum of the polymer from table 1, entry 2 ($C_2D_2Cl_4$, 120 °C).

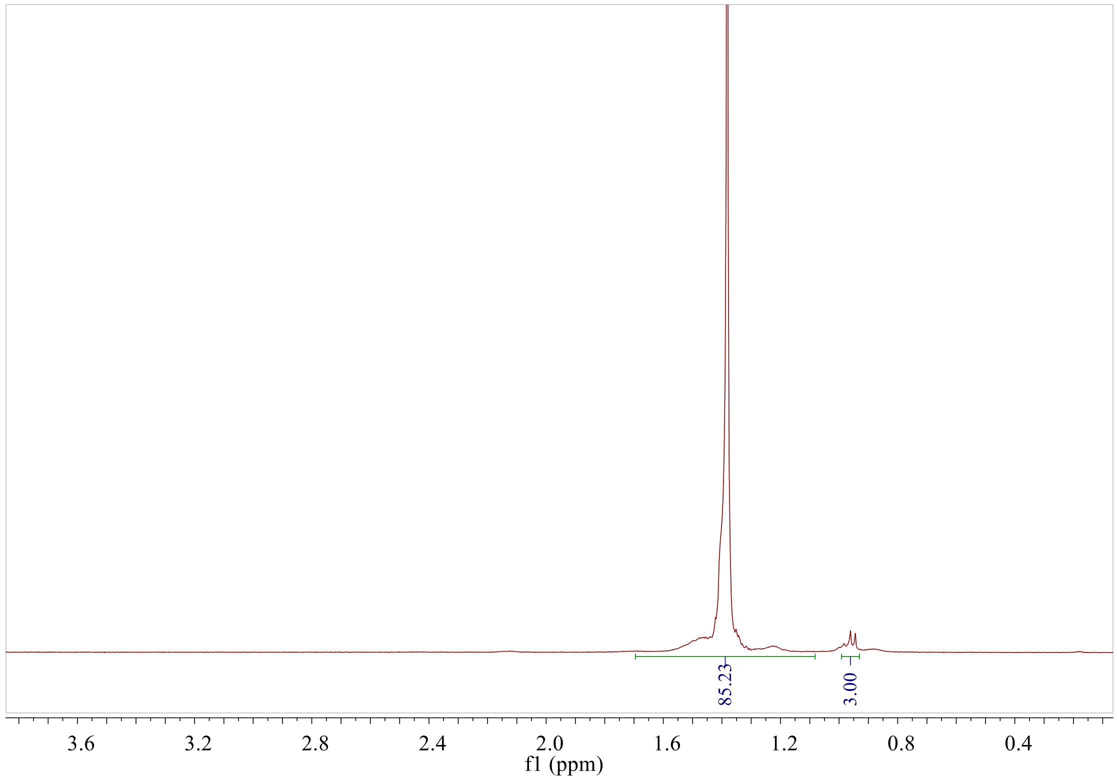

Supplementary Figure 56. ^{1}H NMR spectrum of the copolymer from table 1, entry 3 ($C_2D_2Cl_4$, 120 °C).

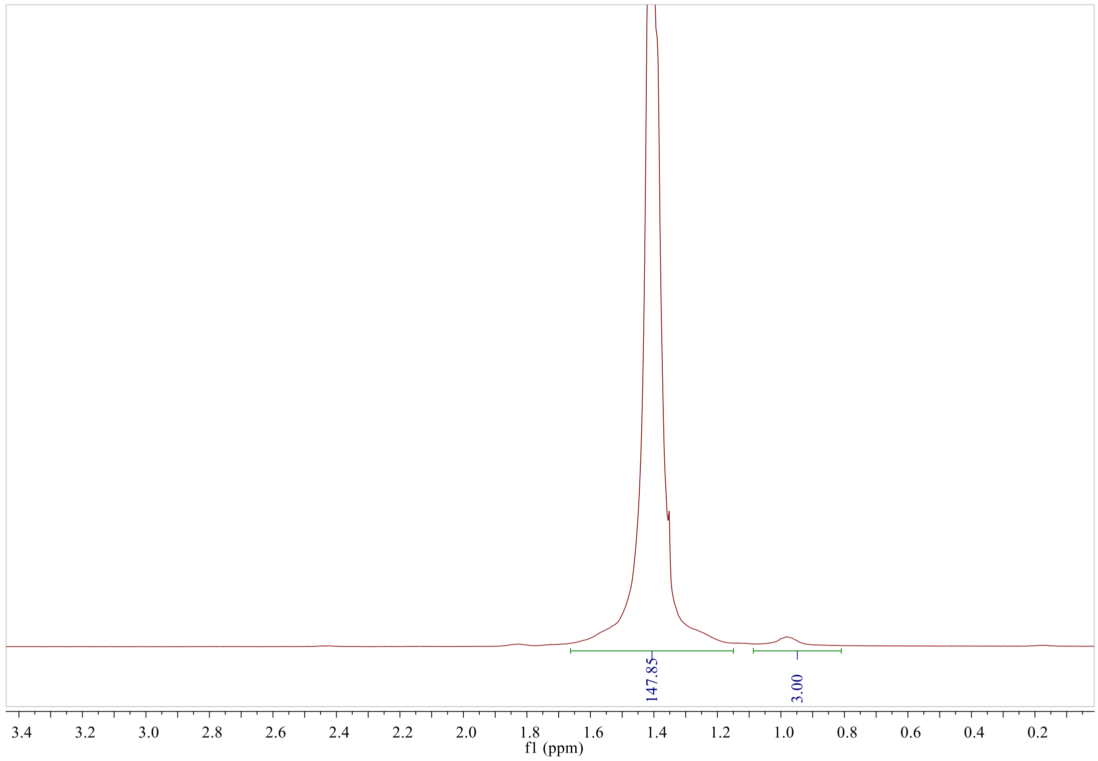

Supplementary Figure 57. ^{1}H NMR spectrum of the polymer from table 1, entry 4 ($C_2D_2Cl_4$, 120 °C).

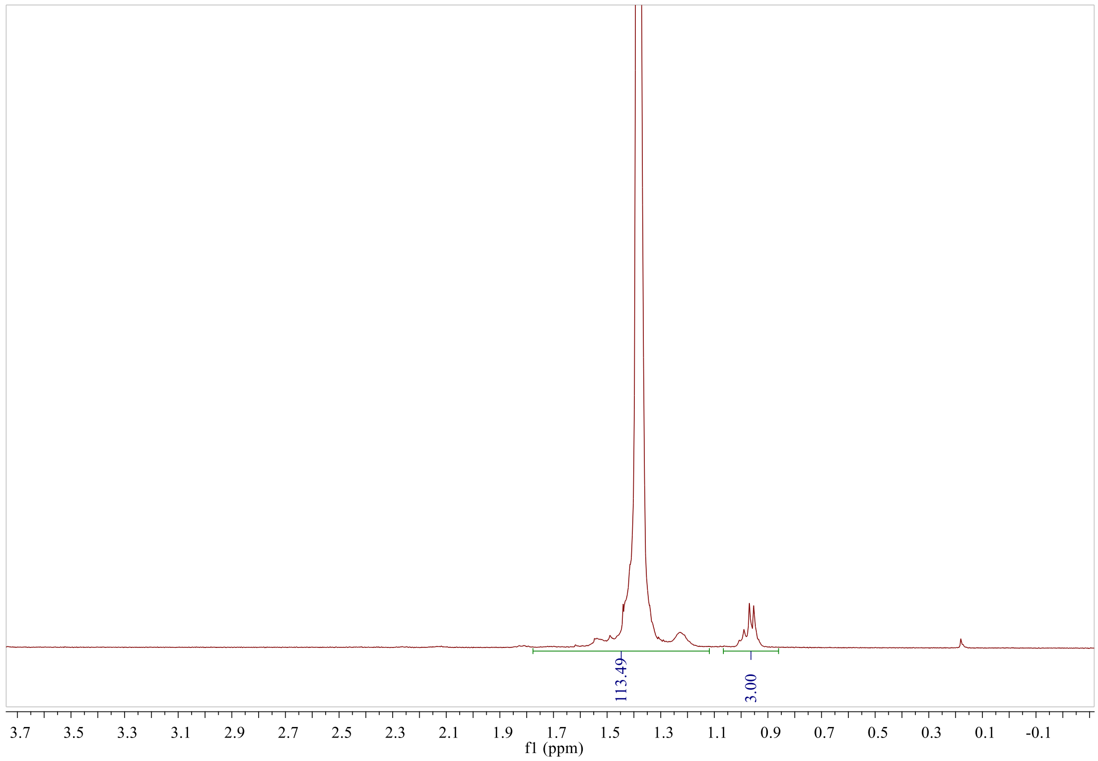

Supplementary Figure 58..^{1}H NMR spectrum of the polymer from table 1, entry 5 ($C_2D_2Cl_4$, 120 °C).

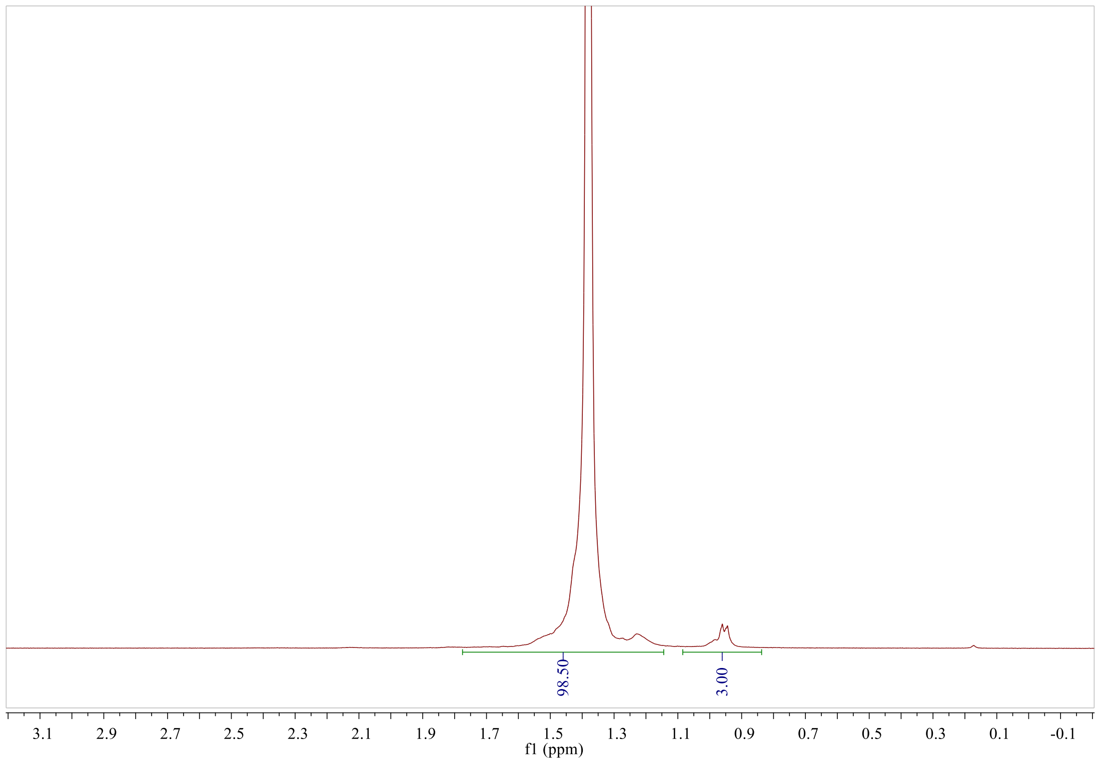

Supplementary Figure 59. ^{1}H NMR spectrum of the polymer from table 1, entry 6 ($C_2D_2Cl_4$, 120 °C).

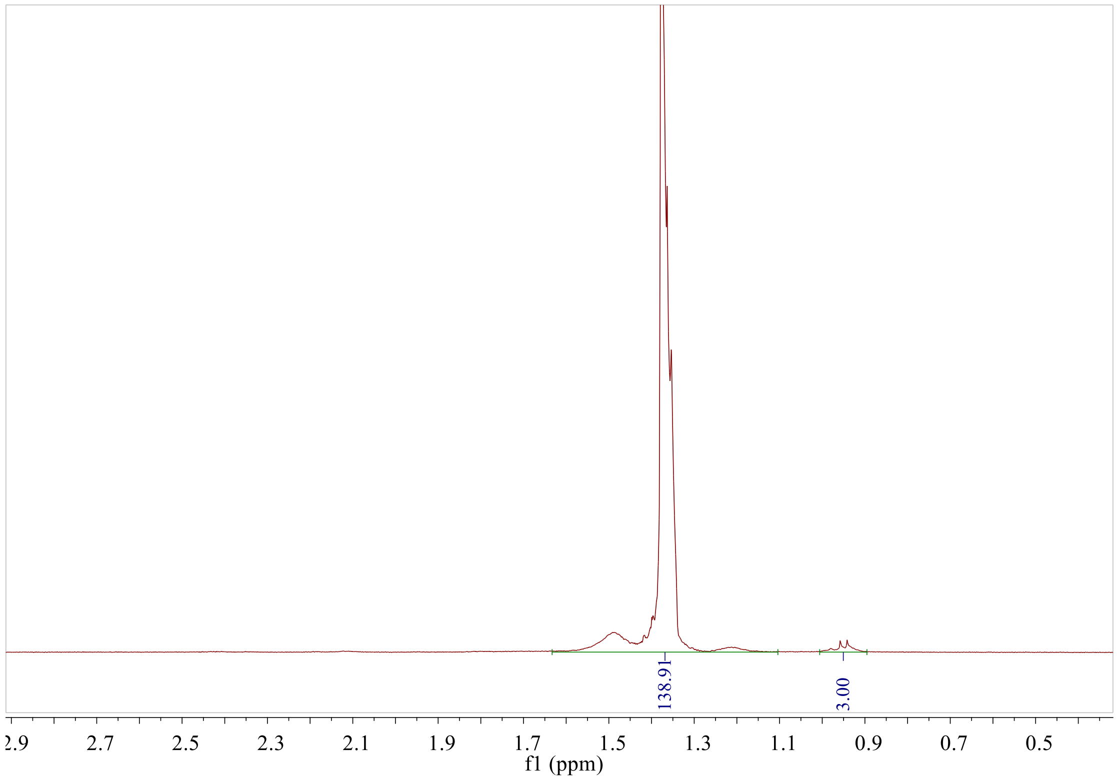

Supplementary Figure 60. ^{1}H NMR spectrum of the polymer from table 1, entry 7 ($C_2D_2Cl_4$, 120 °C).

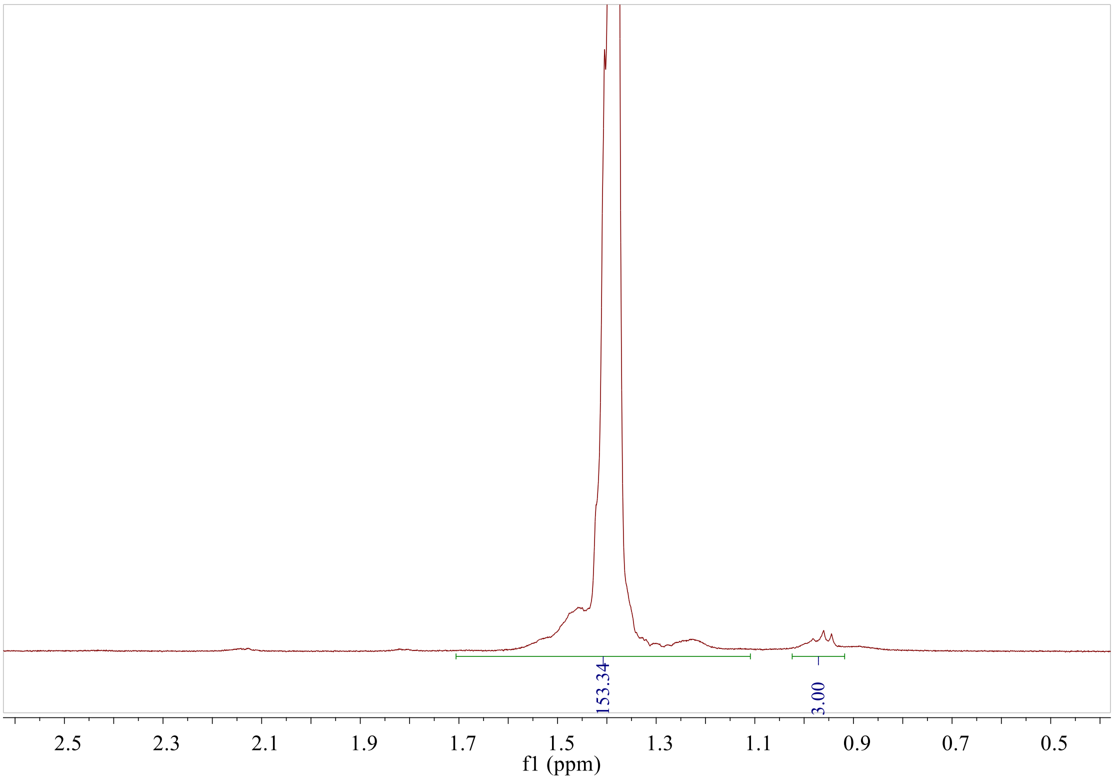

Supplementary Figure 61. ^{1}H NMR spectrum of the polymer from table 1, entry 8 ($C_2D_2Cl_4$, 120 °C).

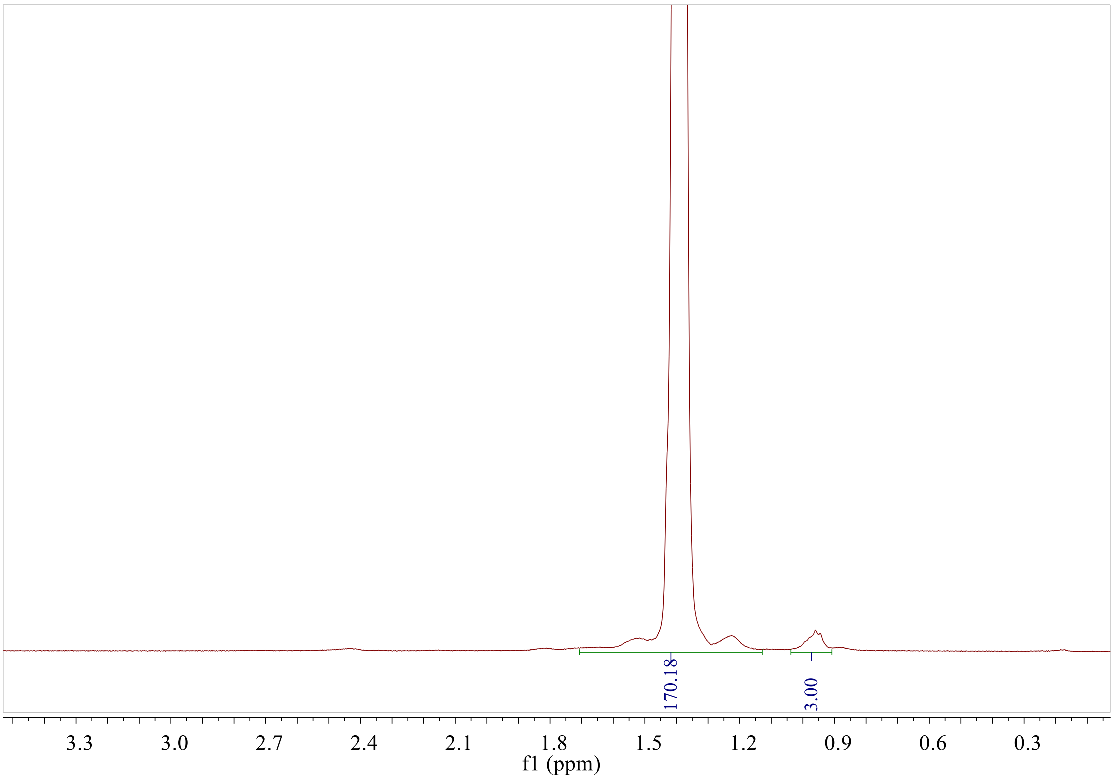

Supplementary Figure 62. ^{1}H NMR spectrum of the polymer from table 1, entry 9 ($C_2D_2Cl_4$, 120 °C).

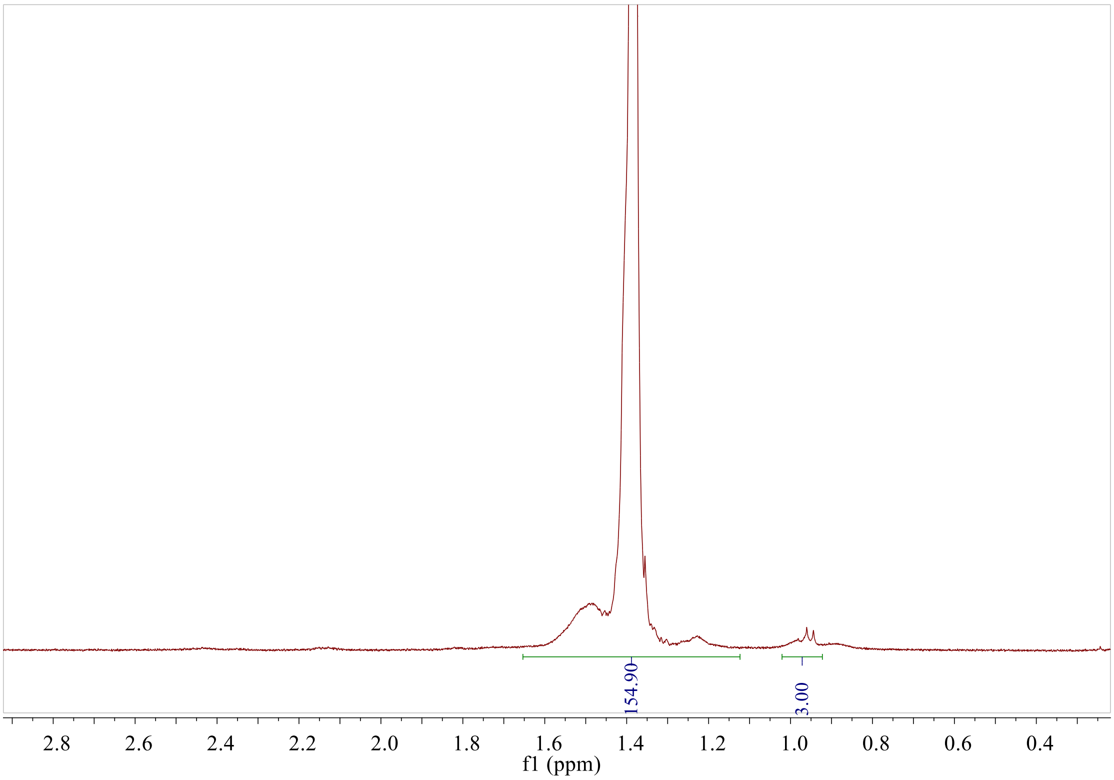

Supplementary Figure 63. ^1H NMR spectrum of the polymer from table 1, entry 10 ($C_2D_2Cl_4$, 120 °C).

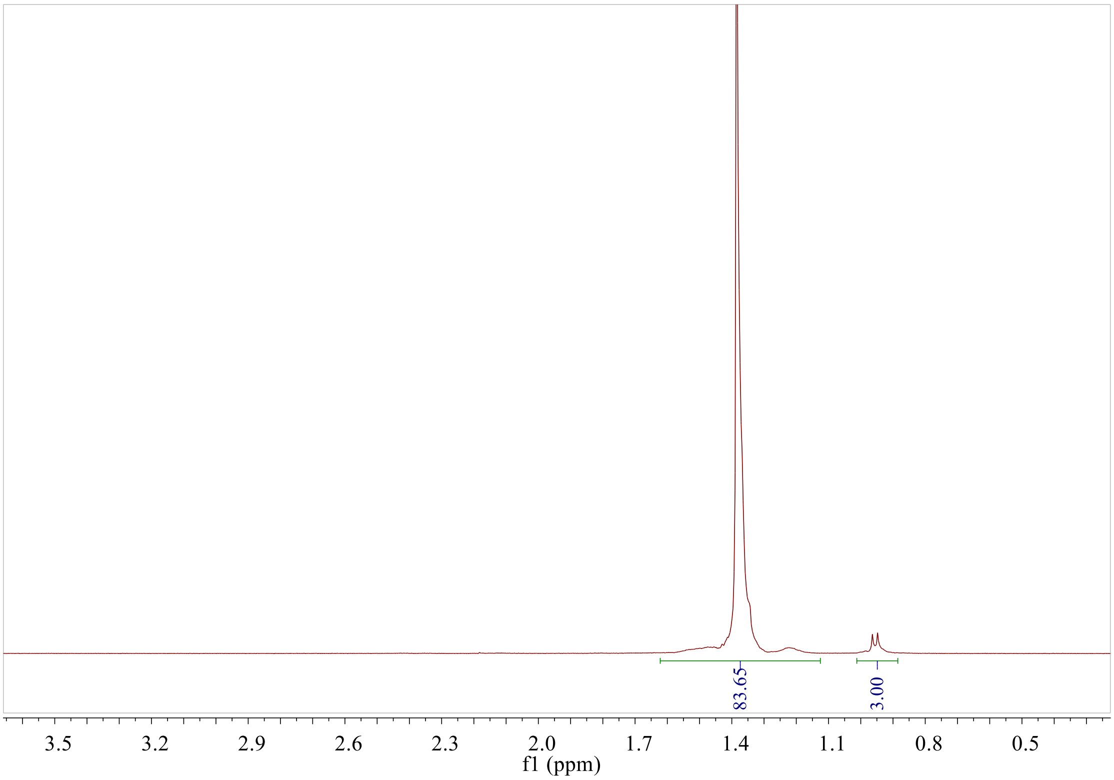

Supplementary Figure 64. ^1H NMR spectrum of the polymer from table 1, entry 11 ($C_2D_2Cl_4$, 120 °C).

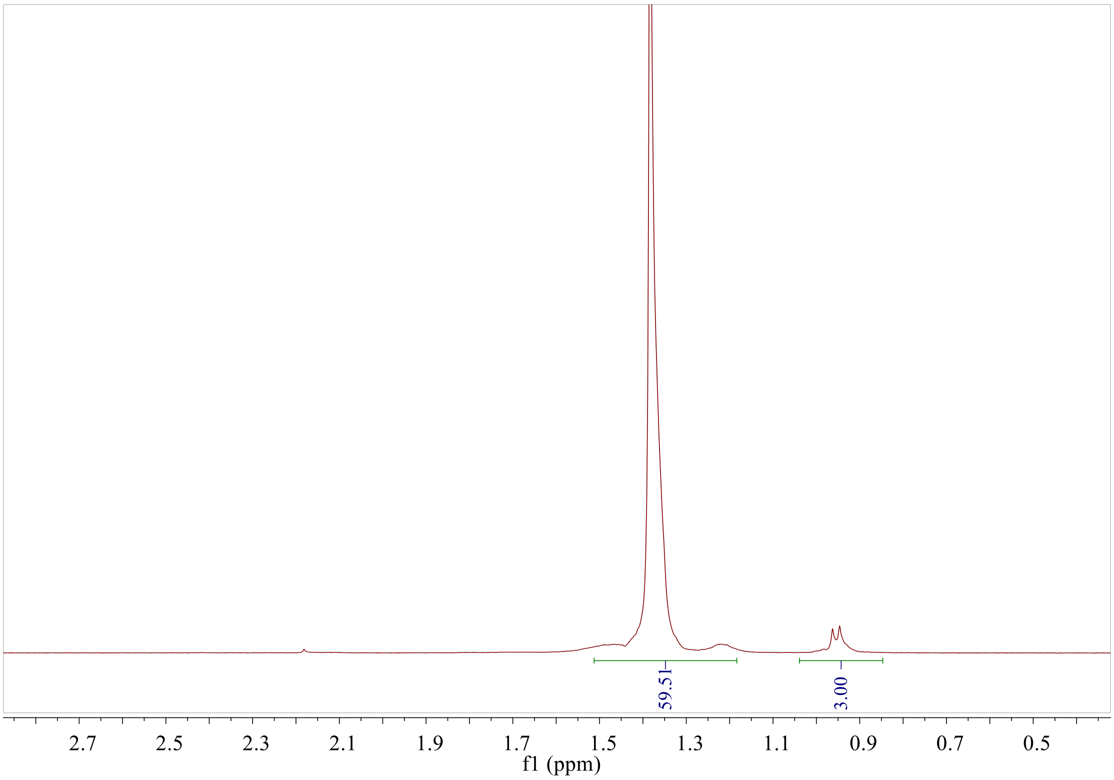

Supplementary Figure 65. ^{1}H NMR spectrum of the polymer from table 1, entry 12 ($C_2D_2Cl_4$, 120 °C).

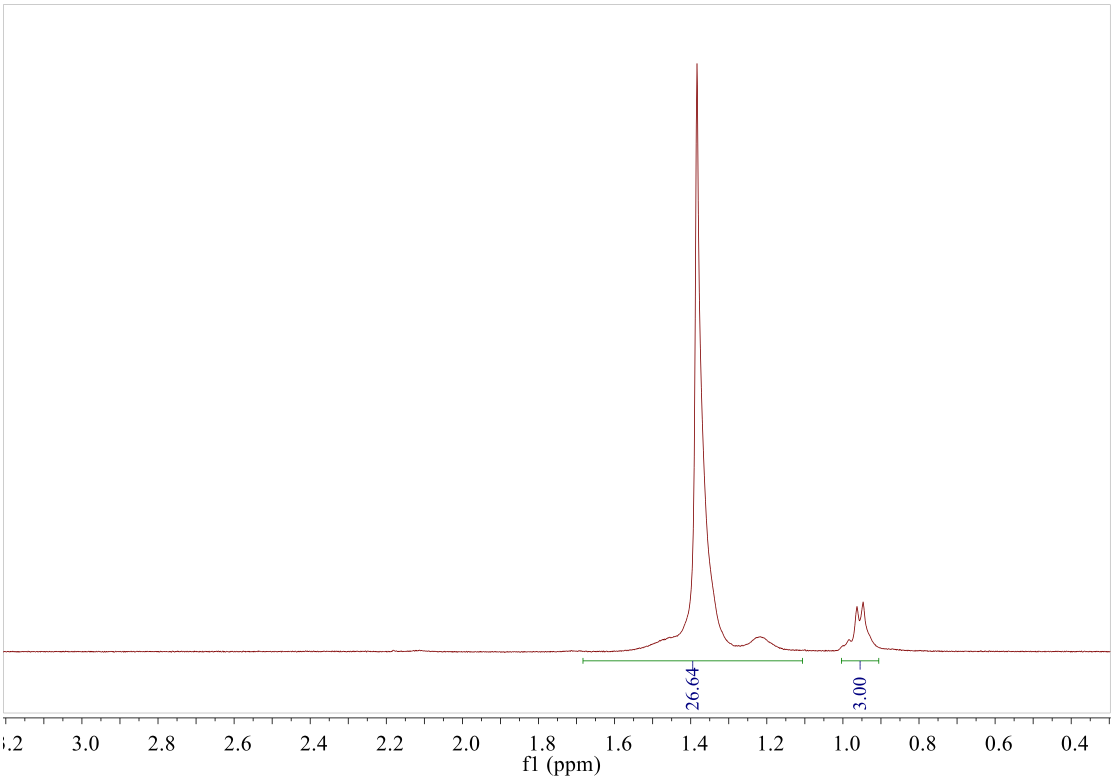

Supplementary Figure 66. ^{1}H NMR spectrum of the polymer from table 1, entry 13 ($C_2D_2Cl_4$, 120 °C).

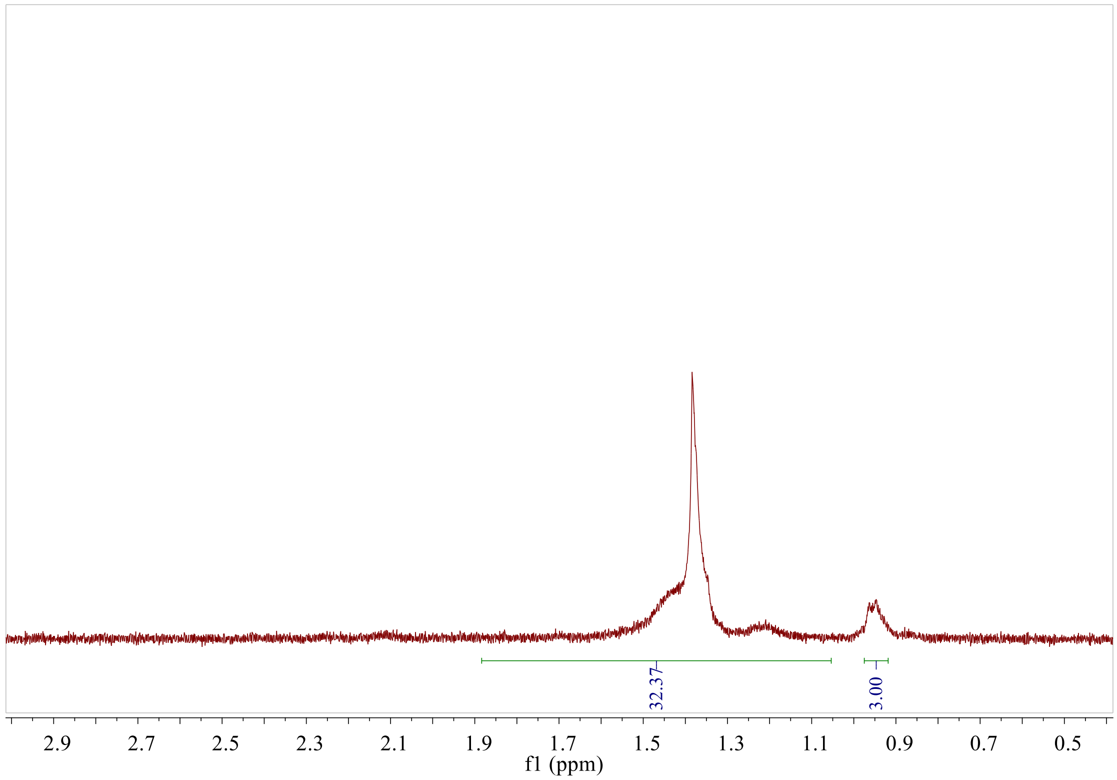

Supplementary Figure 67. ^{1}H NMR spectrum of the polymer from table 1, entry 14 ($C_2D_2Cl_4$, 120 °C).

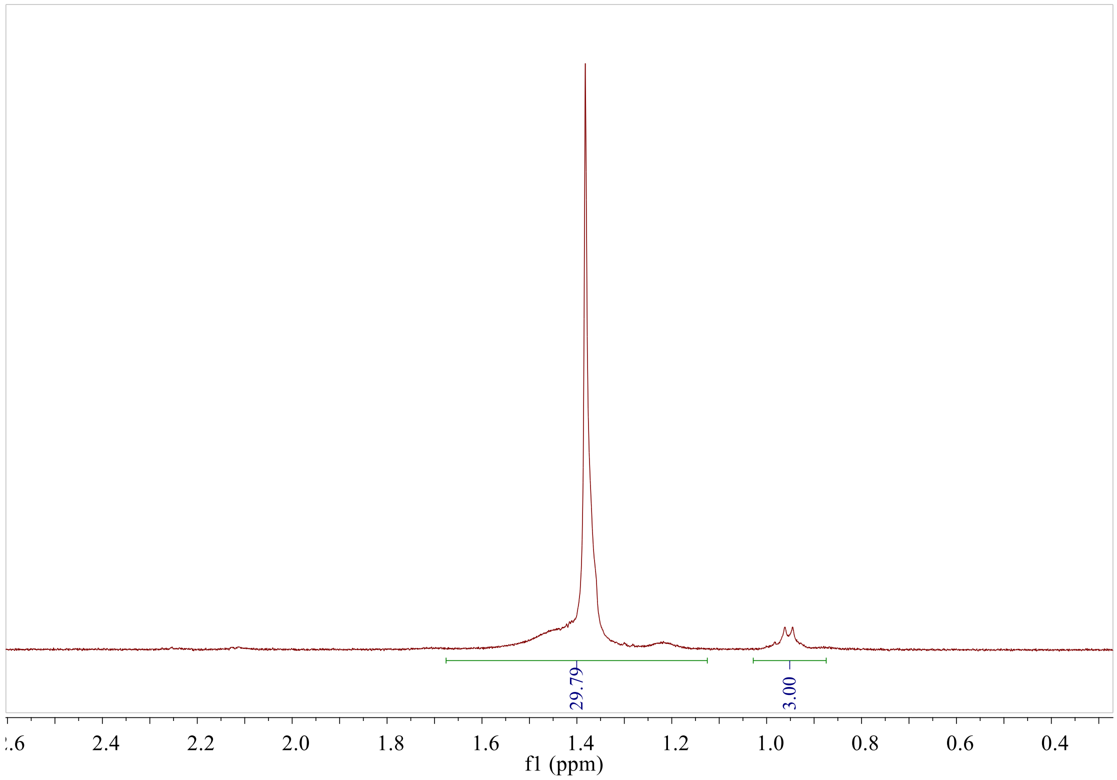

Supplementary Figure 68. ^{1}H NMR spectrum of the polymer from table 1, entry 15 ($C_2D_2Cl_4$, 120 °C).

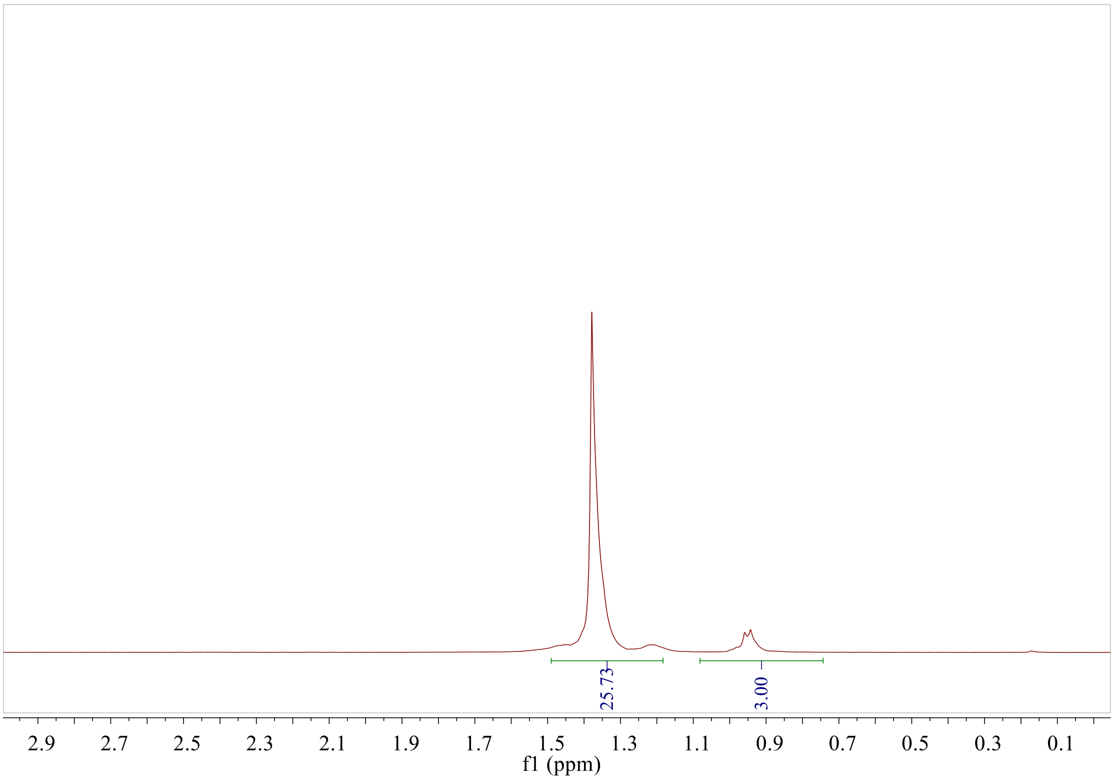

Supplementary Figure 69. ^{1}H NMR spectrum of the polymer from table 1, entry 16 ($C_2D_2Cl_4$, 120 °C).

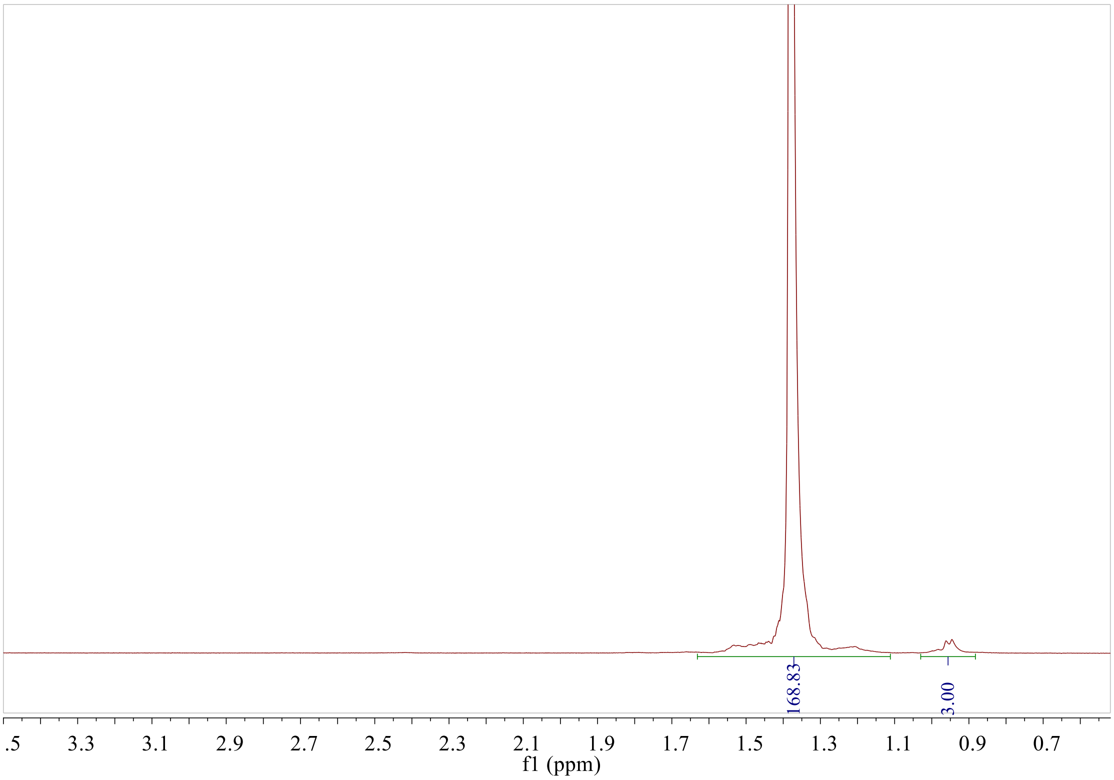

Supplementary Figure 70. ^{1}H NMR spectrum of the polymer from supplementary table 1, entry 1 ($C_2D_2Cl_4$, 120 °C).

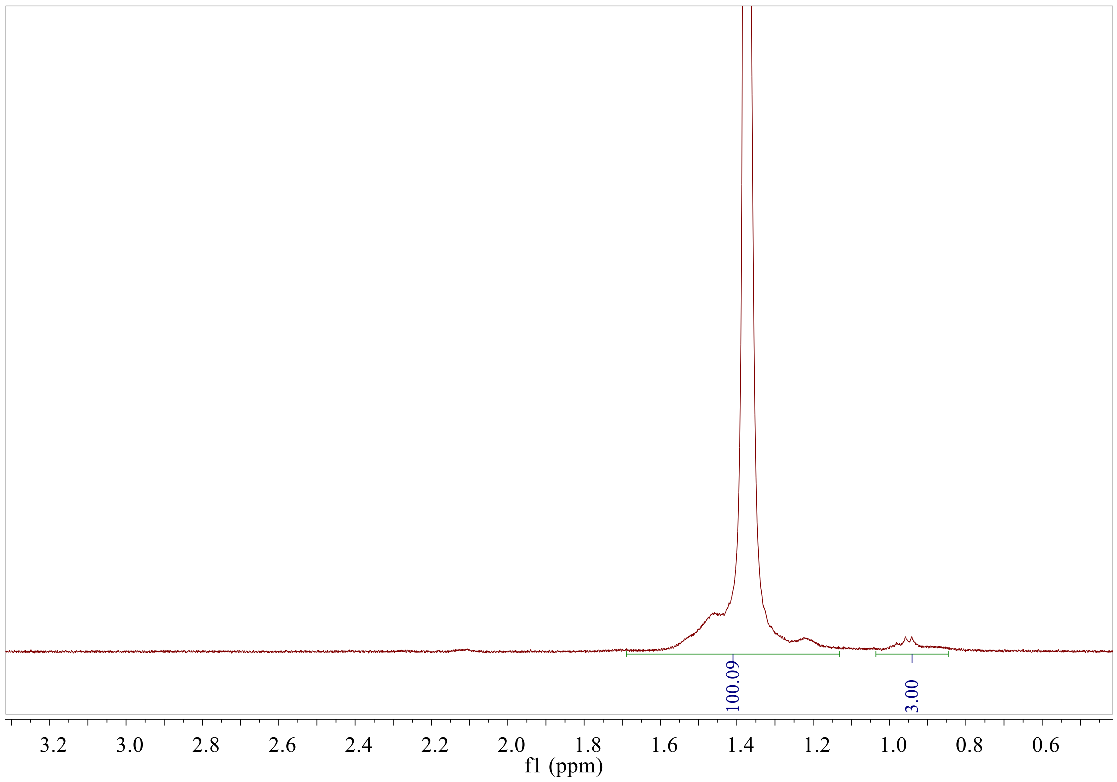

Supplementary Figure 71. ^{1}H NMR spectrum of the polymer from supplementary table 1, entry 2 ($C_2D_2Cl_4$, 120 °C).

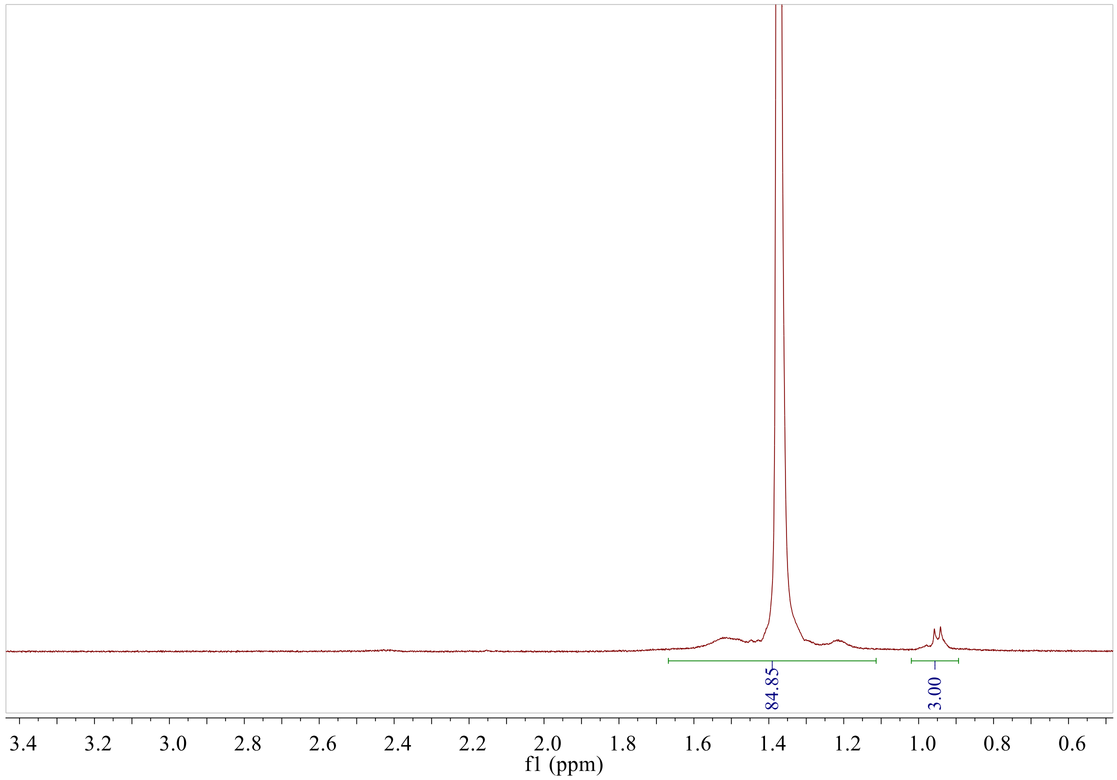

Supplementary Figure 72. ^{1}H NMR spectrum of the polymer from supplementary table 1, entry 3 ($C_2D_2Cl_4$, 120 °C).

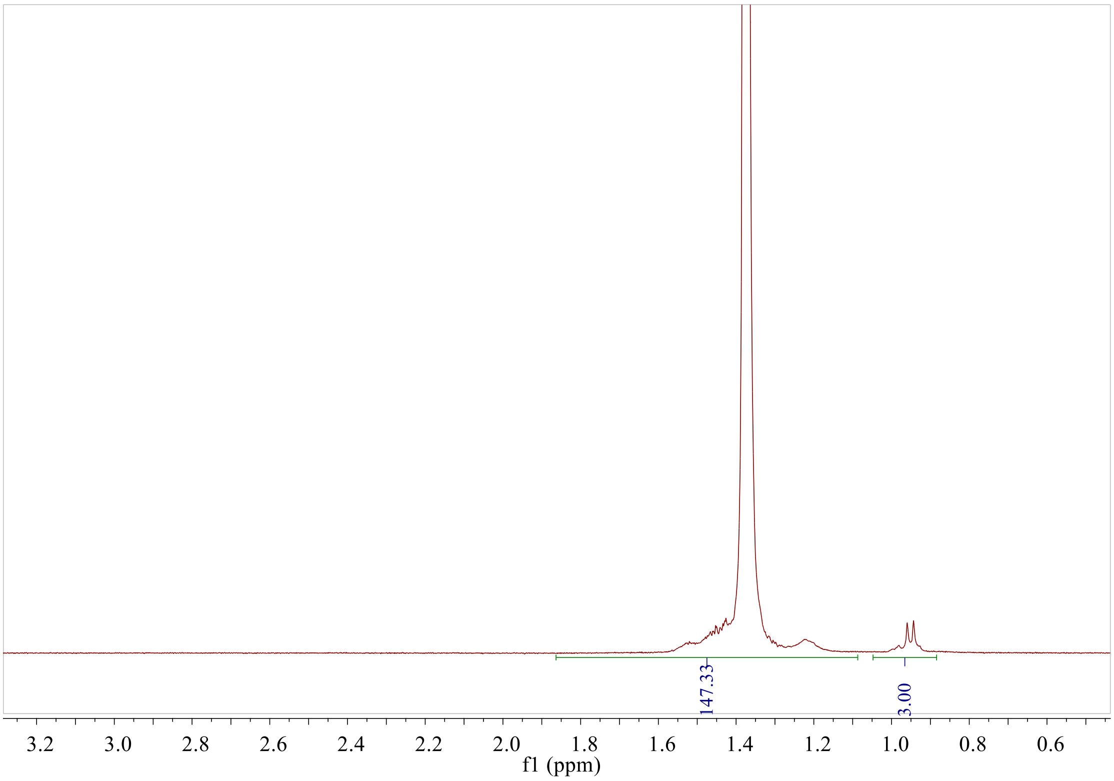

Supplementary Figure 73. ^{1}H NMR spectrum of the polymer from supplementary table 1, entry 4 ($C_2D_2Cl_4$, 120 °C).

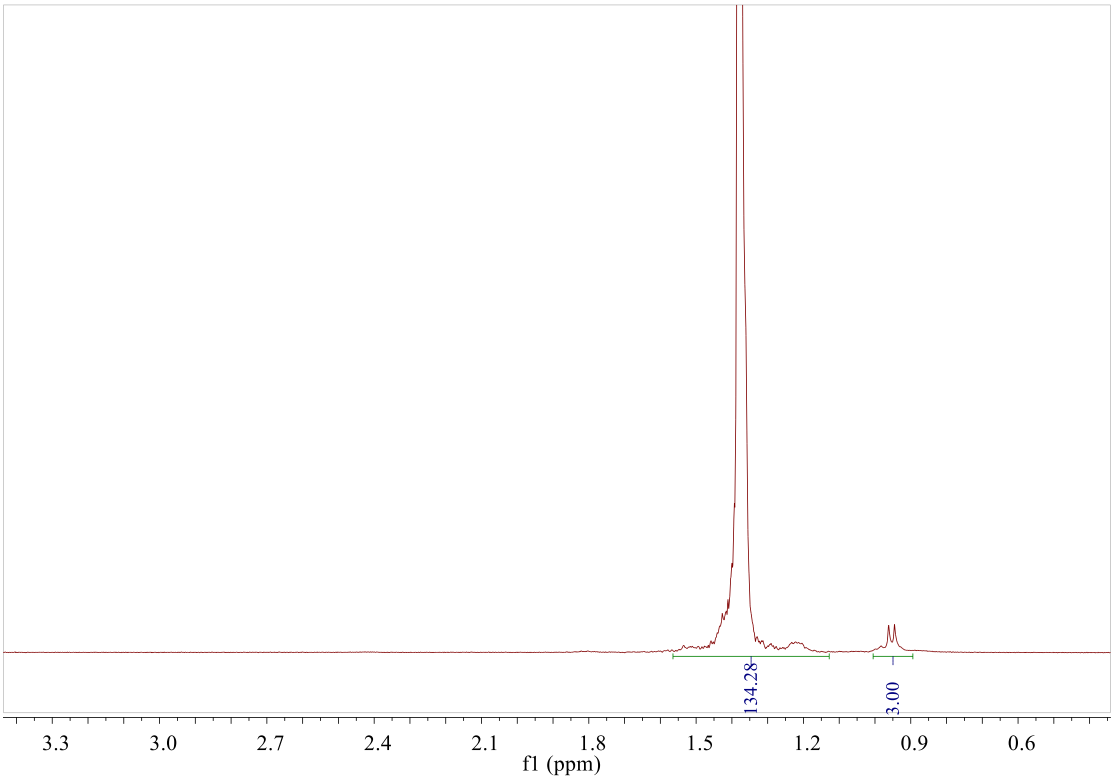

Supplementary Figure 74. ^{1}H NMR spectrum of the polymer from supplementary table 1, entry 5 ($C_2D_2Cl_4$, 120 °C).

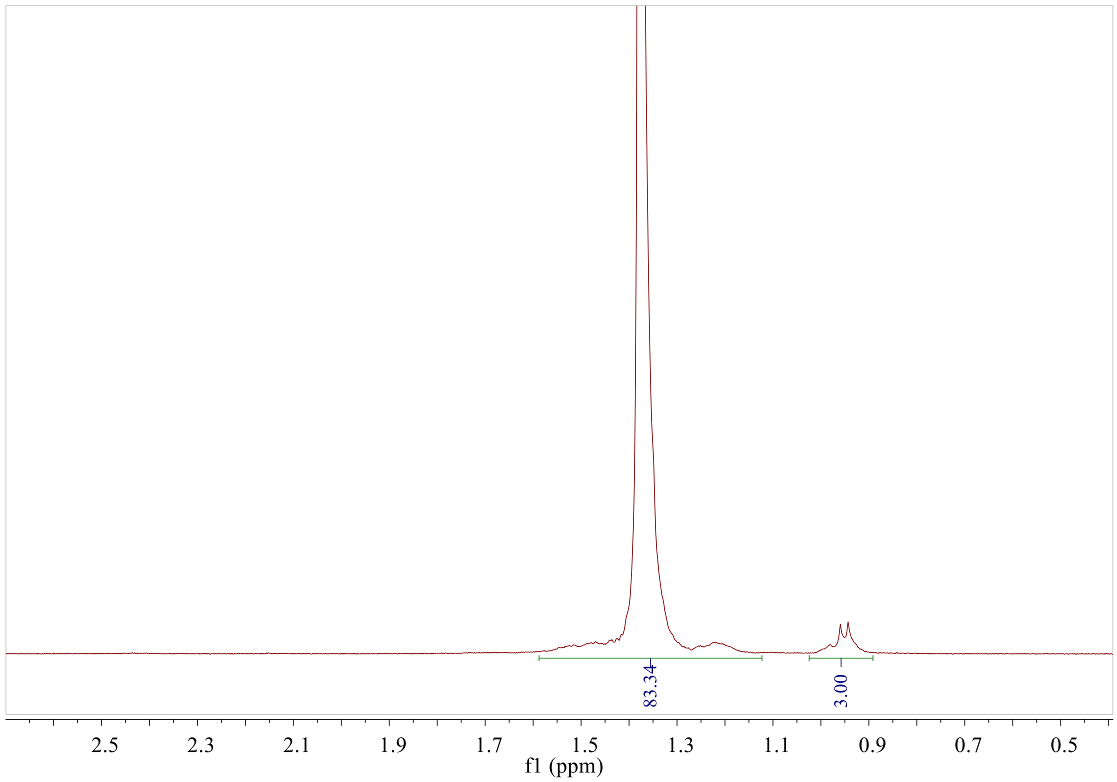

Supplementary Figure 75. ^{1}H NMR spectrum of the polymer from table supplementary table 1, entry 6 ($C_2D_2Cl_4$, 120 °C).

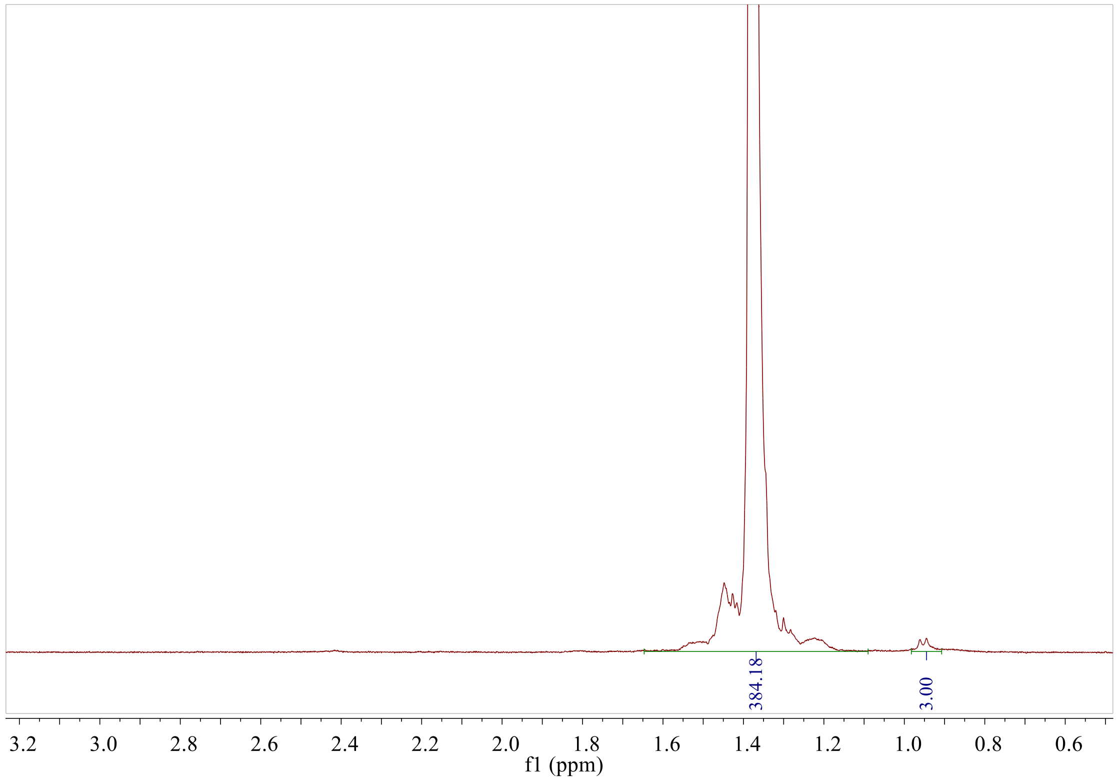

Supplementary Figure 76. ^{1}H NMR spectrum of the polymer from supplementary table 1, entry 7 ($C_2D_2Cl_4$, 120 °C).

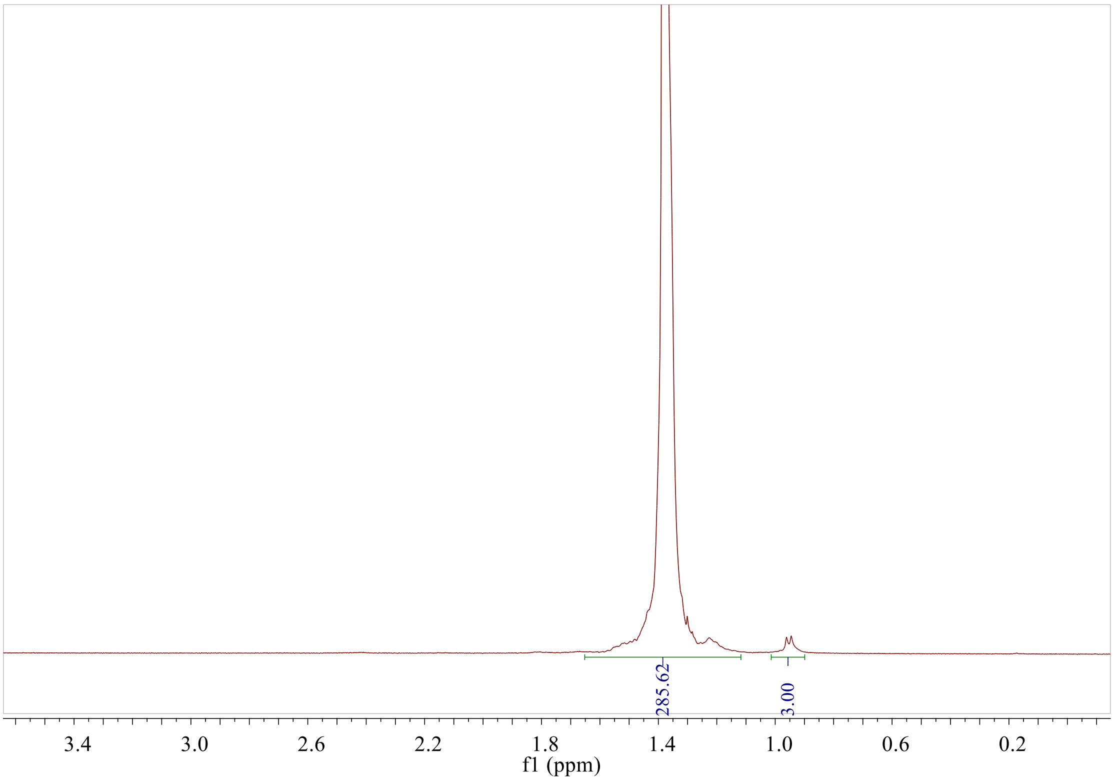

Supplementary Figure 77. ^{1}H NMR spectrum of the polymer from supplementary table 1, entry 8 ($C_2D_2Cl_4$, 120 °C).

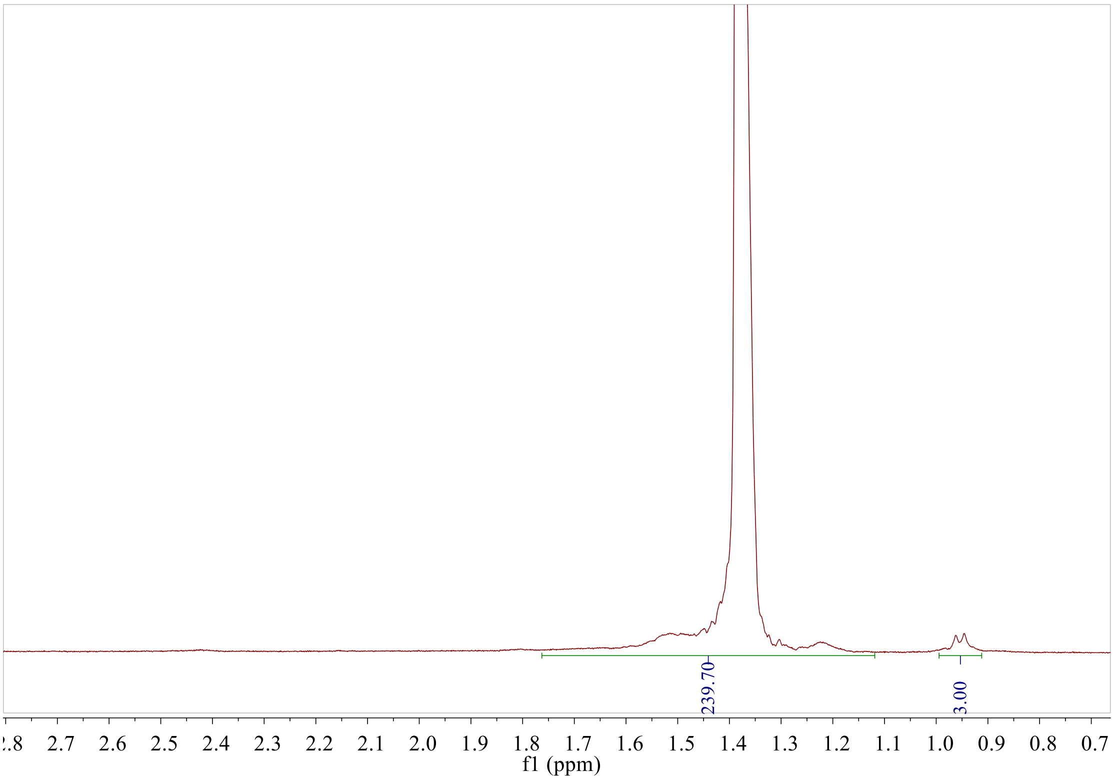

Supplementary Figure 78. ^{1}H NMR spectrum of the polymer from supplementary table 1, entry 9 ($C_2D_2Cl_4$, 120 °C).

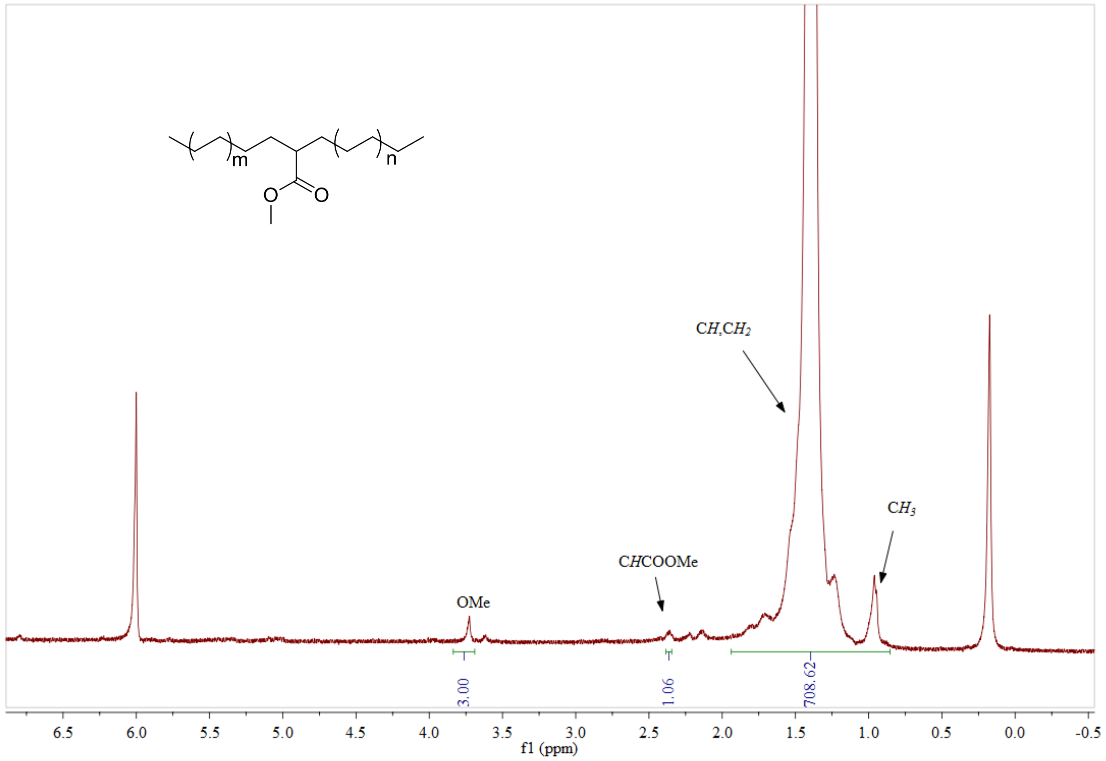

Supplementary Figure 79. ^{1}H NMR spectrum of the copolymer from table 2, entry 1 ($C_2D_2Cl_4$, 120 °C).

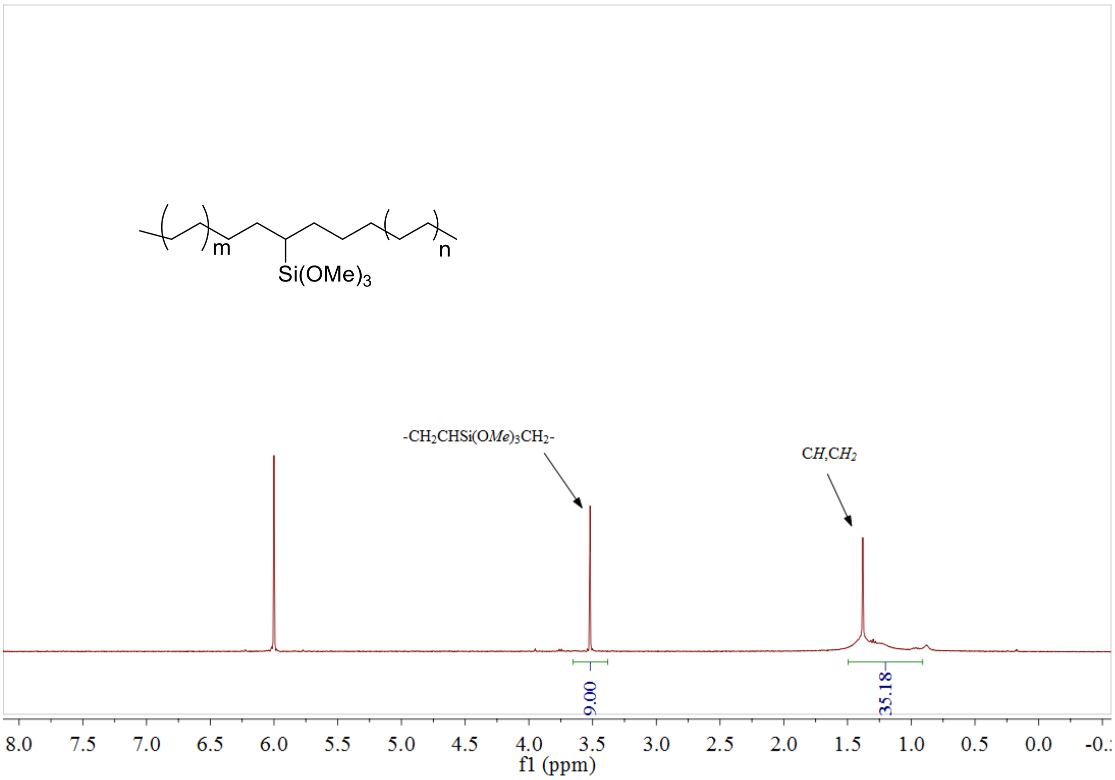

Supplementary Figure 80. ^{1}H NMR spectrum of the copolymer from table 2, entry 2 ($C_2D_2Cl_4$, 120 °C).

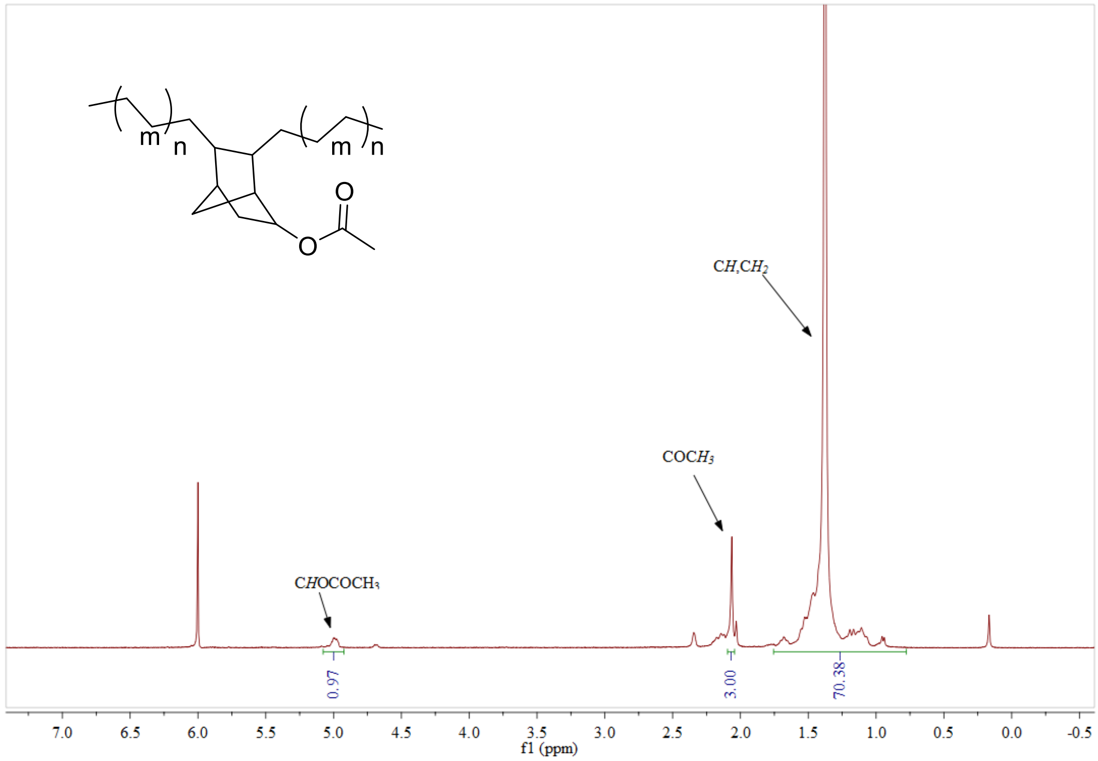

Supplementary Figure 81. ^{1}H NMR spectrum of the copolymer from table 2, entry 3 ($C_2D_2Cl_4$, 120°C).

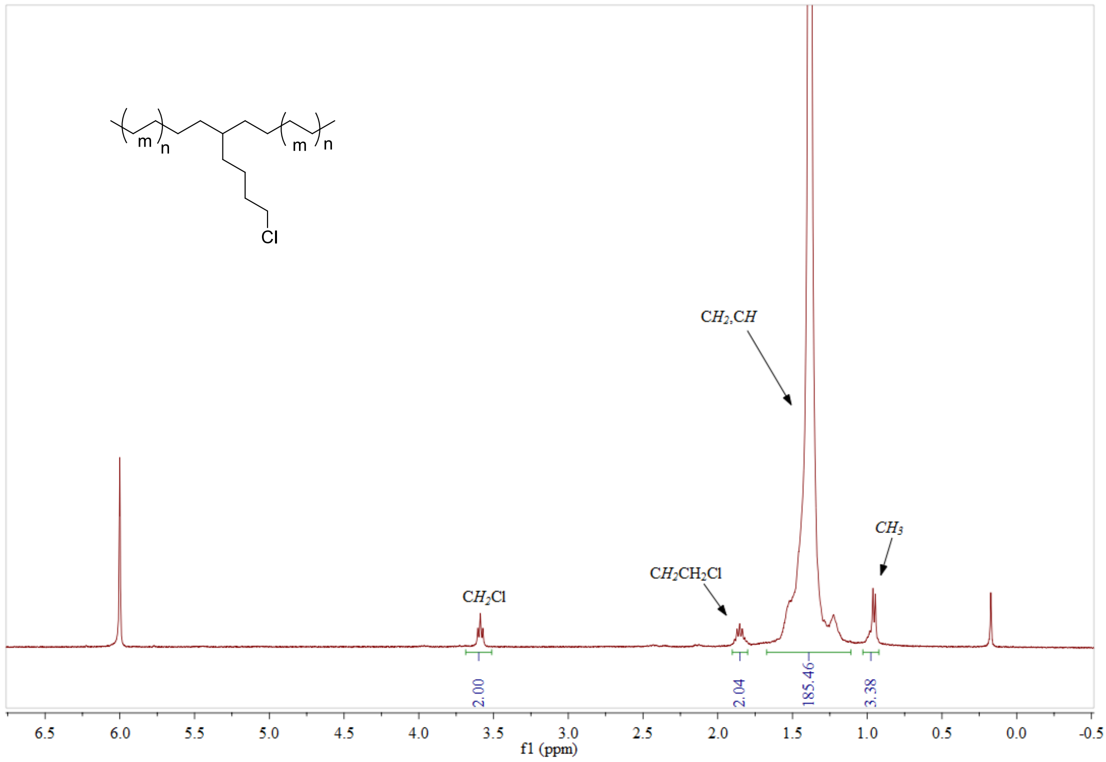

Supplementary Figure 82. ^{1}H NMR spectrum of the copolymer from table 2, entry 4

($C_2D_2Cl_4$, 120 °C).

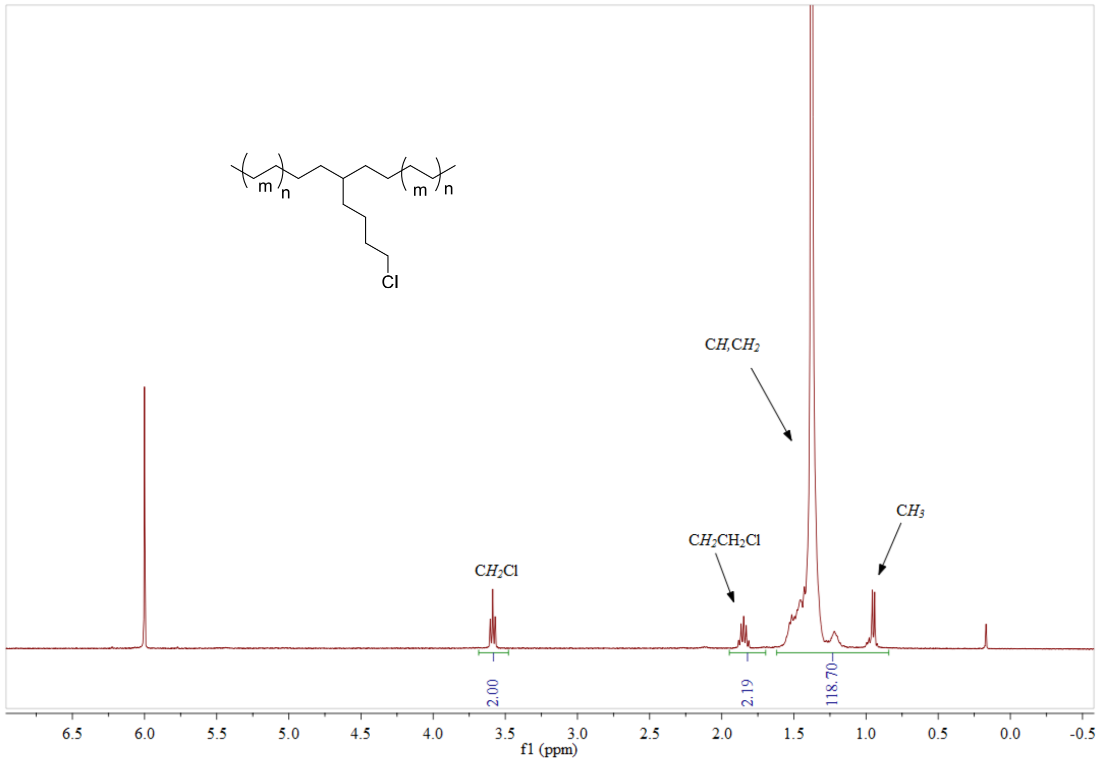

Supplementary Figure 83. ^{1}H NMR spectrum of the copolymer from table 2, entry 5 ($C_2D_2Cl_4$, 120 °C).

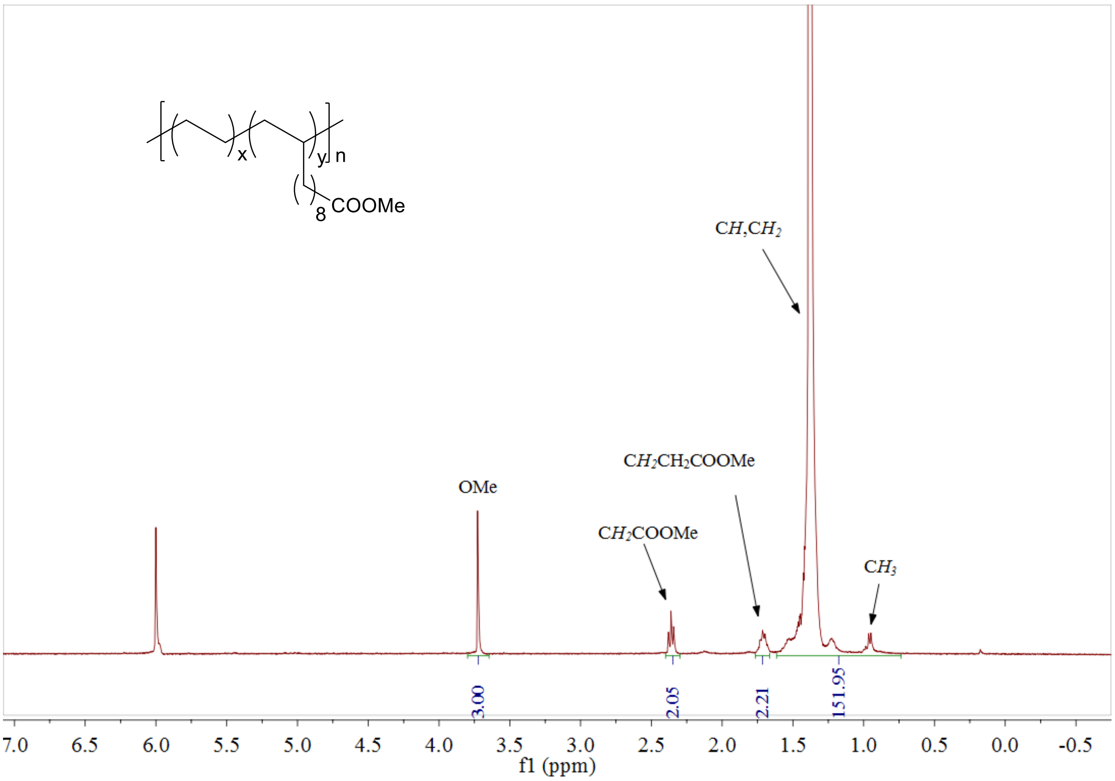

Supplementary Figure 84. ^{1}H NMR spectrum of the copolymer from table 2, entry 6 ($C_2D_2Cl_4$, 120 °C).

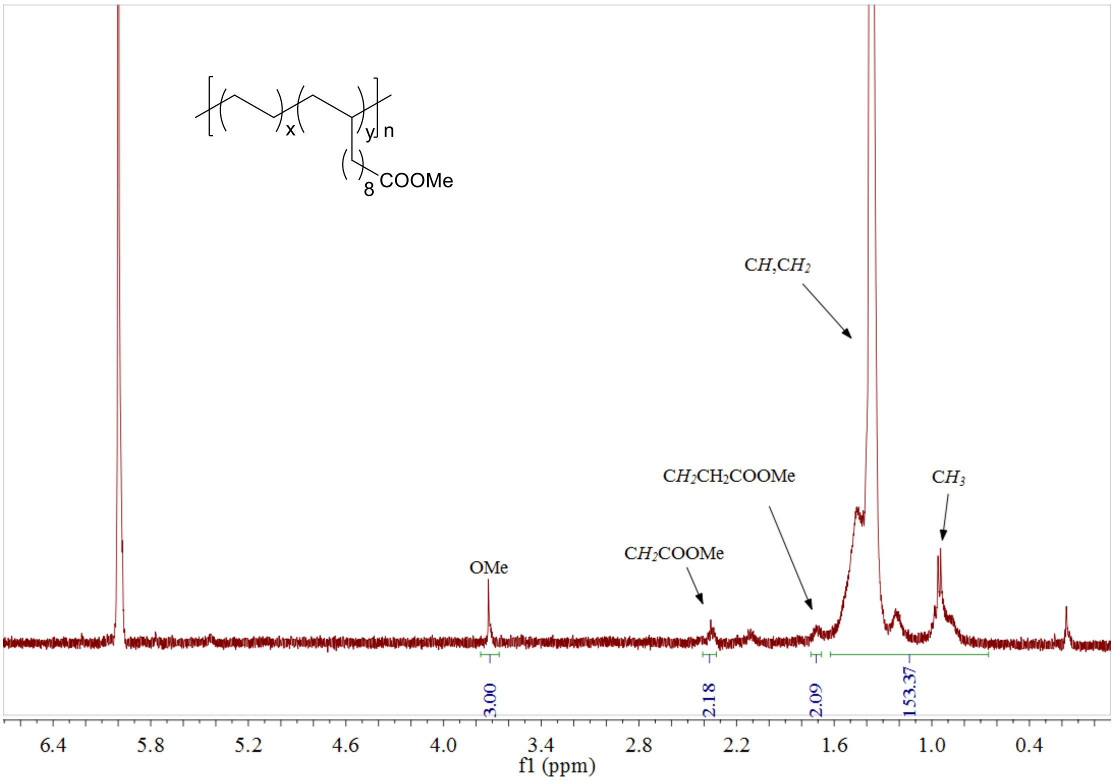

Supplementary Figure 85. ^{1}H NMR spectrum of the copolymer from table 2, entry 7 ($C_2D_2Cl_4$, 120 °C).

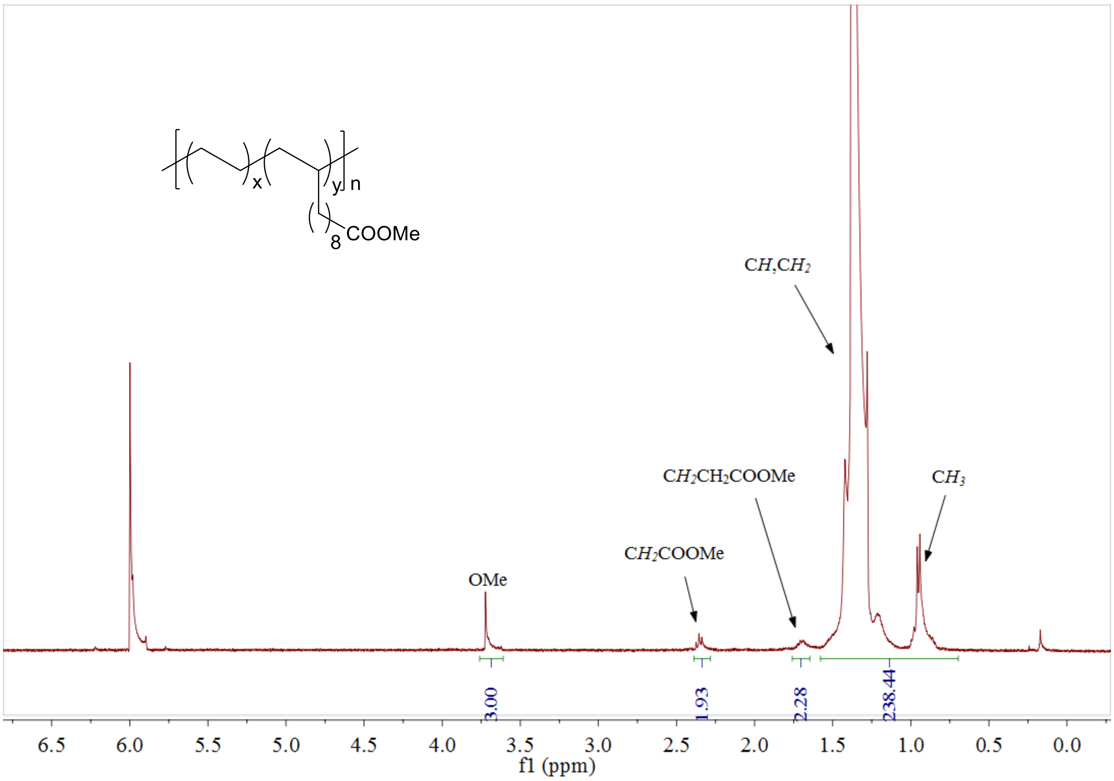

Supplementary Figure 86. ^{1}H NMR spectrum of the copolymer from table 2, entry 8 ($C_2D_2Cl_4$, 120 °C).

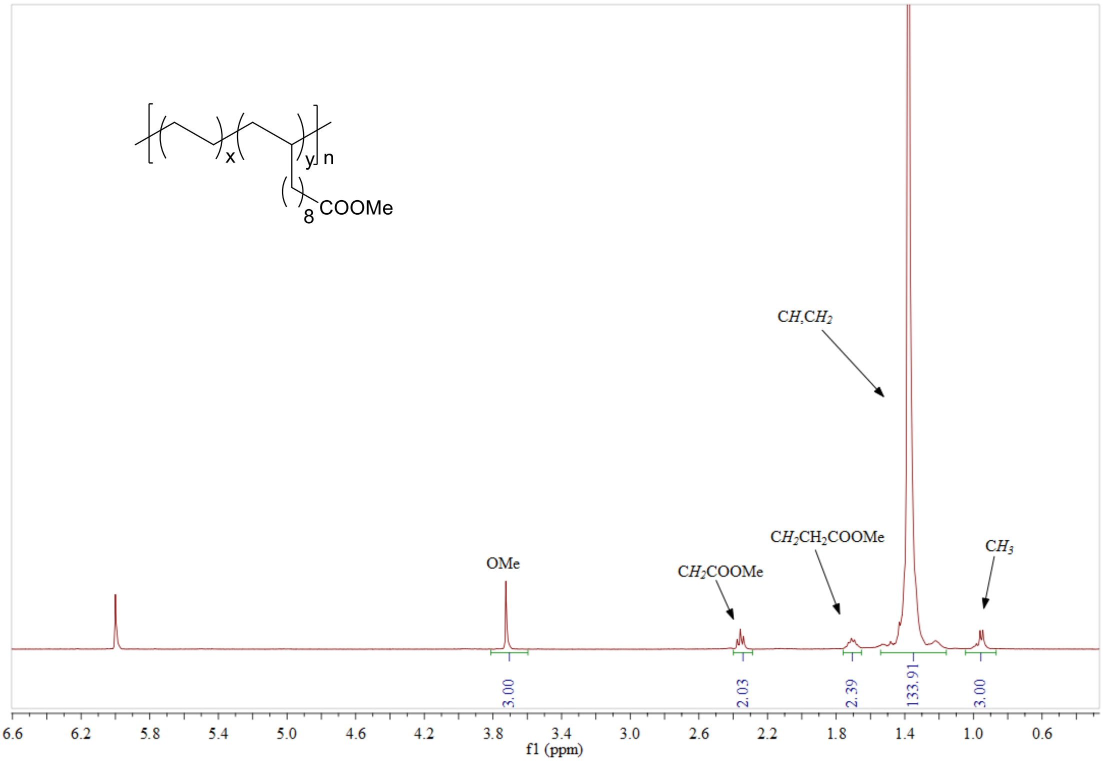

Supplementary Figure 87. ^{1}H NMR spectrum of the copolymer from table 2, entry 9 ($C_2D_2Cl_4$, 120 °C).

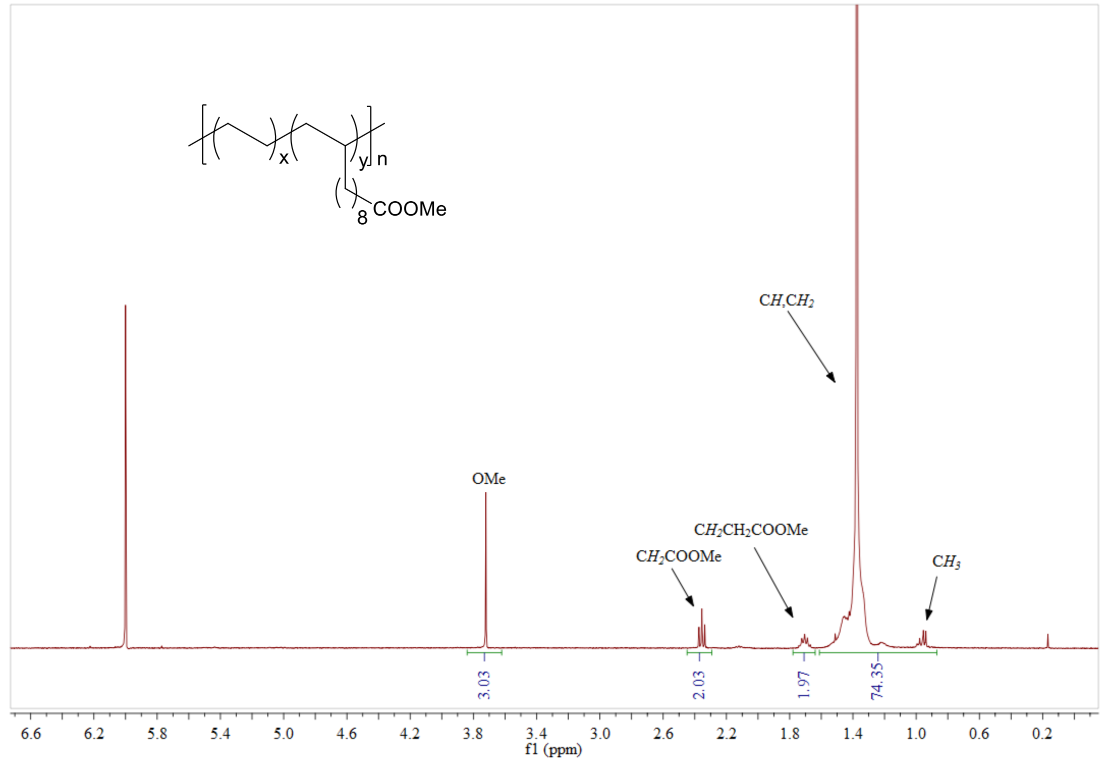

Supplementary Figure 88. ^{1}H NMR spectrum of the copolymer from table 2, entry 10 ($C_2D_2Cl_4$, 120 °C).

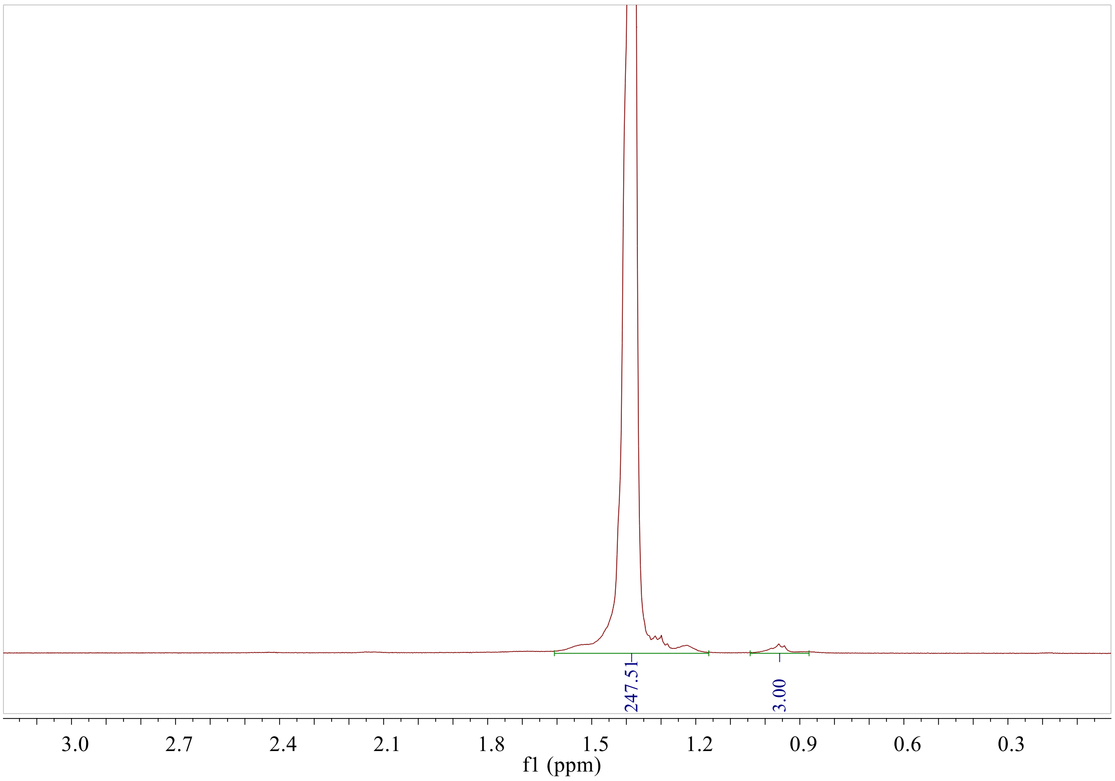

Supplementary Figure 89. ^{1}H NMR spectrum of the polymer from table 3, entry 1 ($C_2D_2Cl_4$, 120 °C).

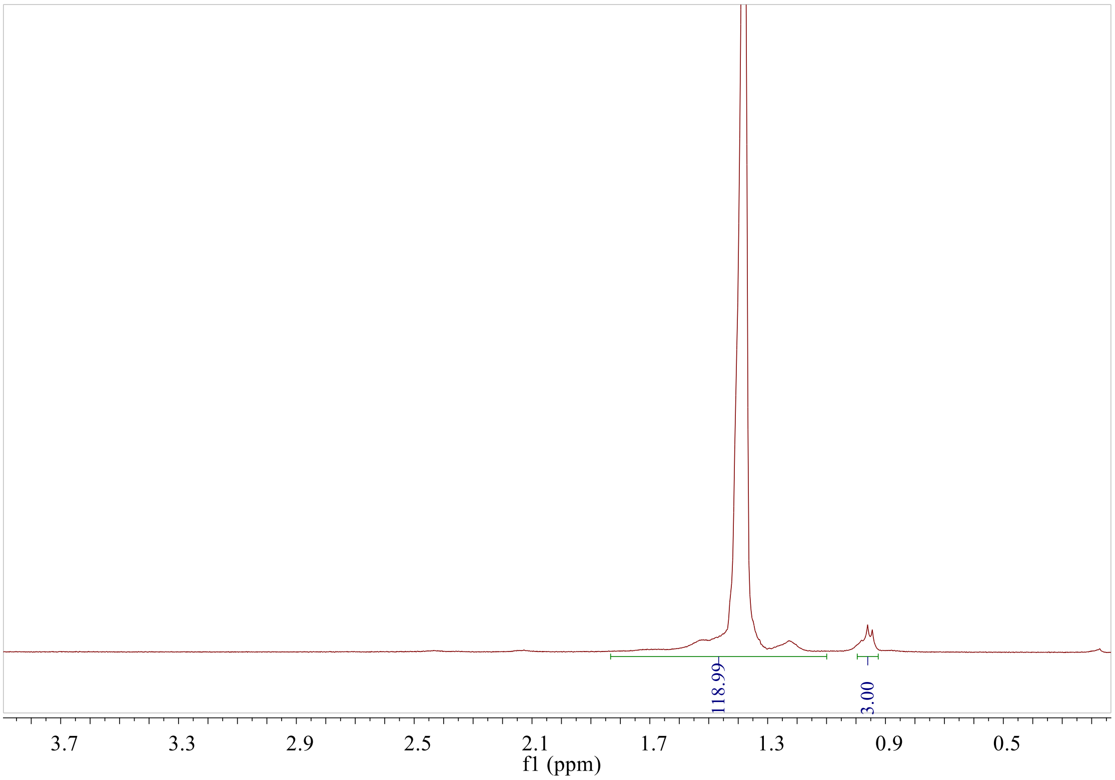

Supplementary Figure 90. ^{1}H NMR spectrum of the polymer from table 3, entry 2 ($C_2D_2Cl_4$, 120 °C).

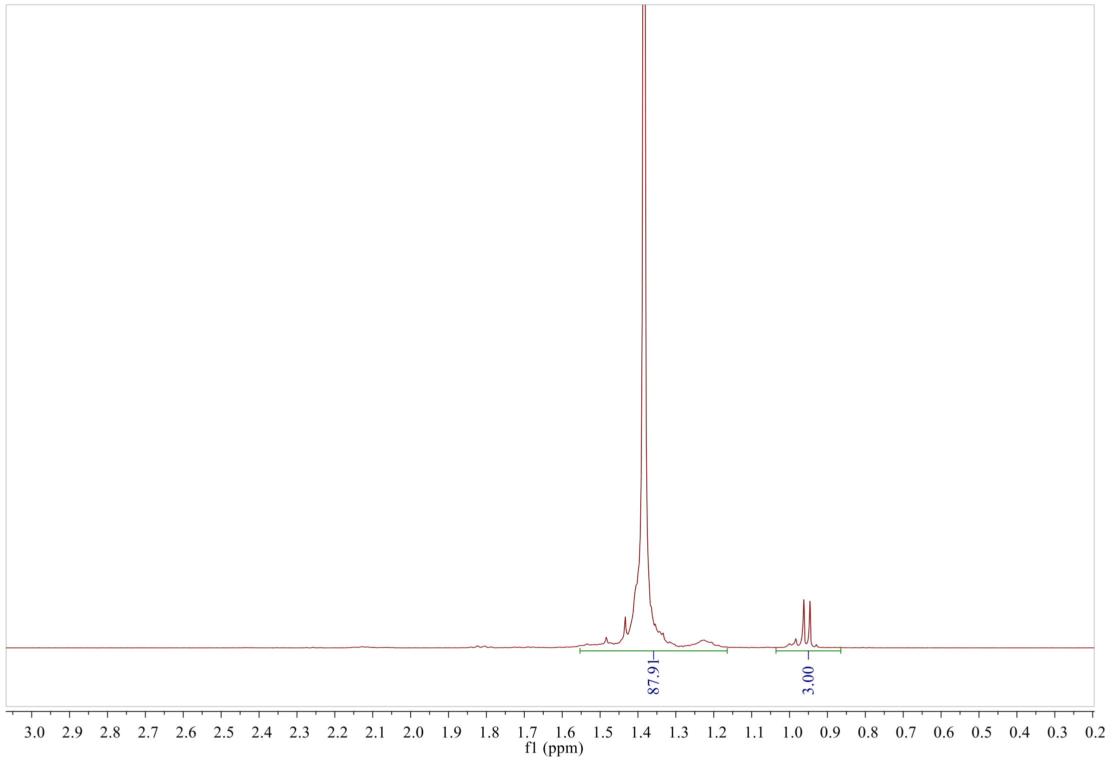

Supplementary Figure 91. ^{1}H NMR spectrum of the polymer from table 3, entry 3 ($C_2D_2Cl_4$, 120 °C).

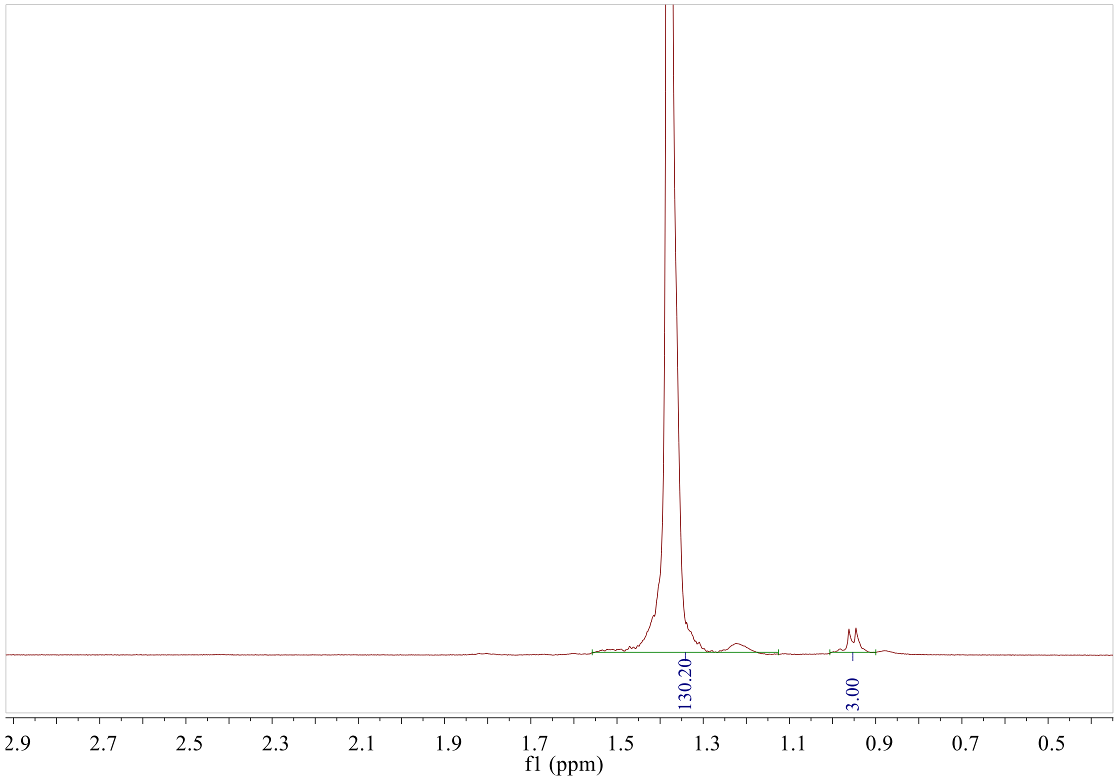

Supplementary Figure 92. ^{1}H NMR spectrum of the polymer from table 3, entry 4 ($C_2D_2Cl_4$, 120 °C).

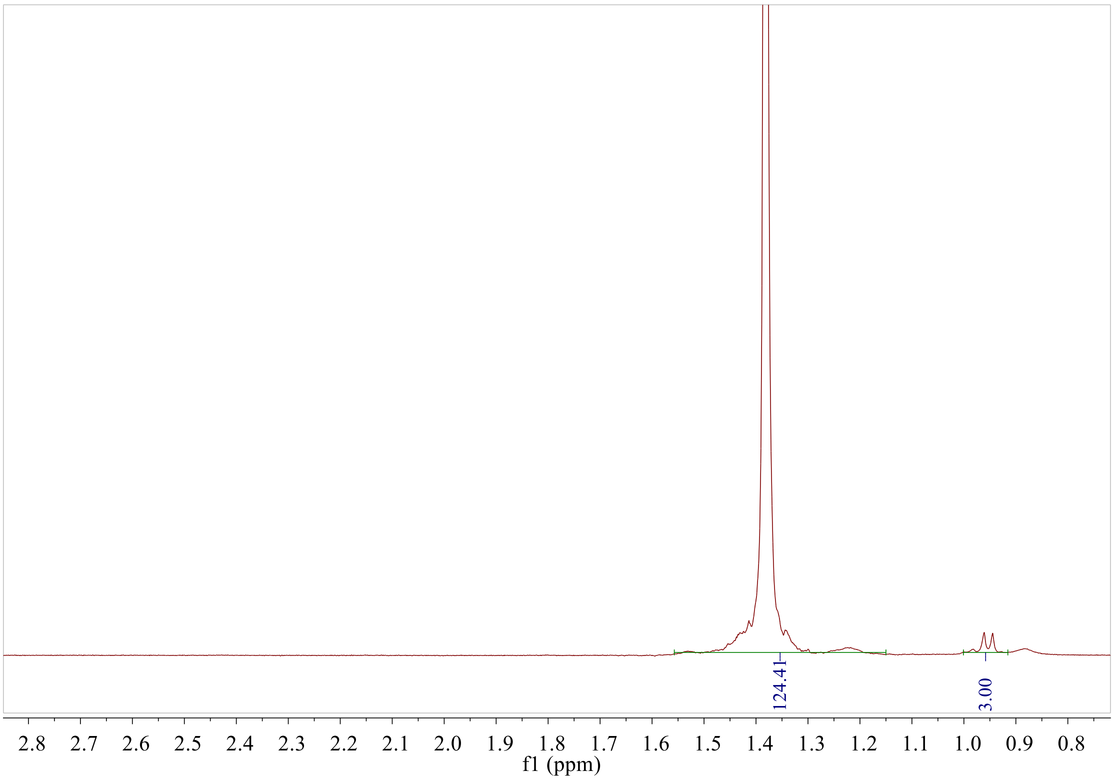

Supplementary Figure 93. ^{1}H NMR spectrum of the polymer from table 3, entry 5 ($C_2D_2Cl_4$, 120 °C).

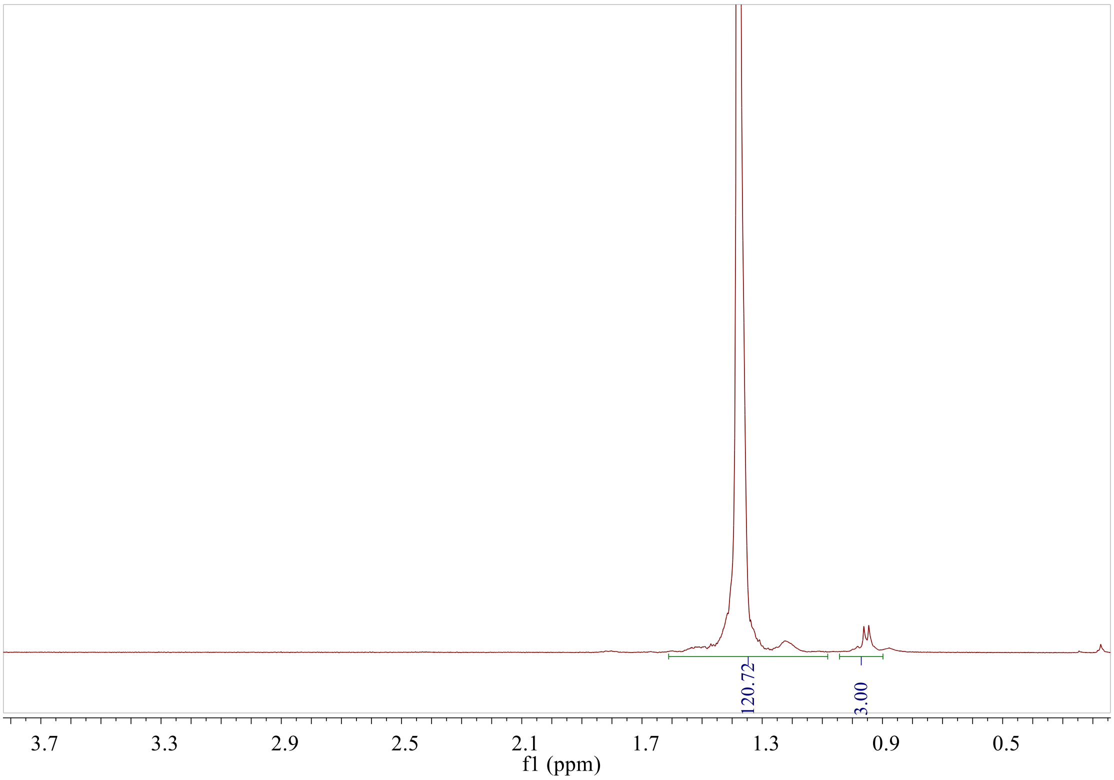

Supplementary Figure 94. ^{1}H NMR spectrum of the polymer from table 3, entry 6 ($C_2D_2Cl_4$, 120 °C).

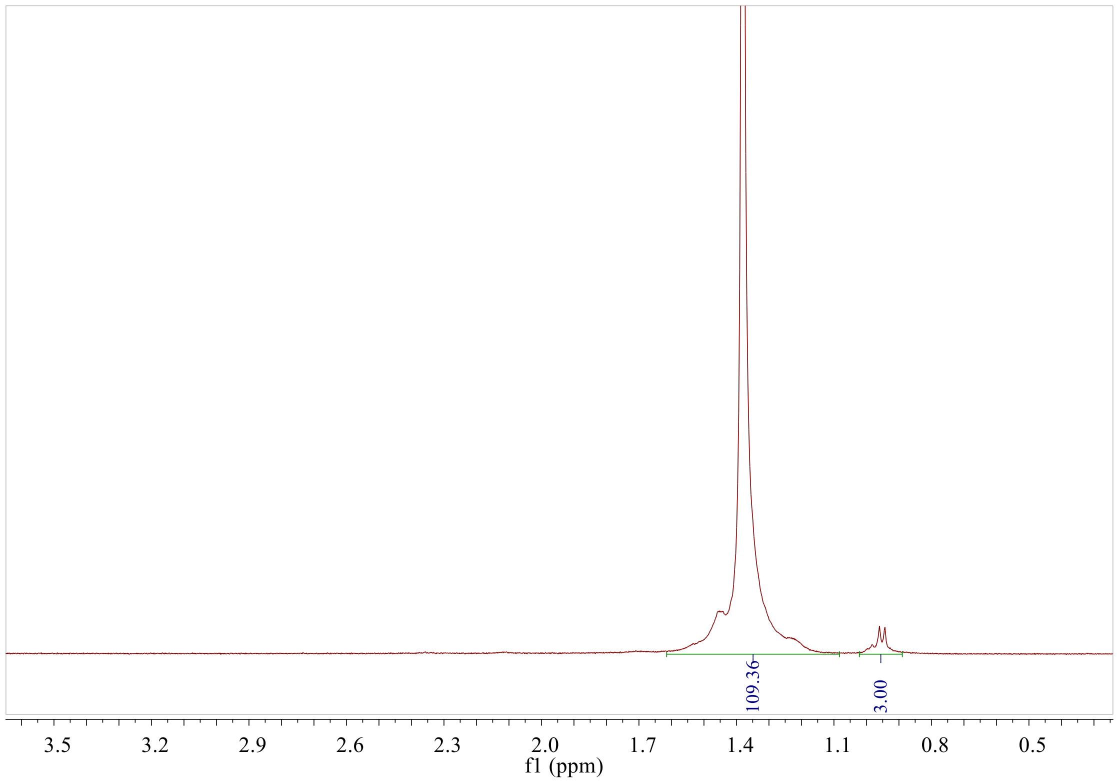

Supplementary Figure 95. ^{1}H NMR spectrum of the polymer from table 3, entry 7 ($C_2D_2Cl_4$, 120 °C).

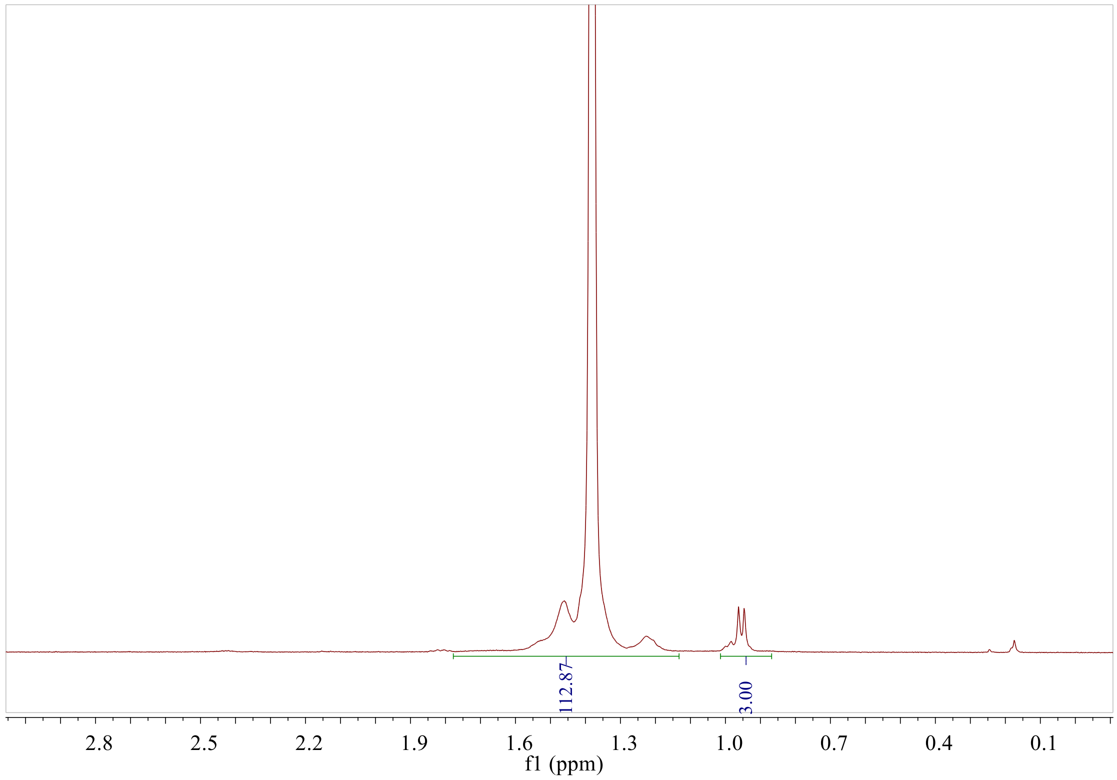

Supplementary Figure 96. ^{1}H NMR spectrum of the polymer from table 3, entry 8 ($C_2D_2Cl_4$, 120 °C).

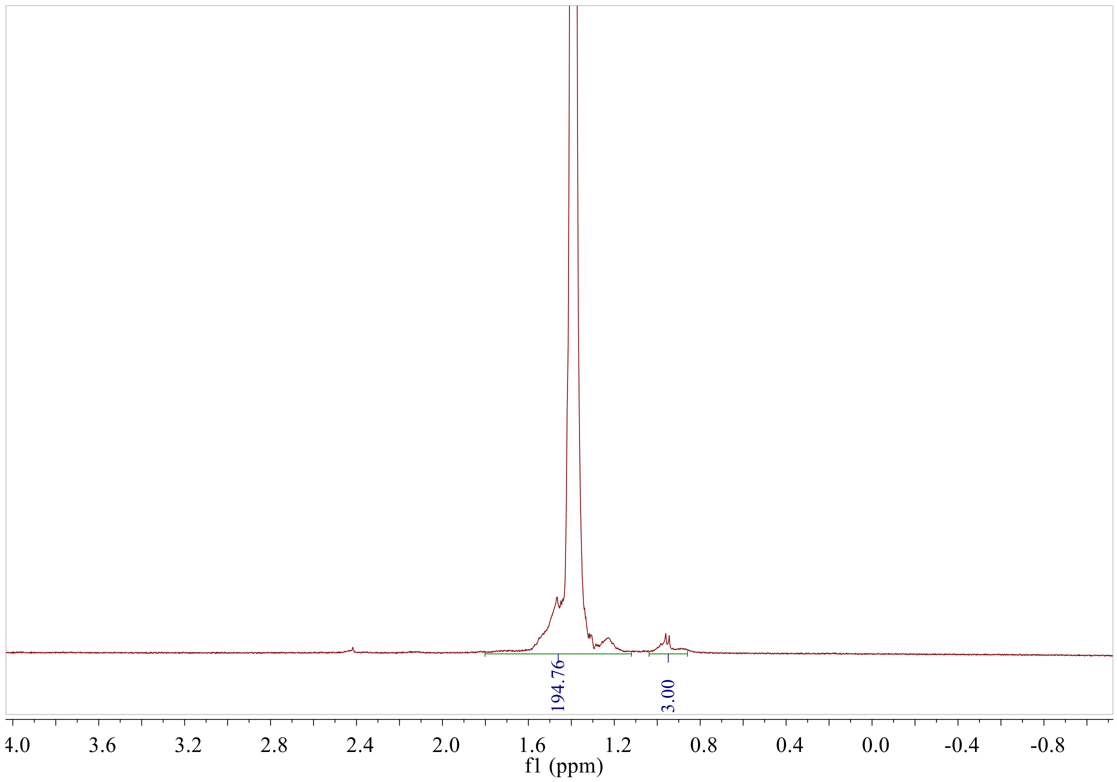

Supplementary Figure 97. ^{1}H NMR spectrum of the polymer from table 3, entry 9 ($C_2D_2Cl_4$, 120 °C).

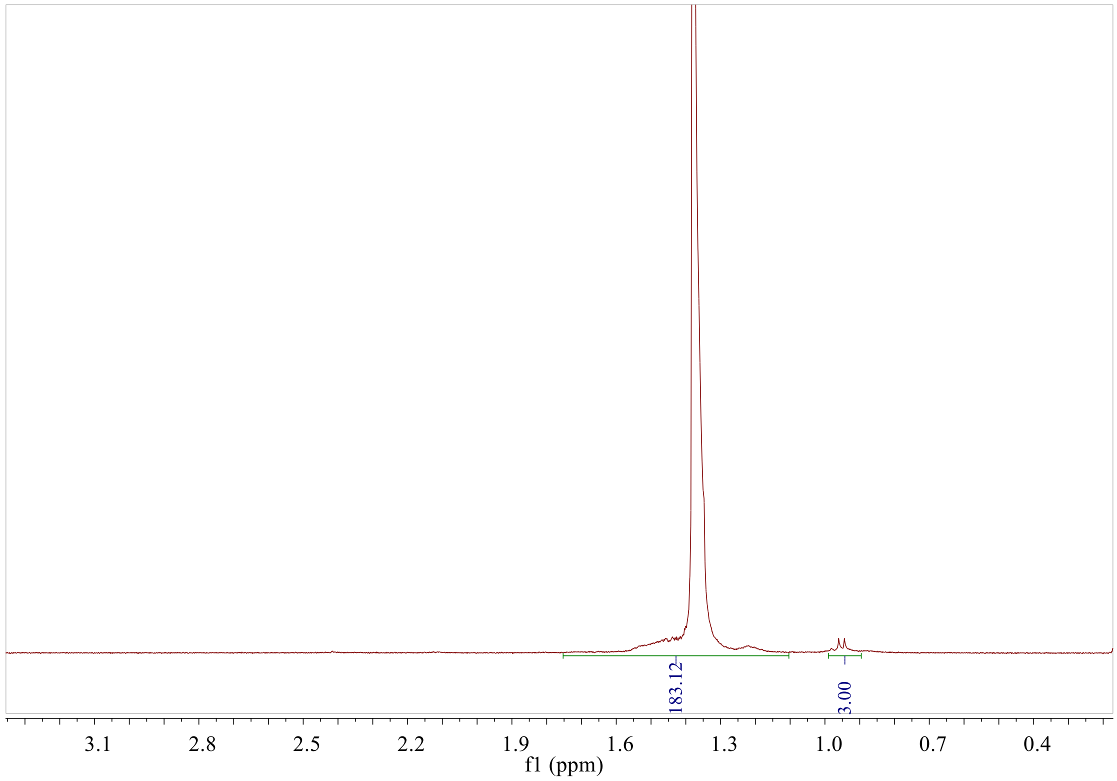

Supplementary Figure 98. ^{1}H NMR spectrum of the polymer from table 3, entry 10 ($C_2D_2Cl_4$, 120 °C).

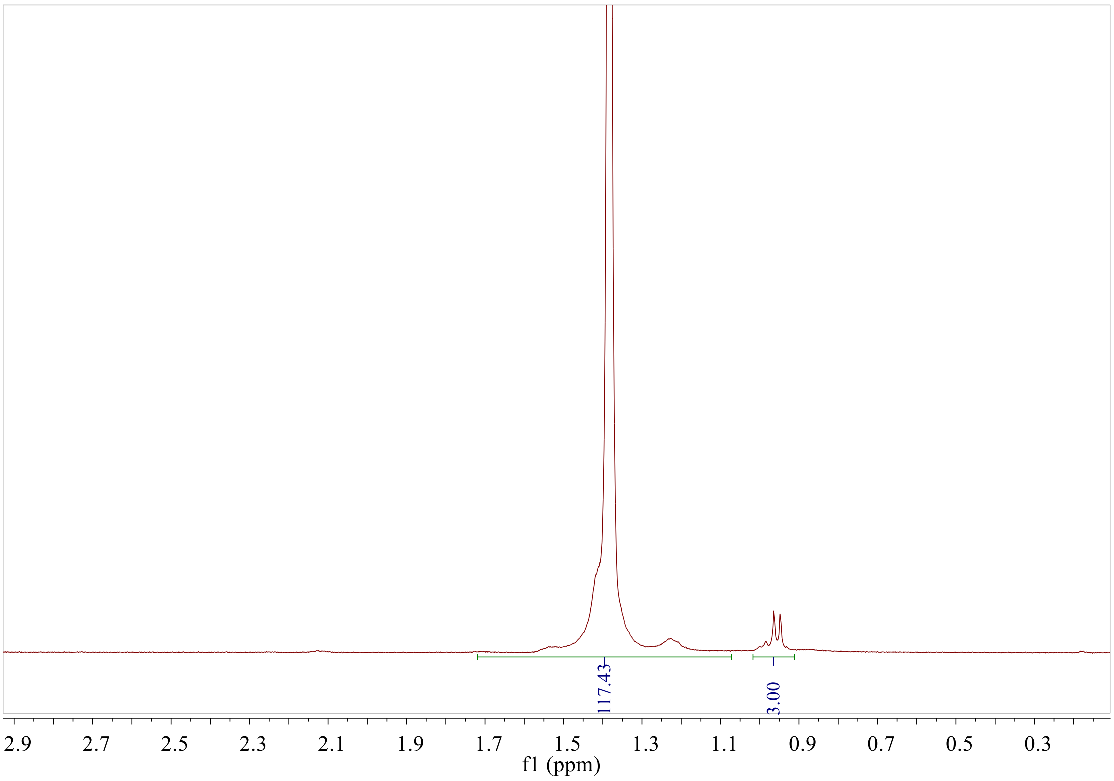

Supplementary Figure 99. ^{1}H NMR spectrum of the polymer from table 3, entry 11 ($C_2D_2Cl_4$, 120 °C).

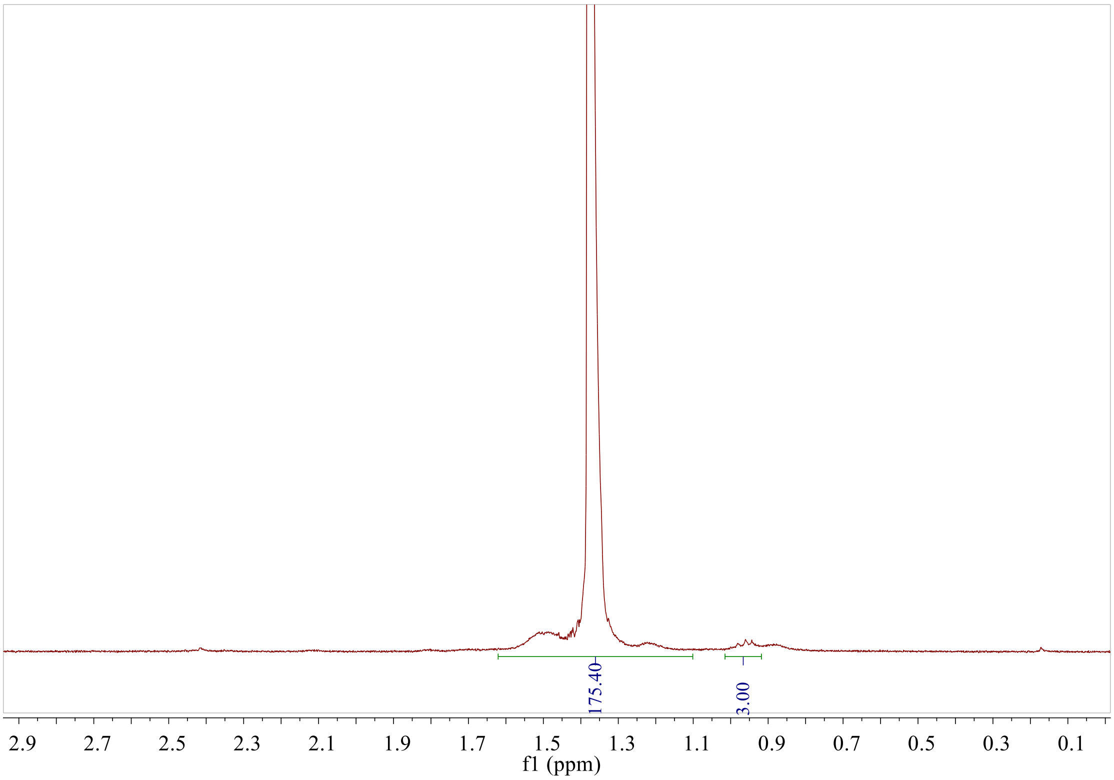

Supplementary Figure 100. ^{1}H NMR spectrum of the polymer from table 3, entry 12 ($C_2D_2Cl_4$, 120 °C).

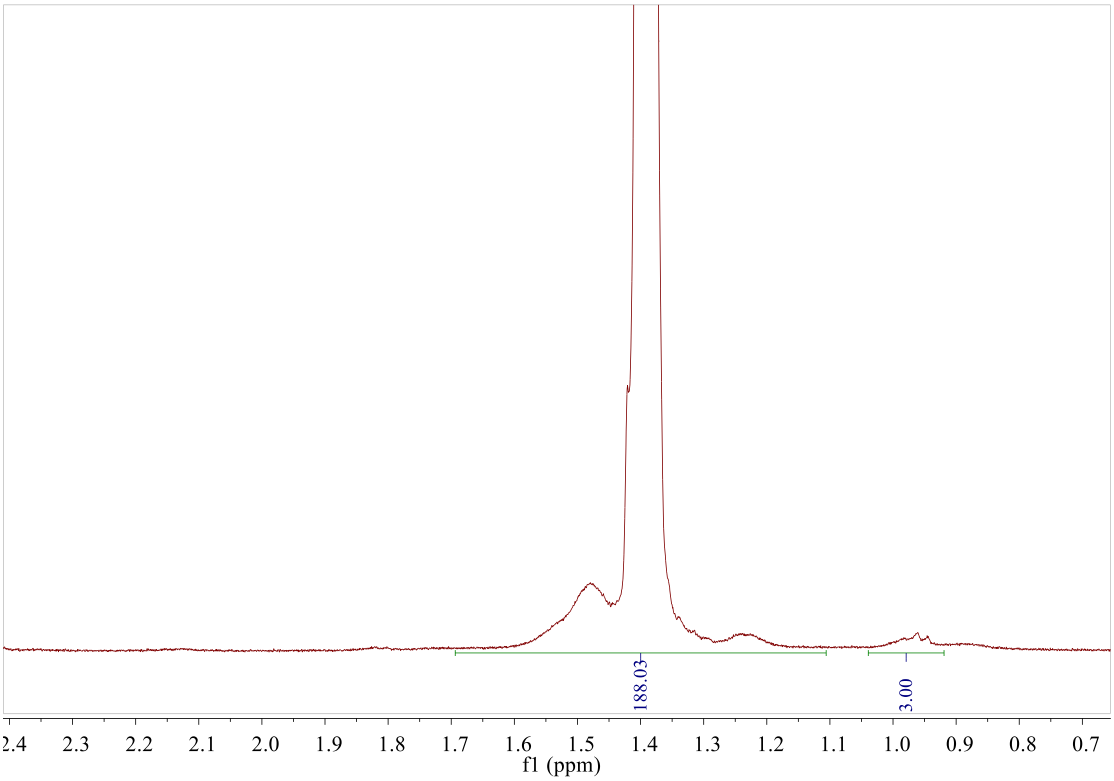

Supplementary Figure 101. ^{1}H NMR spectrum of the polymer from table 3, entry 13 ($C_2D_2Cl_4$, 120 °C).

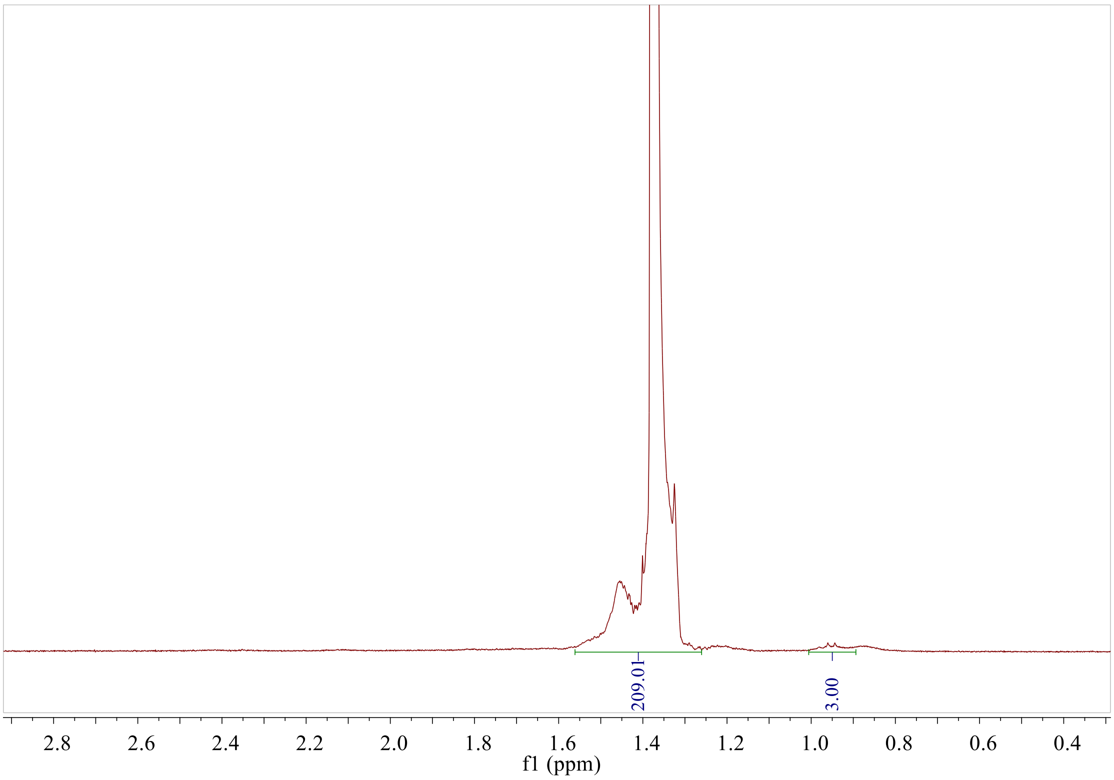

Supplementary Figure 102. ^{1}H NMR spectrum of the polymer from table 3, entry 14 ($C_2D_2Cl_4$, 120 °C).

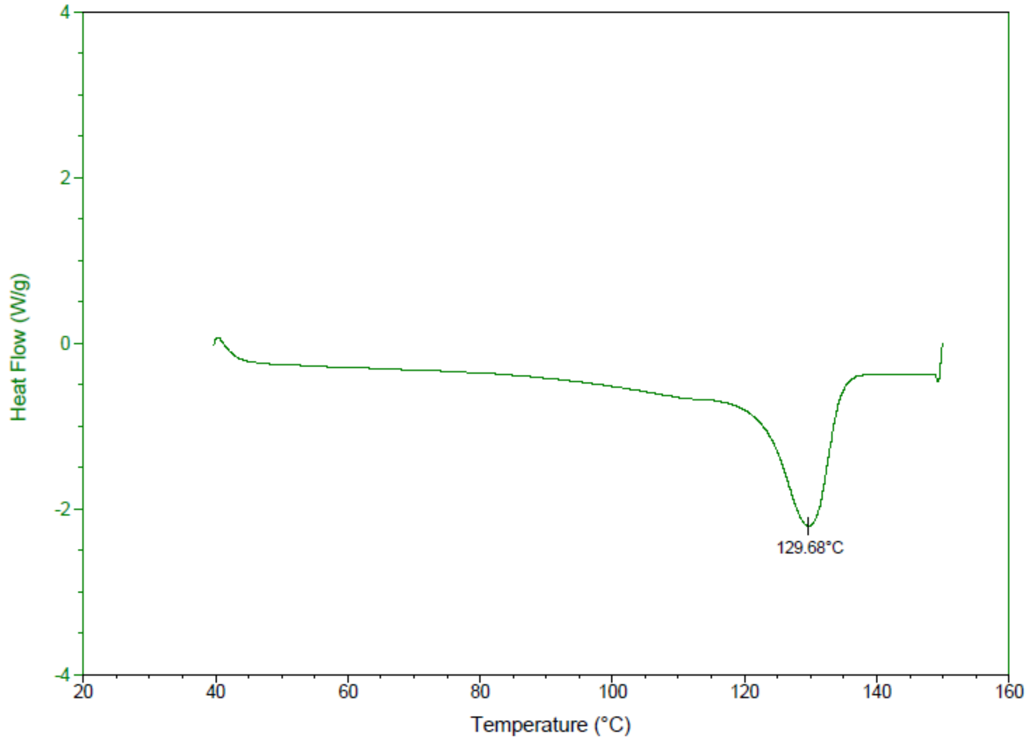

Supplementary Figure 103. DSC of the polymer from table 1, entry 1.

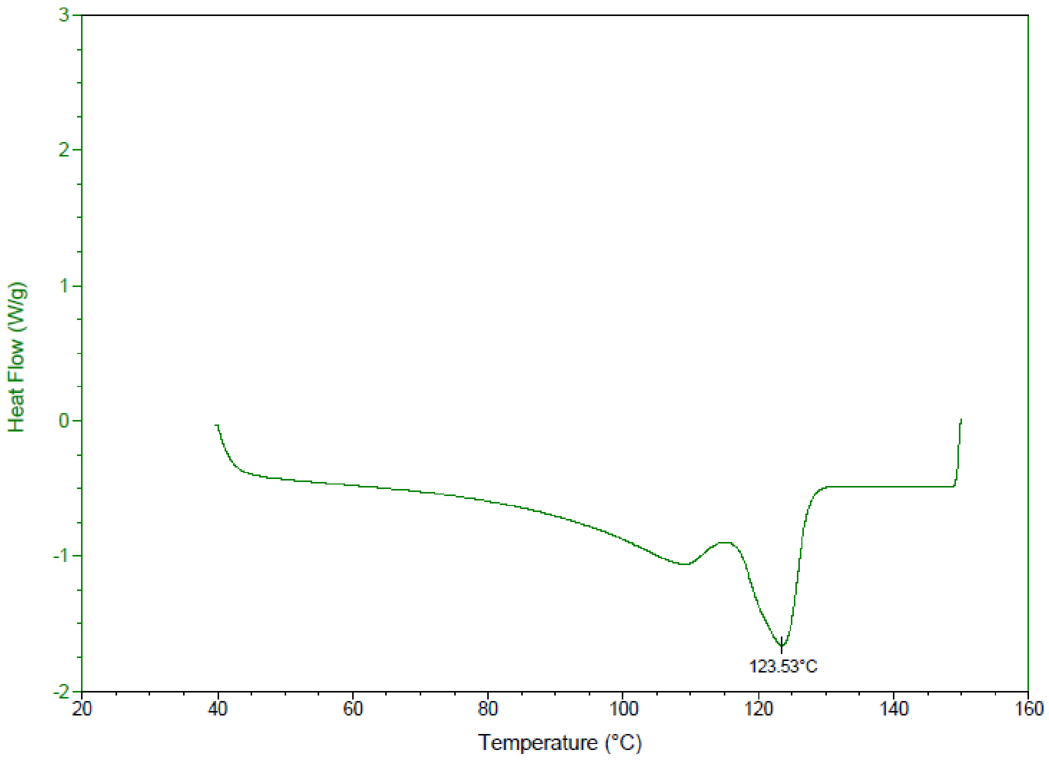

Supplementary Figure 104. DSC of the polymer from table 1, entry 2.

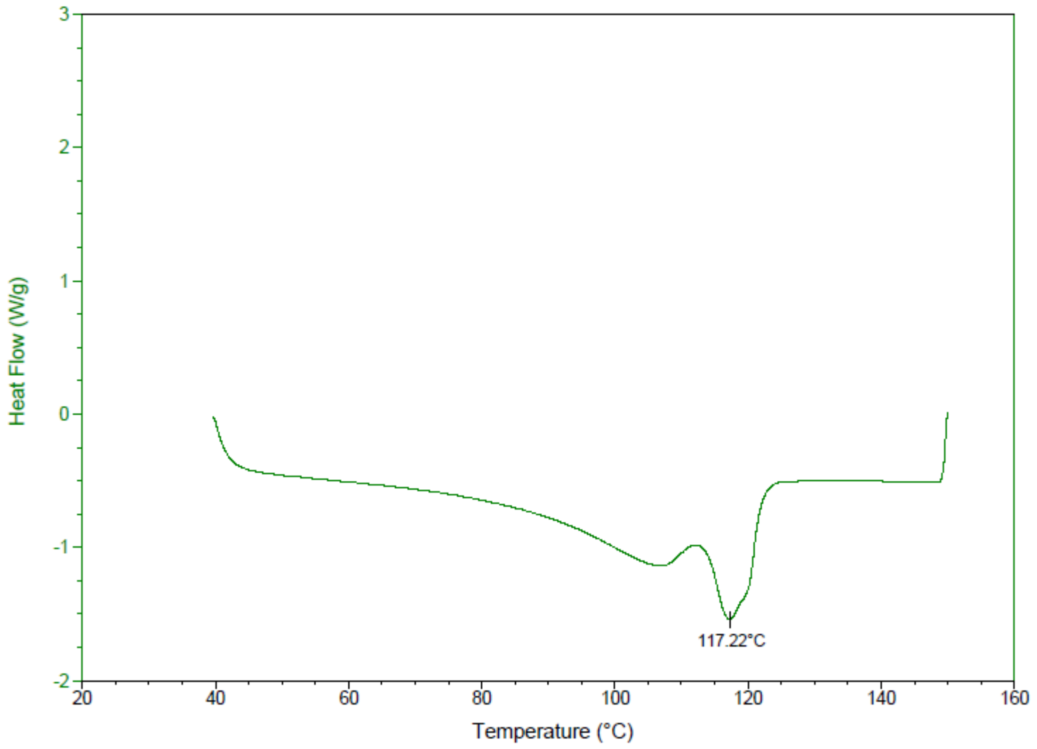

Supplementary Figure 105. DSC of the polymer from table 1, entry 3.

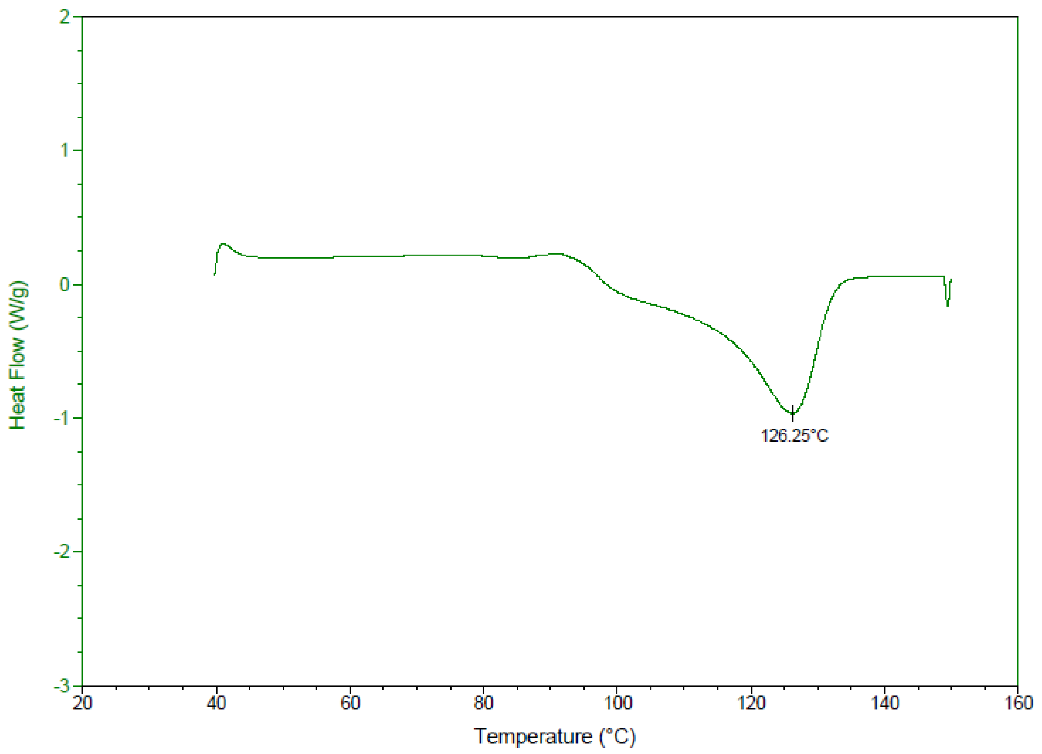

Supplementary Figure 106. DSC of the polymer from table 1, entry 4.

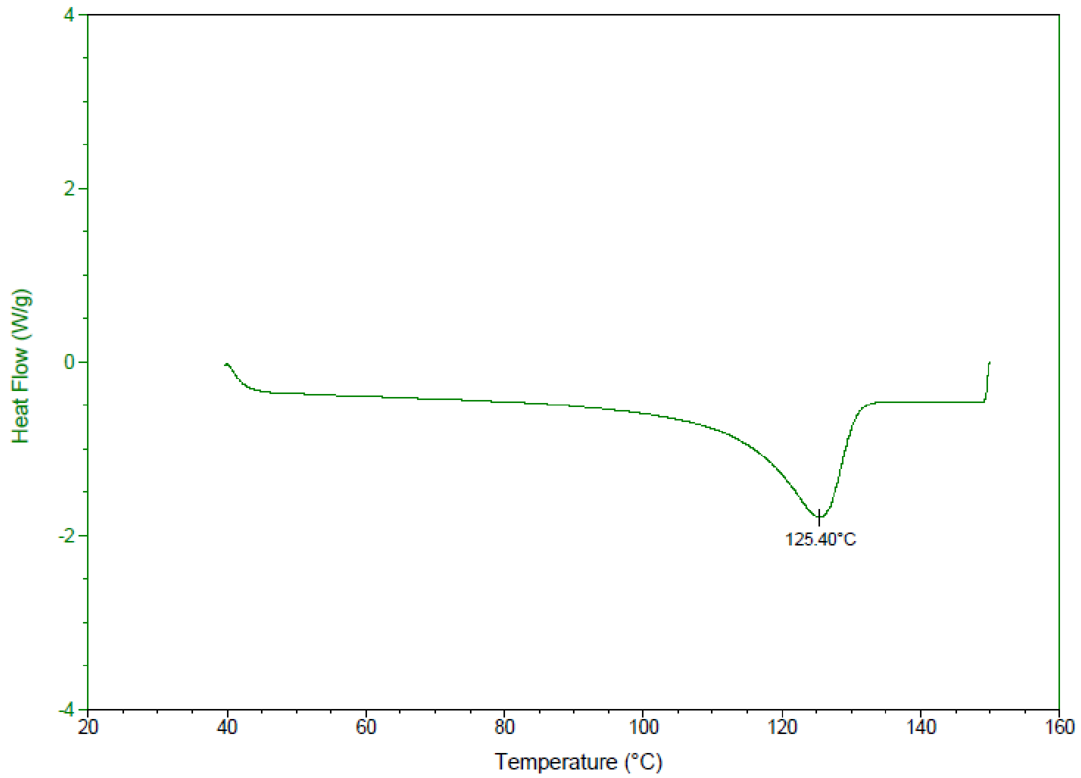

Supplementary Figure 107. DSC of the polymer from table 1, entry 5.

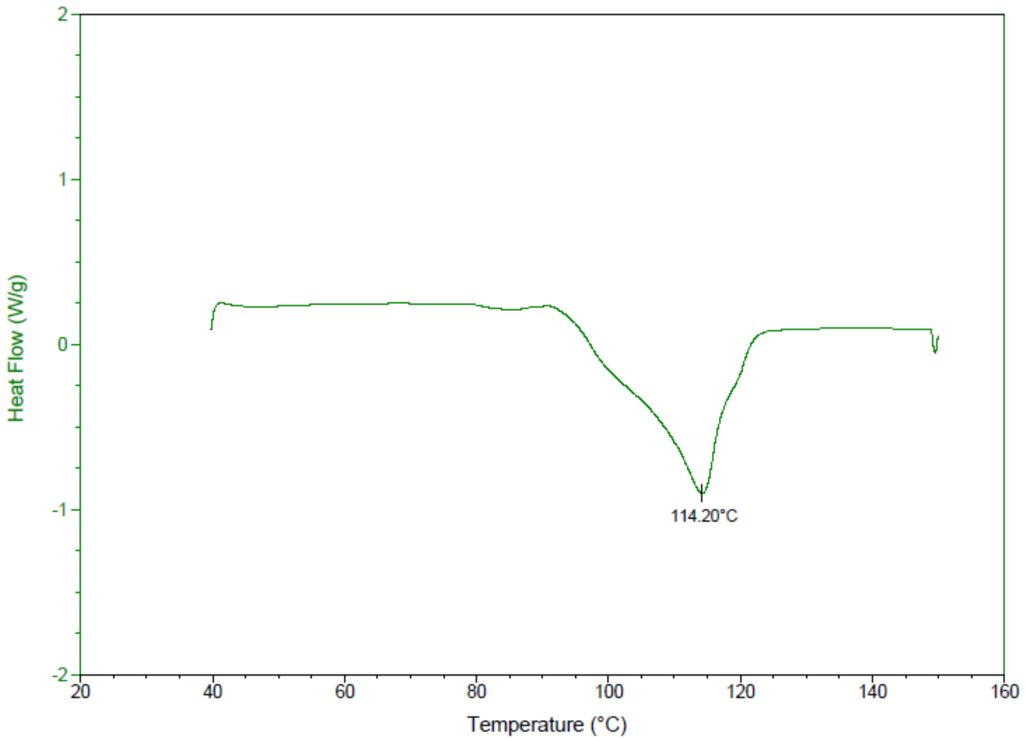

Supplementary Figure 108. DSC of the polymer from table 1, entry 6.

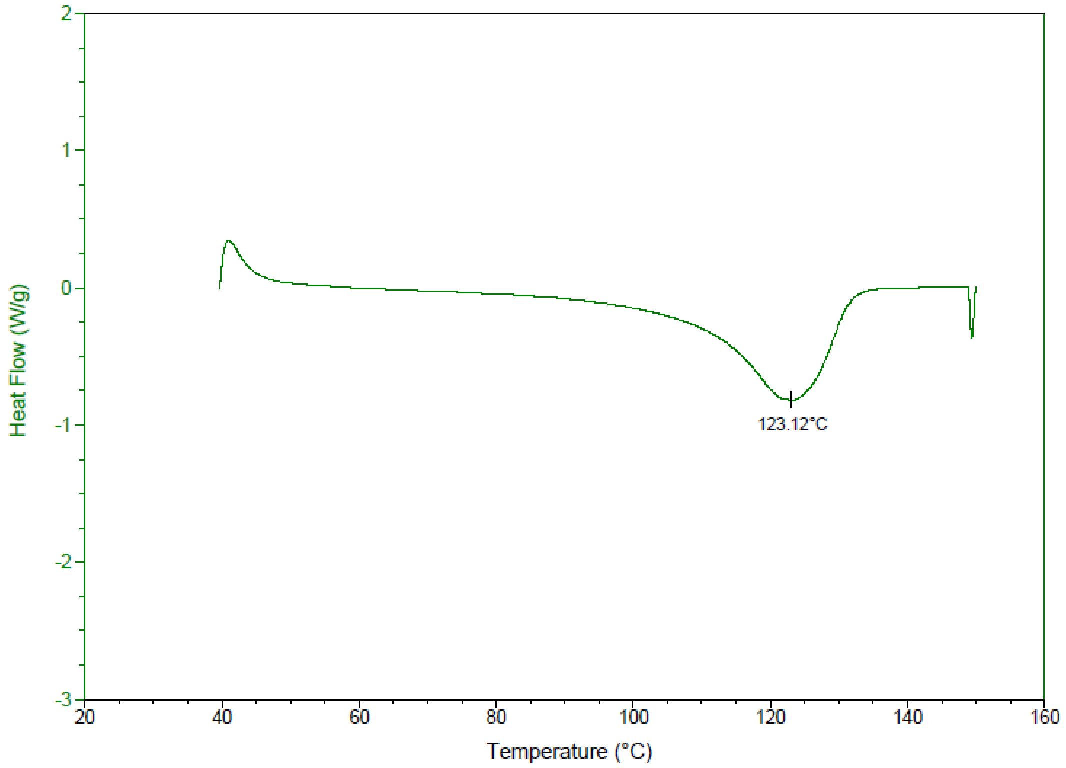

Supplementary Figure 109. DSC of the polymer from table 1, entry 7.

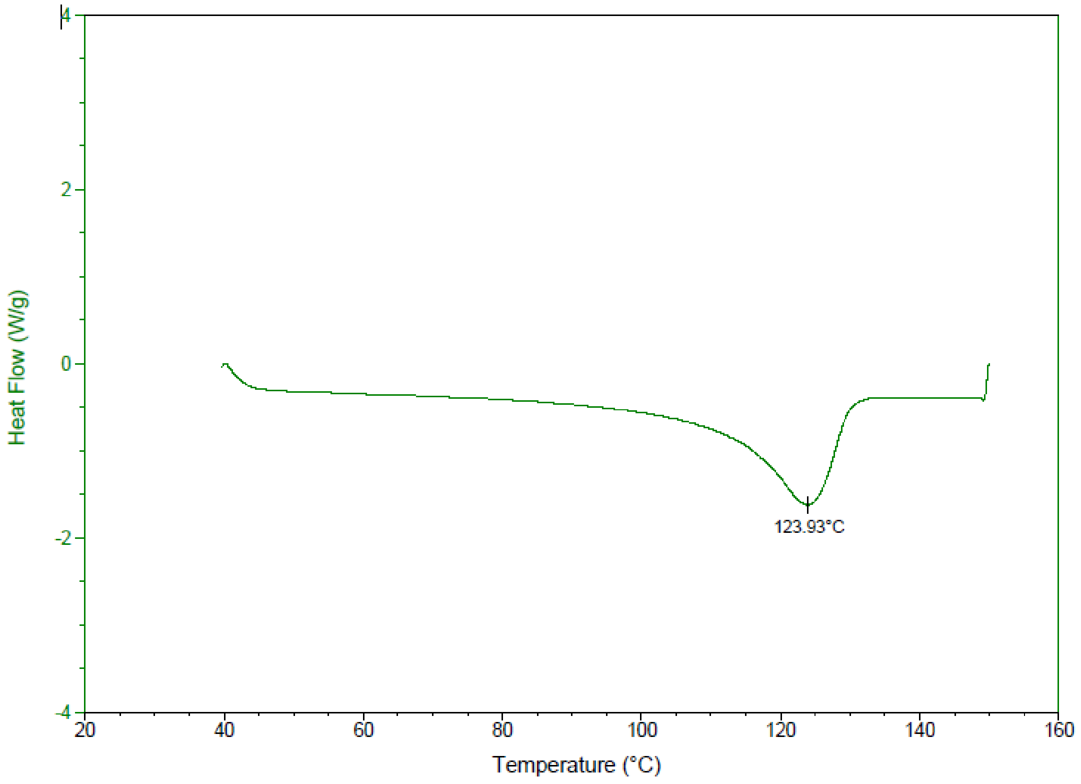

Supplementary Figure 110. DSC of the polymer from table 1, entry 8.

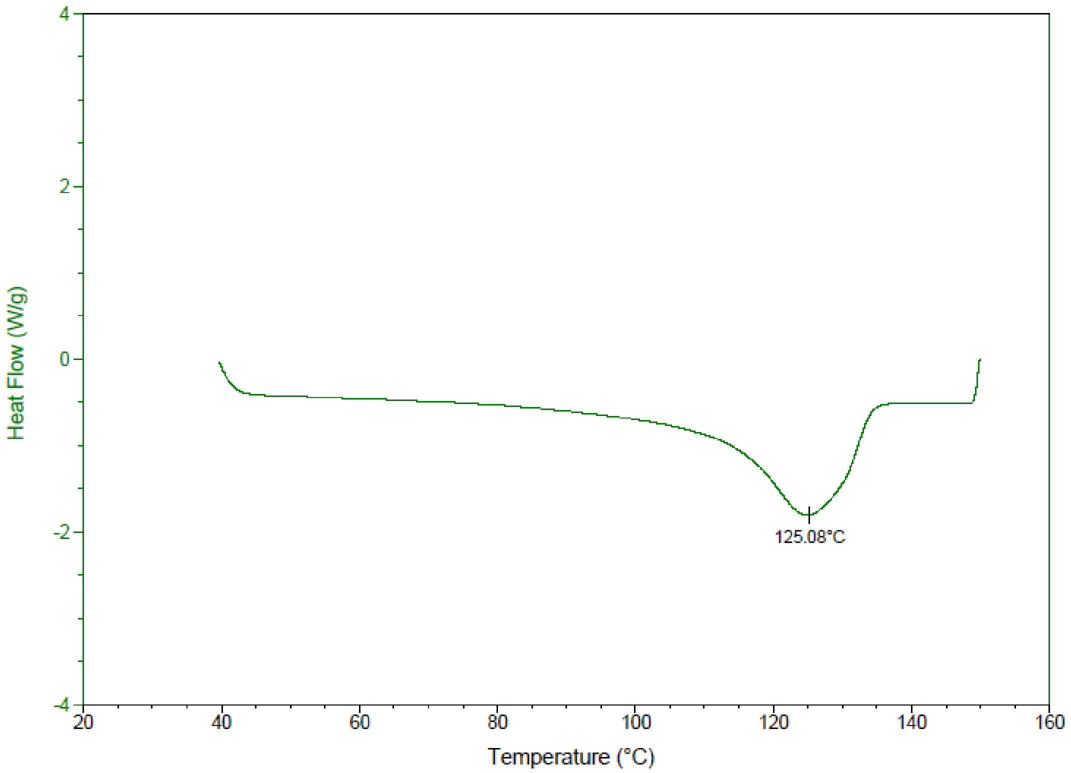

Supplementary Figure 111. DSC of the polymer from table 1, entry 9.

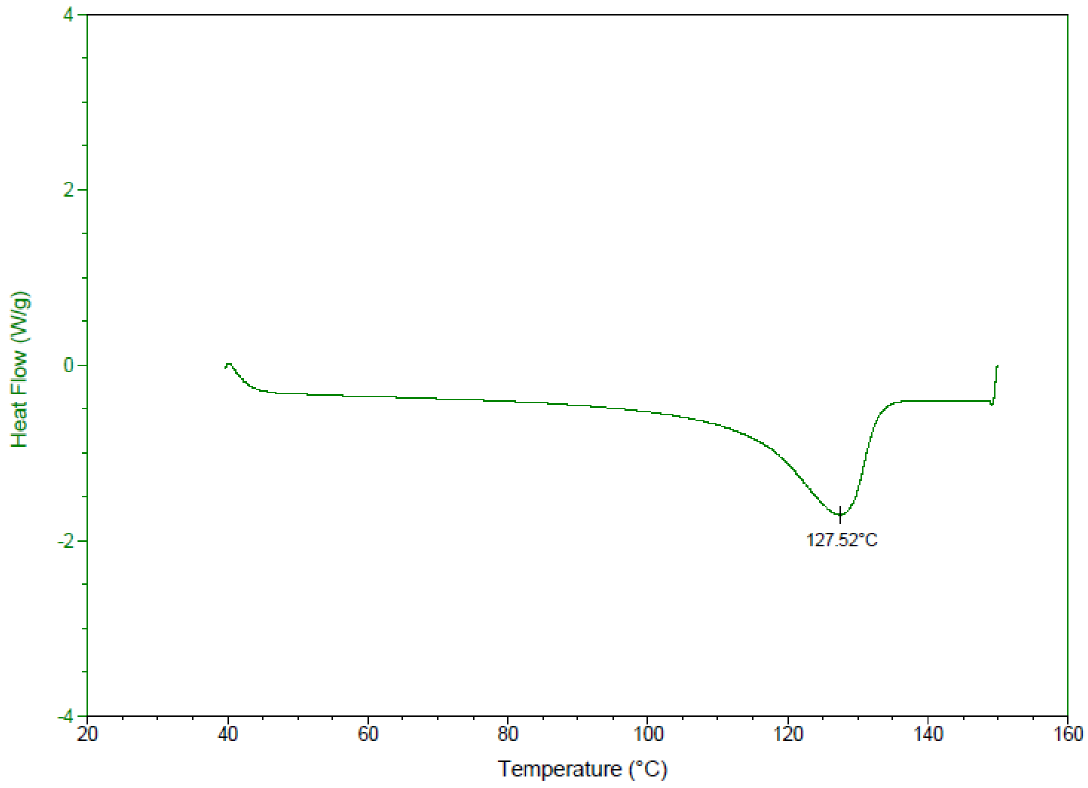

Supplementary Figure 112. DSC of the polymer from table 1, entry 10.

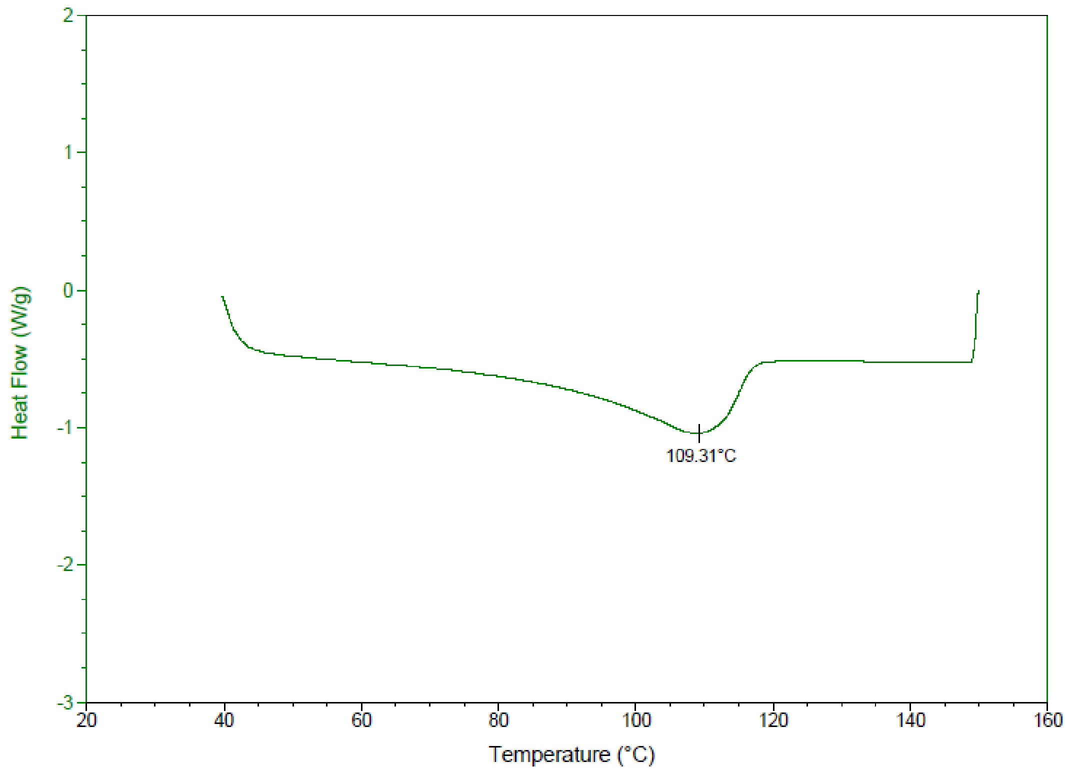

Supplementary Figure 113. DSC of the polymer from table 1, entry 11.

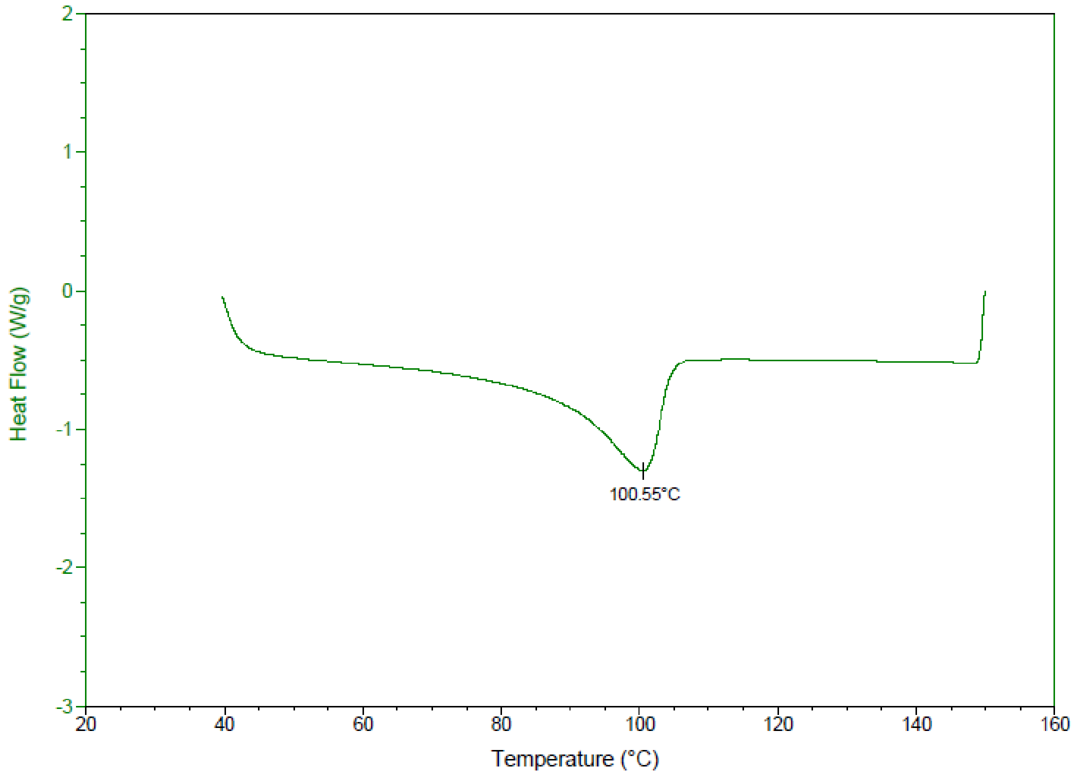

Supplementary Figure 114. DSC of the polymer from table 1, entry 12.

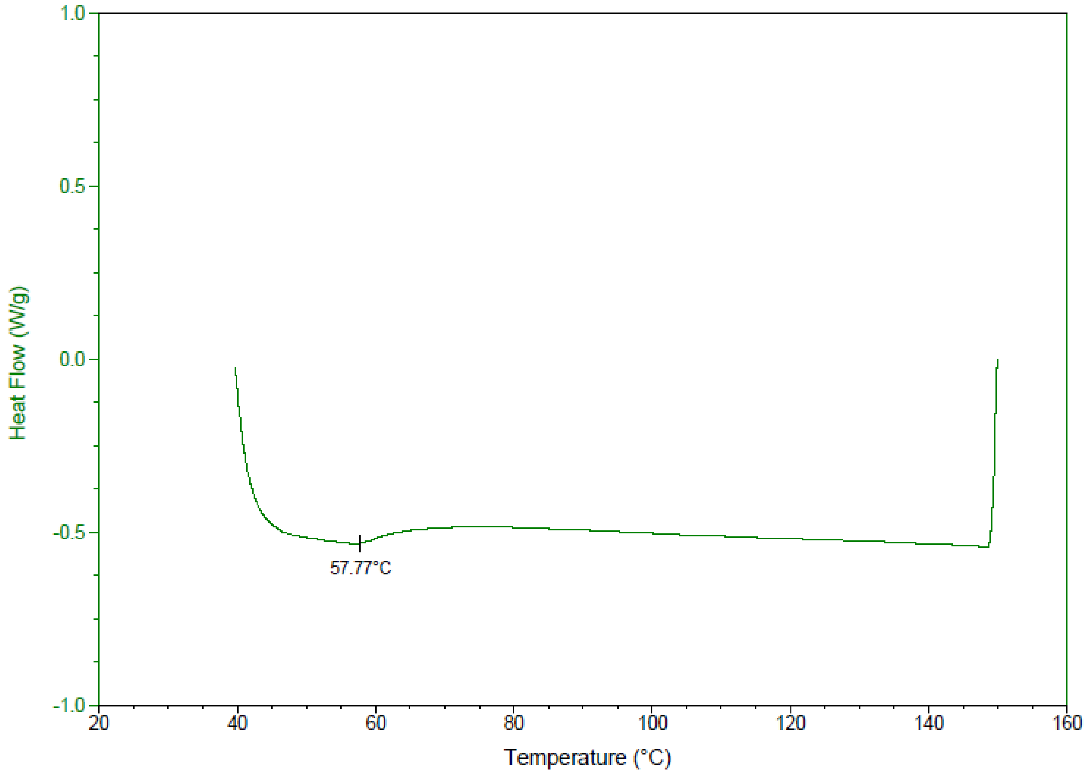

Supplementary Figure 115. DSC of the polymer from table 1, entry 13.

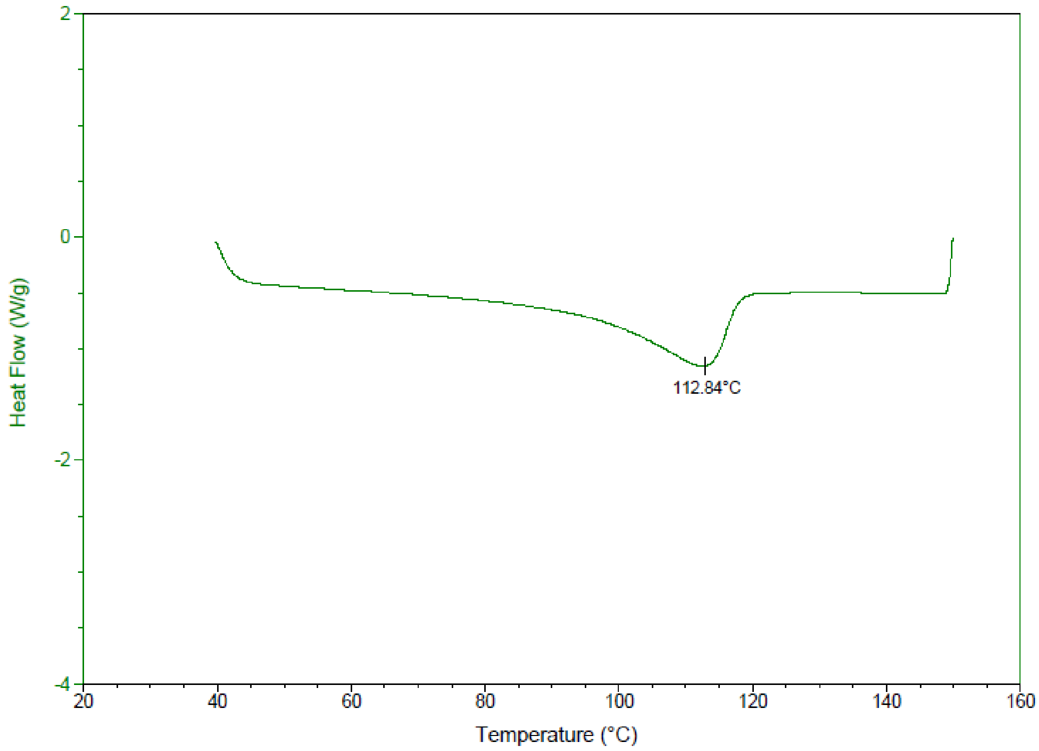

Supplementary Figure 116. DSC of the polymer from table 1, entry 14.

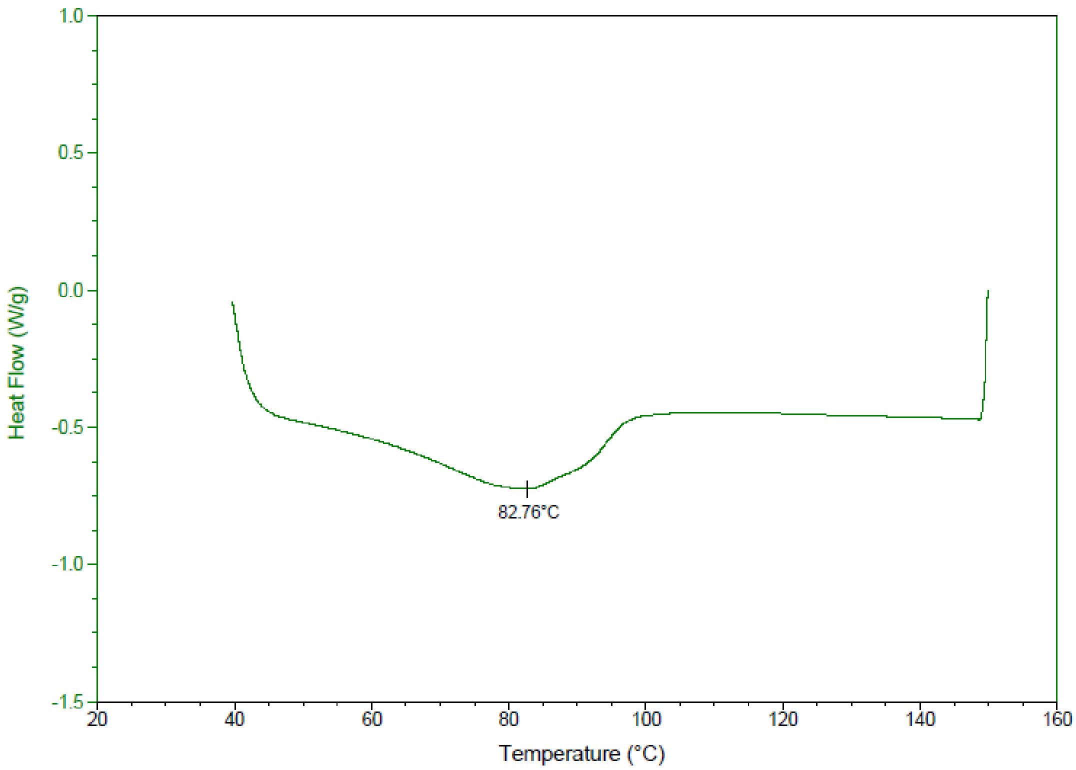

Supplementary Figure 117. DSC of the polymer from table 1, entry 15.

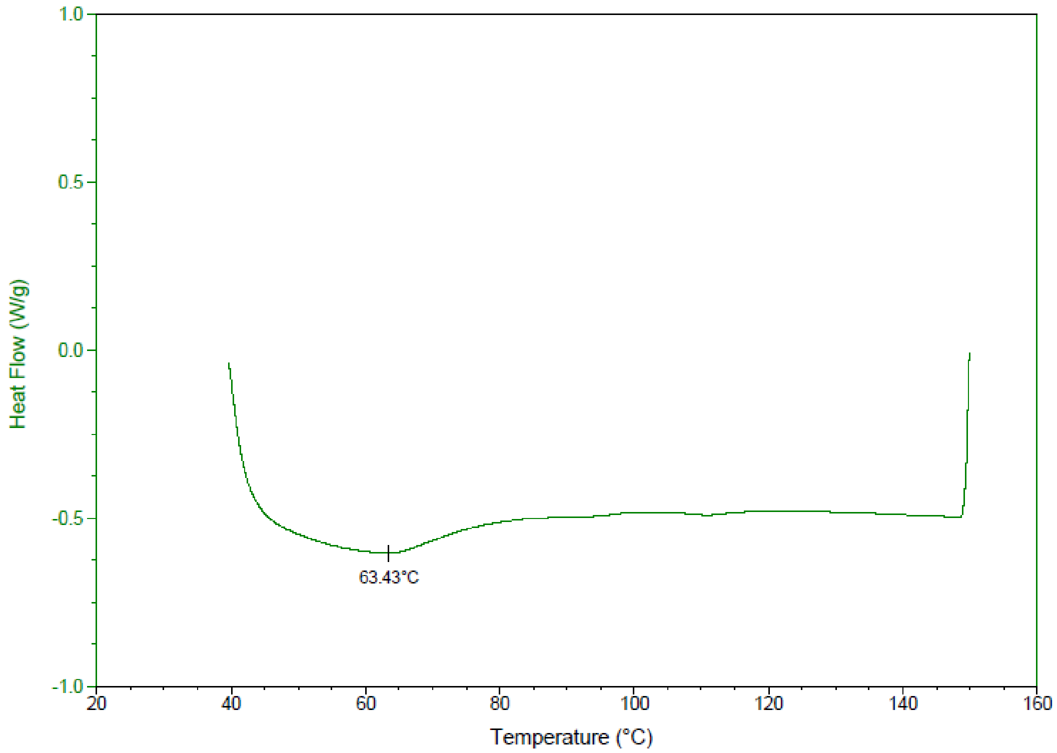

Supplementary Figure 118. DSC of the polymer from table 1, entry 16.

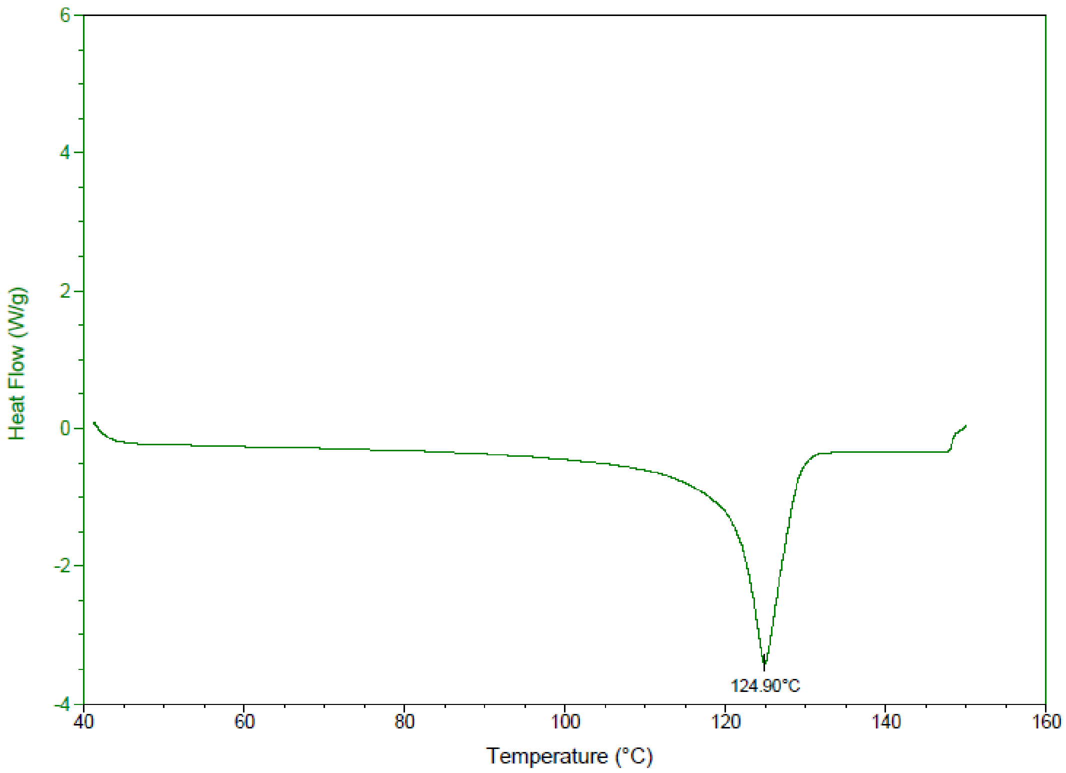

Supplementary Figure 119. DSC of the copolymer from table 2, entry 1.

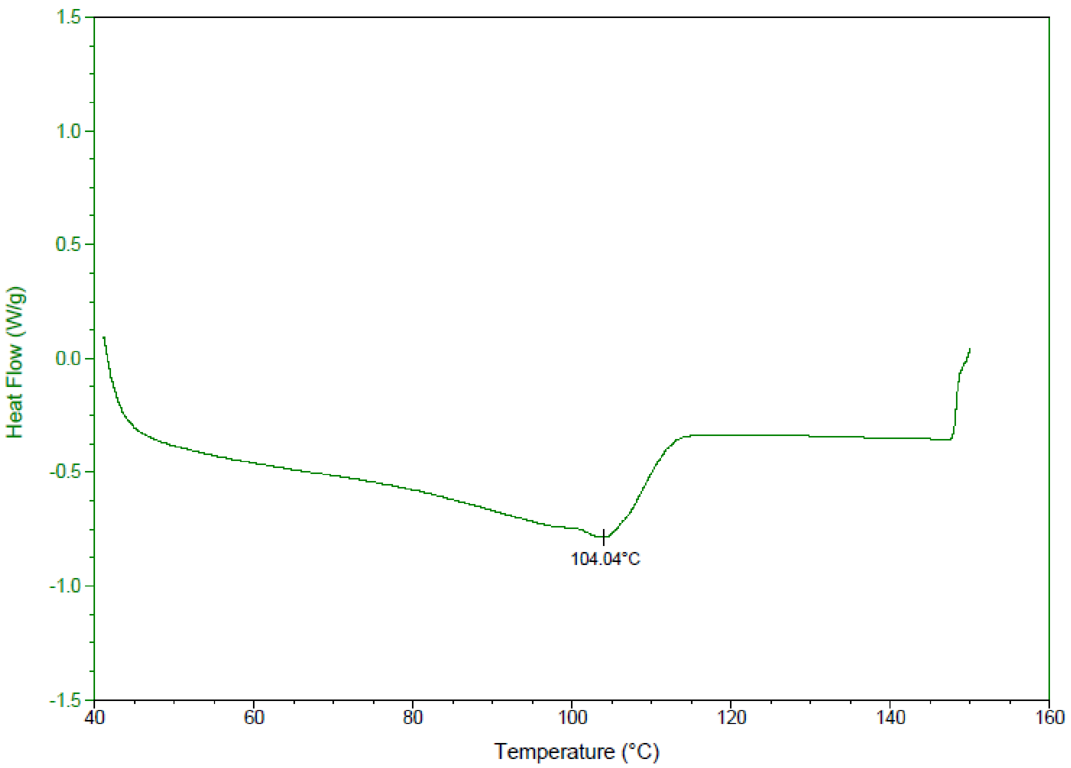

Supplementary Figure 120. DSC of the copolymer from table 2, entry 2.

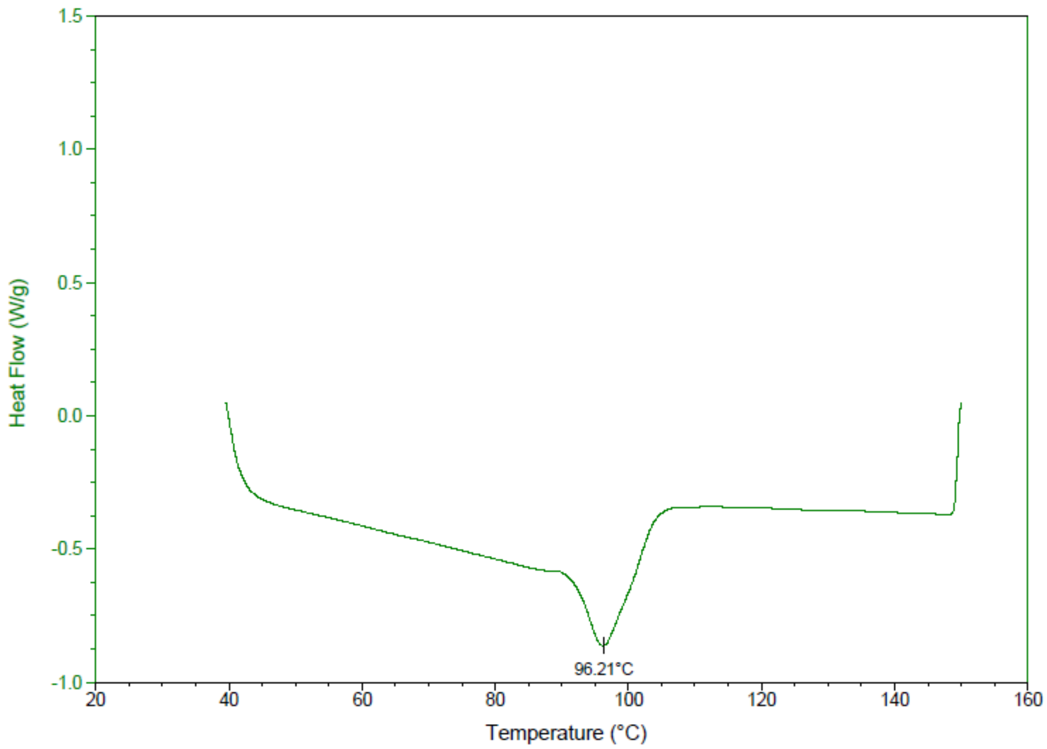

Supplementary Figure 121. DSC of the copolymer from table 2, entry 3.

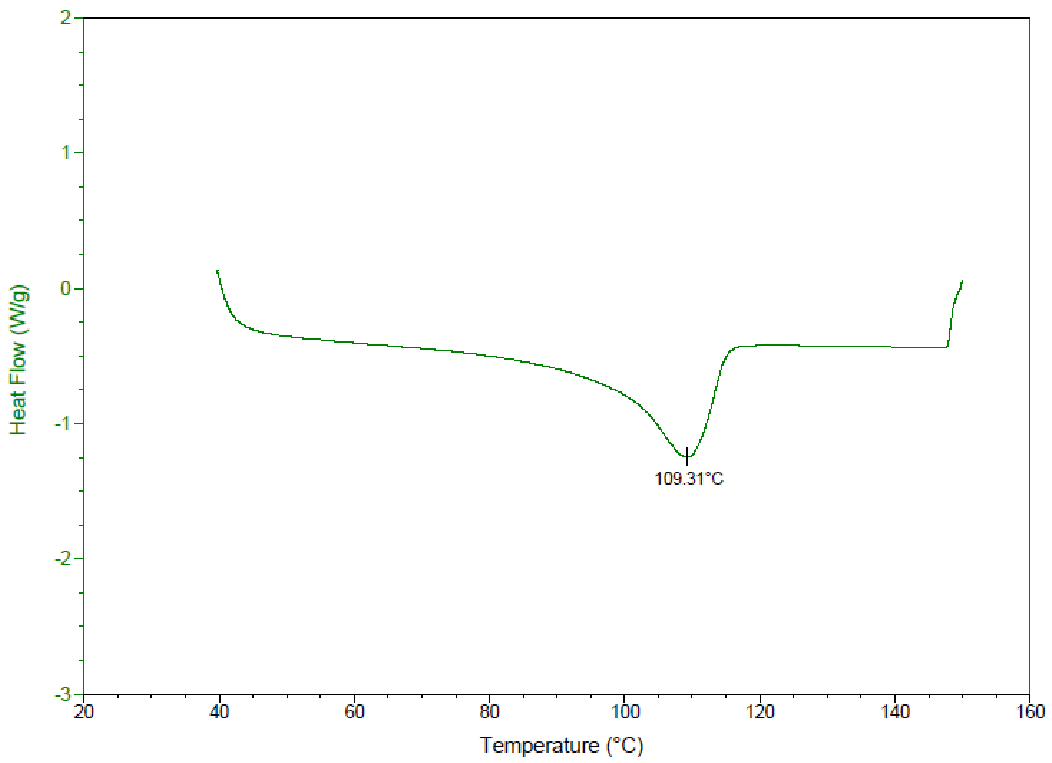

Supplementary Figure 122. DSC of the copolymer from table 2, entry 4.

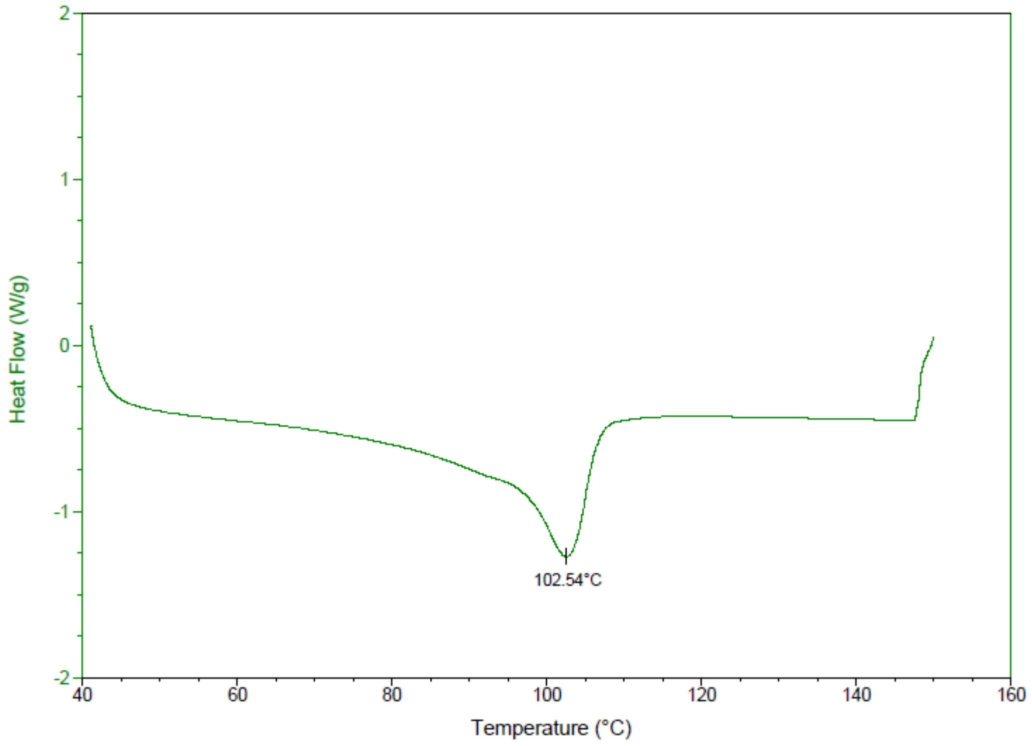

Supplementary Figure 123. DSC of the copolymer from table 2, entry 5.

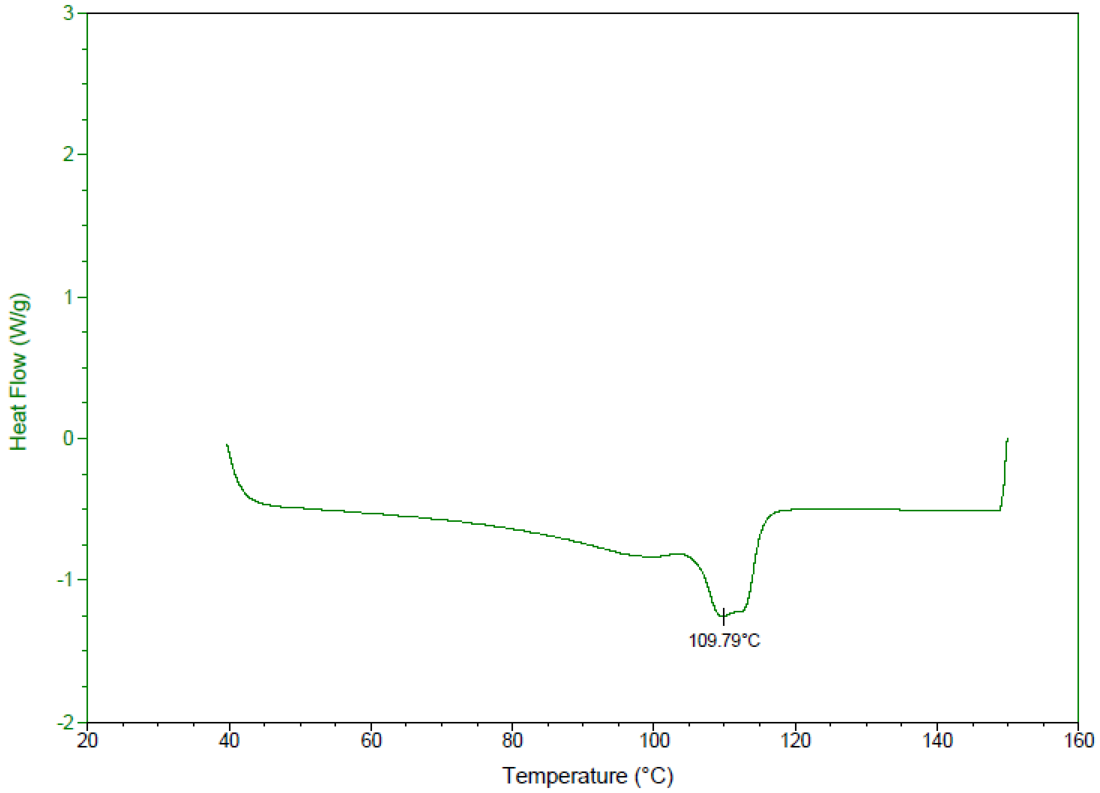

Supplementary Figure 124. DSC of the copolymer from table 2, entry 6.

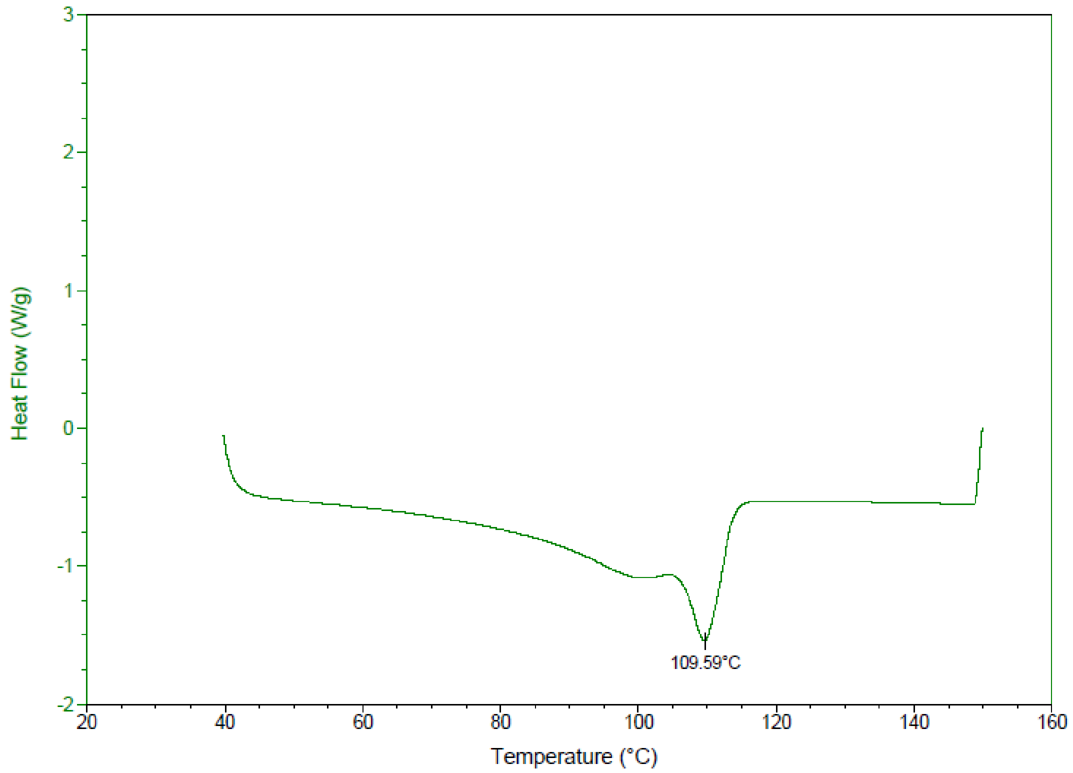

Supplementary Figure 125. DSC of the copolymer from table 2, entry 7.

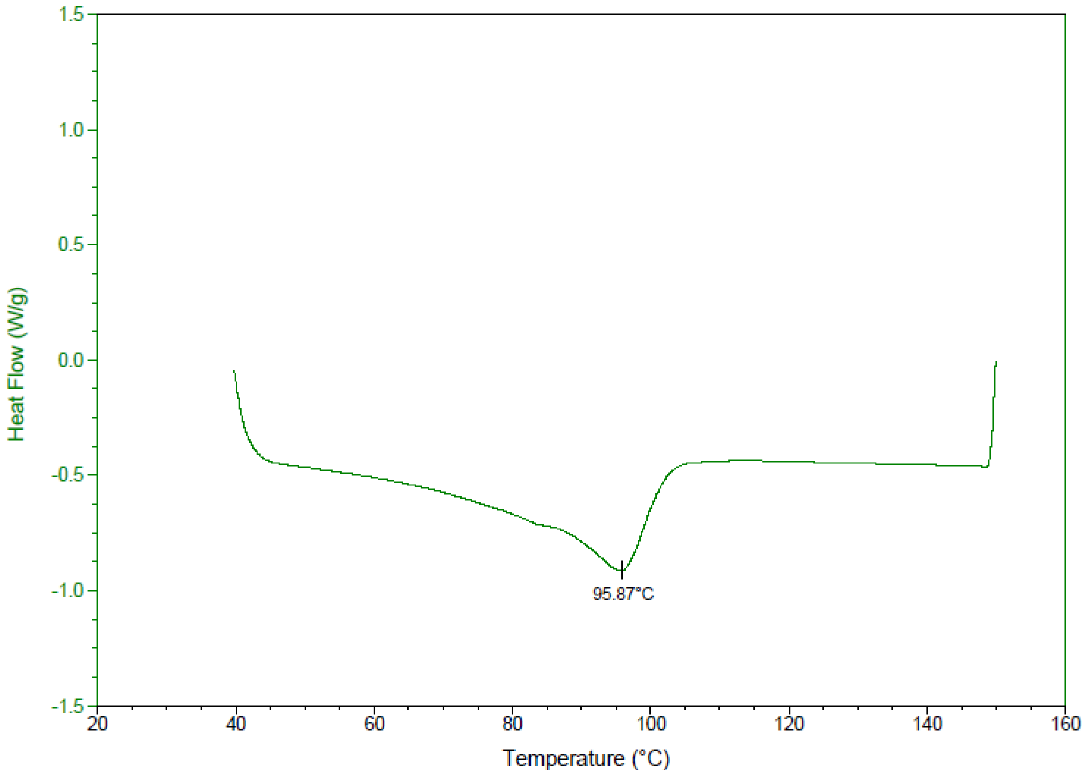

Supplementary Figure 126. DSC of the copolymer from table 2, entry 8.

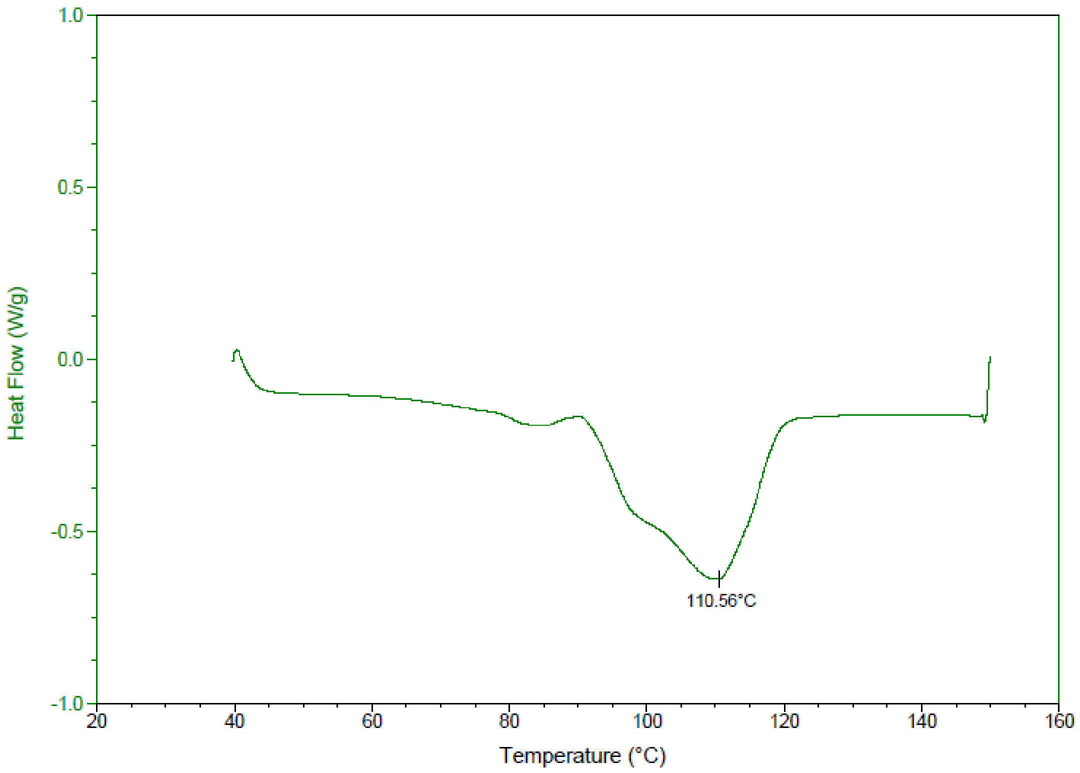

Supplementary Figure 127. DSC of the copolymer from table 2, entry 9.

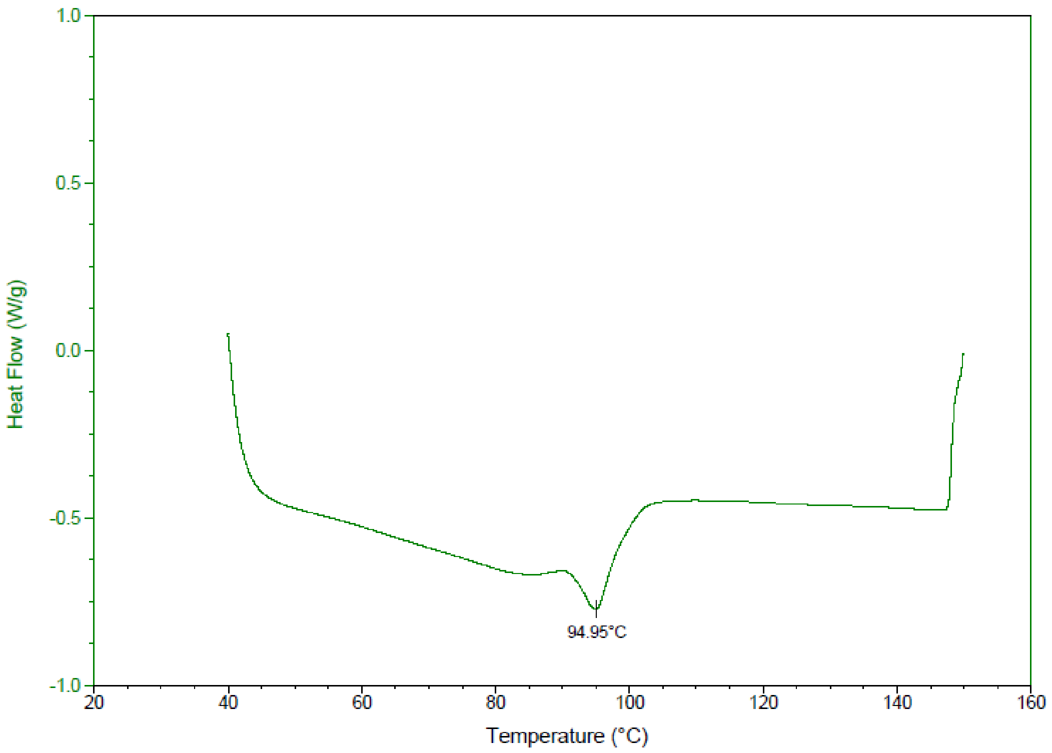

Supplementary Figure 128. DSC of the copolymer from table 2, entry 10.

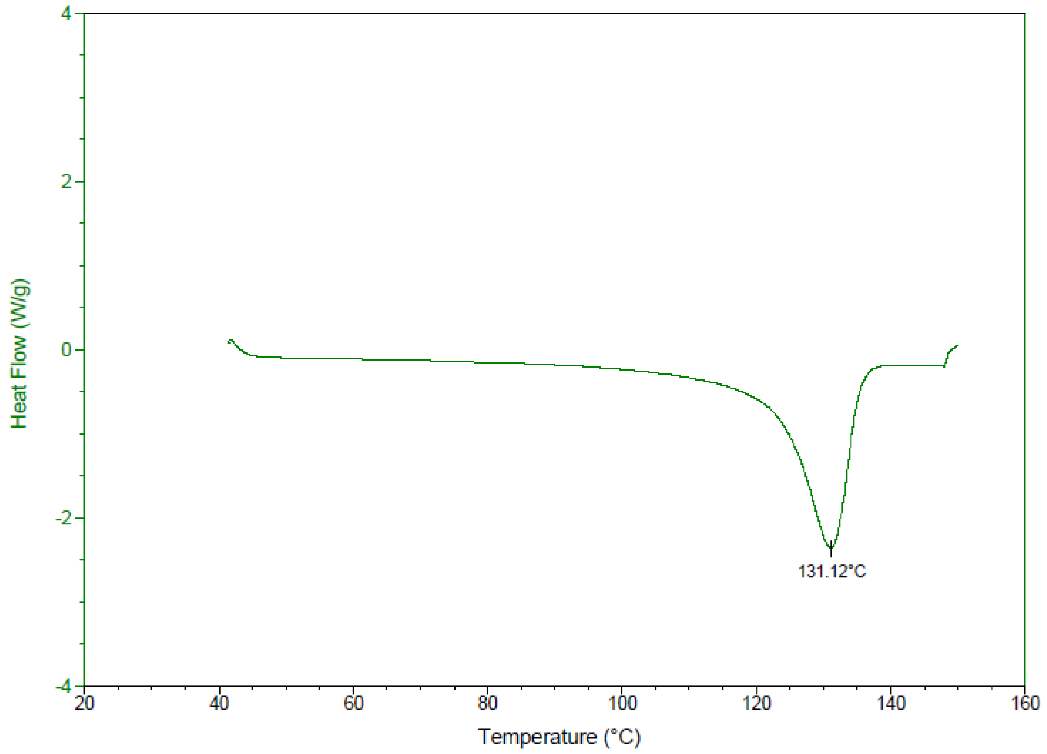

Supplementary Figure 129. DSC of the polymer from table 3, entry 1.

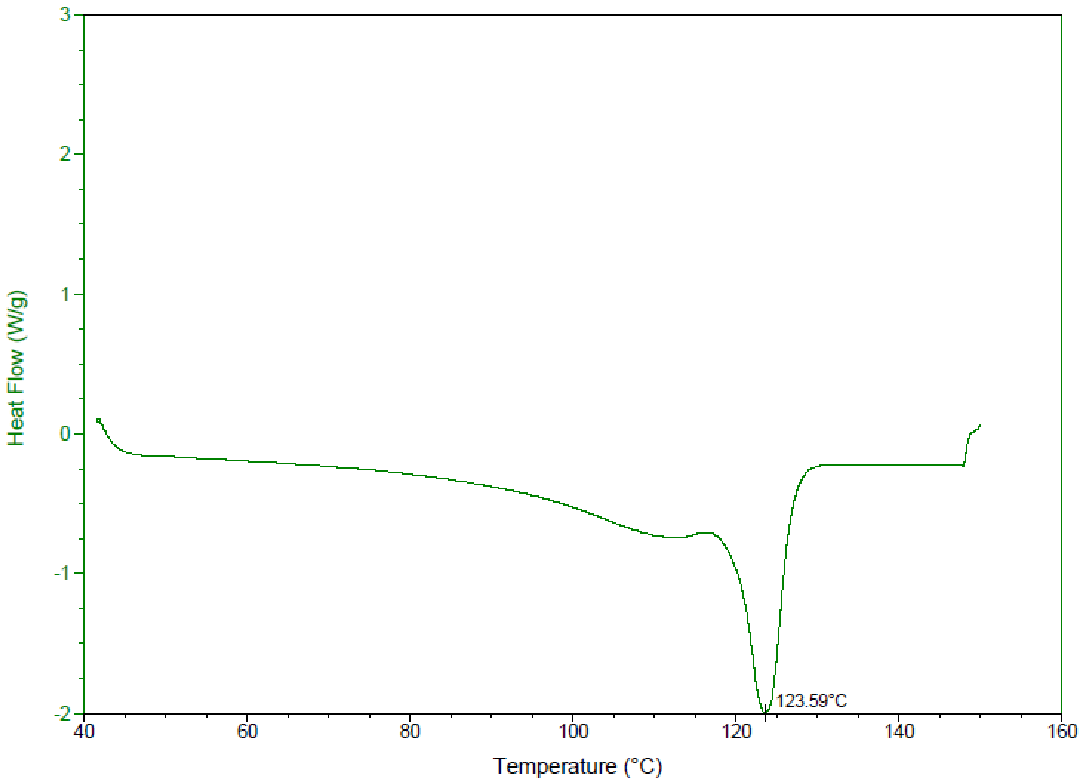

Supplementary Figure 130. DSC of the polymer from table 3, entry 2.

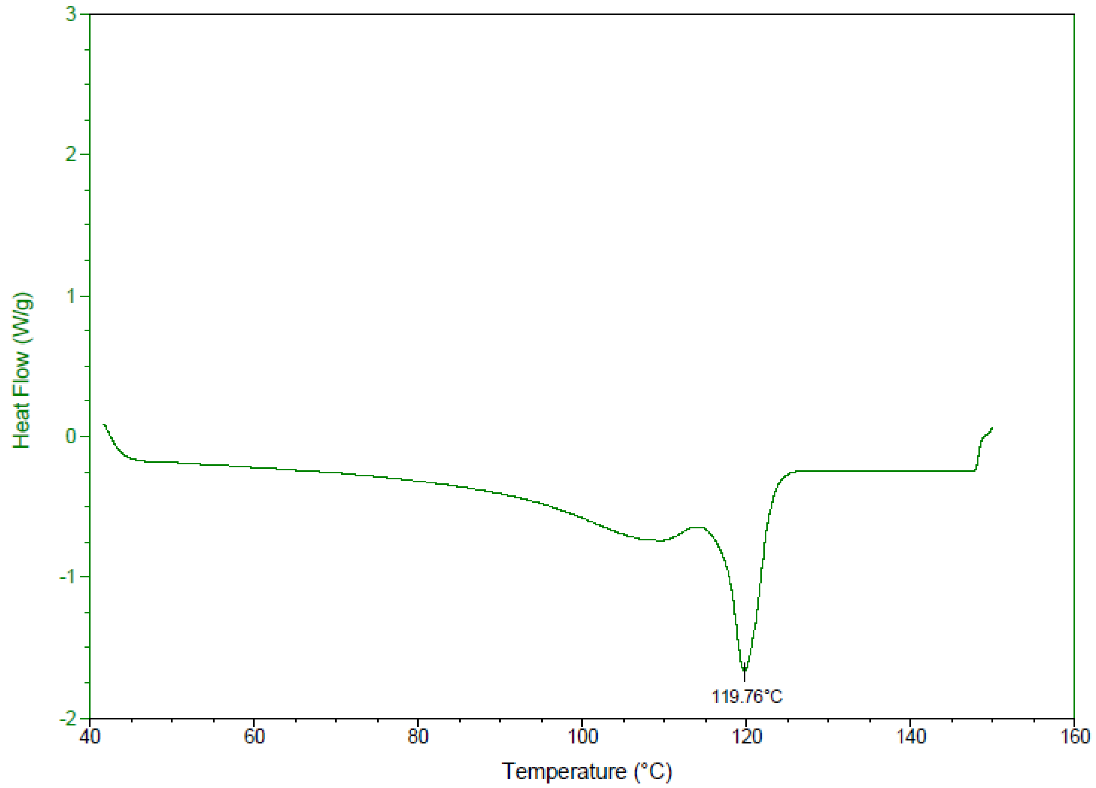

Supplementary Figure 131. DSC of the polymer from table 3, entry 3.

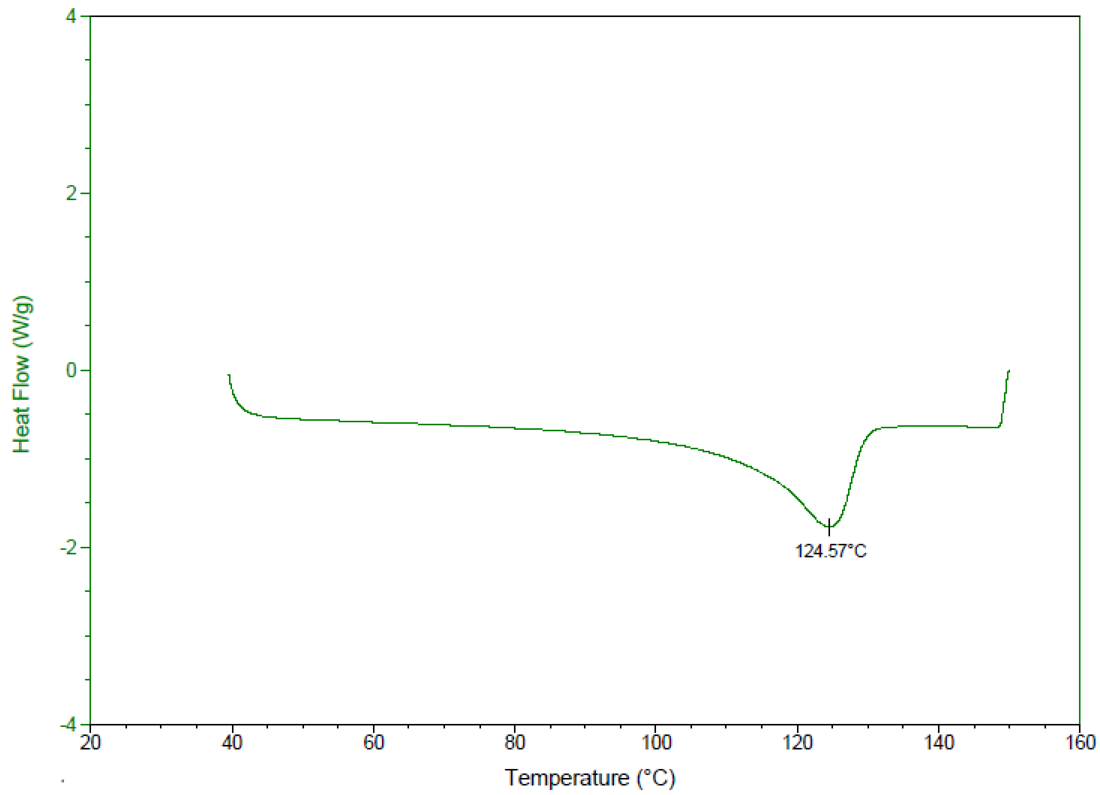

Supplementary Figure 132. DSC of the polymer from table 3, entry 4.

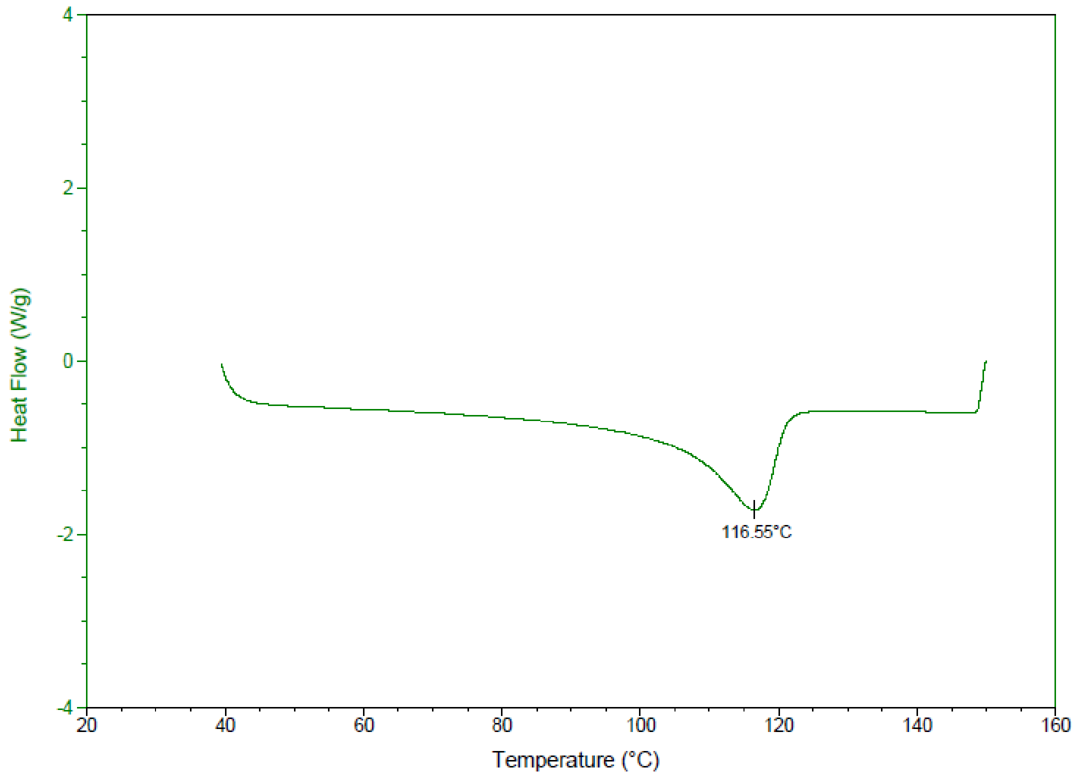

Supplementary Figure 133. DSC of the polymer from table 3, entry 5.

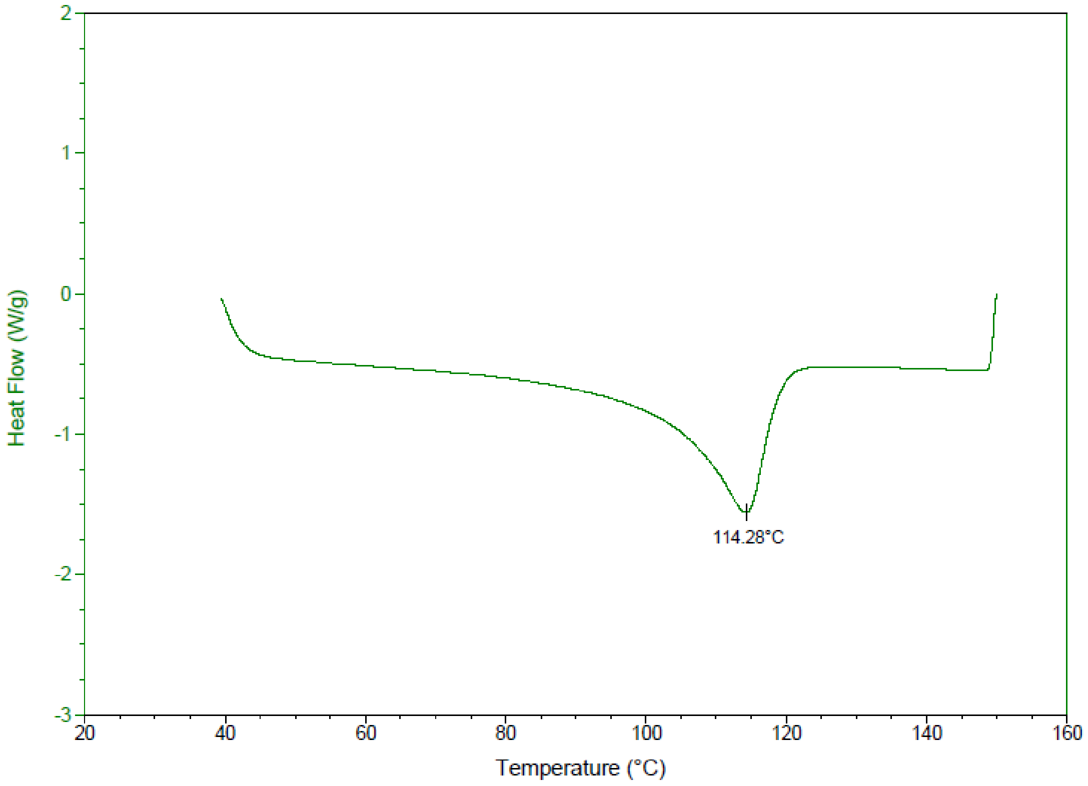

Supplementary Figure 134. DSC of the polymer from table 3, entry 6.

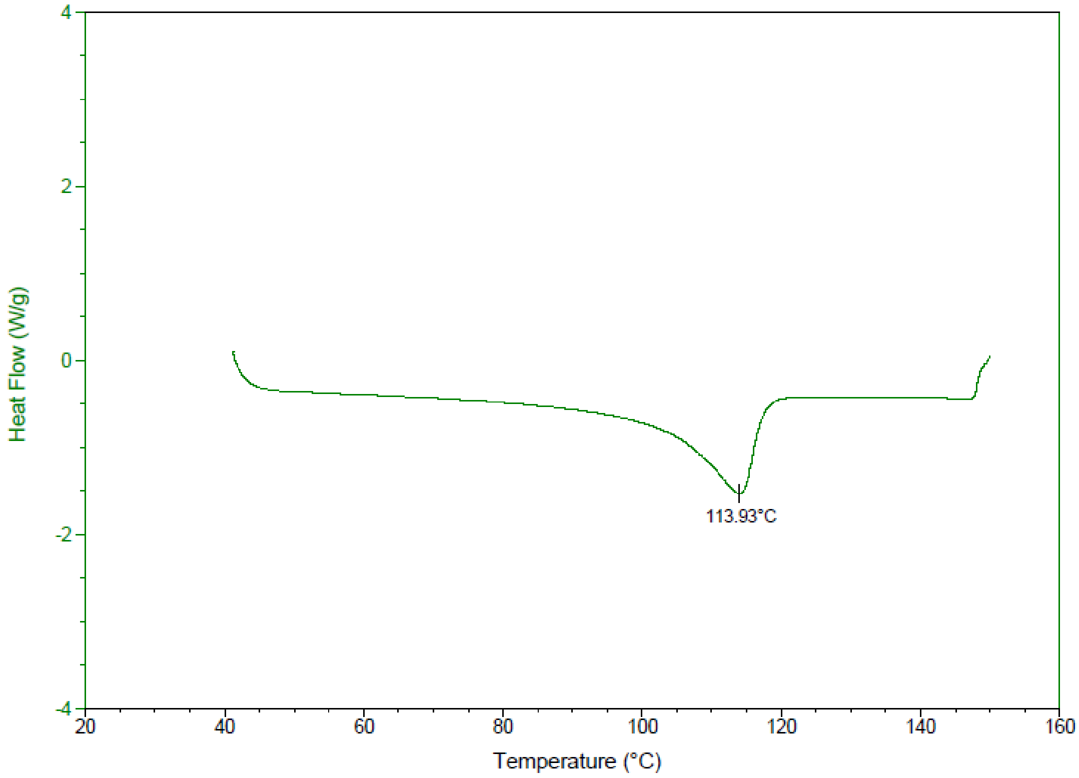

Supplementary Figure 135. DSC of the polymer from table 3, entry 7.

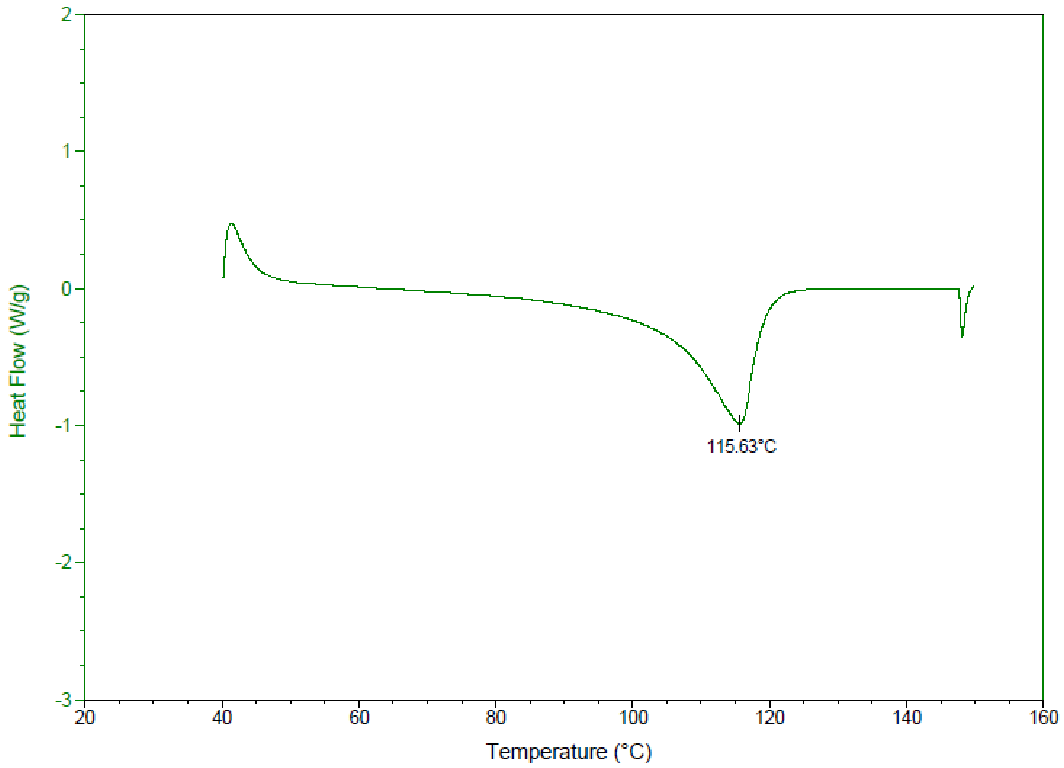

Supplementary Figure 136. DSC of the polymer from table 3, entry 8.

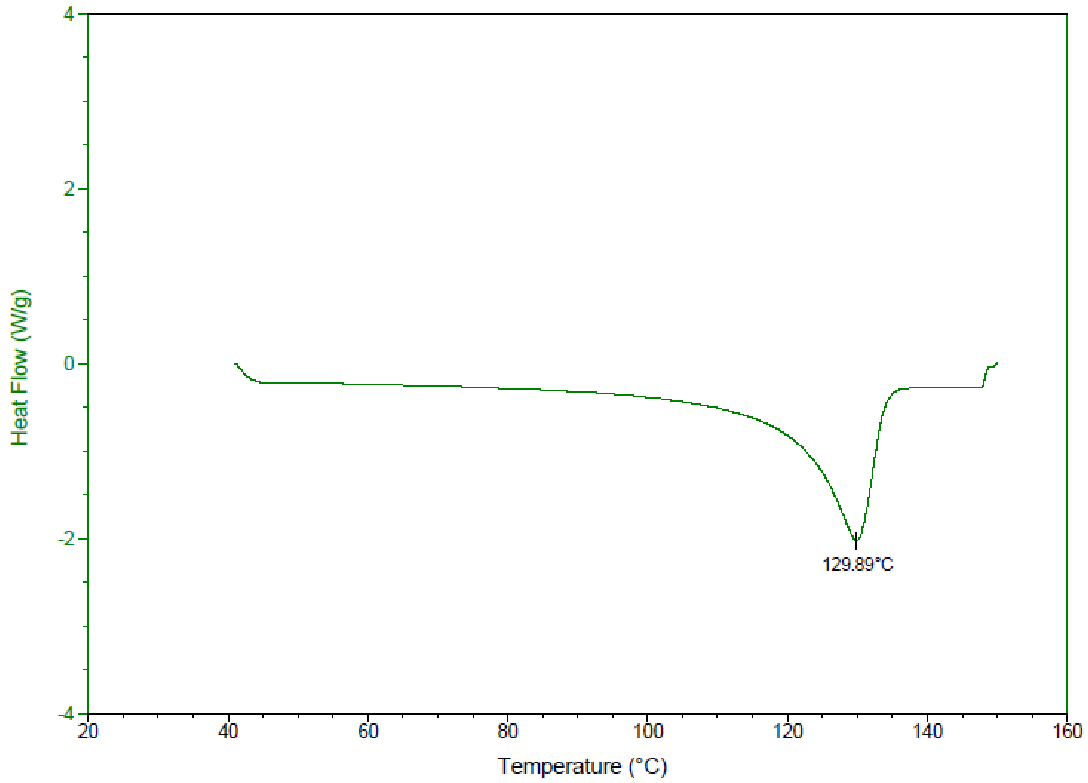

Supplementary Figure 137. DSC of the polymer from table 3, entry 9.

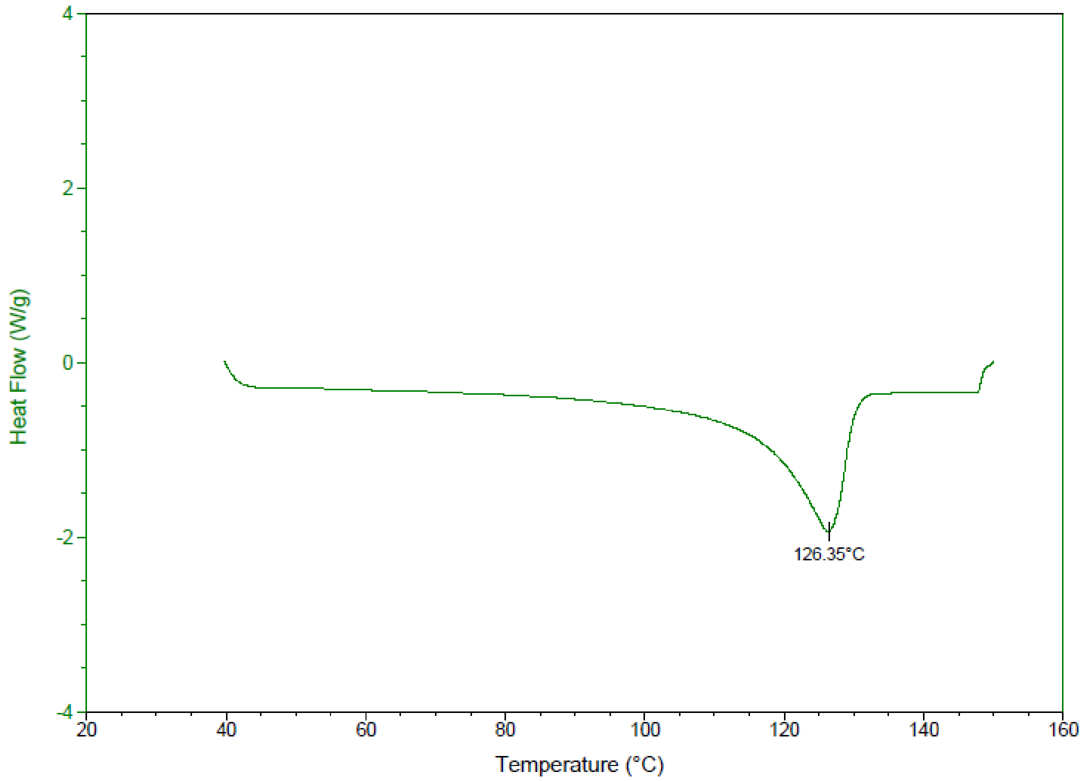

Supplementary Figure 138. DSC of the polymer from table 3, entry 10.

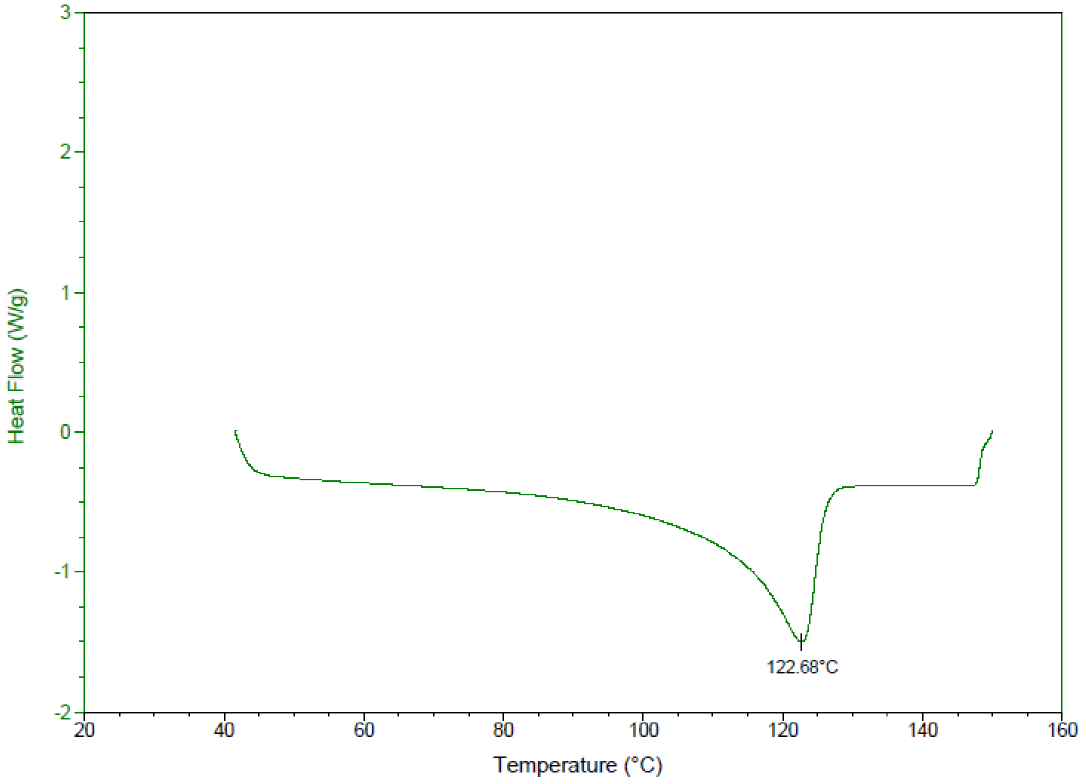

Supplementary Figure 139. DSC of the polymer from table 3, entry 11.

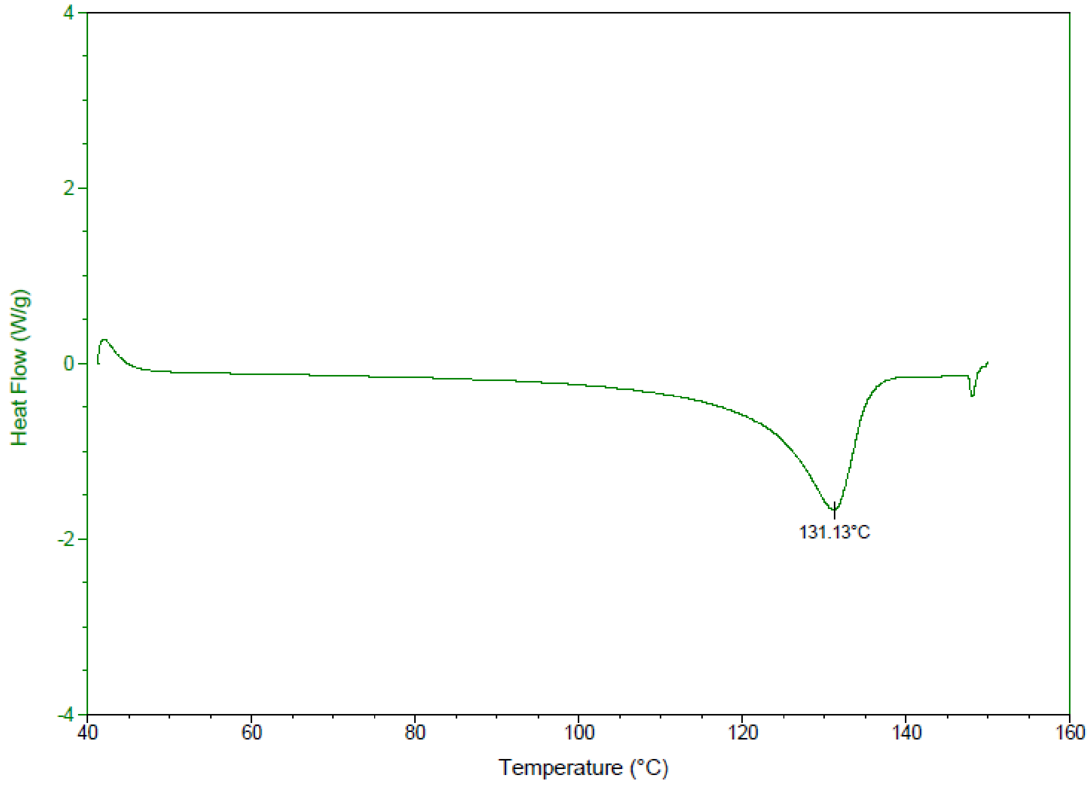

Supplementary Figure 140. DSC of the polymer from table 3, entry 12.

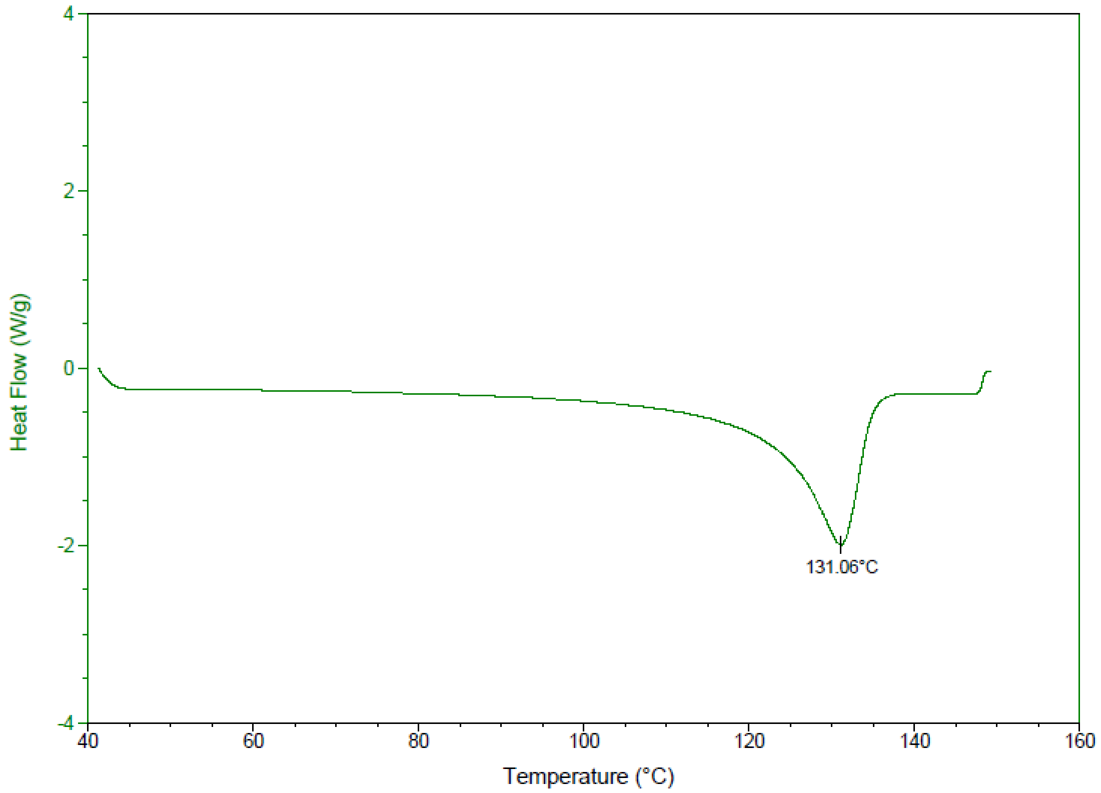

Supplementary Figure 141. DSC of the polymer from table 3, entry 13.

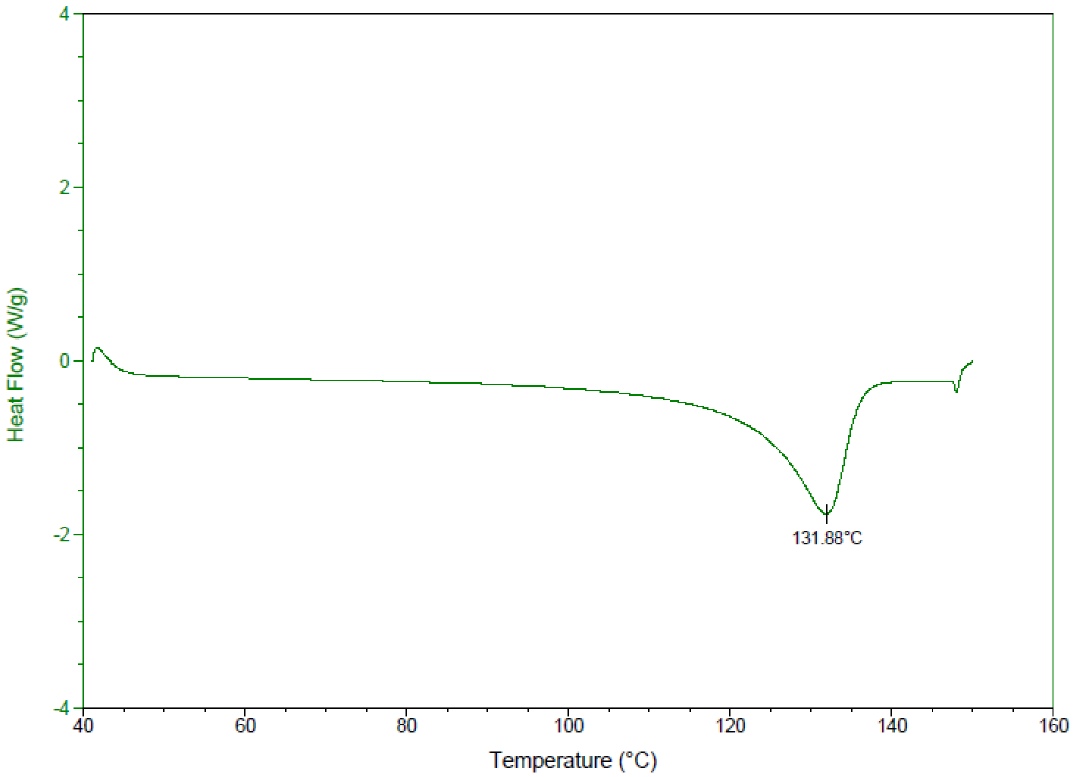

Supplementary Figure 142. DSC of the polymer from table 3, entry 14.

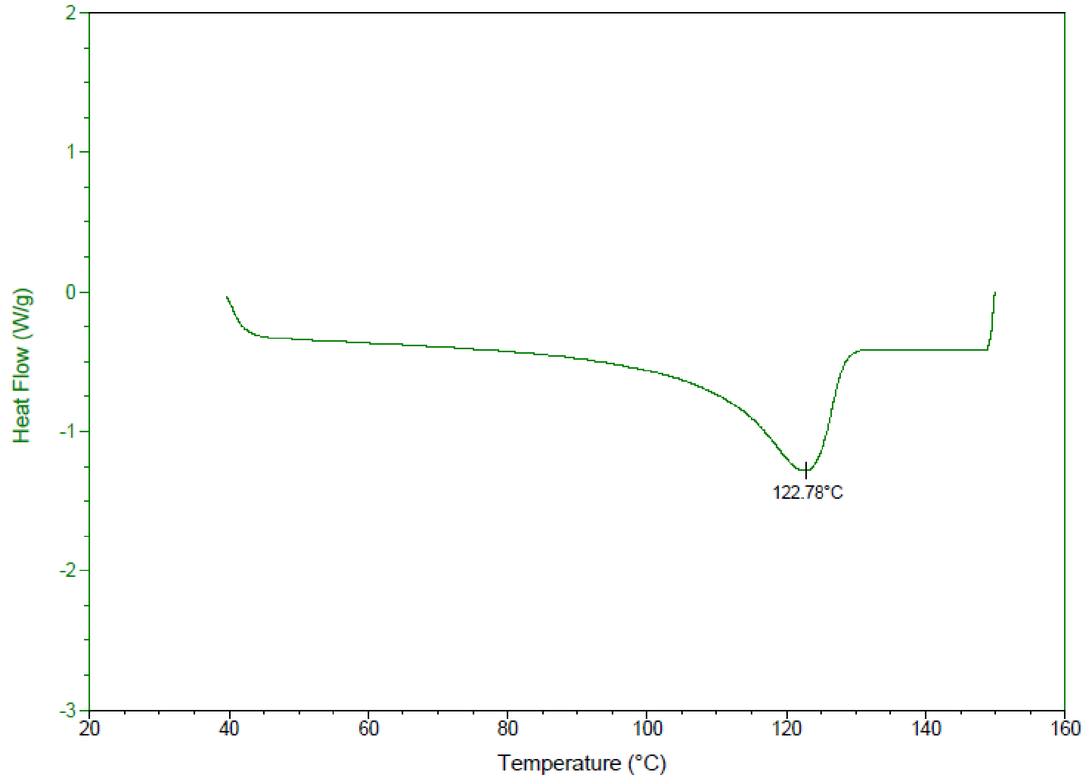

Supplementary Figure 143. DSC of the polymer from supplementary table 1, entry 1.

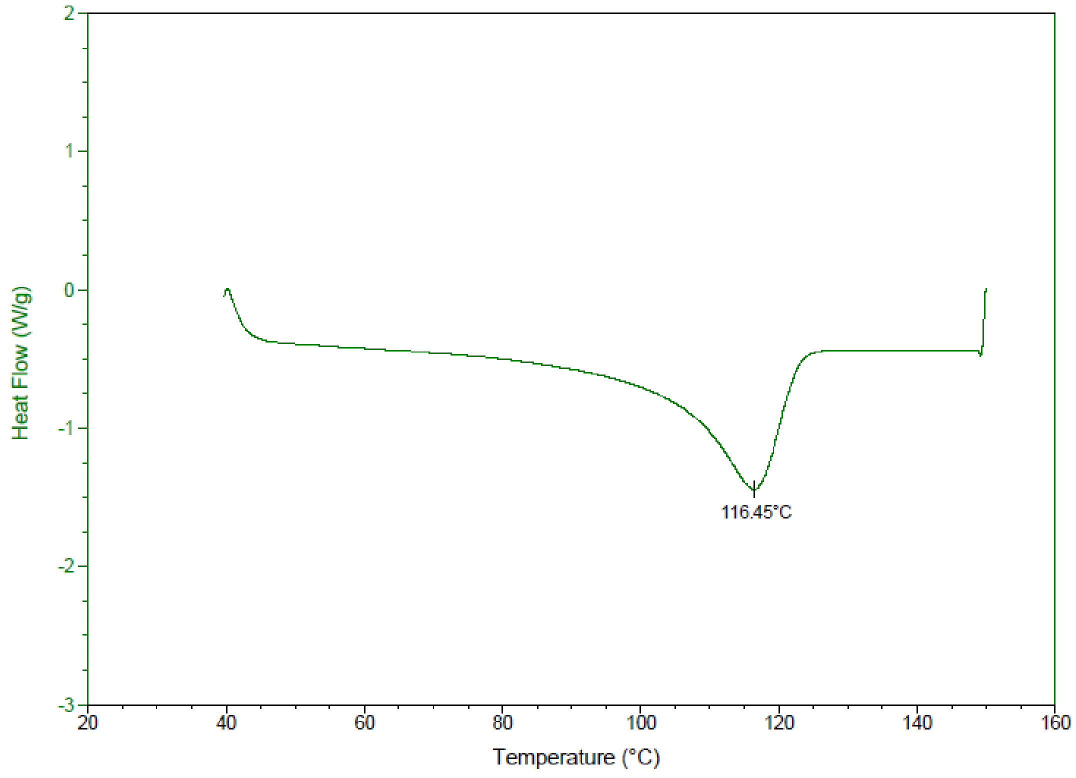

Supplementary Figure 144. DSC of the polymer from supplementary table 1, entry 2.

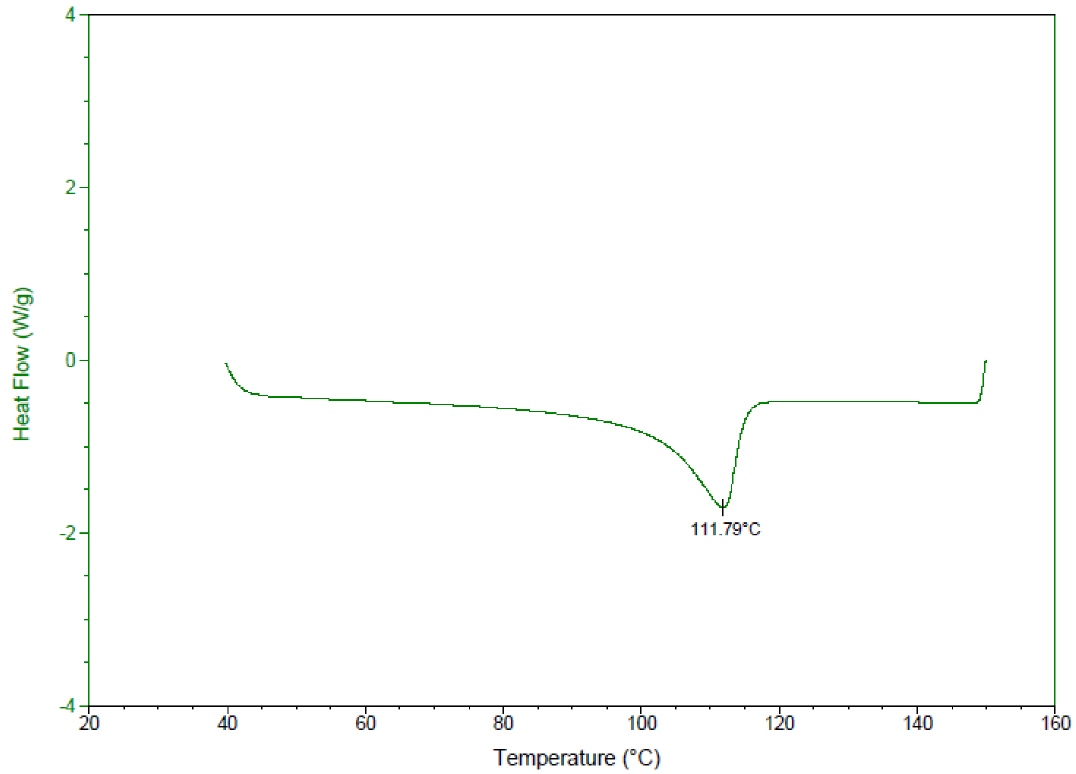

Supplementary Figure 145. DSC of the polymer from supplementary table 1, entry 3.

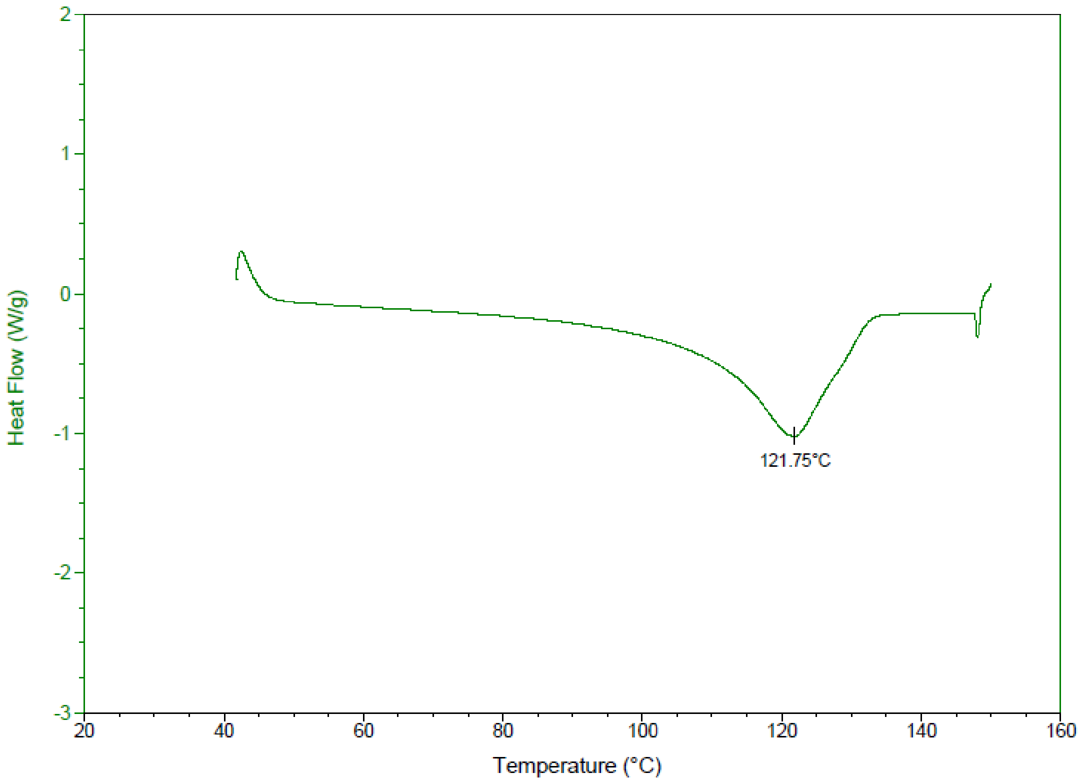

Supplementary Figure 146. DSC of the polymer from supplementary table 1, entry 4.

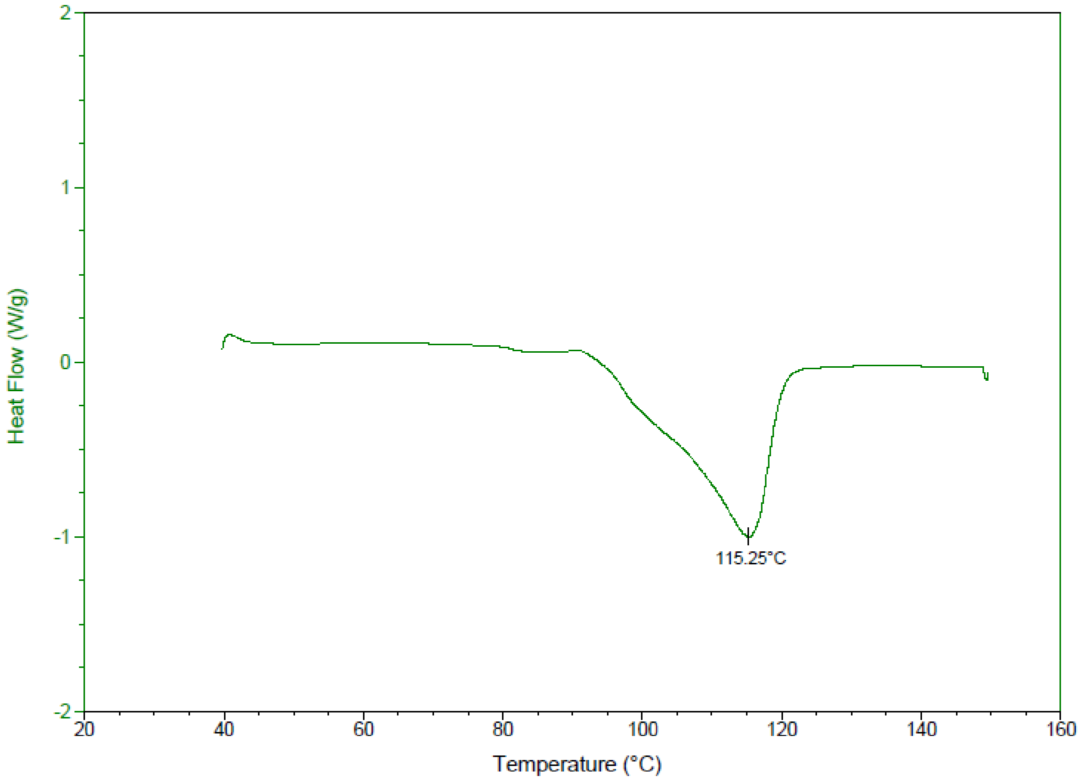

Supplementary Figure 147. DSC of the polymer from supplementary table 1, entry 5.

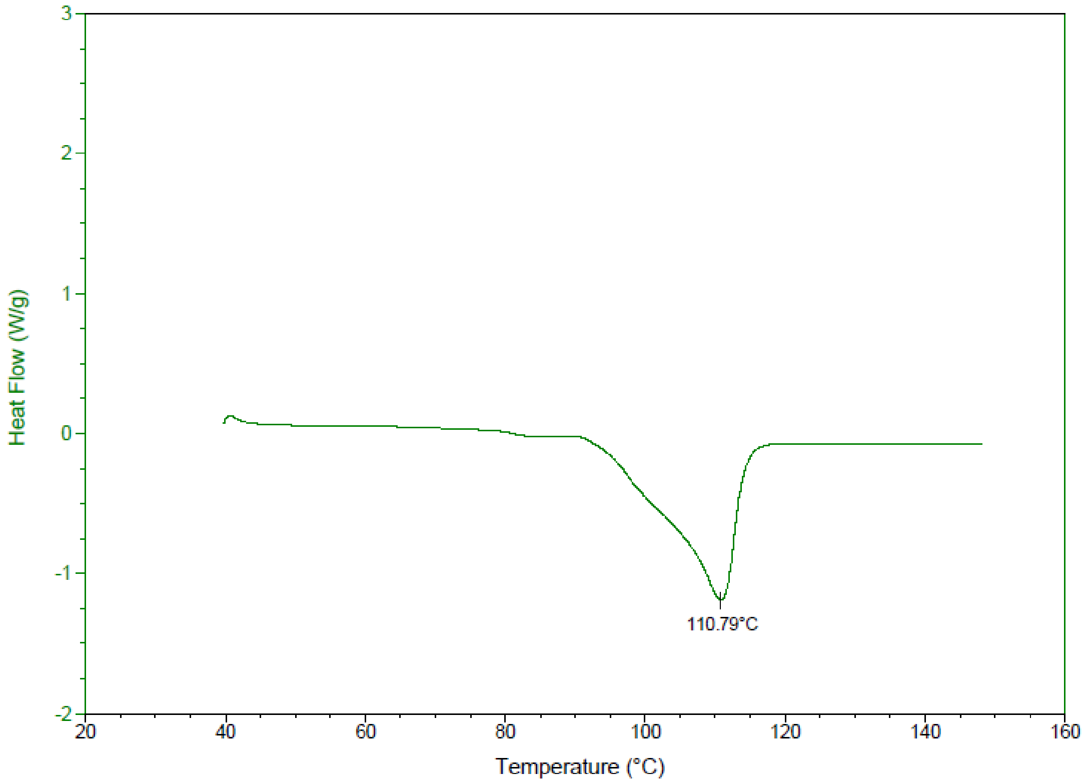

Supplementary Figure 148. DSC of the polymer from supplementary table 1, entry 6.

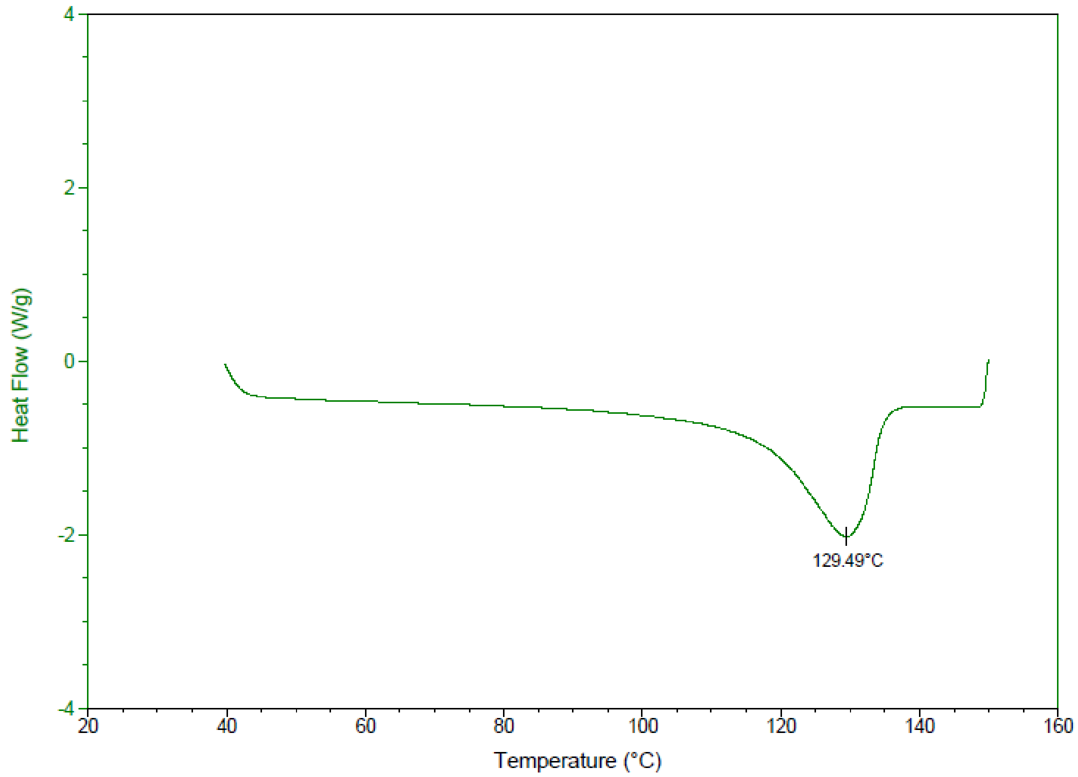

Supplementary Figure 149. DSC of the polymer from supplementary table 1, entry 7.

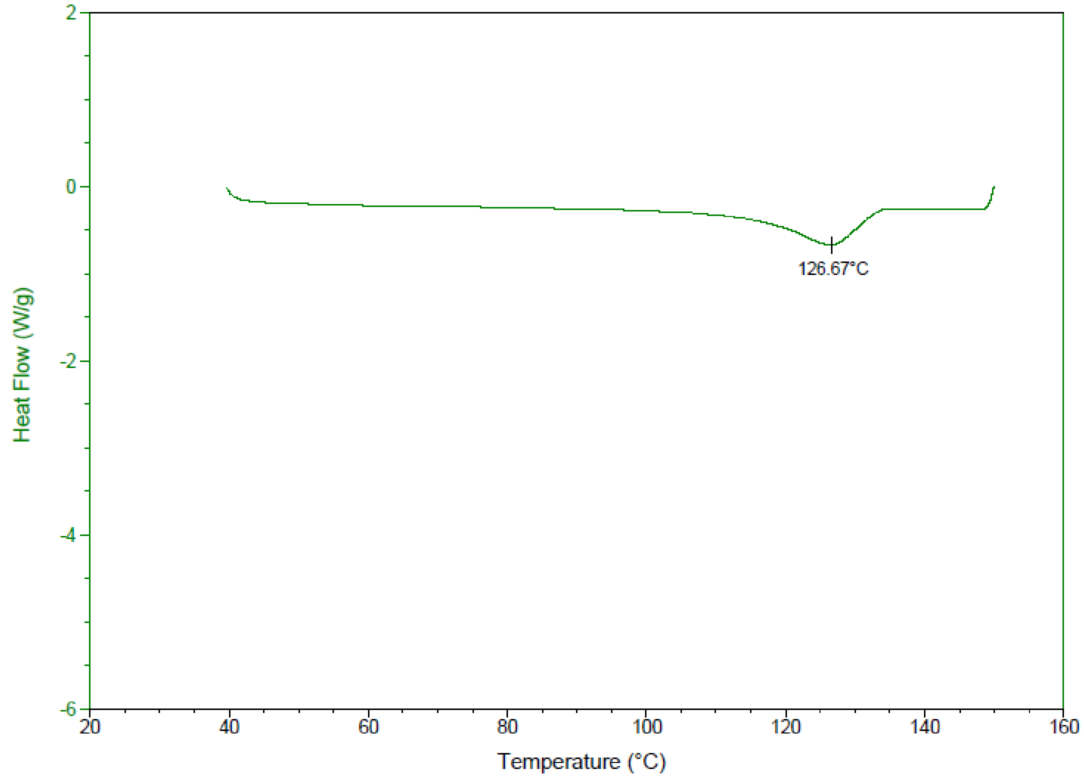

Supplementary Figure 150. DSC of the polymer from supplementary table 1, entry 8.

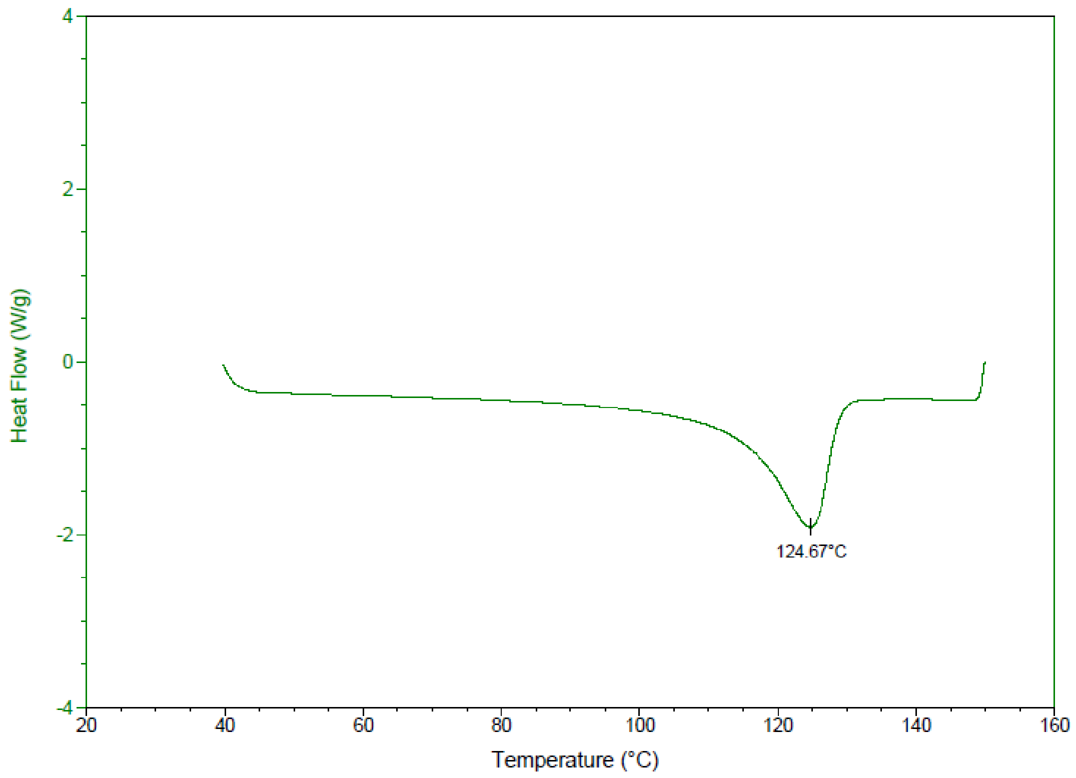

Supplementary Figure 151. DSC of the polymer from supplementary table 1, entry 9.

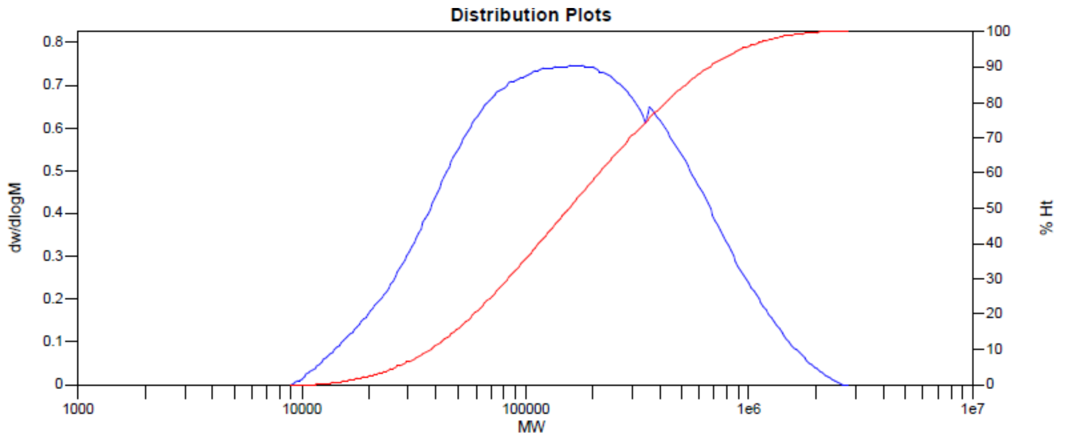

MW Averages

Peak No	Mp	Mn	Mw	Mz	Mz+1	Mv	PD
1	175726	85746	263244	622554	1006528	223603	3.07004

Processed Peaks

Peak No	Name	Start RT (mins)	Max RT (mins)	End RT (mins)	Pk Height (mV)	% Height	Area (mV.secs)	% Area
1		11.50	13.27	15.33	-37.4552	100	4527.93	100

Supplementary Figure 152. GPC data of the polymer from table 1, entry 1.

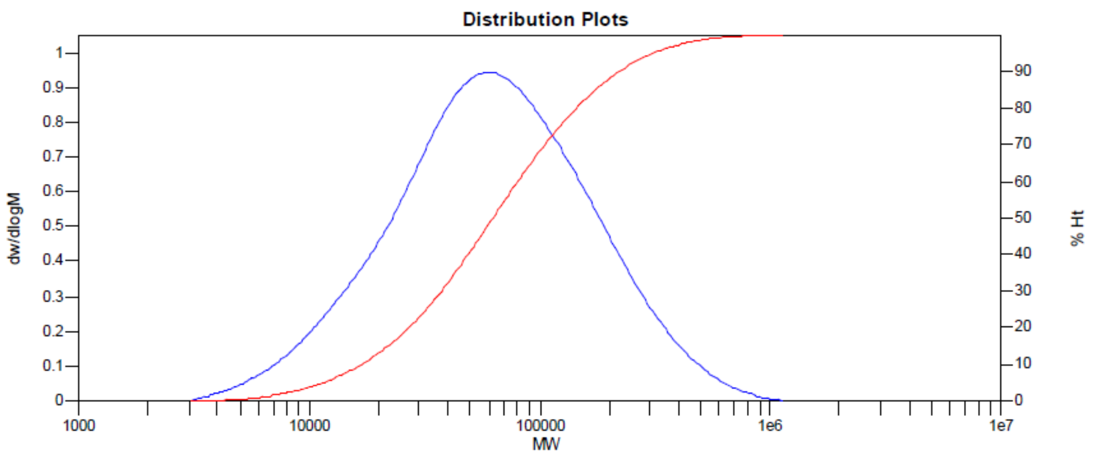

MW Averages

Peak No	Mp	Mn	Mw	Mz	Mz+1	Mv	PD
1	61249	35972	93355	207122	357449	81249	2.59521

Processed Peaks

Peak No	Name	Start RT (mins)	Max RT (mins)	End RT (mins)	Pk Height (mV)	% Height	Area (mV.secs)	% Area
1		12.07	13.97	16.20	-61.9313	100	6129.86	100

Supplementary Figure 153. GPC data of the polymer from table 1, entry 2.

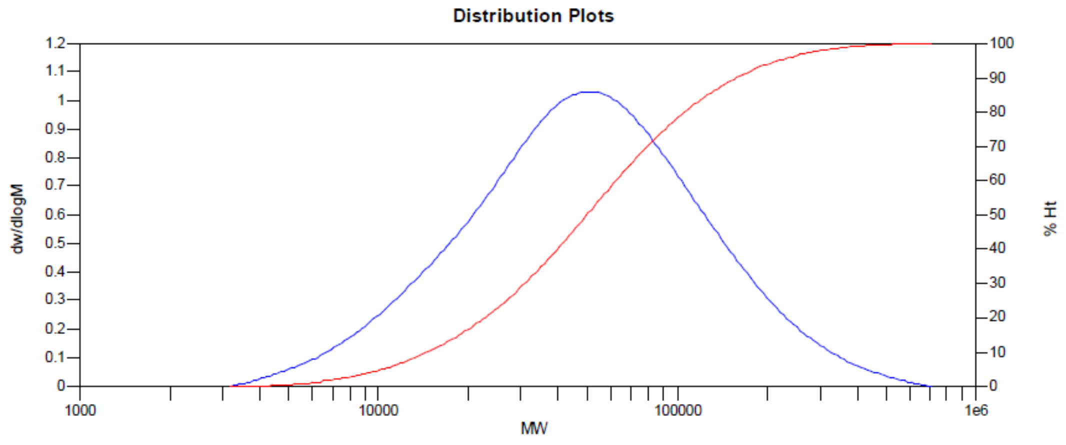

MW Averages

Peak No	Mp	Mn	Mw	Mz	Mz+1	Mv	PD
1	52857	30581	69741	142496	238847	61747	2.28053

Processed Peaks

Peak No	Name	Start RT (mins)	Max RT (mins)	End RT (mins)	Pk Height (mV)	% Height	Area (mV.secs)	% Area
1		12.37	14.07	16.17	-57.9537	100	5286.81	100

Supplementary Figure 154. GPC data of the polymer from table 1, entry 3.

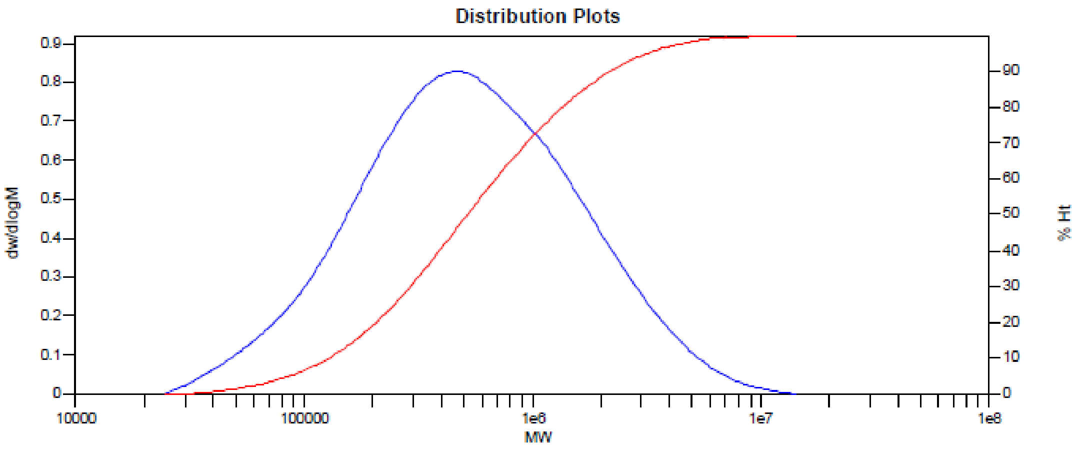

MW Averages

Peak No	Mp	Mn	Mw	Mz	Mz+1	Mv	PD
1	467605	289365	907691	2377431	4394429	764720	3.13684

Processed Peaks

Peak No	Name	Start RT (mins)	Max RT (mins)	End RT (mins)	Pk Height (mV)	% Height	Area (mV.secs)	% Area
1		10.43	12.63	14.60	-58.4578	100	6254.81	100

Supplementary Figure 155. GPC data of the polymer from table 1, entry 4.

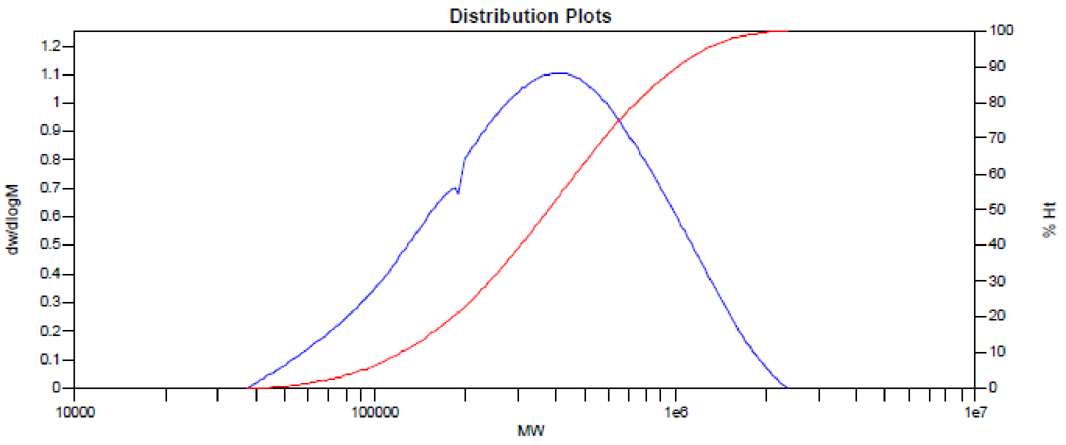

MW Averages

Peak No	Mp	Mn	Mw	Mz	Mz+1	Mv	PD
1	400208	256679	476493	761908	1035941	437678	1.85638

Processed Peaks

Peak No	Name	Start RT (mins)	Max RT (mins)	End RT (mins)	Pk Height (mV)	% Height	Area (mV.secs)	% Area
1		11.60	12.73	14.30	-29.5701	100	2377.09	100

Supplementary Figure 156. GPC data of the polymer from table 1, entry 5.

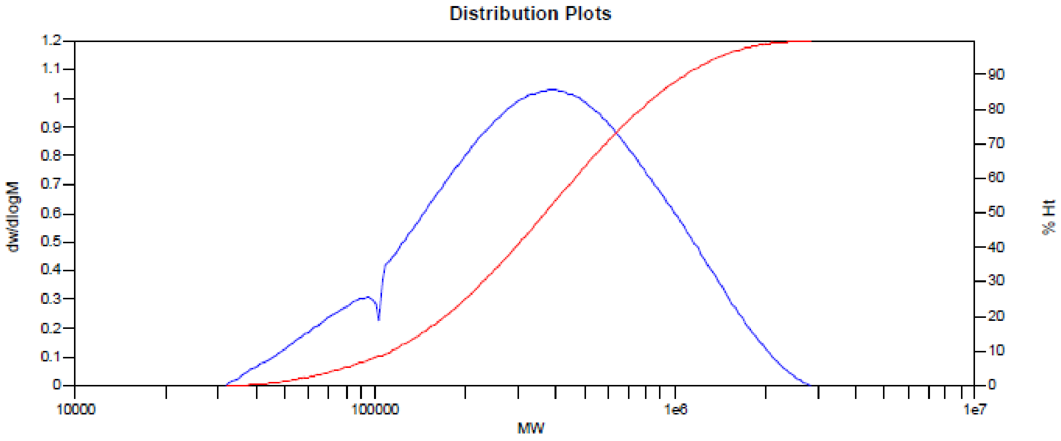

MW Averages

Peak No	Mp	Mn	Mw	Mz	Mz+1	Mv	PD
1	389973	238617	488000	836003	1179095	442392	2.04512

Processed Peaks

Peak No	Name	Start RT (mins)	Max RT (mins)	End RT (mins)	Pk Height (mV)	% Height	Area (mV.secs)	% Area
1		11.48	12.75	14.42	-15.4029	100	1330.58	100

Supplementary Figure 157. GPC data of the polymer from table 1, entry 6.

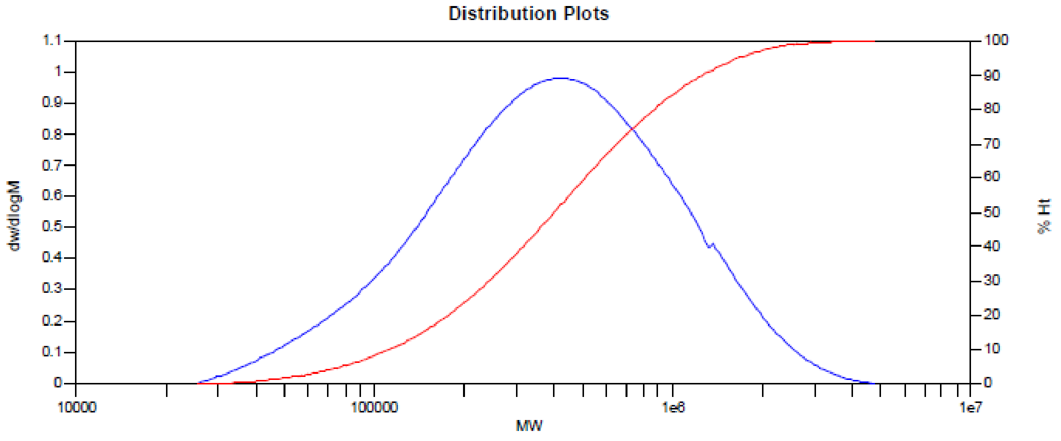

MW Averages

Peak No	Mp	Mn	Mw	Mz	Mz+1	Mv	PD
1	432578	245538	560234	1056270	1609888	499851	2.28166

Processed Peaks

Peak No	Name	Start RT (mins)	Max RT (mins)	End RT (mins)	Pk Height (mV)	% Height	Area (mV.secs)	% Area
1		11.15	12.70	14.57	-63.3531	100	5737.63	100

Supplementary Figure 158. GPC data of the polymer from table 1, entry 7.

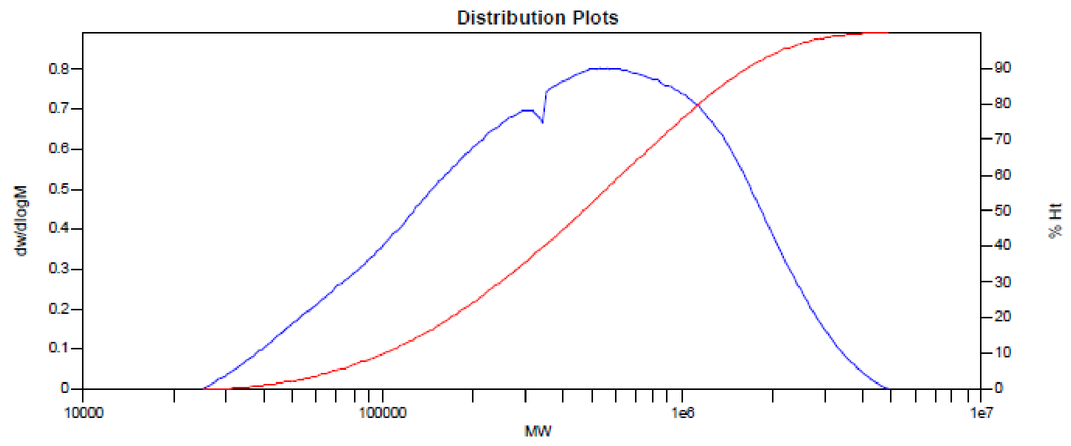

MW Averages

Peak No	Mp	Mn	Mw	Mz	Mz+1	Mv	PD
1	546521	241474	688446	1361574	1974837	601683	2.85102

Processed Peaks

Peak No	Name	Start RT (mins)	Max RT (mins)	End RT (mins)	Pk Height (mV)	% Height	Area (mV.secs)	% Area
1		11.13	12.53	14.58	-23.2646	100	2568.55	100

Supplementary Figure 159. GPC data of the polymer from table 1, entry 8.

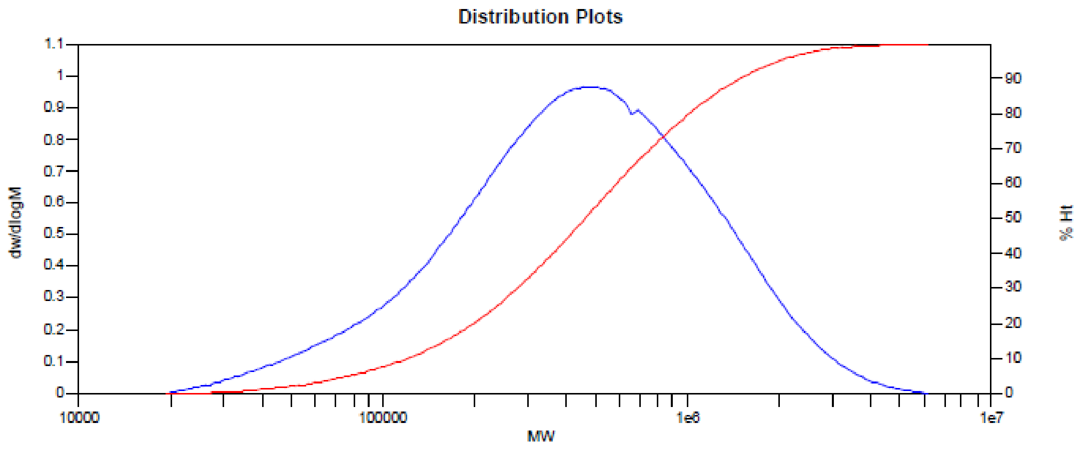

MW Averages

Peak No	Mp	Mn	Mw	Mz	Mz+1	Mv	PD
1	479909	258612	652632	1276471	2000282	577954	2.5236

Processed Peaks

Peak No	Name	Start RT (mins)	Max RT (mins)	End RT (mins)	Pk Height (mV)	% Height	Area (mV.secs)	% Area
1		10.98	12.62	14.77	-65.5327	100	6000.19	100

Supplementary Figure 160.. GPC data of the polymer from table 1, entry 9.

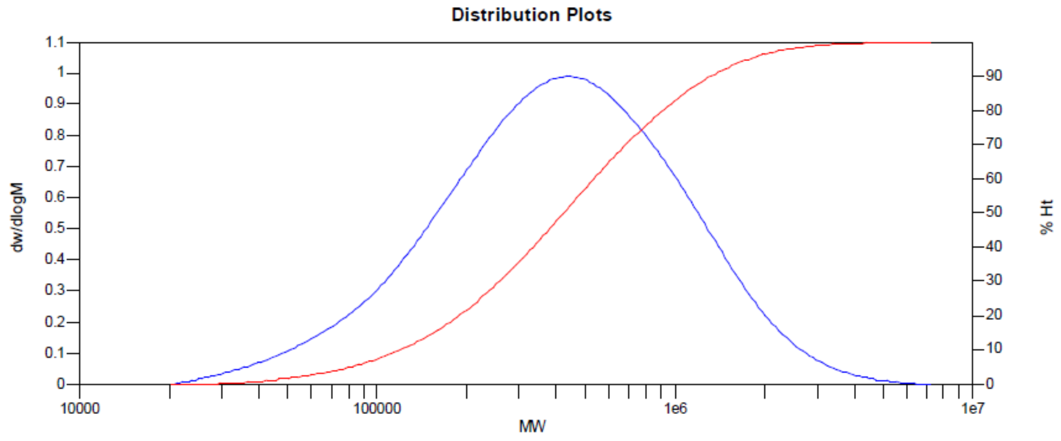

MW Averages

Peak No	Mp	Mn	Mw	Mz	Mz+1	Mv	PD
1	443948	252141	594859	1181454	1968601	528127	2.35923

Processed Peaks

Peak No	Name	Start RT (mins)	Max RT (mins)	End RT (mins)	Pk Height (mV)	% Height	Area (mV.secs)	% Area
1		10.88	12.67	14.73	-75.2575	100	6743.16	100

Supplementary Figure 161. GPC data of the polymer from table 1, entry 10.

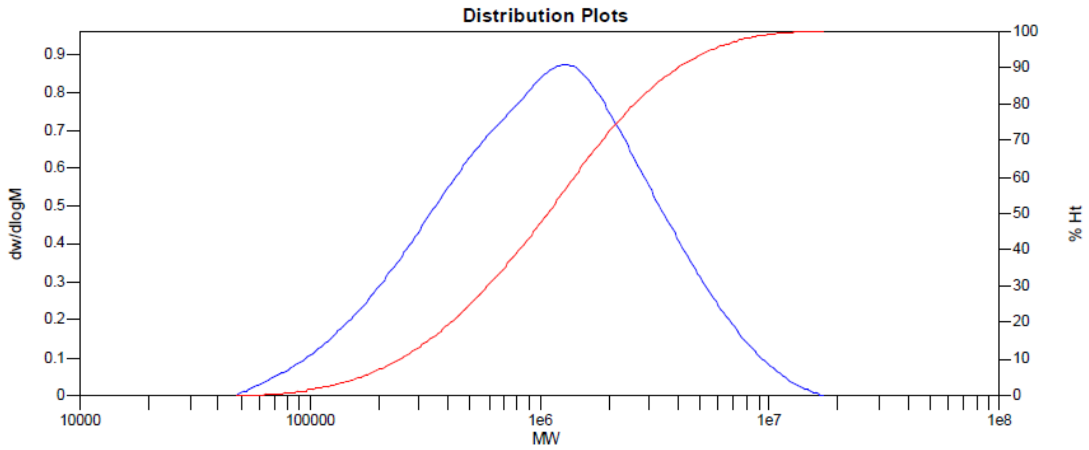

MW Averages

Peak No	Mp	Mn	Mw	Mz	Mz+1	Mv	PD
1	1293188	573382	1697619	3805119	6228635	1462666	2.96071

Processed Peaks

Peak No	Name	Start RT (mins)	Max RT (mins)	End RT (mins)	Pk Height (mV)	% Height	Area (mV.secs)	% Area
1		10.32	11.98	14.13	-41.3454	100	4175.22	100

Supplementary Figure 162. GPC data of the polymer from table 1, entry 11.

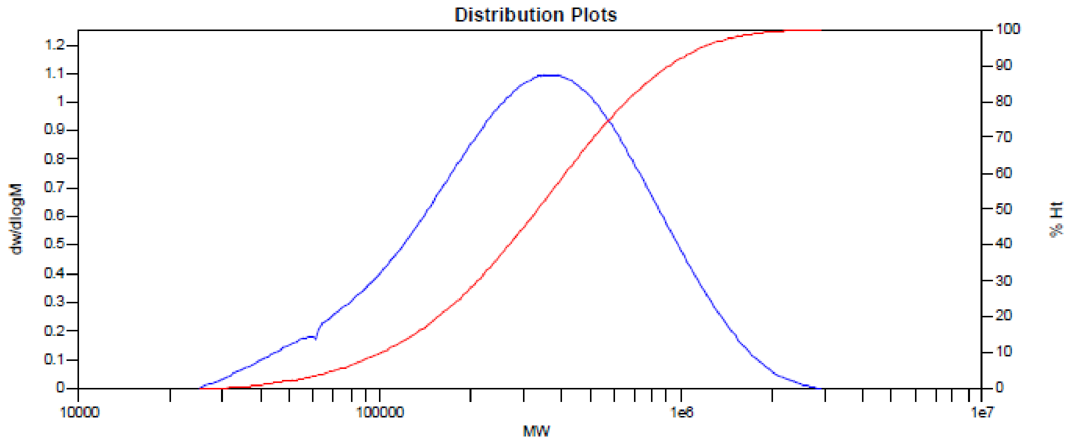

MW Averages

Peak No	Mp	Mn	Mw	Mz	Mz+1	Mv	PD
1	370293	213665	427326	718057	1027470	389364	1.99998

Processed Peaks

Peak No	Name	Start RT (mins)	Max RT (mins)	End RT (mins)	Pk Height (mV)	% Height	Area (mV.secs)	% Area
1		11.47	12.78	14.58	-58.2339	100	4731.01	100

Supplementary Figure 163. GPC data of the polymer from table 1, entry 12.

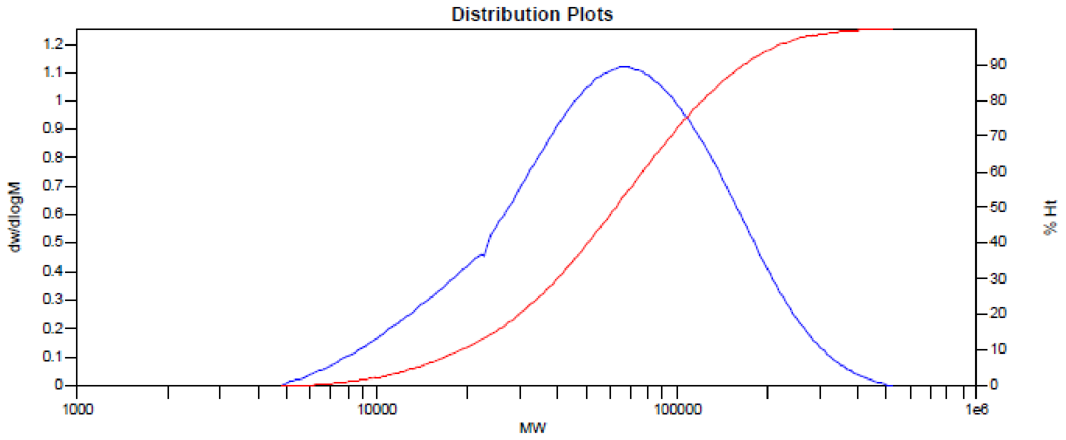

MW Averages

Peak No	Mp	Mn	Mw	Mz	Mz+1	Mv	PD
1	69299	39705	78061	129921	185612	71273	1.96602

Processed Peaks

Peak No	Name	Start RT (mins)	Max RT (mins)	End RT (mins)	Pk Height (mV)	% Height	Area (mV.secs)	% Area
1		12.57	13.88	15.82	-51.2292	100	4271.94	100

Supplementary Figure 164. GPC data of the polymer from table 1, entry 13.

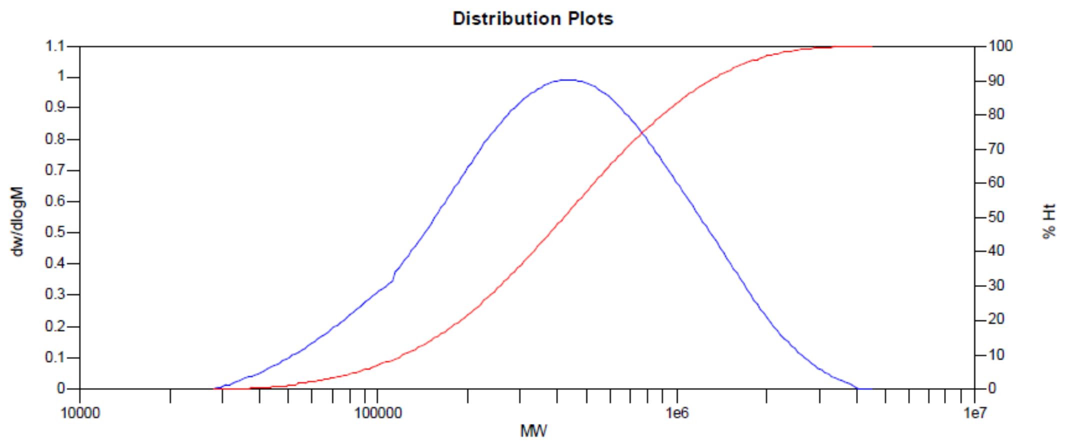

MW Averages

Peak No	Mp	Mn	Mw	Mz	Mz+1	Mv	PD
1	443948	262704	577558	1062579	1585875	517564	2.19851

Processed Peaks

Peak No	Name	Start RT (mins)	Max RT (mins)	End RT (mins)	Pk Height (mV)	% Height	Area (mV.secs)	% Area
1		11.18	12.68	14.50	-55.4035	100	4950.87	100

Supplementary Figure 165. GPC data of the polymer from table 1, entry 14.

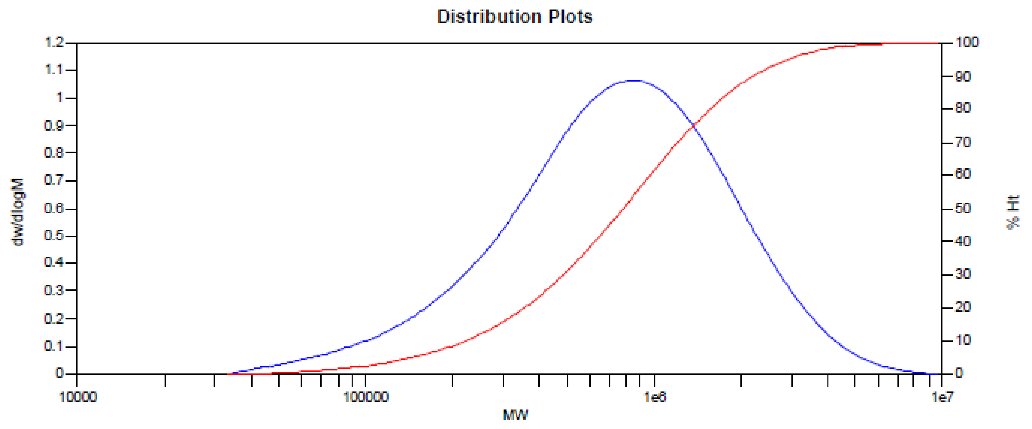

MW Averages

Peak No	Mp	Mn	Mw	Mz	Mz+1	Mv	PD
1	829327	461150	1035131	1860823	2851496	932948	2.24467

Processed Peaks

Peak No	Name	Start RT (mins)	Max RT (mins)	End RT (mins)	Pk Height (mV)	% Height	Area (mV.secs)	% Area
1		10.70	12.27	14.38	-60.1787	100	4987.77	100

Supplementary Figure 166. GPC data of the polymer from table 1, entry 15.

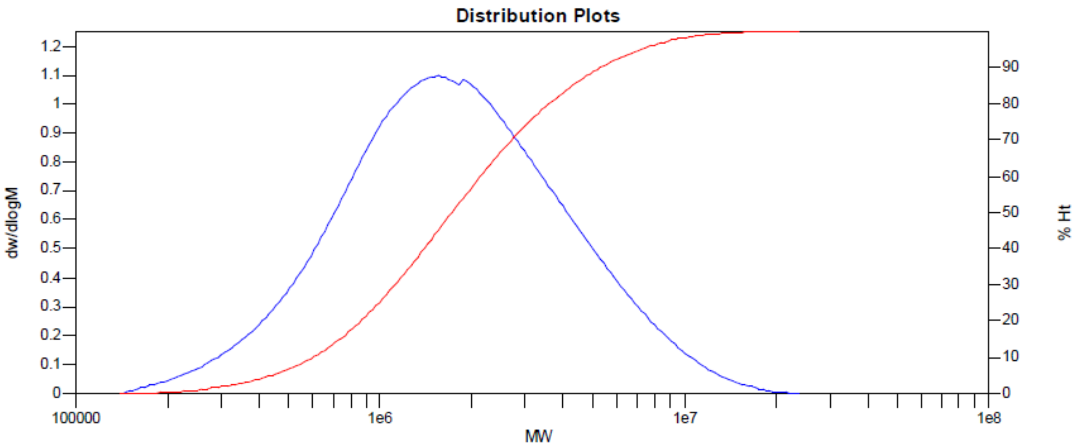

MW Averages

Peak No	Mp	Mn	Mw	Mz	Mz+1	Mv	PD
1	1552947	1226481	2443075	4590118	7337339	2198268	1.99194

Processed Peaks

Peak No	Name	Start RT (mins)	Max RT (mins)	End RT (mins)	Pk Height (mV)	% Height	Area (mV.secs)	% Area
1		10.10	11.87	13.42	-52.0267	100	4177.63	100

Supplementary Figure 167. GPC data of the polymer from table 1, entry 16.

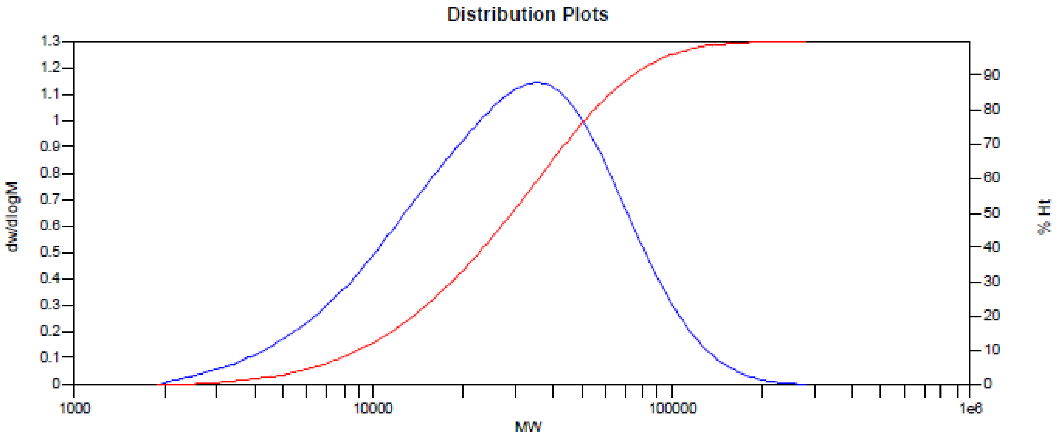

MW Averages

Peak No	Mp	Mn	Mw	Mz	Mz+1	Mv	PD
1	35849	17929	35028	57307	81954	32079	1.95371

Processed Peaks

Peak No	Name	Start RT (mins)	Max RT (mins)	End RT (mins)	Pk Height (mV)	% Height	Area (mV.secs)	% Area
1		12.97	14.33	16.62	-51.1642	100	4280.32	100

Supplementary Figure 168. GPC data of the polymer from table 2, entry 1.

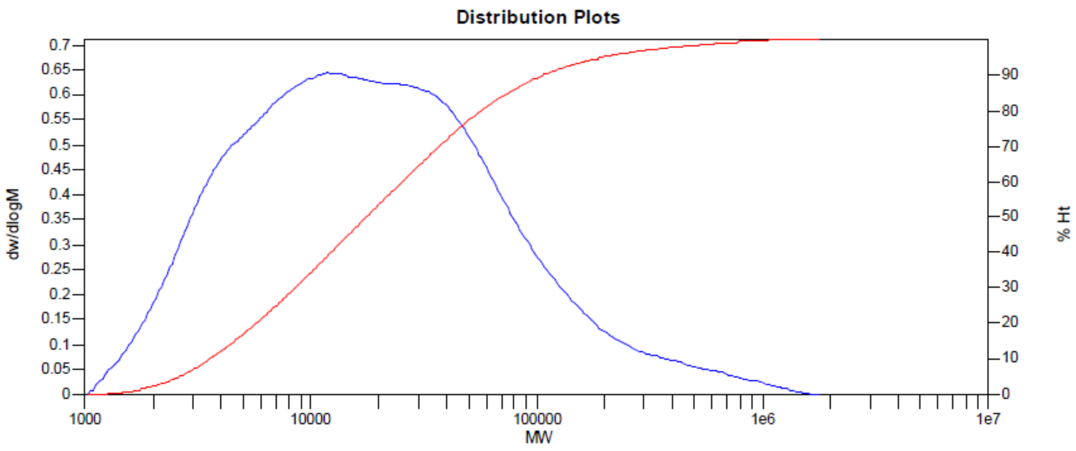

MW Averages

Peak No	Mp	Mn	Mw	Mz	Mz+1	Mv	PD
1	28230	8321	45022	282608	667706	32560	5.41065

Processed Peaks

Peak No	Name	Start RT (mins)	Max RT (mins)	End RT (mins)	Pk Height (mV)	% Height	Area (mV.secs)	% Area
1		11.78	14.92	17.22	-18.3609	100	2904.85	100

Supplementary Figure 169. GPC data of the polymer from table 2, entry 2.

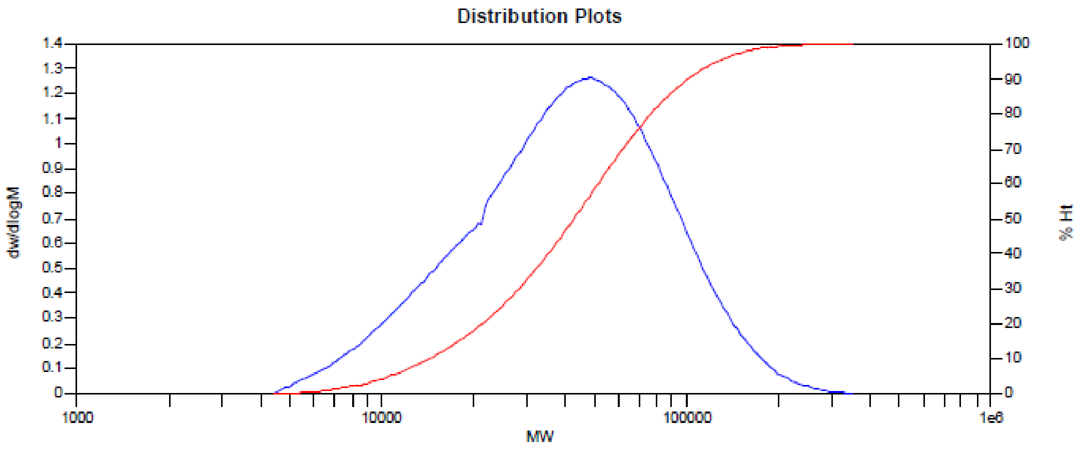

MW Averages

Peak No	Mp	Mn	Mw	Mz	Mz+1	Mv	PD
1	49120	29095	50346	77780	108264	46677	1.7304

Processed Peaks

Peak No	Name	Start RT (mins)	Max RT (mins)	End RT (mins)	Pk Height (mV)	% Height	Area (mV.secs)	% Area
1		12.82	14.12	15.88	-34.5145	100	2583.97	100

Supplementary Figure 170. GPC data of the polymer from table 2, entry 3.

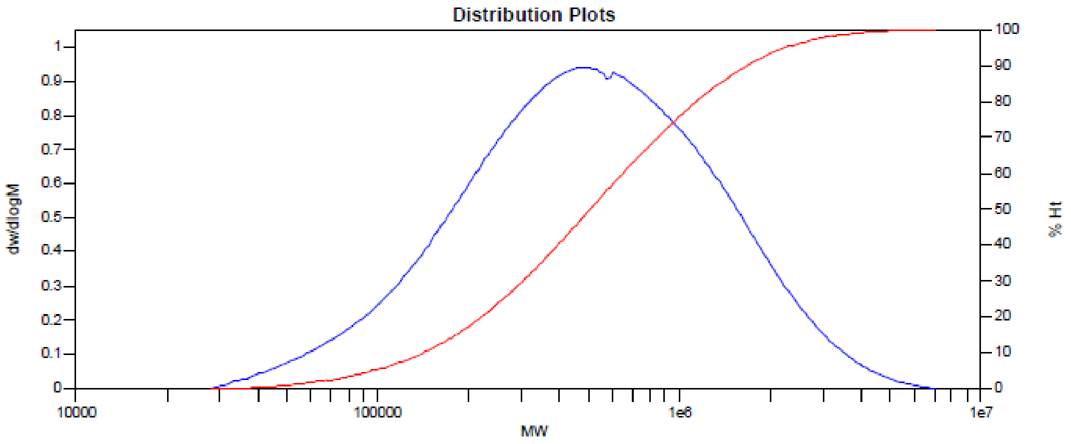

MW Averages

Peak No	Mp	Mn	Mw	Mz	Mz+1	Mv	PD
1	479909	306745	736796	1463801	2315618	651657	2.40198

Processed Peaks

Peak No	Name	Start RT (mins)	Max RT (mins)	End RT (mins)	Pk Height (mV)	% Height	Area (mV.secs)	% Area
1		10.90	12.62	14.50	-70.933	100	6694.67	100

Supplementary Figure 171. GPC data of the polymer from table 2, entry 4.

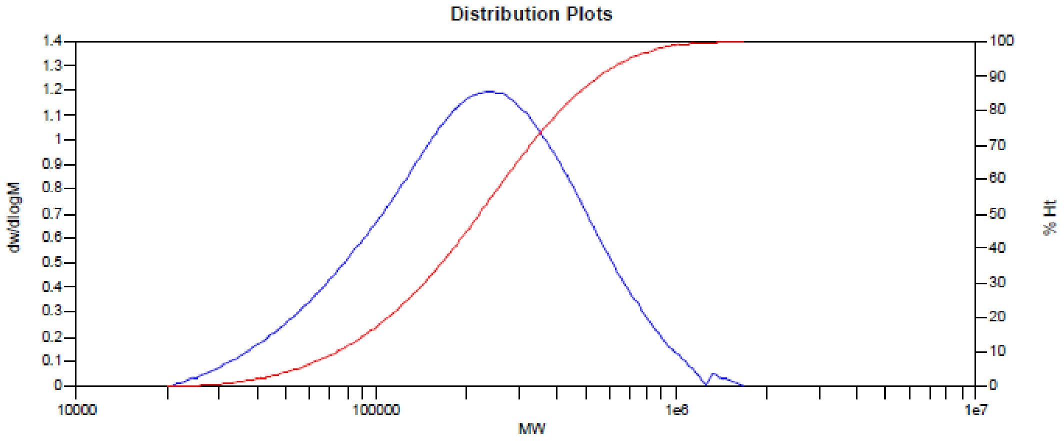

MW Averages

Peak No	Mp	Mn	Mw	Mz	Mz+1	Mv	PD
1	238884	151872	271566	430767	604896	250533	1.78812

Processed Peaks

Peak No	Name	Start RT (mins)	Max RT (mins)	End RT (mins)	Pk Height (mV)	% Height	Area (mV.secs)	% Area
1		11.82	13.07	14.73	-41.7812	100	3131.29	100

Supplementary Figure 172. GPC data of the polymer from table 2, entry 5.

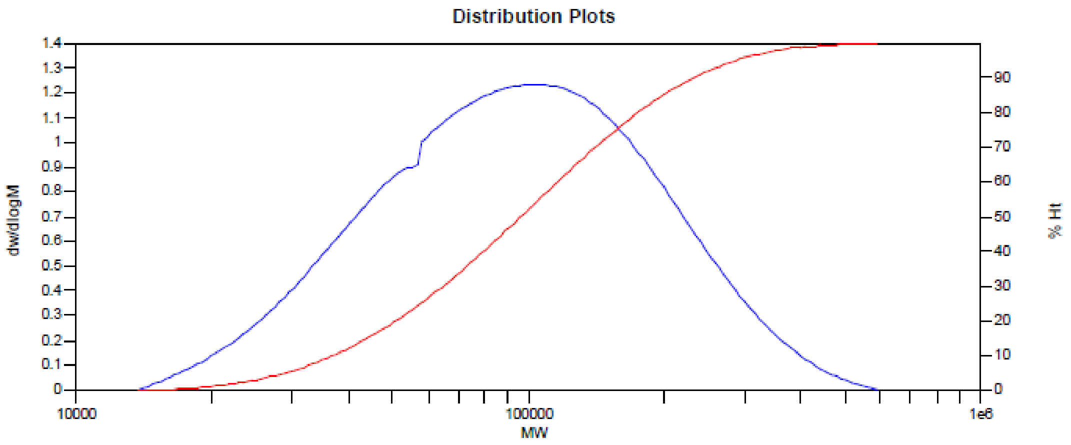

MW Averages

Peak No	Mp	Mn	Mw	Mz	Mz+1	Mv	PD
1	105905	72198	115253	172028	231921	107611	1.59635

Processed Peaks

Peak No	Name	Start RT (mins)	Max RT (mins)	End RT (mins)	Pk Height (mV)	% Height	Area (mV.secs)	% Area
1		12.48	13.60	15.02	-36.4705	100	2701.03	100

Supplementary Figure 173. GPC data of the polymer from table 2, entry 6.

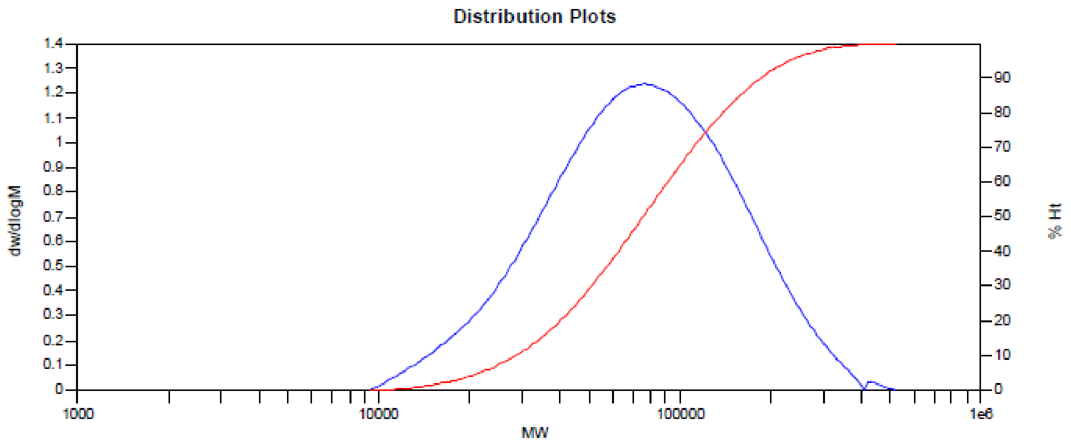

MW Averages

Peak No	Mp	Mn	Mw	Mz	Mz+1	Mv	PD
1	76527	55453	91341	138766	189374	85000	1.64718

Processed Peaks

Peak No	Name	Start RT (mins)	Max RT (mins)	End RT (mins)	Pk Height (mV)	% Height	Area (mV.secs)	% Area
1		12.57	13.82	15.30	-49.1791	100	3679.87	100

Supplementary Figure 174. GPC data of the polymer from table 2, entry 7.

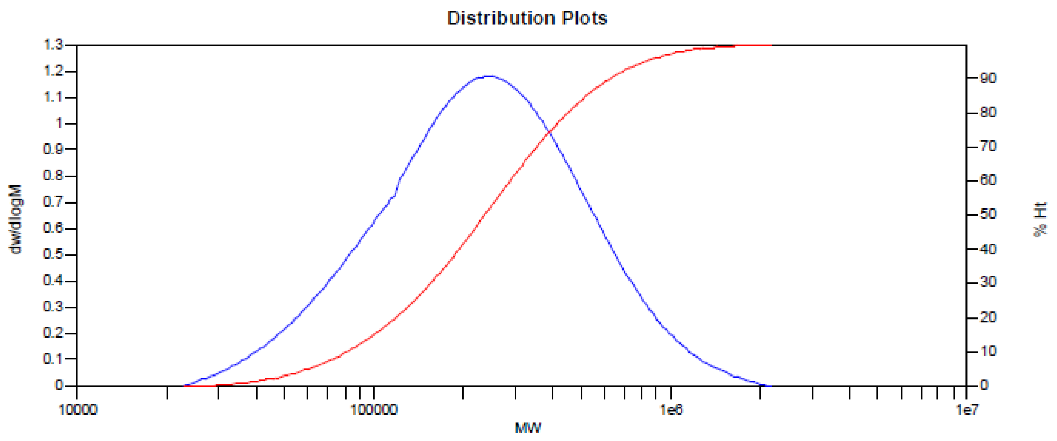

MW Averages

Peak No	Mp	Mn	Mw	Mz	Mz+1	Mv	PD
1	245099	165242	300887	505049	752965	275669	1.82089

Processed Peaks

Peak No	Name	Start RT (mins)	Max RT (mins)	End RT (mins)	Pk Height (mV)	% Height	Area (mV.secs)	% Area
1		11.65	13.05	14.65	-56.545	100	4290.94	100

Supplementary Figure 175. GPC data of the polymer from table 2, entry 8.

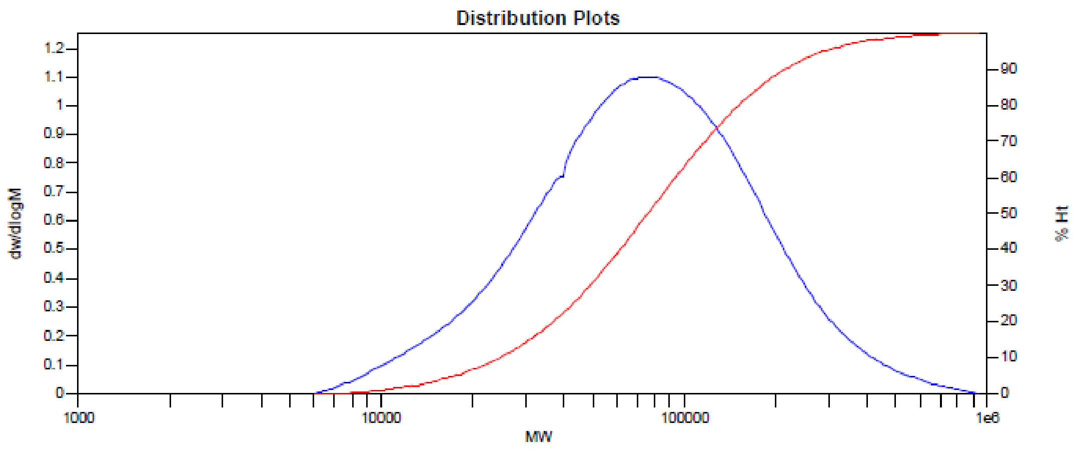

MW Averages

Peak No	Mp	Mn	Mw	Mz	Mz+1	Mv	PD
1	77146	50112	100888	187590	306118	91305	2.01325

Processed Peaks

Peak No	Name	Start RT (mins)	Max RT (mins)	End RT (mins)	Pk Height (mV)	% Height	Area (mV.secs)	% Area
1		12.13	13.77	15.60	-55.3346	100	4675.97	100

Supplementary Figure 176. GPC data of the polymer from table 2, entry 9.

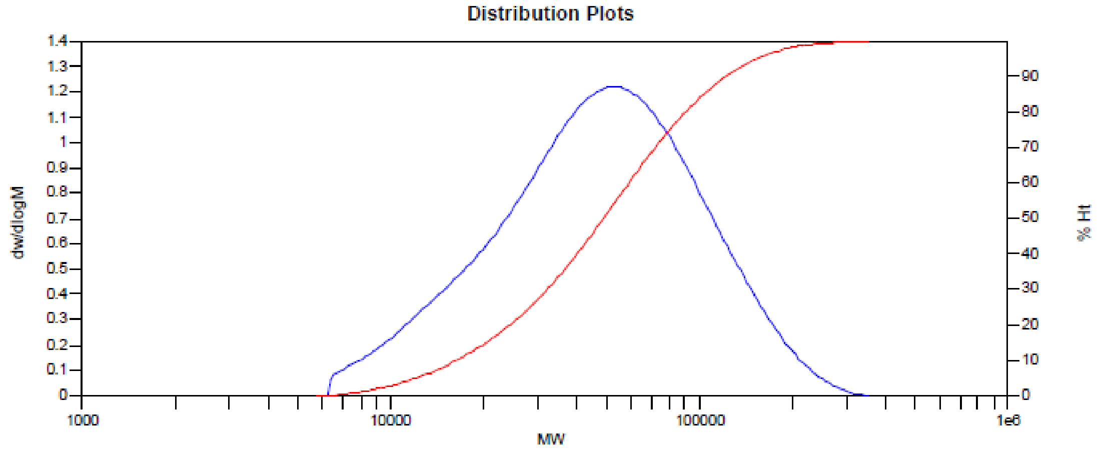

MW Averages

Peak No	Mp	Mn	Mw	Mz	Mz+1	Mv	PD
1	55513	33360	58690	91875	126941	54247	1.75929

Processed Peaks

Peak No	Name	Start RT (mins)	Max RT (mins)	End RT (mins)	Pk Height (mV)	% Height	Area (mV.secs)	% Area
1		12.82	14.03	15.68	-40.7564	100	3140.3	100

Supplementary Figure 177. GPC data of the polymer from table 2, entry 10.

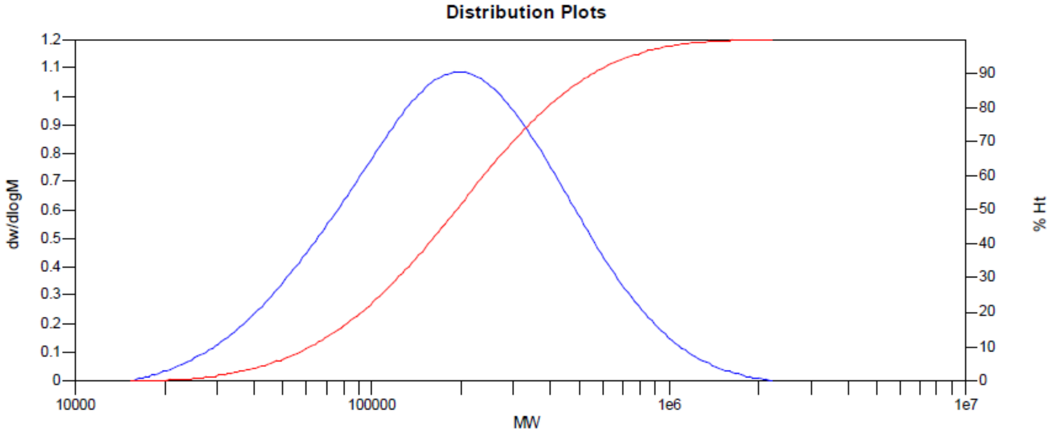

MW Averages

Peak No	Mp	Mn	Mw	Mz	Mz+1	Mv	PD
1	199657	128559	256623	467553	733062	231755	1.99615

Processed Peaks

Peak No	Name	Start RT (mins)	Max RT (mins)	End RT (mins)	Pk Height (mV)	% Height	Area (mV.secs)	% Area
1		11.63	13.18	14.93	-50.7744	100	4209.89	100

Supplementary Figure 178. GPC data of the polymer from table 3, entry 1.

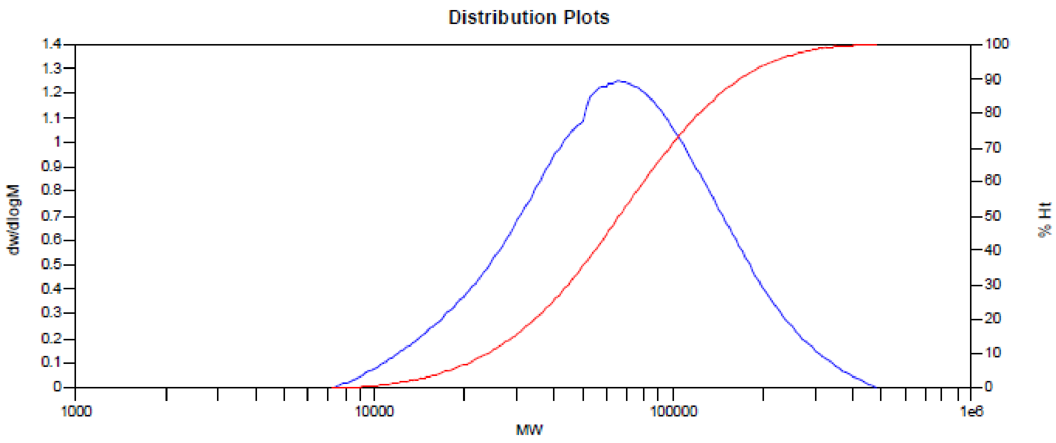

MW Averages

Peak No	Mp	Mn	Mw	Mz	Mz+1	Mv	PD
1	65955	47354	81800	130516	185823	75552	1.72741

Processed Peaks

Peak No	Name	Start RT (mins)	Max RT (mins)	End RT (mins)	Pk Height (mV)	% Height	Area (mV.secs)	% Area
1		12.62	13.90	15.50	-43.0802	100	3212.58	100

Supplementary Figure 179. GPC data of the polymer from table 3, entry 2.

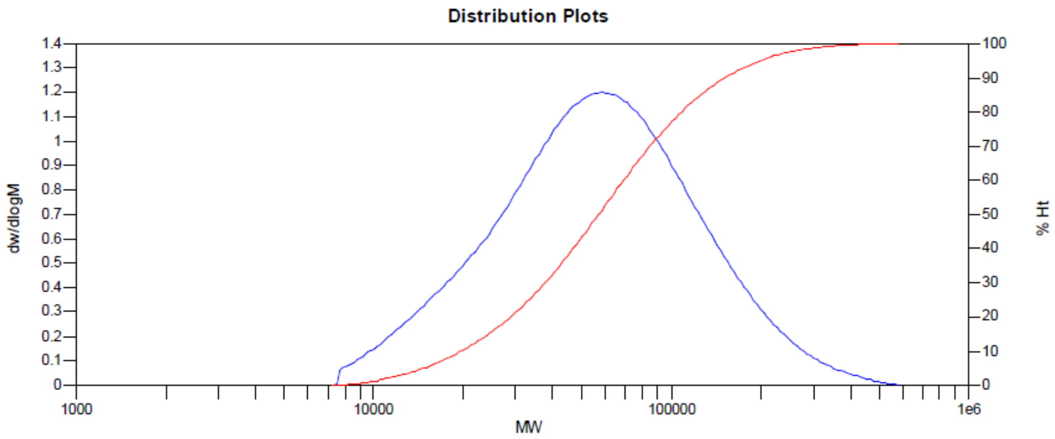

MW Averages

Peak No	Mp	Mn	Mw	Mz	Mz+1	Mv	PD
1	59760	39974	72490	124375	191158	66278	1.81343

Processed Peaks

Peak No	Name	Start RT (mins)	Max RT (mins)	End RT (mins)	Pk Height (mV)	% Height	Area (mV.secs)	% Area
1		12.50	13.98	15.50	-54.4092	100	4244.24	100

Supplementary Figure 180. GPC data of the polymer from table 3, entry 3.

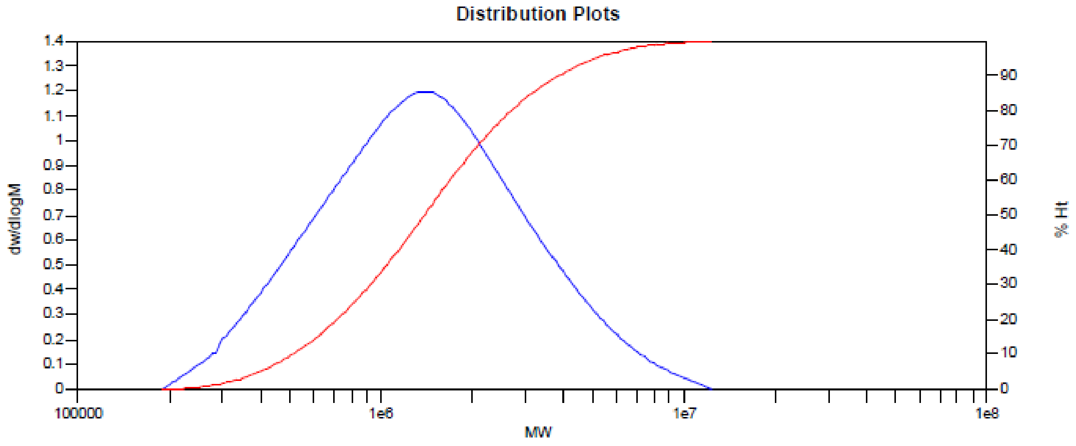

MW Averages

Peak No	Mp	Mn	Mw	Mz	Mz+1	Mv	PD
1	1398720	1042860	1844057	3156242	4753884	1686830	1.76827

Processed Peaks

Peak No	Name	Start RT (mins)	Max RT (mins)	End RT (mins)	Pk Height (mV)	% Height	Area (mV.secs)	% Area
1		10.53	11.95	13.22	-55.3326	100	4065.61	100

Supplementary Figure 181. GPC data of the polymer from table 3, entry 4.

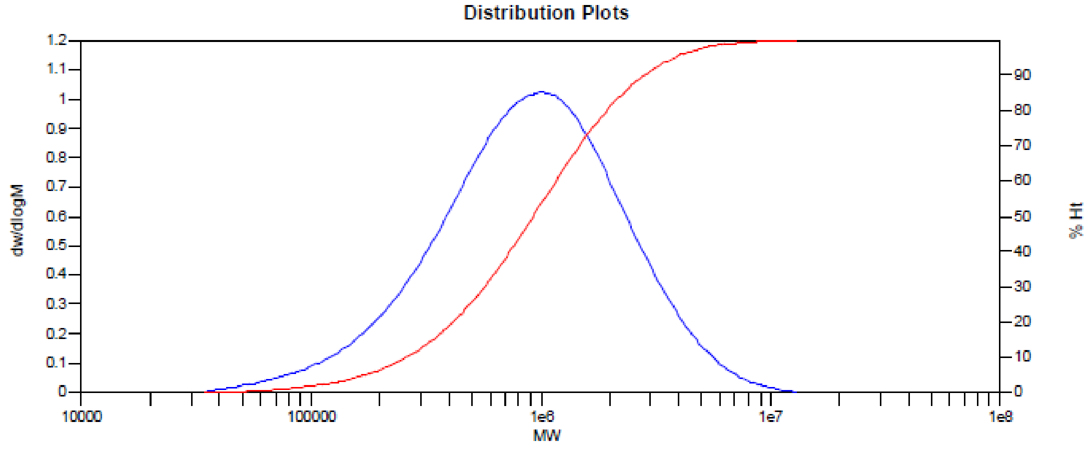

MW Averages

Peak No	Mp	Mn	Mw	Mz	Mz+1	Mv	PD
1	995707	544100	1287709	2488953	4029086	1147943	2.36668

Processed Peaks

Peak No	Name	Start RT (mins)	Max RT (mins)	End RT (mins)	Pk Height (mV)	% Height	Area (mV.secs)	% Area
1		10.50	12.15	14.37	-51.0292	100	4390.96	100

Supplementary Figure 182. GPC data of the polymer from table 3, entry 5.

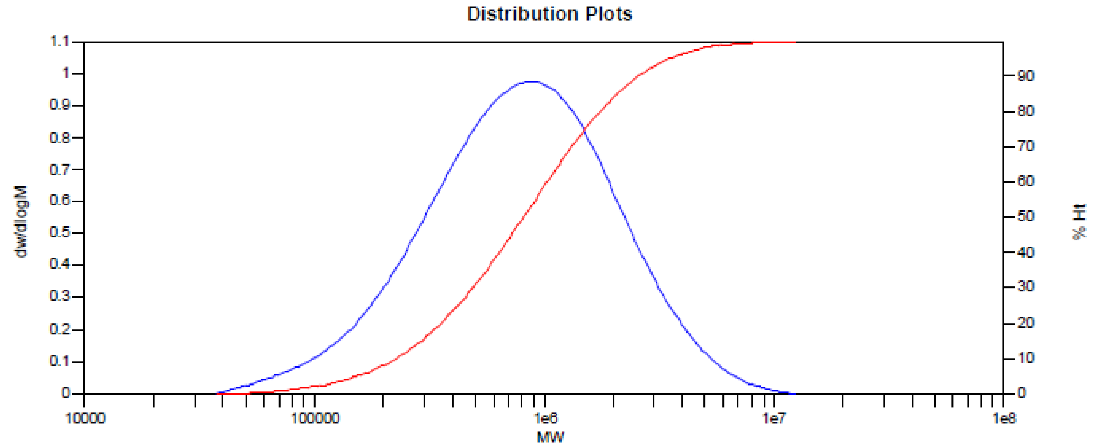

MW Averages

Peak No	Mp	Mn	Mw	Mz	Mz+1	Mv	PD
1	896908	482202	1154636	2304401	3774881	1023299	2.39451

Processed Peaks

Peak No	Name	Start RT (mins)	Max RT (mins)	End RT (mins)	Pk Height (mV)	% Height	Area (mV.secs)	% Area
1		10.53	12.23	14.30	-47.9813	100	4333.47	100

Supplementary Figure 183. GPC data of the polymer from table 3, entry 6.

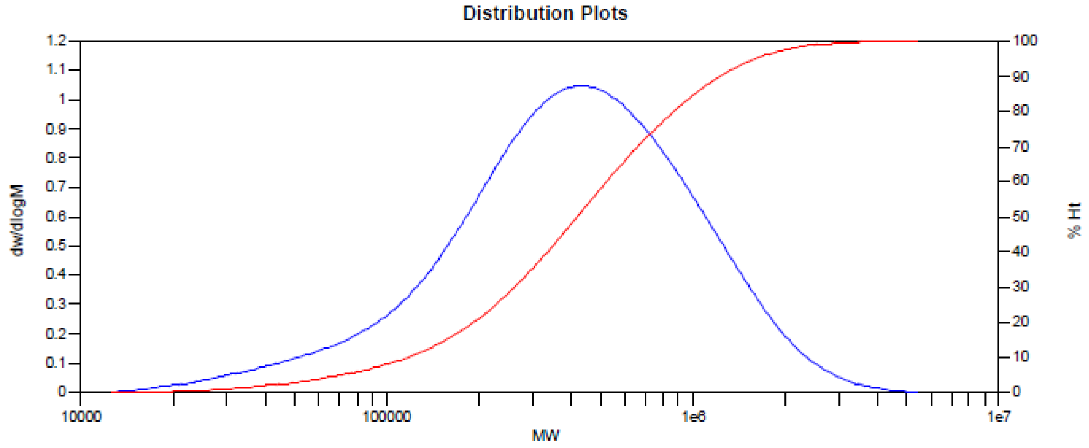

MW Averages

Peak No	Mp	Mn	Mw	Mz	Mz+1	Mv	PD
1	443948	233530	563419	1031662	1587354	505550	2.41262

Processed Peaks

Peak No	Name	Start RT (mins)	Max RT (mins)	End RT (mins)	Pk Height (mV)	% Height	Area (mV.secs)	% Area
1		11.07	12.68	15.08	-56.3494	100	4781.73	100

Supplementary Figure 184. GPC data of the polymer from table 3, entry 7.

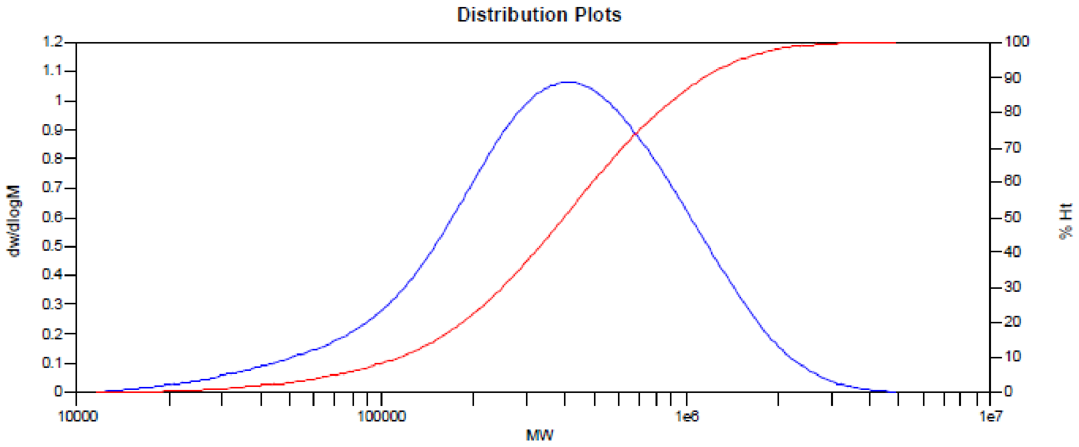

MW Averages

Peak No	Mp	Mn	Mw	Mz	Mz+1	Mv	PD
1	410716	225565	529266	958195	1466992	476145	2.3464

Processed Peaks

Peak No	Name	Start RT (mins)	Max RT (mins)	End RT (mins)	Pk Height (mV)	% Height	Area (mV.secs)	% Area
1		11.13	12.72	15.13	-60.403	100	5043.9	100

Supplementary Figure 185. GPC data of the polymer from table 3, entry 8.

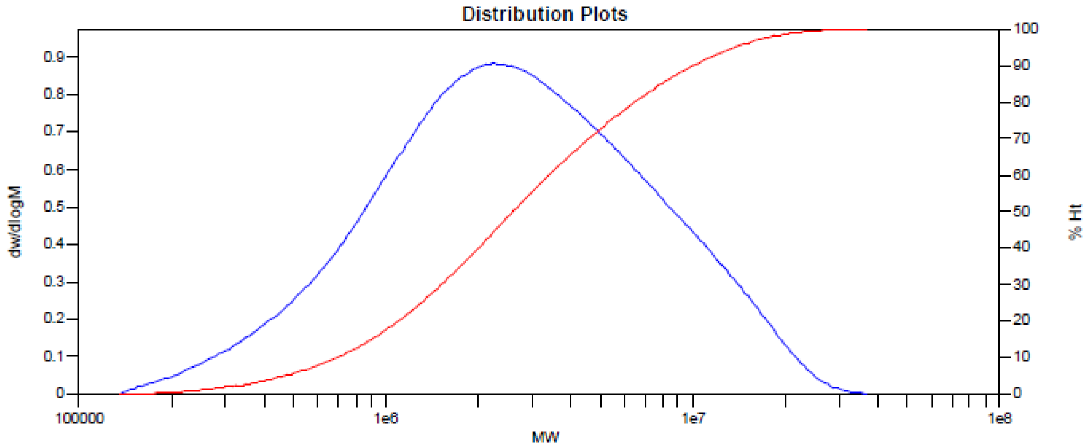

MW Averages

Peak No	Mp	Mn	Mw	Mz	Mz+1	Mv	PD
1	2239094	1556336	4164435	8702165	13271050	3628008	2.67579

Processed Peaks

Peak No	Name	Start RT (mins)	Max RT (mins)	End RT (mins)	Pk Height (mV)	% Height	Area (mV.secs)	% Area
1		9.80	11.65	13.43	23.9626	100	2388.13	100

Supplementary Figure 186. GPC data of the polymer from table 3, entry 9.

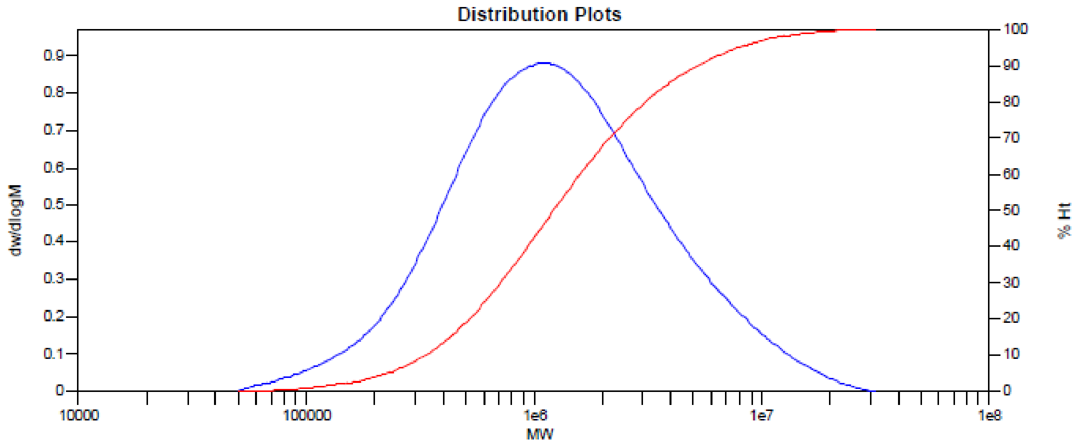

MW Averages

Peak No	Mp	Mn	Mw	Mz	Mz+1	Mv	PD
1	1105438	722370	2191859	5910094	10845823	1843113	3.03426

Processed Peaks

Peak No	Name	Start RT (mins)	Max RT (mins)	End RT (mins)	Pk Height (mV)	% Height	Area (mV.secs)	% Area
1		9.90	12.08	14.10	-46.3928	100	4632.35	100

Supplementary Figure 187. GPC data of the polymer from table 3, entry 10.

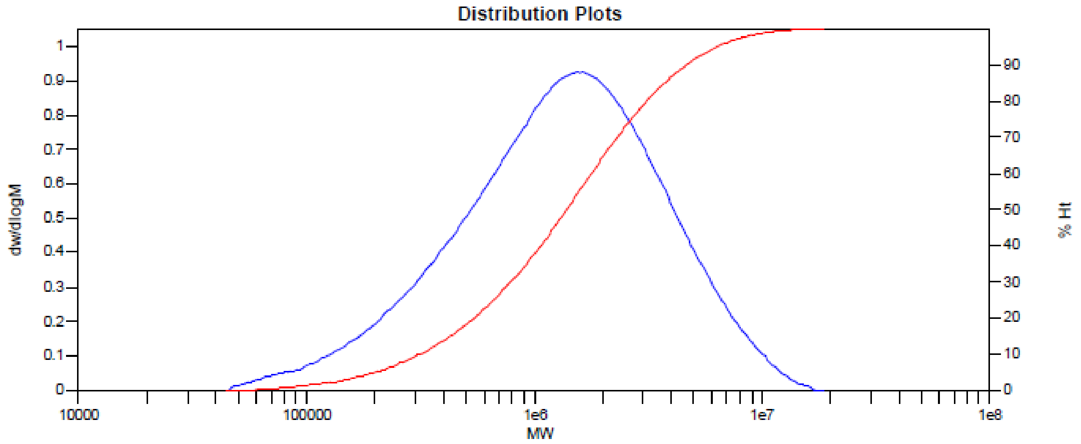

MW Averages

Peak No	Mp	Mn	Mw	Mz	Mz+1	Mv	PD
1	1552947	701313	2022882	4110456	6406931	1772391	2.88442

Processed Peaks

Peak No	Name	Start RT (mins)	Max RT (mins)	End RT (mins)	Pk Height (mV)	% Height	Area (mV.secs)	% Area
1		10.27	11.87	14.18	-16.0966	100	1531.24	100

Supplementary Figure 188. GPC data of the polymer from table 3, entry 11.

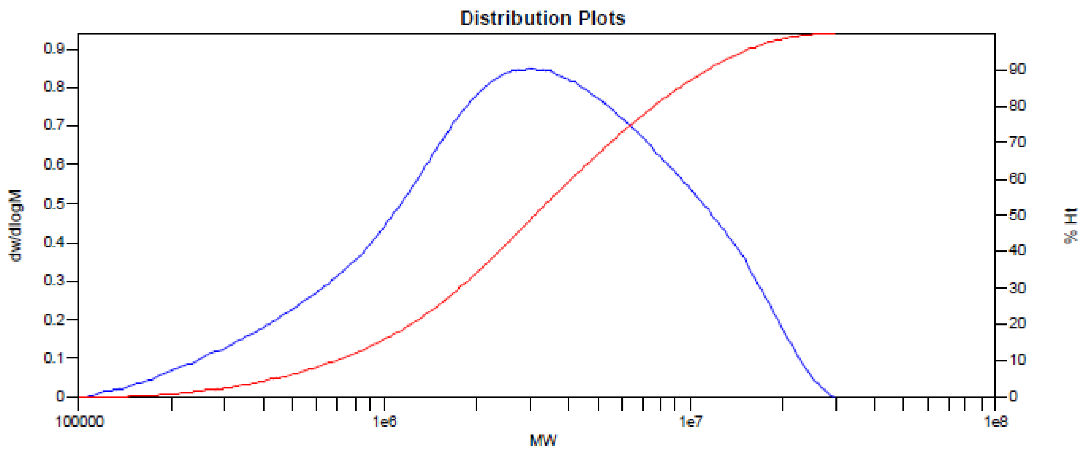

MW Averages

Peak No	Mp	Mn	Mw	Mz	Mz+1	Mv	PD
1	3062362	1593323	4731101	9259732	13203927	4142791	2.96933

Processed Peaks

Peak No	Name	Start RT (mins)	Max RT (mins)	End RT (mins)	Pk Height (mV)	% Height	Area (mV.secs)	% Area
1		9.95	11.43	13.63	16.9115	100	1757.5	100

Supplementary Figure 189. GPC data of the polymer from table 3, entry 12.

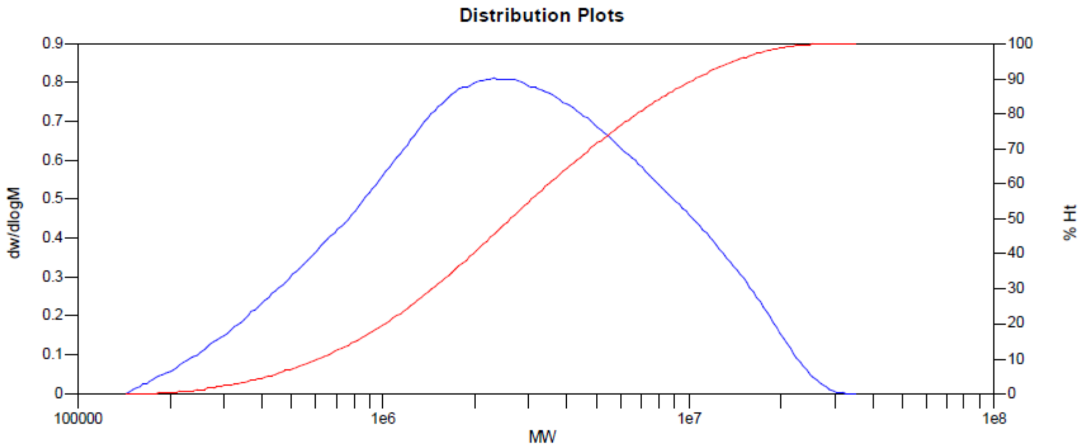

MW Averages

Peak No	Mp	Mn	Mw	Mz	Mz+1	Mv	PD
1	2298343	1464783	4270194	9008538	13344280	3692258	2.91524

Processed Peaks

Peak No	Name	Start RT (mins)	Max RT (mins)	End RT (mins)	Pk Height (mV)	% Height	Area (mV.secs)	% Area
1		9.83	11.68	13.40	16.297	100	1782.77	100

Supplementary Figure 190. GPC data of the polymer from table 3, entry 13.

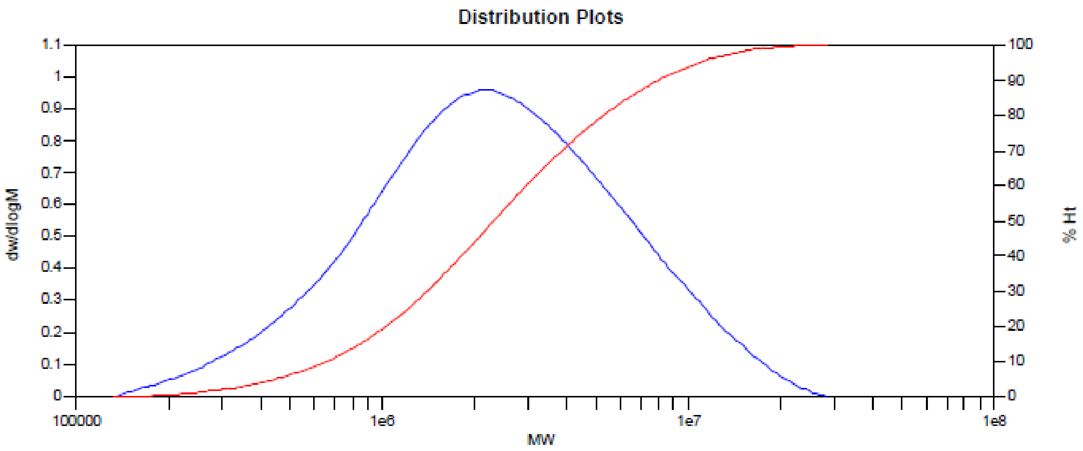

MW Averages

Peak No	Mp	Mn	Mw	Mz	Mz+1	Mv	PD
1	2125117	1443750	3499224	7026930	10900709	3086155	2.4237

Processed Peaks

Peak No	Name	Start RT (mins)	Max RT (mins)	End RT (mins)	Pk Height (mV)	% Height	Area (mV.secs)	% Area
1		9.98	11.67	13.45	21.7707	100	2002.77	100

Supplementary Figure 191. GPC data of the polymer from table 3, entry 14.

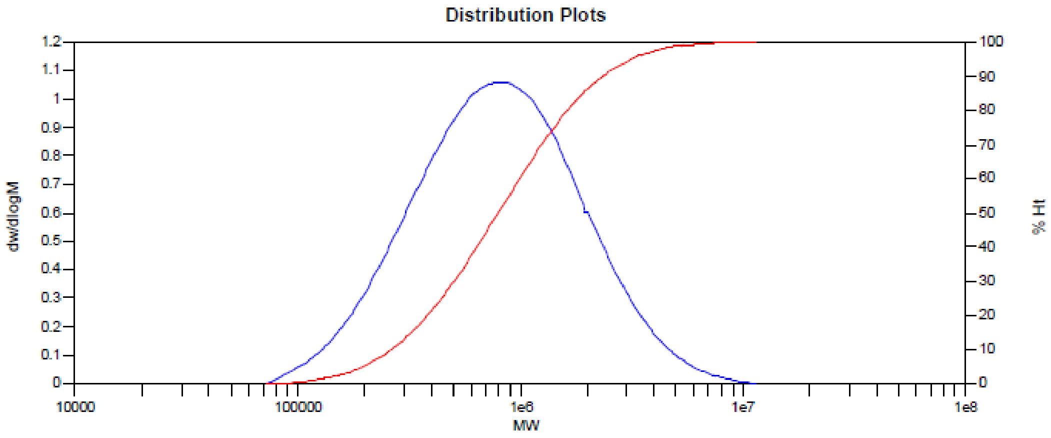

MW Averages

Peak No	Mp	Mn	Mw	Mz	Mz+1	Mv	PD
1	829327	554686	1102926	2077710	3406510	992794	1.98838

Processed Peaks

Peak No	Name	Start RT (mins)	Max RT (mins)	End RT (mins)	Pk Height (mV)	% Height	Area (mV.secs)	% Area
1		10.58	12.30	13.85	-64.4034	100	5363.71	100

Supplementary Figure 192. GPC data of the polymer from supplementary table 1, entry 1.

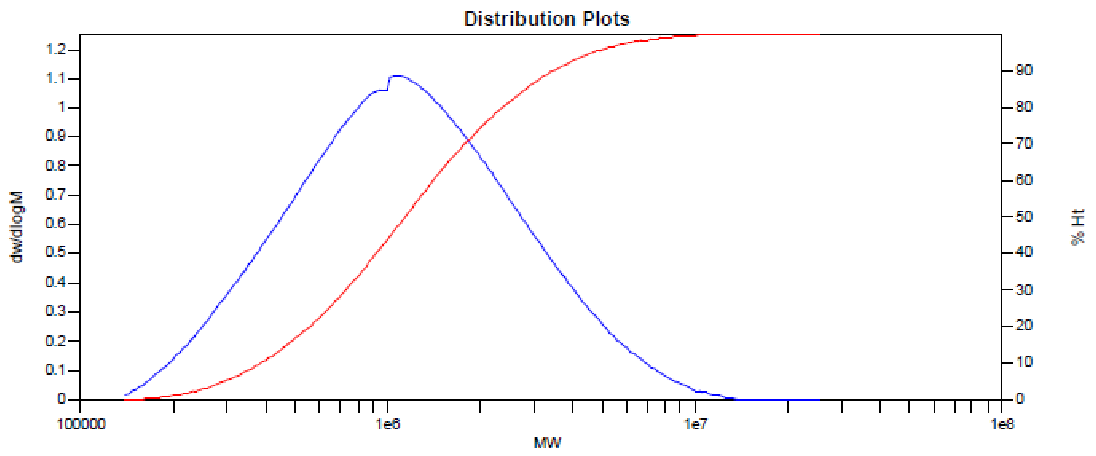

MW Averages

Peak No	Mp	Mn	Mw	Mz	Mz+1	Mv	PD
1	1076917	818811	1598586	2990468	4758841	1439334	1.95233

Processed Peaks

Peak No	Name	Start RT (mins)	Max RT (mins)	End RT (mins)	Pk Height (mV)	% Height	Area (mV.secs)	% Area
1		10.05	12.10	13.42	-45.8181	0	3640.46	100

Supplementary Figure 193. GPC data of the polymer from supplementary table 1, entry 2.

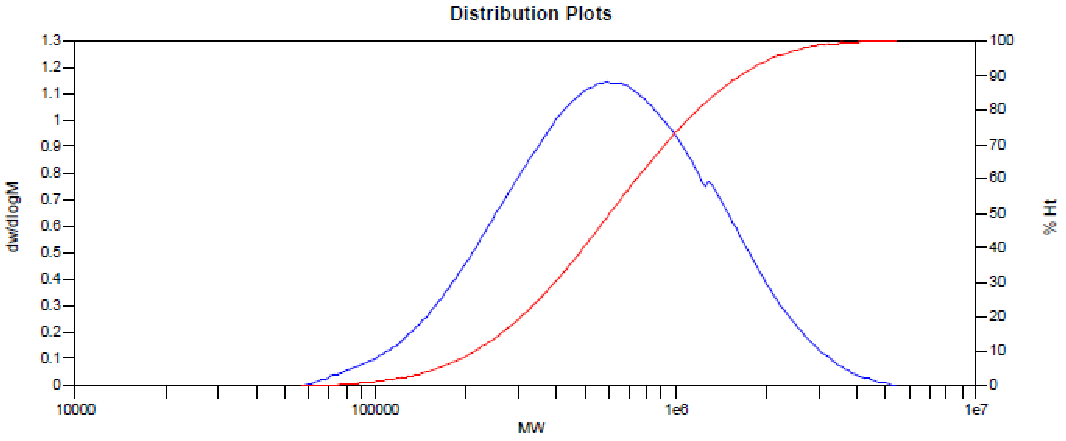

MW Averages

Peak No	Mp	Mn	Mw	Mz	Mz+1	Mv	PD
1	590902	434141	786329	1306177	1900016	720854	1.81123

Processed Peaks

Peak No	Name	Start RT (mins)	Max RT (mins)	End RT (mins)	Pk Height (mV)	% Height	Area (mV.secs)	% Area
1		11.07	12.48	14.02	-60.9027	100	4704.72	100

Supplementary Figure 194. GPC data of the polymer from supplementary table 1, entry 3.

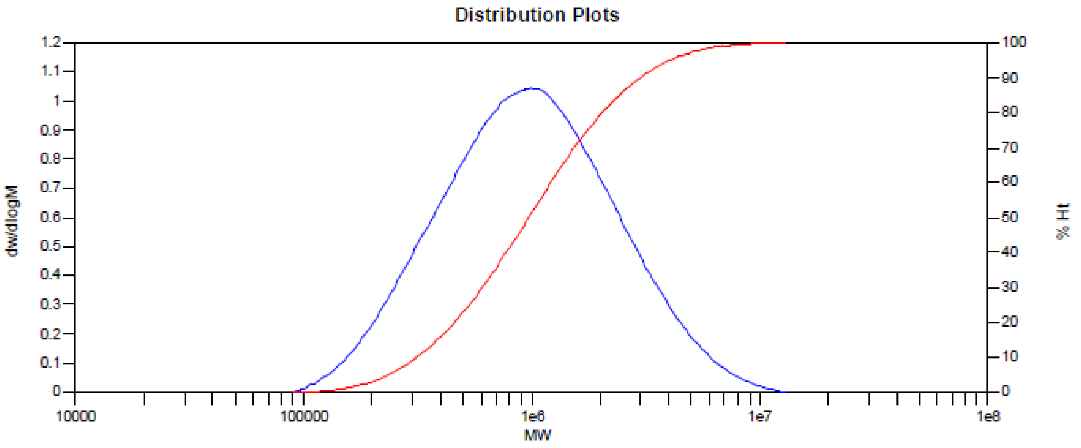

MW Averages

Peak No	Mp	Mn	Mw	Mz	Mz+1	Mv	PD
1	995707	674229	1377228	2640683	4246689	1233627	2.04267

Processed Peaks

Peak No	Name	Start RT (mins)	Max RT (mins)	End RT (mins)	Pk Height (mV)	% Height	Area (mV.secs)	% Area
1		10.50	12.15	13.70	-52.3911	100	4419.77	100

Supplementary Figure 195. GPC data of the polymer from supplementary table 1, entry 4.

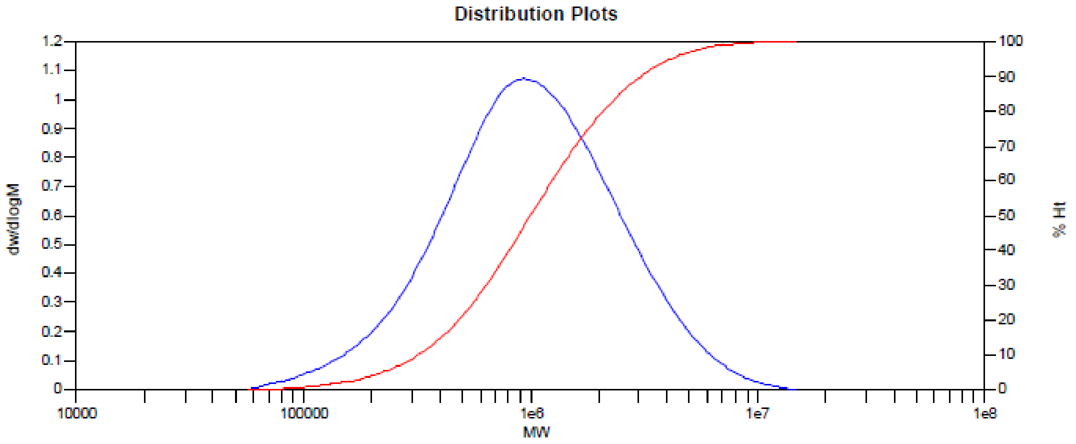

MW Averages

Peak No	Mp	Mn	Mw	Mz	Mz+1	Mv	PD
1	945011	661592	1415305	2734344	4474499	1266439	2.13924

Processed Peaks

Peak No	Name	Start RT (mins)	Max RT (mins)	End RT (mins)	Pk Height (mV)	% Height	Area (mV.secs)	% Area
1		10.42	12.18	14.02	-64.1101	100	5275.29	100

Supplementary Figure 196. GPC data of the polymer from supplementary table 1, entry 5.

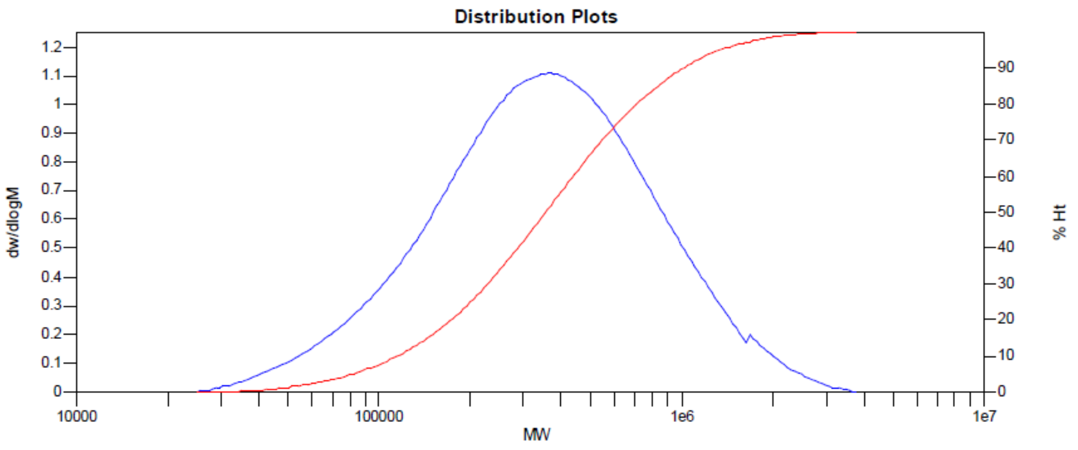

MW Averages

Peak No	Mp	Mn	Mw	Mz	Mz+1	Mv	PD
1	370293	236996	472475	843461	1296596	427905	1.9936

Processed Peaks

Peak No	Name	Start RT (mins)	Max RT (mins)	End RT (mins)	Pk Height (mV)	% Height	Area (mV.secs)	% Area
1		11.30	12.78	14.58	-57.9508	100	4660.25	100

Supplementary Figure 197. GPC data of the polymer from supplementary table 1, entry 6.

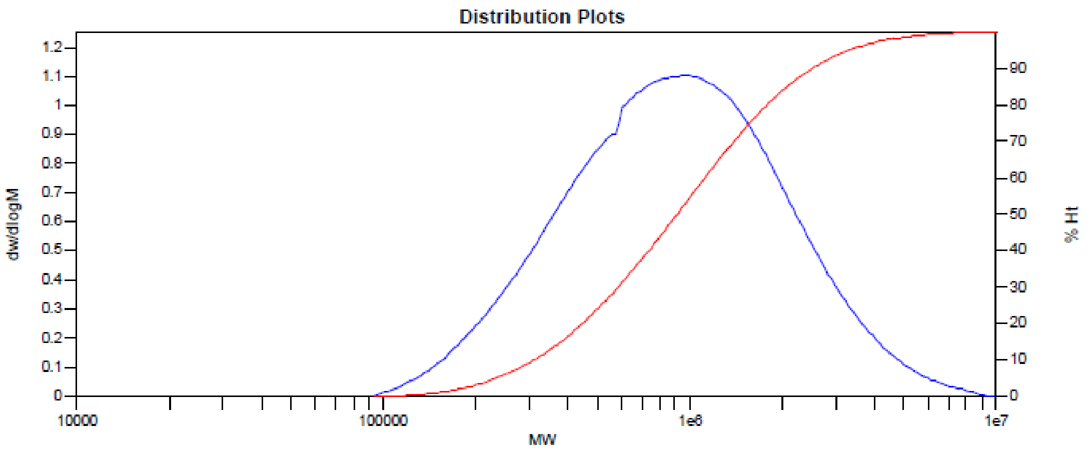

MW Averages

Peak No	Mp	Mn	Mw	Mz	Mz+1	Mv	PD
1	970027	647209	1206040	2107116	3241897	1098196	1.86345

Processed Peaks

Peak No	Name	Start RT (mins)	Max RT (mins)	End RT (mins)	Pk Height (mV)	% Height	Area (mV.secs)	% Area
1		10.67	12.18	13.68	-45.1869	100	3609.85	100

Supplementary Figure 198. GPC data of the polymer from supplementary table 1, entry 7.

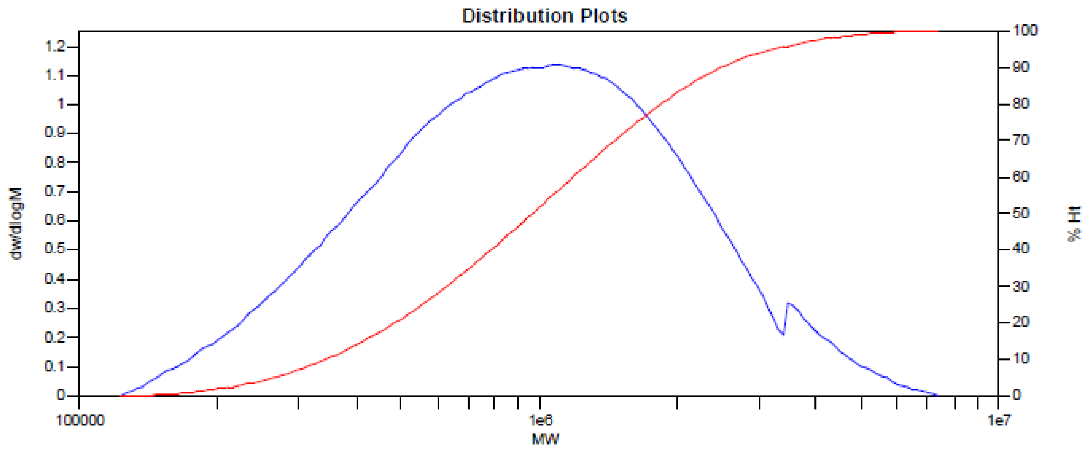

MW Averages

Peak No	Mp	Mn	Mw	Mz	Mz+1	Mv	PD
1	1105438	703870	1226295	1978616	2819929	1129837	1.74222

Processed Peaks

Peak No	Name	Start RT (mins)	Max RT (mins)	End RT (mins)	Pk Height (mV)	% Height	Area (mV.secs)	% Area
1		10.87	12.10	13.50	-19.9098	100	1542.42	100

Supplementary Figure 199. GPC data of the polymer from supplementary table 1, entry 8.

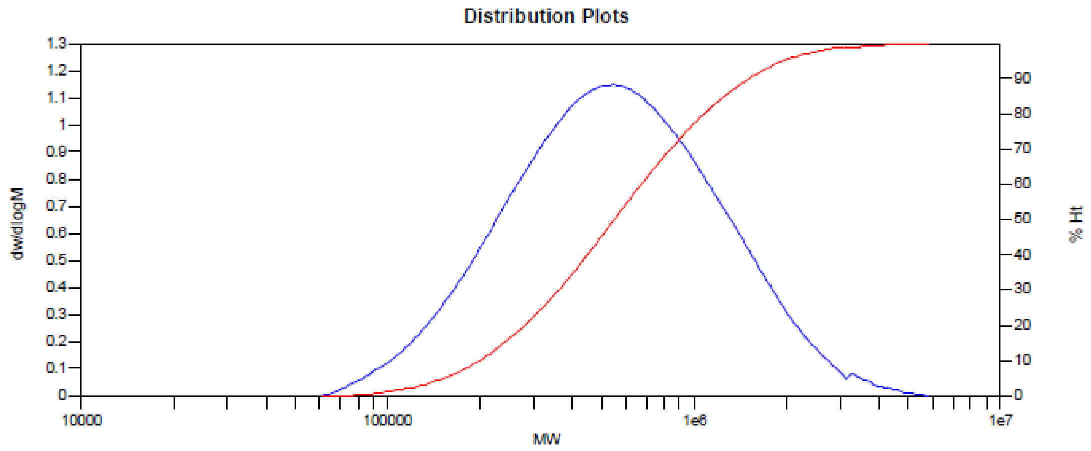

MW Averages

Peak No	Mp	Mn	Mw	Mz	Mz+1	Mv	PD
1	546521	401970	721374	1233469	1884676	659844	1.7946

Processed Peaks

Peak No	Name	Start RT (mins)	Max RT (mins)	End RT (mins)	Pk Height (mV)	% Height	Area (mV.secs)	% Area
1		11.02	12.53	13.97	-58.4204	100	4494.08	100

Supplementary Figure 200. GPC data of the polymer from supplementary table 1, entry 9.

Supplementary Tables

Supplementary Table 1. Ethylene homopolymerization with Ni complexes.[a]

Ent.	Cat.	*T*(°C)	P/atm	Yield (g)	Act.[b]	M_n[c] (10^4)	PDI[c]	*B*[d]	T_m(°C)[e]
1	Ni5	20	8	1.0	2.0	55.5	2.0	12	122.8
2	Ni5	50	8	1.1	2.2	81.9	2.0	19	116.5
3	Ni5	80	8	0.8	1.6	43.4	1.8	23	111.8
4	Ni6	20	8	0.8	1.6	67.4	2.0	13	121.8
5	Ni6	50	8	1.3	2.6	66.2	2.1	15	115.3
6	Ni6	80	8	0.9	1.8	23.7	2.0	23	110.8
7	Ni7	20	8	1.1	2.2	64.7	1.9	5	129.5
8	Ni7	50	8	2.0	4.0	70.3	1.7	7	126.7
9	Ni7	80	8	0.6	1.2	40.2	1.8	8	124.7

[a]Polymerization conditions: catalyst = 1 μmol; toluene = 28 mL, CH_2Cl_2 = 2 mL, time = 3 min. [b]The yields and activities are average of at least two runs. Activity is in unit of 10^7 g·mol^{-1}·h^{-1}. [c]Determined by GPC in trichlorobenzene at 150 °C. [d]B = branches per 1000 carbons, determined by ^{1}H NMR spectroscopy. [e]Determined by differential scanning calorimetry (DSC).

Supplementary Table 2. Polymer yield as a function of time for **Ni-OH@SiO2** and **Ni-OH** in ethylene polymerization.[a]

Ent.	Cat.	*T*(°C)	Time(min)	P/atm	Yield[b] (g)	Yield[c] (g)	Yield[d] (g)
1	Ni-OH	100	10	8	0.39	0.41	0.4
2	Ni-OH	100	30	8	0.52	0.48	0.5
3	Ni-OH	100	60	8	0.50	0.54	0.52
4	Ni-OH	100	120	8	0.56	0.54	0.55
5	Ni-OH@SiO_2	100	10	8	0.25	0.35	0.3
6	Ni-OH@SiO_2	100	30	8	0.82	0.78	0.8
7	Ni-OH@SiO_2	100	60	8	1.32	1.18	1.25
8	Ni-OH@SiO_2	100	120	8	1.8	1.6	1.7

[a]homogeneous polymerization conditions: ethylene = 8 atm, **Ni-OH** = 1 μmol, heptane = 29 mL, DCM = 1 mL; heterogeneous polymerization conditions: ethylene = 8 atm, **Ni-OH@SiO2** = 1 μmol catalyst supported on 100 mg of SiO_2, heptane = 30 mL. [b]The yields are weighed of first run. [c]The yields are weighed of second run. [d]The yields are average of two runs.

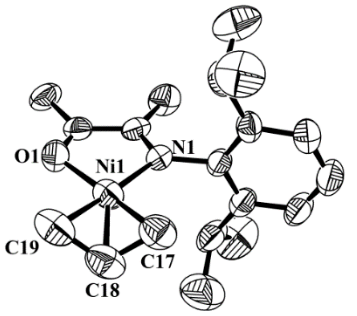

Supplementary Table 3. Crystal data and structure refinement for Ni1. Hydrogen atoms and the $BAr^F{}_4$ groups were omitted for clarity. Selected bond lengths (Å) and angles (°): Ni1-N1 = 1.925(3), Ni1-O1 = 1.946(3), Ni1-C17 = 1.98(8), Ni1-C18 = 1.916(18), Ni1-C19 = 1.95(7), N1-Ni1-O1 = 82.05(13), C19-Ni1-C17 = 74(3).

Formula	$C_{51}H_{40}BF_{24}NNiO$
Formula weight	1208.36
Temperature[K]	298(2)
λ(Mo-Kα)[Å]	0.71073
Crystal system	Monoclinic
Space group	P2(1)/c
a[Å]	17.6429(15)
b[Å]	12.9630(11)
c[Å]	27.503(2)
α[°]	90.00
β[°]	118.950(4)
γ[°]	90.00
Volume[$Å^3$]	5504.1(8)
Z	4
D(calc)[$g \cdot cm^{-3}$]	1.458
μ[mm^{-1}]	0.471
F(000)	2440
θ min-max (°)	2.347-26.005
h	-20→19
k	-15→12
l	-31→32
Reflections collected	27586
Reflections unique	9701
R(int)	0.0650
Data / restraints / parameters	4299 / 0 / 967
Final R indices [I>2σ(I)]	R_1 = 0.0499<br>wR_2 = 0.0499
R indices (all data)	R_1 = 0.1411<br>wR_2 = 0.1252
GOF on F^2	0.930

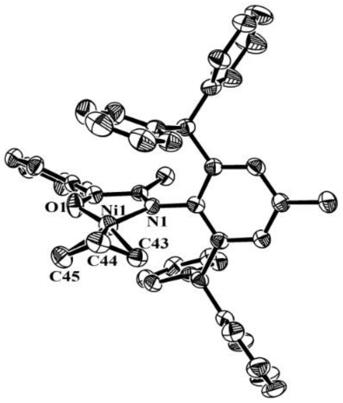

Supplementary Table 4. Crystal data and structure refinement for Ni6. Hydrogen atoms and the $BAr^F{}_4$ groups were omitted for clarity. Selected bond lengths (Å) and angles (°): Ni1-C44 = 1.921(14), Ni1-O1 = 1.923(4), Ni1-N1 = 1.942(4), Ni1-C43 = 2.01(5), Ni1-C45 = 2.03(2), O1-Ni1-N1 = 81.29(16), C43-Ni1-C45 = 72.3(17).

Formula	C78H54BCl2F24NNiO
Formula weight	1617.64
Temperature[K]	298(2)
λ(Mo-Kα)[Å]	0.71073
Crystal system	Triclinic
Space group	P-1
a[Å]	14.1200(12)
b[Å]	16.1501(14)
c[Å]	17.7202(15)
α[°]	95.010(2)
β[°]	101.091(3)
γ[°]	102.728(3)
Volume[$Å^3$]	3832.3(6)
Z	2
D(calc)[$g \cdot cm^{-3}$]	1.402
μ[mm^{-1}]	0.425
F(000)	1640
θ min-max (°)	*2.715*- 26.122
h	-16-9
k	-19-19
l	-20-21
Reflections collected	19547
Reflections unique	13308
R(int)	0.0506
Data / restraints / parameters	13308/0/1143
Final R indices [I>2σ(I)]	R_1 =0.0782<br>wR_2 = 0.1841
R indices (all data)	R_1 = 0.1602<br>wR_2 = 0.2171
GOF on F^2	1.034

Supplementary References.

1. Abakumov, G. A., Cherkasov, V. K., Druzhkov, N. O., Kocherova, T. N. & Shavyrin, A. S. New polydentate ligands based on sterically hindered o-benzoquinones (pyrocatechols) containing the 1,4-diazadiene group. *Russ. Chem. Bull., Int. Ed.* **60**, 112-117 (2011).
2. Dai, S. Y., Zhou, S. X., Zhang, W. & Chen, C. L. Systematic Investigations of Ligand Steric Effects on α-Diimine Palladium Catalyzed Olefin Polymerization and Copolymerization. *Macromolecules* **49**, 8855-8862 (2016).
3. Wang, R. K., Sui, X. L., Pang, W. M. & Chen, C. L. Ethylene Polymerization by Xanthene-Bridged Dinuclear α-Diimine Ni^{II} Complexes. *ChemCatChem* **8**, 434-440 (2016).
4. Tomita, R., Koike, T. & Akita, M. Photoredox-Catalyzed Stereoselective Conversion of Alkynes into Tetrasubstituted Trifluoromethylated Alkenes. *Angew. Chem., Int. Ed.* **54**, 12923-12927 (2015).
5. Mi, C., Li, L., Meng, X.-G., Yang, R.-Q. & Liao, X.-H. Highly Selective Oxidation of Unsaturated Hydrocarbons to Carbonyl Compounds by Two-Phase Catalysis. *Tetrahedron* **72**, 6705-6710 (2016).
6. Chang, C.-L., Kumar, M. P. & Liu, R.-S. A Highly Efficient Ruthenium-Catalyzed Rearrangement of α,β-Epoxyketones to 1,2-Diketones. *J. Org. Chem.* **69**, 2793-2796 (2004).
7. Rahimi, A., Ulbrich, A., Coon, J. J. & Stahl, S. S. Formic-acidinduced depolymerization of oxidized lignin to aromatics. *Nature* **515**, 249-252 (2014).
